# Elucidating the fundamental forces in protein crystal formation: the case of crambin†
†Electronic supplementary information (ESI) available: Detailed computational details; description of the methodology used to compute the BSSE-corrected crystal formation energies and charge transfers; supplementary tables including energetics obtained with different functional, basis set, dispersion correction; coordinates (in PDB format) of the crambin protein molecule in its optimized gas phase geometry and as found in the three optimized 0W, 84W, 172W crystals; coordinates (in CIF format) of the three optimized 0W, 84W and 172W crambin crystals. See DOI: 10.1039/c5sc03447g


**DOI:** 10.1039/c5sc03447g

**Published:** 2015-11-24

**Authors:** Massimo Delle Piane, Marta Corno, Roberto Orlando, Roberto Dovesi, Piero Ugliengo

**Affiliations:** a Department of Chemistry and NIS Centre , University of Torino , via Pietro Giuria 7 , 10125 , Torino , Italy . Email: piero.ugliengo@unito.it

## Abstract

Molecular simulations of proteins have been usually accomplished through empirical or semi-empirical potentials, due to the large size and inherent complexity of these biological systems. On the other hand, a theoretical description of proteins based on quantum-mechanical methods would however provide an unbiased characterization of their electronic properties, possibly offering a link between these and the ultimate biological activity. Yet, such approaches have been historically hindered by the large amount of requested computational power. Here we demonstrate the feasibility of periodic all-electron density functional theory calculations in the description of the crystal of the protein crambin (46 aminoacids), which is determined with exceptional structural accuracy. We have employed the hybrid B3LYP functional, coupled to an empirical description of London interactions (D*) to simulate the crambin crystal with an increasing amount of lattice water molecules in the cell (up to 172H_2_O per cell). The agreement with the experiment is good for both protein geometry and protein–water interactions. The energetics was computed to predict crystal formation energies, protein–water and protein–protein interaction energies. We studied the role of dispersion interactions which are crucial for holding the crambin crystal in place. B3LYP-D* electrostatic potential and dipole moment of crambin as well as the electronic charge flow from crambin to the solvating water molecules (0.0015*e* per H_2_O) have also been predicted. These results proved that quantum-mechanical simulations of small proteins, both free and in their crystalline state, are now feasible in a reasonable amount of time, by programs capable of exploiting high performance computing architectures, allowing the study of protein properties not easily amenable through classical force fields.

## Abstract 2

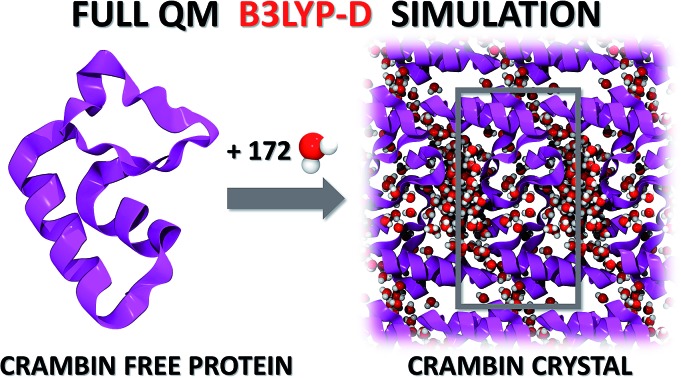
This study demonstrates the feasibility of periodic all-electron hybrid density functional theory calculations in the description of protein crystals, using crambin as a test case.

## Introduction

Most of our knowledge on the structure and behavior of proteins, the fundamental building blocks of life, derives from the continuous interplay between experimental data and simulations. Indeed, the latter often “give life” to the former by providing a favored view on the microscopic processes that may happen *in vivo*.

Due to the large size of the required models (>10^3^ to 10^4^ atoms), simulations of proteins have been historically limited to those based on empirical potentials (*i.e.* classical molecular mechanics). The steadily increasing available computational power has now broadened the scope of such classical simulations to models containing millions of atoms,^1^,^2^ with a record-breaking simulation of more than 60 million atoms recently reported in literature (HIV-1 capsid).^3^ Contemporarily, quantum mechanical semiempirical methods have also become competitive for the simulation of small/medium sized proteins, due to linear-scaling algorithms and parallelization.^4^

At the other end of the spectrum, purely quantum-mechanical simulations have commonly been considered feasible for systems up to hundreds of atoms,^1^ too small a size for the majority of biological systems. Therefore, the most common application of quantum-mechanics to the study of proteins is the QM/MM approach, through which only a small part of the system (*e.g.* the active site of an enzyme) is treated quantum-mechanically (QM) while the remaining part is simulated through a classical approach (MM).^5^ However, recent evolutions in High Performance Computing (HPC) architectures^6^ (also thanks to the introduction of machines based on Graphical Processing Units, GPUs) and the concurrent development of more efficient (in terms of exploited computational power and reduction of memory consumption) quantum-mechanical software^7^–^11^ have dramatically increased the size and complexity of the systems that can be modeled by fully *ab initio* methods. This is important, as a quantum-mechanical based approach for protein modeling would provide unique information not available through a classical, force-field based, approach.^12^,^13^ For instance, the availability of the electron density allows to rigorously describe polarizability and charge transfer in proteins, not easily amenable from classical force-fields. Even more important is the natural approach to chemical reactivity of these methods, with the description of bond breaking and formation.^14^

Nevertheless, the quantum-mechanical simulation of proteins is still in its early stages. The first pioneeristic fully *ab initio* protein simulation dates back to 1998, in which the geometry optimization of the isolated crambin molecule (642 atoms) was carried out at the Hartree–Fock level (HF/4-21G) by van Alsenoy *et al.*^15^ In the later years, some fully Density Functional Theory (DFT) based simulations of proteins have been reported,^10^,^14^,^16^,^17^ albeit only in limited cases the geometry has been optimized.^14^ Kulik *et al.* performed molecular geometry optimizations of 58 protein molecules (from 70 up to 590 atoms), with different *ab initio* methods, both HF and DFT (benchmarking different functionals, also hybrids) and different basis sets (STO-3G, MINI, 3-21G, 6-31G). They reported an average backbone RMSD with respect to the experiment between 0.57 and 0.77 Å, depending on the adopted approach, comparable to results obtained with highly parameterized force-fields.^14^ More recently, Lever *et al.* managed to perform a transition state search on the chorismate mutase enzyme (about 2000 atoms) by treating the whole molecule quantum-mechanically (PBE functional with a minimal set of strictly localized functions).^10^ As regards *ab initio* molecular dynamics (AIMD) simulations beyond the traditional QM/MM approach, Ufimtsev *et al.* reported the trajectory of the 900-atoms bovine pancreatic trypsin inhibitor, with only the surrounding water treated classically.^18^ Actually, some authors have reported “unsurmountable difficulties” in treating proteins with DFT due to lack of convergence of the self consistent field, ascribed to vanishing HOMO–LUMO gaps (particularly with non-hybrid functionals).^14^,^19^ To overcome such issues and reduce the computational time requested by a full treatment of protein structures, some researchers have proposed and applied several “divide & conquer” techniques, in which small protein portions are separately treated quantum-mechanically and the results are then merged through various approaches.^20^–^26^

To the best of our knowledge, the present work reports, for the first time, a fully hybrid DFT crystal structure geometry optimization and characterization of a protein, inclusive of lattice solvating water molecules. We have successfully applied the Becke, 3 parameters, Lee–Yang–Parr (B3LYP) hybrid functional,^27^ augmented with the Grimme's contribution to describe vdW interactions (D*).^28^ Despite the enormous development in new and sophisticated functionals, B3LYP is still recognized as one of the most widely used and well balanced functionals as for its accuracy, due to the large database of results accumulated over the years.^29^

As a test case, we chose crambin, a thionin protein found in the seeds of the plant *Crambe abyssinica*.^30^ It is a small (46 aminoacids), highly hydrophobic and water insoluble protein^31^ which has been extensively studied both experimentally^30^–^38^ and computationally.^15^,^16^,^25^,^39^–^45^ The reasons of our choice are twofold. First of all, since long ago its crystal structure has been solved by diffraction techniques up to an atomic resolution of 0.48 Å, close to that of small molecules.^33^ This has recently allowed determining the position of a large number of solvating water molecules belonging to the first and second solvation shells. The second reason to focus on crambin is that, although it is one of the smallest known proteins, it still exhibits a distinct secondary structure, with both α-helices and β-sheets, thus being representative of the main features found in larger proteins. The first recorded modelization of crambin is a full optimization of its crystal structure through classical potential by Jorgensen *et al.*, in 1988.^39^ Nine years later, Stewart was able to optimize the geometry of free crambin through the PM3 semi-empirical method.^46^ The following year, it became the first free protein fully treated *ab initio*, in the aforementioned HF simulation by van Alsenoy *et al.*^15^ A DFT calculation, albeit without optimization, was reported some years ago,^16^ while more recently its Raman spectrum was computed through HF within the fragment molecular orbital method.^25^ None of the quantum-mechanical simulations on crambin, however, included an explicit description of water surrounding the protein, both in solution and in its crystal. Since water is essential to protein activity, its inclusion in simulations has proven essential to reproduce actual conditions and behavior.^32^ In the present work, we deal with both the free crambin molecule and its crystal structure at different degrees of lattice water solvation, providing the structure, the energetics of the crambin–crambin, water–crambin and water–water interactions as well as the electrostatic potential, crambin dipole moment and the charge flux between crambin and solvating water molecules.

## Computational details

The development version of the CRYSTAL14 code^47^ was adopted for all the calculations, performed within the density functional theory, adopting the Becke, 3 parameters, Lee–Yang–Parr (B3LYP) hybrid functional.^27^ A split valence double-ζ basis set (6-31G(d)) plus polarization functions was applied to C, N, S and O,^48^ while an Ahlrichs's pVDZ basis was used for H.^49^ For the sake of simplicity, the chosen combination of Hamiltonian and basis will be identified as B3LYP-D*/6-31G(d,p) in the following. For the periodic cases, the Hamiltonian matrix was diagonalized only at the *Γ* point, due to the large volume of the unit cells.^50^ The dispersion contribution was added to the DFT energies and gradients, by means of the empirical dispersion correction originally proposed by Grimme and known as D2 correction,^51^ with the modified parameters proposed by Civalleri *et al.* for the treatment of molecular crystals using the B3LYP hybrid functional (hereafter referred as B3LYP-D*), correcting for the known overbinding issue of the standard D2 implementation.^28^ The B3LYP-D* approach has been found to produce an excellent agreement for cohesive energies and structures of a representative set of molecular crystals (mean absolute deviation on cohesive energies: 6.3 kJ mol^–1^).^28^ Both cell parameters and internal coordinates were optimized using the analytical gradient method. A more complete description of the computational parameters is included in the ESI.†

As anticipated in the introduction, we modeled three crambin crystals differing for the amount of solvating water, namely with 0W, 84W and 172W water molecules (vide infra) resulting in 1284, 1536 and 1800 atoms and 12 354, 14 370 and 16 482 Atomic Orbitals (AOs) in the unit cell, respectively.

The energetics of the protein crystal and of the protein–protein and protein–water interactions was decomposed in various contributions as in ref. 52 and detailed in the ESI.† All energies have been corrected for the Basis Set Superposition Error (BSSE), using the same counterpoise method adopted for intermolecular complexes.^53^ For some processes of Table 3, a B3LYP-D*/6-31G(d,p) fully optimized water box (84 molecules; side: 13.4 Å) representing liquid water was taken as a reference.

For comparison, some single point energy calculations on the B3LYP-D*/6-31G(d,p) geometries were performed with the Heyd, Scuseria and Ernzerhof 06 (HSE06) hybrid functional,^54^,^55^ while the Grimme's D3 dispersion correction^56^ was also tested to compare with the D* results.

### Hardware and performances

All calculations were run on a Cray Cascade XC40 Supercomputer. CRYSTAL14 was used in its most recent massively parallel version, that allows treatment of very large unit cells for crystalline systems, like the ones described in the present paper, on HPC architectures with high efficiency in terms of CPU time and memory requirements.^7^ An average optimization step (energy + gradient calculation), at the aforementioned all-electron B3LYP-D*/6-31G(d,p) level of theory, for the largest studied system (172W) required about 23 minutes on 1920 cores, while ∼5 minutes on 720 cores were required for an isolated crambin molecule (642 atoms, 7185 atomic orbitals). As a comparison, the average optimization step of the molecular structure of γ-chymotrypsin (PDB: ; 8GCH),^57^ composed by 4 chains, 244 aminoacids, 349H_2_O molecules for a total of 4575 atoms, took about 3 hours on 1200 cores. Finally, the computational cost of one-electron properties is very low in the current CRYSTAL14 implementation:^58^ the electrostatic potential of the crambin molecule was computed in less than a minute on 256 Intel Ivy-Bridge cores, using the latest parallel implementation of the CRYSTAL14 properties portion of the code.

## Results and discussion

### Crambin crystal models

All the results in this paper were obtained starting from the experimental crambin geometry resolved by Chen *et al.* (PDB: ; 4FC1, space group: *P*2_1_, 2 proteins per cell).^59^ The authors have obtained the 1.1 Å, ultrahigh resolution neutron diffraction structure of hydrogen/deuterium (H/D) exchanged crambin, with 94.9%, of the hydrogen atom positions resolved. Furthermore, they have been able to accurately determine a number of well-ordered solvent water molecules in the first and second hydration shells (42 irreducible water molecules, 84 symmetry related in total). Although experimental structures at higher resolutions are available (also with refined position of solvent molecules),^33^ we chose ; 4FC1 since, at variance with other sources, the deposited PDB file contains all hydrogen positions and all water molecules having unity occupancy factor, which is an essential requirement for the simulations. We further validated our starting point by checking the backbone Root Mean Square Deviation (RMSD) in Å with respect to two other deposited structures resolved at higher resolution through X-ray diffraction, ; 1EJG (0.54 Å)^60^ and ; 3NIR (0.48 Å).^33^ These RMSDs were 0.127 and 0.148 Å, respectively. When multiple occupancies of specific protein atoms were present in the experimental structure, we always chose those with the highest occupancy factor.

**Fig. 1:**
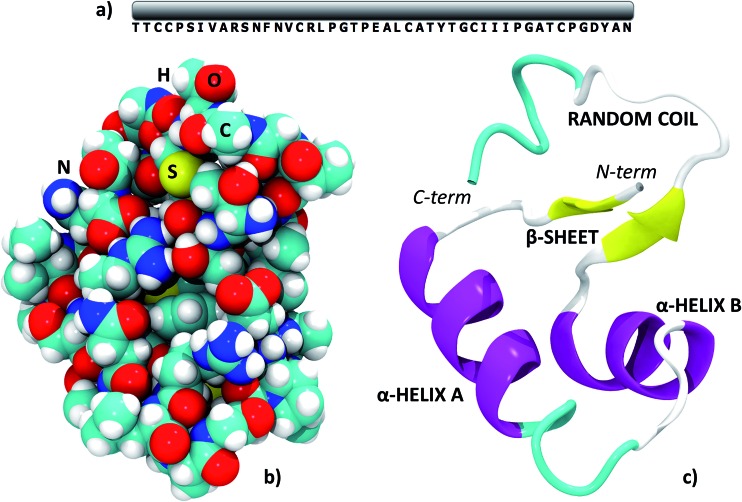
The structure of crambin. (a) Primary structure; (b) space-filled 3D model; (c) secondary structure with indications of the main domains. PDB structure: 4FC1.

Crambin is a thionin plant protein consisting of 46 aminoacids, whose sequence is reported in Fig. 1a. Apolar residues constitute about two thirds of the protein and this is reflected in its hydrophobic character. Crambin is globally neutral: apart from the C- and N-termini, only four residues are charged (Arg10, Arg17, Glu23, Asp43) and are clustered together. Furthermore, the six cysteines form three di-sulfide bonds that stabilize the structure. Crambin exists in two sequence isomers: the PL form has Pro and Leu at positions 22 and 25, while the SI form has Ser and Ile at the same positions.^35^ Both the experimental structure and all models of this paper refer to the PL form. Fig. 1b and c reports a space-filled view and the secondary structure of the experimental crambin. The protein contains two antiparallel α-helices and a short β-sheet near the N-terminus, while the area close to the C-terminus is a random coil.

**Fig. 2:**
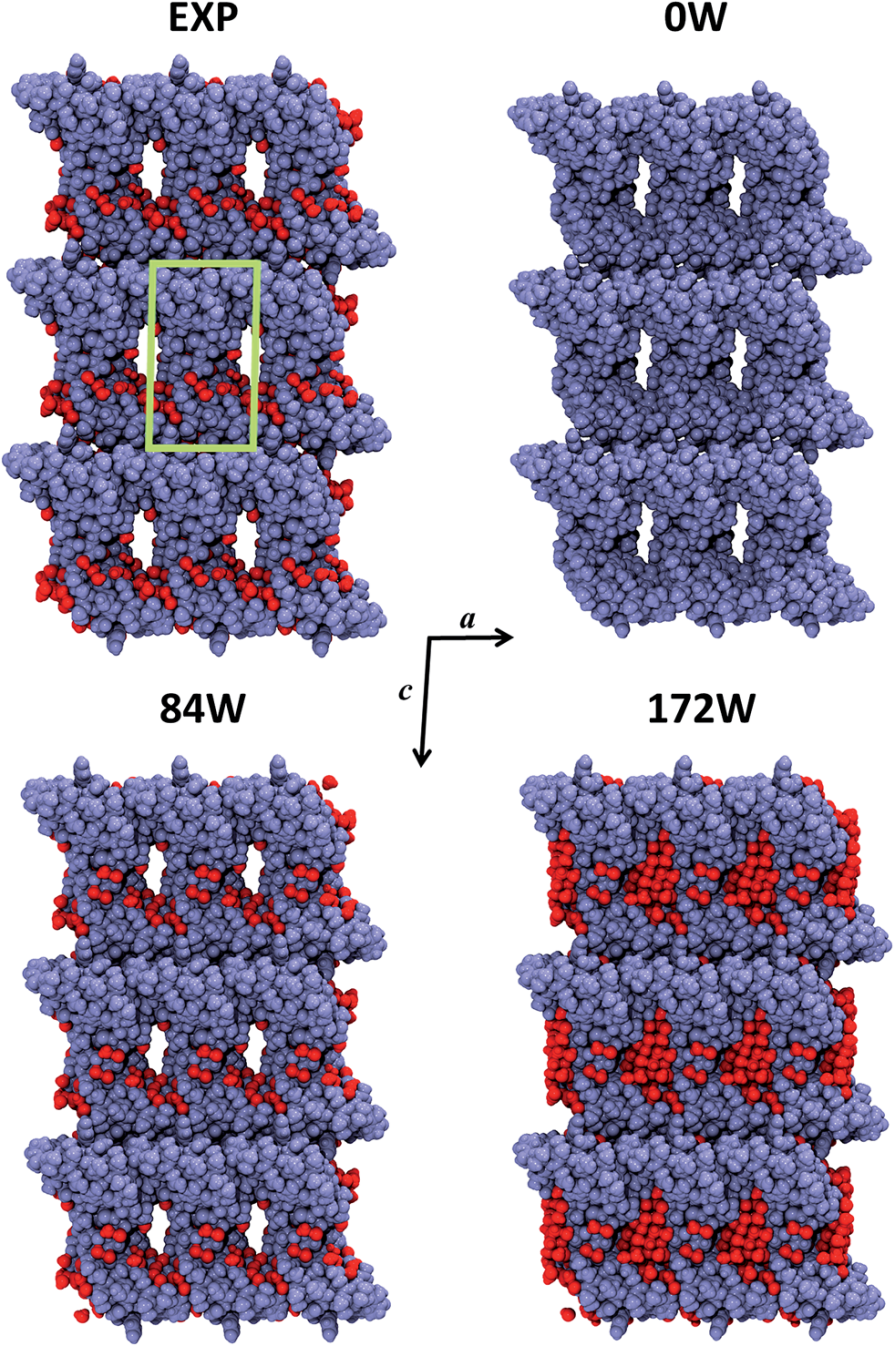
Crystal portion views of crambin. In all cases, a space-filled 3D view of a crystal box corresponding to 3 × 3 × 3 unit cells is reported (only complete molecules are shown), along the *b* axis. Color code: purple, protein atoms; red, water atoms; green, cell borders. EXP: experimental crystal structure (PDB: ; 4FC1); note that in this case only the 84 well-determined water molecules are shown, while the experimental crystal has a 30% water content (v/v). *n*W: B3LYP-D*/6-31G(d,p) optimized crambin crystals with *n*H_2_O per cell included in the simulation.

Crambin forms exceptionally well-ordered crystals.^33^ The experimentally determined crambin crystal structure is shown in Fig. 2 (EXP). Each unit cell contains two equivalent protein molecules (purple, in figure) related by the *P*2_1_ space symmetry.

The two molecules interact mainly through van der Waals (vdW) interactions, excluding most of the water molecules from the area of interaction.^37^Fig. 2 (EXP) also reports the 84 experimentally defined positions of water molecules (red). These solvent molecules represent only a fraction of the total water content in the experimental crystal, that is estimated as ∼30% in volume.^34^ Based on the experimental data, the total number of expected water molecules has been estimated in literature to be 170,^34^ 162 38 or 182,^36^ while Jorgensen *et al.* simulated the crambin crystal with a total of 182 water molecules in the cell. Regardless of the exact total number, most of this “missing” water would locate inside the channel made up by four symmetry-related protein molecules facing one another, as can be observed from the crystal packing of Fig. 2.^34^ This ellipsoid type channel is about 13 × 8 Å wide and extends infinitely along the *b* direction.^34^

Due to these considerations and with the aim of studying the role of water in a protein crystal, three periodic models of the crambin crystal were generated. These models are reported in Fig. 2. The first model, named 0W, represents a crambin crystal from which all water molecules have been removed. The 84W model is a crambin crystal hydrated by the 84 crystallographically determined water molecules only. Finally, the 172W model is representative of a real crambin crystalline sample, complete with all solvent molecules. To obtain this model, we started from the 84W structure and we used the PACKMOL utility^61^ and the MOLDRAW molecular visualization program^62^ to add a cylinder of 70 water molecules (radius = 6 Å, length along the *b* direction = 18 Å) inside the solvent channel per unit cell. We partially optimized the structure and manually added more water to fill in any remaining empty space; we repeated the process until we reached the final 172 total water molecules in the unit cell. The three 0W, 84W and 172W models contain 1284, 1536, 1800 atoms (12 354, 14 370, 16 482 AOs) in the unit cell respectively. For 0W and 84W the *P*2_1_ symmetry was conserved, while the 172W model was necessarily treated within *P*1 space group, as any symmetry beside identity was lost in the generation process.

### Geometry optimizations

The 0W, 84W and 172W crambin crystal models were fully (both cell parameters and atomic coordinates) optimized at the chosen B3LYP-D*/6-31G(d,p) level of theory. Convergence in the total energy calculation was easily achieved, using the default parameters of the CRYSTAL14 code.

Fig. 2 shows the final optimized geometries, viewed along the *b* axis, of the three 0W, 84W and 172W models, while Table 1 reports the experimental and calculated cell parameters. In all cases, we see a significant shrinking of the unit cell volume. However, increasing the water content greatly improves the results. In the 0W case a dramatic 20% shrinking is observed with significant modifications of the crystal structure: Fig. 2 shows that protein molecules move closer to each other, with a concurrent impending occlusion of the empty solvent pore. Including the 84 crystallographic water molecules (84W) reduces the volume shrinking at 12%, with a deformation of the cell due to the missing water inside the pore. As expected, the optimized fully hydrated crystal structure (172W) is the closest to the experiment, although a less than 10% volume shrinking is still present. This shrinking is equally distributed along the three crystallographic directions. Actually this underestimation reduces to 5% if compared with experimental crystal structures obtained at 100 K,^33^,^60^ since our static simulations do not take into account any thermal effect, that is likely to induce a temperature-dependent increase in unit cell volume. Indeed, our reference experimental crystal was measured at 290 K 59 and its volume (17 611 Å^3^) is the highest among deposited crambin crystal structures (average volume 16 984 Å^3^). Furthermore, it has been recently found that Zero Point Energy (ZPE) contributions (not included in our simulations) have a significant influence on crystal cell volumes. For instance, in the case of ice, this effect has been computed as a 0.5–5.5% increase, depending on the polymorph.^63^ The remaining shrinking can be due to several other effects that are expected to work in the same direction. First of all, our simulations surely include an overbinding due to the BSSE (both inter- and intra-molecular), caused by the relatively small basis set (see “Crystal formation energies” section for further comments on the applied methodology). Moreover, missing or misplaced crystal water molecules may still have an influence on the simulation unit cell volume.

**Table 1:** Cell parameters of crystalline crambin^*a*^

	*a*	*b*	*c*	*α*	*β*	*γ*	Vol.
EXP	22.795	18.826	41.042	90.00	90.89	90.00	17 610.6
0W	21.027	17.356	38.545	90.00	89.61	90.00	14 065.9
*Change*	*–7.8%*	*–7.8%*	*–6.1%*	*0.0%*	*–1.4%*	*0.0%*	*–20.1%*
84W	21.741	17.852	39.862	90.00	89.90	90.00	15 471.7
*Change*	*–4.6%*	*–5.2%*	*–2.9%*	*0.0%*	*–1.1%*	*0.0%*	*–12.2%*
172W	22.116	18.106	39.732	90.00	90.78	90.03	15 908.4
*Change*	*–3.0%*	*–3.8%*	*–3.2%*	*0.0%*	*–0.1%*	*0.03%*	*–9.7%*

^*a*^All changes are in % with respect to experiment (EXP). Data in Å. EXP: experimental (PDB: 4FC1); 0W: B3LYP-D*/6-31G(d,p) optimized with 0H_2_O per cell; 84W: B3LYP-D*/6-31G(d,p) optimized with 84H_2_O per cell; 172W: B3LYP-D*/6-31G(d,p) optimized with 172H_2_O per cell. For 0W and 84W the original space group (*P*2_1_) has been maintained, while for 172W symmetry has been reduced to *P*1.

**Fig. 3:**
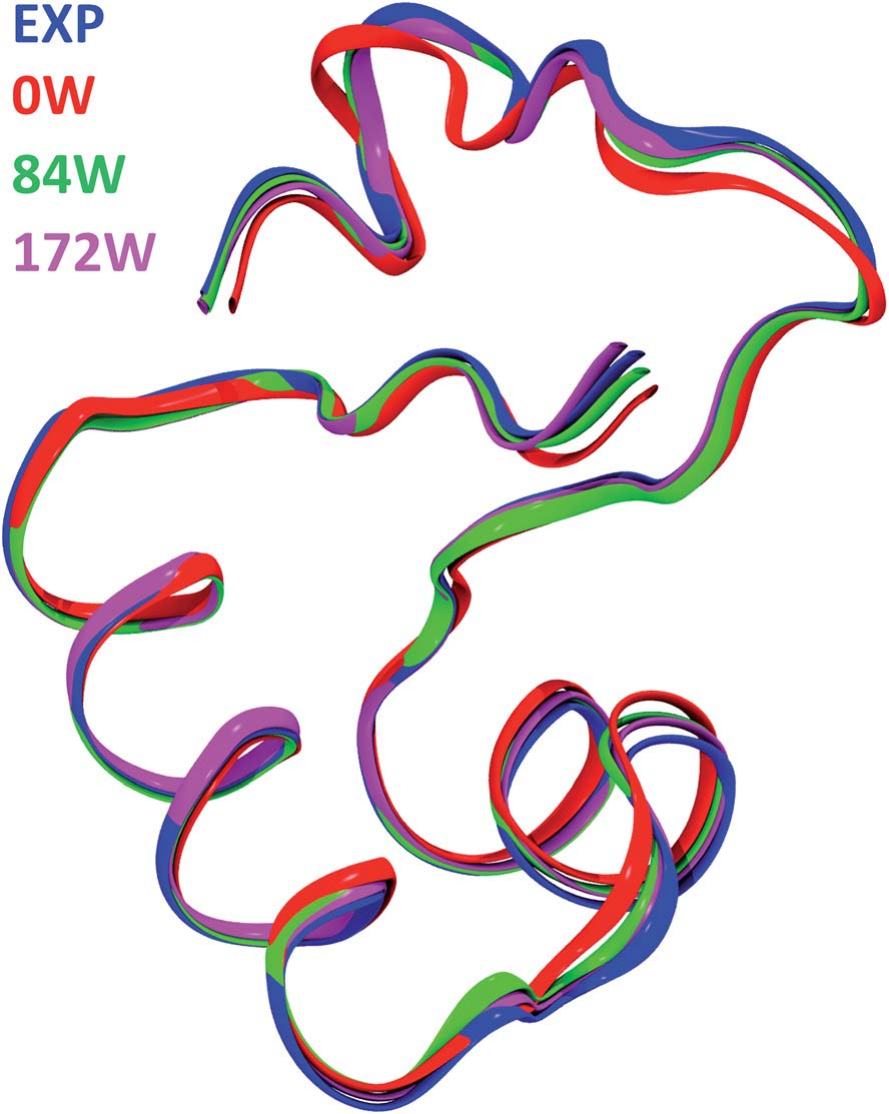
Comparison of crambin structures. Backbone as a ribbon representation. Color code: blue, experimental crystal structure (PDB: 4FC1); red, B3LYP-D*/6-31G(d,p) optimized crambin crystals with 0H_2_O per cell; green, B3LYP-D*/6-31G(d,p) optimized crambin crystals with 84H_2_O per cell, magenta, B3LYP-D*/6-31G(d,p) optimized crambin crystals with 172H_2_O per cell.

Fig. 3 shows a superposition of the backbones of all studied models, together with the experimental structure, while Table 2 includes the computed Root Mean Square Deviations (RMSD) in Å with respect to the experiment. The chosen level of theory predicts a secondary structure of crambin in excellent agreement with the experiment, without dramatic changes in geometry, regardless of the water content. In all cases, the backbone RMSD is <0.8 Å. However, the number of water molecules included in the simulation greatly affects this result, with the backbone RMSD moving from 0.781 Å in the 0W case to 0.432 and 0.381 Å in the 84W and 172W cases, respectively. Indeed, also Kulik *et al.* were able to improve their backbone RMSD by adding explicit (albeit classically modeled) water molecules in their DFT protein optimizations.^14^ Expectedly, the structural features of the different motifs in crambin affects how well the simulation predicts their geometry. As reported in Table 2, the RMSD of α-helices and β-sheets regions is lower than the whole protein. Moreover, these regions are already well conserved in the 0W model, since their structure is mostly determined by intra-molecular H-bond interactions. *Vice versa*, when water is not included, the description of the random coil region close to the C-terminus deviates from experiment, because its conformation likely depends on the interactions with the surrounding solvent.

**Table 2:** Root mean square deviations (RMSD)^*a*^

RMSD (Å)	0W//EXP	84W//EXP	172W//EXP
Backbone^a^	0.781	0.432	0.381
α-Helix A^b^	0.442	0.243	0.177
α-Helix B^b^	0.672	0.360	0.274
β-Sheet^b^	0.551	0.364	0.297
Random coil^b^	1.148	0.547	0.593

^*a*^Values in Å with respect to the experimental crambin crystal structure (EXP) after full B3LYP-D*/6-31G(d,p) optimization as computed for ^a^all backbone atoms, ^b^backbone atoms of defined regions (*cf.*Fig. 1c) in the cases: 0W: optimized with 0H_2_O per cell; 84W: optimized with 84H_2_O per cell; 172W: optimized with 172H_2_O per cell.

Comparison of the present RMSD values with data in literature shows good performance of the present approach. The HF/4-21G optimization of crambin (dry and free molecule) by van Alsenoy *et al.* resulted in a 0.4 Å backbone RMSD with respect to the experiment, albeit only a small number of optimization steps (79) was performed at the time.^15^ More recently, a fragment molecular orbital HF optimization of crambin (free molecule embedded in water solvent described by a polarizable continuum model) obtained a 0.525 Å backbone RMSD with respect to the experiment.^25^

As regard how the optimization is affected by the starting geometry, we fully optimized a 0W crambin crystal starting from a 0.54 Å X-ray structure (PDB: 1EJG) and compared the result with the one so far described. The backbone RMSD between the two B3LYP-D*/6-31G(d,p) optimized crambin structures was computed as 0.622 Å, while being 0.127 Å between the corresponding starting geometries. While this means that the two optimizations converged to different local minima, the two 0W geometries differed by just 71 kJ mol^–1^ per cell, dispersion included (10 kJ mol^–1^ per cell, without dispersion).

### Crystal formation energies

The outcome of an *ab initio* calculation allows to work out accurate and unbiased energetics for the studied systems. Here, we evaluated the BSSE-corrected formation energies for the three 0W, 84W and 172W crambin crystals, both with and without including the dispersion (vdW) contribution (*cf.* ESI for related formulae). To this aim, we considered different processes that bring the separate components to the final crystal structures, as reported in Table 3.

**Table 3:** Energetics of the crambin crystal formation

Process	*n*	Δ*E*^C^^*a*^	Δ*E*^CD^^*b*^	disp^*c*^
2P + *n*W → CRY(*n*W)	0	+442 [+0.3]	–1008 [–0.8]	–1450 [–1.1]
	84	–3400 [–2.2]	–6332 [–4.1]	–2932 [–1.9]
	172	–6971 [–3.9]	–11282 [–6.3]	–4311 [–2.4]
2P + W_*n*_(liq) → CRY(*n*W)	84	+2162	+79	–2082
	172	+2464	–107	–2571
CRY(0W) + *n*W → CRY(*n*W)	84	–3842 (–46)	–5324 (–63)	–1482 (–17)
	172	–7413 (–43)	–10274 (–60)	–2861 (–17)
CRY(0W) + W_*n*_(liq) → CRY(*n*W)	84	+1720 (+20)	+1087 (+13)	–632 (–7)
	172	+2022 (+12)	+901 (+5)	–1121 (–7)

^*a*^Reaction energies, using as reference the optimized crambin and water (when included) molecules in gas phase, obtained without dispersion (B3LYP//B3LYP-D*) and corrected for the Basis Set Superposition Error (BSSE).

^*b*^As ^*a*^, but obtained with dispersion (B3LYP-D*//B3LYP-D*). In parentheses, the contribution for each individual water molecule (when applicable) is reported.

^*c*^Dispersion contribution evaluated as: Δ*E*^CD^ – Δ*E*^C^. P: B3LYP-D*/6-31G(d,p) optimized crambin molecule. W: B3LYP-D*/6-31G(d,p) optimized water molecule. CRY: crambin crystal B3LYP-D*/6-31G(d,p) optimized in the three 0W, 84W, 172W cases. W_*n*_(liq): B3LYP-D*/6-31G(d,p) optimized liquid water box containing *n* water molecules. In square brackets, the atom contribution to some formation energies. All values are in kJ mol^–1^.

Considering the 0W case, its formation energy starting from two isolated B3LYP-D*/6-31G(d,p) optimized crambin molecules in gas phase is computed as exothermic only when dispersion is included (–1008 kJ mol^–1^) and amount to a mere –0.8 kJ mol^–1^ per atom contribution. The value obtained without dispersion (+442 kJ mol^–1^) suggests that the limited H-bond protein–protein interactions (two, between the symmetry related molecules in the unit cell) and the macroscopic dipole–dipole (*vide infra*) interactions cannot overcome the structure deformation occurring when going from gas to condensed phase. This unequivocally shows that a dry crambin crystal would be kept together only by vdW interactions. The structure deformation cost is reported in Table 4 as +487 kJ mol^–1^, including dispersion. This is an important outcome of the present approach in which it is shown that electrostatic, induction and exchange repulsion alone (rigorously computed within the B3LYP approach), despite the large crambin dipole (*vide infra*), cannot keep the crystal of crambin in place.

**Table 4:** Deformation energies of crambin (P) and water (W) when moving from gas (protein) and liquid (water) phase to the crambin crystal^*a*^

Model	P(mol) → P(CRY)^*b*^		W_*n*_(liq) → W_*n*_(CRY)^*c*^	
	Δ*E*	Δ*E*^D^	Δ*E*	Δ*E*^D^
0W	+485	+487	—	—
84W	+610	+682	+55	+62
172W	+723	+813	+32	+35

^*a*^The superscript ^D^ indicates that dispersion is included.

^*b*^Deformation energy of a crambin molecule when moving from the free gas phase optimized geometry to its crystal form, for the three 0W, 84W and 172W cases.

^*c*^Deformation energy per water molecule when moving from a liquid periodic box to the crambin crystal, for the 84W and 172W cases. All values are in kJ mol^–1^ either per protein or per water molecule.

We considered different processes resulting in the hydrated 84W and 172W structures. The formation of these crystals (Table 3) from all isolated components in gas phase (2P + *n*W → CRY(*n*W), with *n* = 84 or 172) is obviously strongly exothermic for both models and regardless of the inclusion of dispersion, since it is mostly driven by the formation of new water–water and protein–water interactions. Such interactions overcome the structure deformation cost for crambin when going from gas to condensed phase, evaluated as +682 kJ mol^–1^ and +813 kJ mol^–1^ for the 84W and 172W cases, respectively (Table 4). These numbers are larger than for the 0W case (+487 kJ mol^–1^), since crambin rearranges its structure in presence of water to maximize the interactions. Despite the relevant role of H-bond interactions in the hydrated crystals, dispersion still constitutes 49% and 37% of the formation energies in the 84W and 172W cases, respectively.

A more realistic reference system is the one starting from liquid water (2P + W_*n*_(liq) → CRY(*n*W), with *n* = 84 or 172), that is using an optimized liquid water box as a reference in spite of isolated water molecules (*cf.* computational details). As reported in Table 3, for the 84W case such process is always endothermic, since the newly formed protein–water interactions do not compensate for the loss of H-bonds between water molecules in the liquid. However, dispersion partially compensates for this effect as it reduced the repulsion of +2162 kJ mol^–1^ due to pure B3LYP to +79 kJ mol^–1^. Indeed, when dispersion is included, such process is slightly favored for the 172W case (–107 kJ mol^–1^), while it remains strongly endothermic for the dispersion free calculation (+2464 kJ mol^–1^). Table 4 reports the energy loss per water molecule when moving from the liquid to the crystal phases. This value is the balance between lost and formed interactions in the crystal formation process and resulted higher (+62 kJ mol^–1^) for the 84W case than for the 172W one (+35 kJ mol^–1^). This behavior is due to the higher similarity with liquid-like water for the 172W case compared with the 84W one.

By properly combining the above results, we computed the hydration energies of the dry 0W crystal, starting both from gas phase and liquid water, to generate the two 84W and 172W structures (CRY(0W) + *n*W → CRY(*n*W) and CRY(0W) + W_*n*_(liq) → CRY(*n*W), with *n* = 84 or 172). As reported in Table 3, such processes are *exothermic* when starting from isolated water molecules and *endothermic* when starting from liquid water, in agreement with the known hydrophobic, insoluble nature of crambin in water. When normalizing the reaction energies per water molecule for both the 84W and 172W cases, it turns out that each water molecule accounts for about –60 kJ mol^–1^ when referring to gas phase, with dispersion contributing by 28% of this energy. When referring to liquid water, the process leading to the 172W case is less endothermic than the 84W one, albeit still unfavored, due to the reduced energy cost paid by water in the process (*vide supra*).

These computed formation energies suggest that the formation of a protein crystal, and more specifically for a hydrophobic protein like crambin, is due to a balance between lost and formed interactions, with a significant role of vdW forces playing a comparable, if not greater, role, than H-bond interactions.

As regard how these results are dependent on the chosen level of theory, we investigated the role of the DFT functional, the basis set and the employed dispersion correction. We performed single point energy calculations on the 84W model using a different DFT hybrid functional (HSE06 54): no change in relative stabilities (Table S1 in ESI,† to be compared with Table 3) was seen, but an overbinding was observed with respect to the B3LYP functional. Considering the basis set effect, particularly regarding the BSSE, we evaluated that about 50% of the uncorrected B3LYP/6-31G(d,p) cohesive energy of the crambin crystal (in both the 84W and 172W cases) is BSSE-driven spurious attraction. Confirming that we are far from basis set convergence, reducing the basis size (from 6-31G(d,p) to 6-31G, removing polarization functions) significantly varied computed cohesive energy values (Table S2) with a further overbinding. Finally, some overestimation of dispersion interactions by the D* correction might be considered, since it is derived from the relatively old Grimme's D2 implementation.^28^,^51^ However, single point energy calculations with the more recent Grimme's D3 correction,^56^ inclusive of the three-body term, resulted in cohesive energies (Table S3 in ESI†) very close to those obtained with the D* approach, confirming the validity of the re-parameterization by Civalleri *et al.*

### Protein interactions

The computed crystal formation energies of Table 3, although informative, provide few clues on the specific interactions that occur inside the crambin crystal. To investigate on this point, we computed the energies of the most representative interactions in both the 84W and 172W models. These results are included in Table 5.

**Table 5:** Interactions in the crambin crystal

Interaction	Δ*E*^C^^*a*^	Δ*E*^CD^^*b*^	disp^*c*^
Crambin–crambin	–52	–102	–50
Crambin–water shell (84W)^*d*^	–18	–27	–9
Crambin–water shell (172W)^*d*^	–15	–23	–8
Tyr29–water_Tyr29_^*e*^	–21	–25	–4
Ile7–water_Ile7_^*e*^	–8	–16	–8
Nterm–Glu23–Arg17–water^*e*^	–54	–66	–12

^*a*^Interaction energies, using as reference the individual components, frozen at the geometry of the crystal, obtained without including dispersion (B3LYP//B3LYP-D*), corrected for the Basis Set Superposition Error (BSSE).

^*b*^Same as ^*a*^ but obtained including dispersion (B3LYP-D*//B3LYP-D*), corrected for the BSSE.

^*c*^Dispersion contribution evaluated as: Δ*E*^CD^ – Δ*E*^C^.

^*d*^The water shell is here defined as all the complete water molecules within 4 Å of any protein atom in the two 84W and 172W cases (*cf.*Fig. 4).

^*e*^These cases refer to Fig. 5b–d; in each interaction only the water molecules interacting with the given residues were included. All values are in kJ mol^–1^ either per protein or water molecule.

**Fig. 4:**
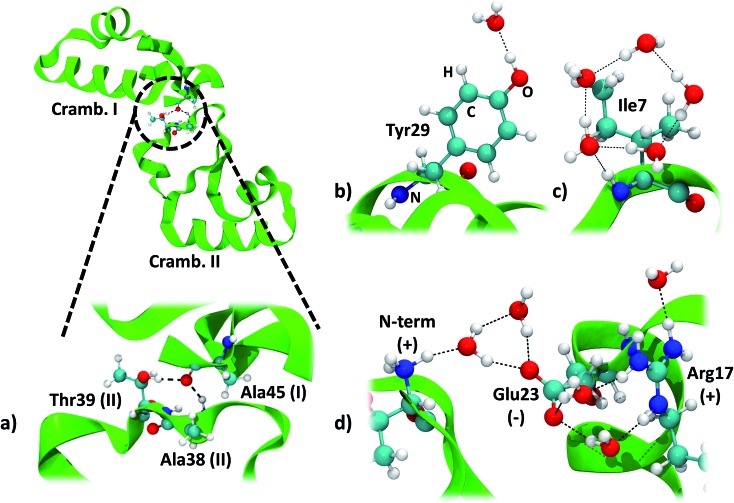
Main interactions in the crambin crystal. All views are from the 84W case (*cf.*Fig. 2). (a) Crambin–crambin interactions in the unit cell: overall view of the interaction and magnification of the involved residues. (b) Water interacting with polar residues (Tyr29). (c) Water arrangement around apolar residues (Ile7). (d) Water arrangement around charged residues (cluster made by the N-terminus, Glu23 and Arg17). Color code: red, oxygen; white, hydrogen; blue, nitrogen; cyan, carbon; green, 84W protein backbone; black dashed lines, H-bonds.

The two symmetry related crambin molecules in the unit cell interact through a combination of vdW and H-bond interactions. Fig. 4a shows these contacts for the 84W case: two H-bonds are formed between Ala45 (backbone's CO) of one molecule and Ala38 (backbone's NH) and Thr39 (residue's OH) of the other. The crambin–crambin interaction energy resulted in –102 kJ mol^–1^ per protein molecule when dispersion is included, reducing to –52 kJ mol^–1^ when only H-bond interactions are considered (Table 5), confirming that half of the crambin–crambin interaction is due to the dispersion contribution. It is worth noting that these values differ from those reported in Table 3 for the 0W crystal formation, since in the crystal each protein interacts with all nearby replicas.

An important and much debated topic is the strength and biological role of water–protein interactions.^32^ Here we take advantage of the *ab initio* level of theory of our simulations, to contribute at that issue by providing energetics for the crambin–water interaction. Water molecules around a protein are usually classified as internal and strongly bound water, surface hydration water and bulk water.^64^ No internal water is found in the crambin crystal, while both bulk (in the solvent channel) and surface water have been identified.^34^

**Fig. 5:**
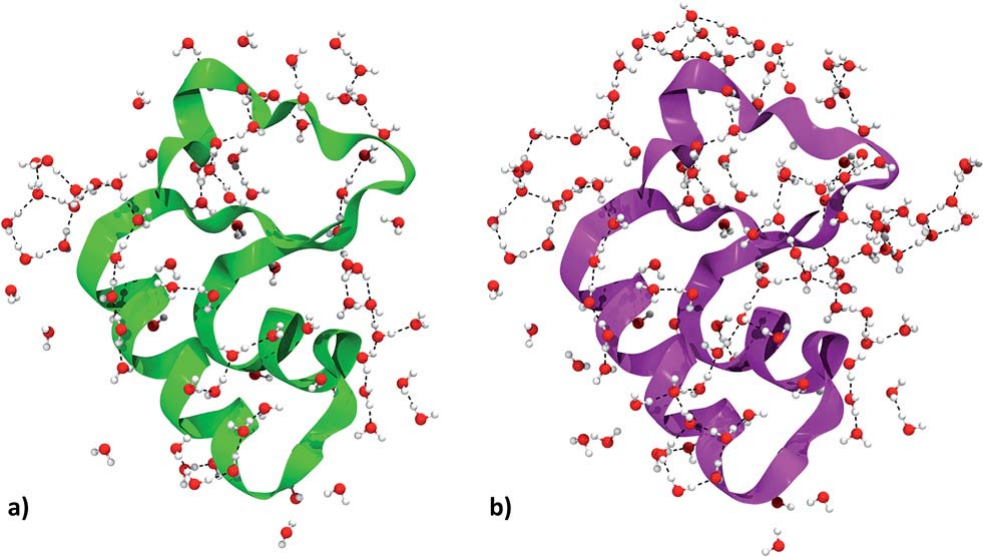
Crambin solvation shell, here defined as all the complete water molecules within 4 Å of any protein atom. (a) Solvation shell of the 84W case (*cf.*Fig. 2). (b) Solvation shell of the 172W case (*cf.*Fig. 2). Color code: red, oxygen; white, hydrogen; green, 84W protein backbone; magenta, 172W protein backbone; black, water–water H-bonds.

We focused on water molecules interacting with the crambin surface, by identifying the protein solvation shells in both the 84W and 172W optimized crystals. We included in each shell all complete water molecules within 4 Å of any protein atom. These shells are shown in Fig. 5 and are constituted by 83 and 126H_2_O molecules for the 84W and 172W cases, respectively. A total of 71 H-bond interactions are formed between water and crambin in the 84W crystal (0.86 H-bonds per H_2_O), while this value rises to 86 H-bonds in the 172W model (0.68 H-bonds per H_2_O). These numbers suggest that the most directly interacting water molecules were already included in the original pool of the 84 well-ordered water molecules, as determined by neutron diffraction. The relatively low number of H-bond interactions is due to the abundance of apolar residues in crambin. We computed the BSSE-corrected average interaction energy per water molecule for the two models (Table 5), resulting in –27 kJ mol^–1^ and -23 kJ mol^–1^ for the 84W and 172W crystal, respectively, dispersion included. A comparison with the dispersion-free values of Table 5 confirms that, expectedly, vdW plays a limited role in water–protein interactions. We also used the computed Mulliken net charges of the systems to evaluate the charge transfer associated with this interaction, corrected for the BSSE according to the procedure described in ESI.† A net flux of charge from the protein to the water shell is observed amounting to 0.1381*e* (0.0016*e* per H_2_O) and 0.1871*e* (0.0015*e* per H_2_O) for the two 84W and 172W cases, respectively. It is worth stressing that these quantities are not accessible from calculations based on the classical force-fields for proteins. Nadig *et al.* observed the same direction of charge transfer (from protein to solvent) in their semi-empirical simulation on *E. coli* cold-shock protein A, a small hydrophobic protein like crambin.^65^ On the other hand, recent *ab initio* molecular dynamics simulations on a more hydrophilic protein, the bovine pancreatic trypsin inhibitor, computed a charge transfer from water to the protein surface, suggesting that flux direction varies widely with protein composition.^18^

The details of the water–crambin interaction have been revealed by a number of experimental research papers,^34^,^36^,^59^ thanks to the availability of high resolutions structures. Water forms a number of H-bonds with crambin, particularly with backbone COs and NHs. It also stabilizes charged residues at the surface.^34^ Furthermore, the apolar character of crambin is known to cause structuration of hydration water, that forms clathrate-like pentagonal rings around hydrophobic residues.^34^ All these interactions are present in our models. Interestingly, some randomly added water molecules in the 172W case moved during optimization to form new pentagonal rings around hydrophobic patches of the protein, confirming that the hydrophobic nature of crambin favors this kind of water organization.

We computed the energy of some specific water–crambin interactions, following Finney's classification of water–protein interactions concerning metals, polar residues and charged residues.^66^ Since no metal is present in our case, we selected, from the 84W model, one water interacting with a polar residue (the OH group of Tyr29, Fig. 4b). We selected also a group of five water molecules interacting with a cluster of charged residues (made up by the positively charged N-terminus, the deprotonated carboxyl of Glu23 and the positively charged guanidinium group of Arg17, Fig. 4d). Furthermore, we decided to investigate one water pentagonal ring, specifically the one around Ile7 (Fig. 4c). Results are reported in Table 5. Interaction energy per water molecule with Tyr29 is evaluated as –25 kJ mol^–1^ (16% dispersion), while is –16 kJ mol^–1^ (50% dispersion) around Ile7. Finally, the average interaction energy per water molecule around the charged residues is –66 kJ mol^–1^ (18% dispersion). The average water–protein charge transfer is estimated, for these three cases, as 0.0235 (H_2_O–Tyr29), 0.0080 (H_2_O–Glu23/Arg17) and 0.0170*e* (Ile7), always in favor of the protein. The order of stability of the interactions is, as expected, charged > polar > apolar. However, due to the vdW contribution, also apolar groups are bound quite strongly to the hydration water.

### Other properties

**Fig. 6:**
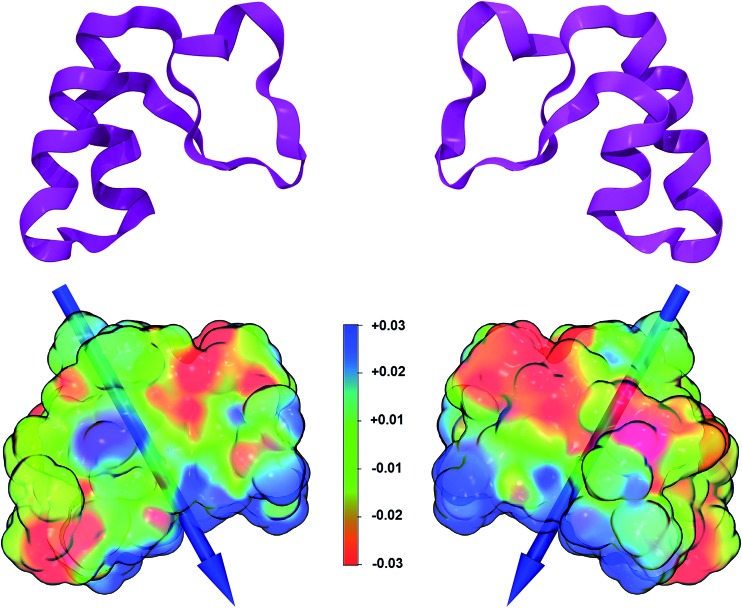
Electrostatic potential and dipole moment of crambin, as extracted from the 172W crystal structure. Left and right pictures are flipped by 180°. Top: Ribbon view of crambin secondary structure. Bottom: Electrostatic potential mapped on the B3LYP-D*/6-31G(d,p) electron density; an isosurface (isovalue = 0.0001*e*) is shown. Blue, green and red colors correspond to positive, neutral, and negative values of the electrostatic potential (range values: MIN –0.03 au; MAX +0.03 au.). The computed dipole moment is represented as a blue vector whose length is proportional to its module (34 Debyes). The center of mass of the protein is located at the middle point of the dipole vector.

A large number of properties can be extracted from the analysis of the wavefunction obtained at the end of an *ab initio* simulation. Here we computed the dipole moment and the electrostatic potential for the B3LYP/6-31G(d,p) crambin molecule, as extracted from the 172W optimized crystal (Fig. 6).

The electrostatic potential, mapped on the B3LYP-D*/6-31G(d,p) electron charge density, was computed using a relatively dense 200 × 200 × 200 grid: it shows (Fig. 6) well defined positive and negative patches on the protein surface, although, as expected for a highly hydrophobic protein, large portions are apolar. The *versus* of the dipole moment is oriented toward a large positive region of the electrostatic potential and its module amounts to 34 Debye. To see how the *ab initio* value compares to what obtained through other methodologies, the same crambin geometry was analyzed through both the semiempirical MOPAC code,^46^ with a single point PM7 energy calculation, and a classical approach.^67^ The module of the crambin dipole were 40 and 49 Debye, at PM7 and with classical method, respectively, suggesting that less accurate methods tend to overestimate the dipole magnitude. On the other hand, all three methods predicted a very similar dipole orientation.

## Conclusions

The present work has demonstrated the applicability of accurate quantum mechanical methods, based for the first time on all-electron DFT with hybrid functionals, to the simulation of proteins and of protein crystals in particular. Moreover, in contrast with previous results in literature,^14^,^19^ no convergence issue was reported, with the default calculation parameters of the CRYSTAL14 code. Geometry of the crambin protein, chosen as a convenient test case, was well predicted at the chosen B3LYP-D*/6-31G(d,p) level of theory, with values comparable to highly parameterized force fields. However, explicit insertion of crystallographic water in the model has proven essential to accurately reproduce both protein crystal parameters and protein secondary structure. The chosen approach provided accurate unbiased energetics for the crambin crystal formation, elucidating the role of the different contributions (H-bond *vs.* vdW interactions, geometry modifications, water reorganization) to the process. Furthermore, the water–crambin interactions in the crystal were investigated, both geometrically and energetically, by elucidating the strength and structure of different kind of solvent–protein contacts. Particularly, the formation of stable clathrate-like structures around hydrophobic patches of the protein surface was confirmed as energetically favored by our simulations. Finally, some important properties, such as the electrostatic potential and the dipole moment, were computed through a full *ab initio* approach. As a peculiar feature of the *ab initio* approach, we computed the electronic charge transfer resulting in a flux of 0.0015*e* per H_2_O from the crambin molecule towards the lattice water molecules. We also evaluated the same quantity for selected subset of water clusters solvating key point of the crambin in the crystal. Although these results are limited to interactions in the solid state, it is reasonable to suppose that the kind of interactions here described for the crystalline phase are also present in the liquid phase.

More generally, this work confirmed that, thanks to modern high performance computing architectures and the improvements in quantum-chemistry computational codes, *ab initio* simulations of the crystal of small proteins, including both energy calculations and geometry optimizations, are achievable in a reasonable amount of time. Crambin's 46 aminoacids, however, classify it as a relatively small protein, compared to the median length of eukaryotic proteins, that has been estimated as 361 aminoacids.^68^ To demonstrate the feasibility of the chosen approach also to proteins of average size, we performed a limited number of optimizations steps (∼10) on the molecular structure of γ-chymotrypsin (PDB: ; 8GCH),^57^ composed by 4 chains, 244 aminoacids, 349H_2_O molecules for a total of 4575 atoms. We did not encounter convergence issues also in this case and an optimization step took on average about 3 hours on 1200 Cray XC40 Cores. Such performance confirms the feasibility of the method to the simulation of average-sized proteins, while probably ruling out its routine application. However, it must be noted that 1000 optimizations steps on the γ-chymotrypsin model on 1200 cores would cost about 3.6 million CPU hours, below the average allocation of present day high performance computing project calls.

As a final remark, we are well aware that the present approach is a static one, *i.e.* the considered systems are without temperature effects. While protein mobility as a single molecule in water solvent is a key factor for their biological functionality, the atomic motion is far more restricted in the crystal. Indeed, diffraction experimental data provides atomic thermal factor as a measure of the libration of each atom around its equilibrium position through the anisotropic displacement parameters (ADP) which are now computable with the CRYSTAL14 code. We therefore believe that the full calculation of the vibrational spectrum of the crambin crystal through which the ADP are computed will provide direct information on the atomic motion amplitude to be compared with the experimentally determined ones. Work is in progress in our laboratory to carry out such calculation.

## Supplementary Material

Supplementary information
