# Supplementary material for: Elucidating the fundamental forces in protein crystal formation: the case of crambin
Source: Chem Sci. 2015 Nov 24;7(2):1496–507. doi: 10.1039/c5sc03447g (PMC5963673; doi:10.1039/c5sc03447g)
Supplement: Supplementary file 1 [file SC-007-C5SC03447G-s001.pdf]

Electronic Supplementary Material (ESI) for Chemical Science.
This journal is © The Royal Society of Chemistry 2015

Quantum-mechanical simulation of protein crystals and water-protein interactions: the case of crambin

Massimo Delle Piane, Marta Corno, Roberto Orlando, Roberto Dovesi, Piero Ugliengo†

Department of Chemistry and NIS Centre, University of Torino, via Pietro Giuria 7, 10125, Torino, Italy

SUPPORTING INFORMATION

COMPUTATIONAL DETAILS

The development version of the CRYSTAL14 code[1] was adopted for all the *ab initio* calculations in its massively parallel implementation.[2] CRYSTAL14 permits the study of systems of all dimensionalities, from molecules to crystals. It employs a Gaussian type basis, paired with both Density Functional Theory (DFT) and Hartree Fock (HF) Hamiltonians.

Hamiltonian

All calculations were performed within the Density Functional Theory, adopting the Becke, 3 parameters, Lee-Yang-Parr (B3LYP) hybrid functional.[3] Due to the use of a local basis set and suitable truncation of the exchange series, the present implementation of hybrid functionals in CRYSTAL14 is very efficient compared to other codes based on plane waves, being only about three times slower than pure GGAs.[2] The electron density and its gradient were integrated over a pruned grid consisting of 974 angular and 75 radial points per atom generated through the Gauss–Legendre quadrature and Lebedev schemes. The accuracy of this grid can be estimated from the integrated

number of electrons, that is 6760.0006 *e* for the largest studied system (6760 in the unit cell). Values of the tolerances that control the Coulomb and exchange series in periodical systems were kept at the default values (10^{-6} and 10^{-12} Ha).[4] For the periodic cases, the Hamiltonian matrix was diagonalized only at the Γ point, due to the large volume of the unit cells.[5] The eigenvalue level-shifting technique was used to initially lock the system in a non-conducting state, with the level shifter set to 0.6 Ha, which is then relaxed at the end of the self consistent field iterations.[6] To further help convergence of the Self Consistent Field (SCF), the Fock/KS matrix at a given cycle was mixed with 30% of the one of the previous cycle. The dispersion contribution was added to the DFT energies and gradients, by means of the empirical dispersion correction originally proposed by Grimme and known as D2 correction,[7] with the modified parameters proposed by Civalleri *et al.* for the treatment of molecular crystals using the B3LYP hybrid functional (hereafter referred as B3LYP-D*).[8]

Basis Set

A split valence double-ζ basis set (6-31G(d,p)) plus polarization functions was applied to C, N, S and O,[9] while an Ahlrichs's pVDZ basis was used for H.[10] For the sake of simplicity, the chosen combination of Hamiltonian and basis will be identified as B3LYP-D*/6-31G(d,p) in the following. As anticipated in the introduction, we modeled three crambin crystals differing for the amount of solvating water, namely with 0W, 84W and 172W water molecules investigated in the present work (*vide infra*) resulting in 12354, 14370 and 16482 Atomic Orbitals (AOs) in the unit cell, respectively. Both cell parameters and internal coordinates were optimized using the analytical gradient method.

Geometry optimizations

The Hessian is upgraded with the Broyden-Fletcher-Goldfarb-Shanno (BFGS) algorithm.[11] The initial Hessian for most calculations was generated by means of a classical model as proposed by Schlegel.[12] Due to memory constraints, for the largest studied system (172W, 1800 atoms/cell), the initial Hessian guess was changed to the identity matrix. Default convergence values were used for the maximum allowed gradient, the maximum atomic displacement and their maximum allowed root

mean square values for convergence (0.00045 Ha·bohr^{-1}, 0.00180 Bohr, 0.00030 Ha·bohr^{-1} and 0.00120 Bohr, respectively).

Computation of crystal formation energies

The energetics of the protein crystal and of the protein-protein and protein-water interactions was decomposed in various contributions as in Ref. [13] and detailed below.

For a general crystal CRY with n proteins P and m water molecules W in the unit cell, the formation energy ΔE can be defined as:

$$\Delta E = E(CRY/CRY) - n \cdot E(P/P) - m \cdot E(W/W) \quad (1)$$

where $E(CRY/CRY)$, $E(P/P)$ and $E(W/W)$ are the energies of the optimized crystal, free protein and free water, respectively (the symbol after the / refers to the geometry at which the energy was evaluated). ΔE is determined by the balance between the energy gain due to the new interactions present in the crystal with respect to the isolated compounds and the energy loss due to the structural deformations occurring for P and W from the gas to the condensed phase. For a crystal to be thermochemically stable $\Delta E < 0$. A deformation-free formation energy ΔE^* is defined as:

$$\Delta E^* = E(CRY/CRY) - \sum_{i=1}^{n} E(P_i/CRY) - \sum_{i=1}^{m} E(W_i/CRY) \quad (2)$$

where $E(P_i/CRY)$ is the energy of the i-th isolated protein taken at the geometry of the optimized crystal, and $E(W_i/CRY)$ is the energy of the i-th water molecule taken in the geometry of the optimized crystal. Eq. 2 can be easily reformulated to include the Basis Set Superposition Error (BSSE) correction, using the same counterpoise method adopted for intermolecular complexes:[14]

$$\Delta E^{*C} = E(CRY/CRY) - \sum_{i=1}^{n} E(P_i + gh/CRY) - \sum_{i=1}^{m} E(W_i + gh/CRY) \quad (3)$$

where "$+\ gh$" means that the energy is computed with the addition of the ghost functions of the surrounding molecules. In periodic systems, this is usually accomplished by including ghost functions within a chosen cutoff distance (here, 40 Å) around the molecule. The $BSSE$ can then be defined as:

$$BSSE = \Delta E^{*} - \Delta E^{*C} \tag{4}$$

and the final, BSSE corrected, formation energy ΔE^{C} is defined as:

$$\Delta E^{C} = \Delta E - BSSE \tag{5}$$

Eqs. 1-5 were then adapted to the different systems considered in the present work. For protein-protein and protein-water interactions of Table 4, the energetics was studied deformation-free, that is with the structure of the components frozen at the geometry of the interacting system. For some processes of Table 3, a water box representing liquid water was taken as a reference. To study such processes, Eqs. 1-3 must be reformulated as follows:

$$\Delta E = E(CRY/CRY) - n \cdot E(P/P) - E(W_m/W_m) \tag{6}$$

$$\Delta E^{*} = E(CRY/CRY) - \sum_{i=1}^{n} E(P_i/CRY) - E(W_m/CRY) \tag{7}$$

$$\Delta E^{*C} = E(CRY/CRY) - \sum_{i=1}^{n} E(P_i + gh/CRY) - E(W_m + gh/CRY) \tag{8}$$

where $E(W_m/W_m)$ is the energy of an optimized liquid water box containing m molecules, while $E(W_m + gh/CRY)$ and $E(W_m/CRY)$ are the energies of the 3D framework of m water molecules as found in the crystal with and without the protein ghost functions, respectively. Eqs. 4-5 remain valid. To evaluate $E(W_m/W_m)$, a water box of 84 molecules (side: 13.4 Å) was generated through the PACKMOL utility,[15] thermalized through a 10 ps by PBE-D2/TZVP *ab initio* molecular dynamics simulation at 300 K with the CP2K[16] software and then B3LYP-D*/6-31G(d,p) fully optimized with CRYSTAL14. For the 172W case $E(W_{84}/W_{84})$ was scaled by a factor 172/84.

Computation of BSSE-corrected charge transfers

Consider the interaction between two components, A and B, so that:

$$A + B \rightarrow AB \tag{1}$$

These two systems have, when isolated, total electronic charges Q_A and Q_B, corresponding to their total number of electrons. These electrons are distributed among the different atoms composing the systems:

$$Q_A = \sum_{i=1}^{N_A} Q_i \tag{2}$$

where N_A is the total number of atoms in A and Q_i is the electronic charge on the ith atom, that may be estimated through a Mulliken population analysis.

The interaction complex AB has a total electronic charge Q_{AB}:

$$Q_{AB} = Q_{A*} + Q_{B*} \tag{3}$$

where Q_{A*} and Q_{B*} are the electronic charges on A and B when involved in the interaction. Of course, due to the charge transfer associated to this interaction, $Q_{A*} \neq Q_A$ and $Q_{B*} \neq Q_B$.

This charge transfer (QT) can then be defined as:

$$QT_{B \rightarrow A} = Q_{A*} - Q_A \tag{4}$$

If $QT_{B \rightarrow A} > 0$, a net flux of electronic charge from B to A has occurred, while B has obtained charge if $QT_{B \rightarrow A} < 0$. Of course, since the total electronic charge must be conserved:

$$QT_{B \rightarrow A} = -QT_{A \rightarrow B} \tag{5}$$

The Basis Set Superposition Error (BSSE) is known to affect any calculation based on atom-centered basis functions. This error is due to the superposition, in the AB complex, of the functions centered on both A and B atoms, so that AB is described by a richer basis set than the A and B components alone. This discrepancy causes inconsistencies when comparing total energies, such as when computing interaction energies. The same problem is present when dealing with equation (4), since

Q_{A*} and Q_A come from calculations with a different number of basis functions, *i.e.* Q_A misses functions centered on B. When these functions are present (when computing Q_{A*}), the effect of the BSSE is a net flux of electronic charge in order to partially occupy them.

This un-physical BSSE-caused charge transfer (QT^{BSSE}) can be evaluated by doing a calculation on A and B when the ghost functions of the other component are present:

$$QT_A^{BSSE} = Q_{A[B]} - Q_A$$
$$QT_B^{BSSE} = Q_{[A]B} - Q_B \quad (6)$$

where the square brackets mean that only the ghost functions of the component are included. QT^{BSSE} is always a negative quantity.

The BSSE-corrected charge transfer due to the interaction (QT^C) is then computed by removing the spurious charge fluxes of equations (6) from the uncorrected charge transfer of equation (4):

$$QT_{B\to A}^{C} = QT_{B\to A} - QT_A^{BSSE} + QT_B^{BSSE} \quad (7)$$

where signs are conveniently put to account for charge fluxes directions.

Bibliography

(1) Dovesi, R.; Orlando, R.; Erba, A.; Zicovich-Wilson, C. M.; Civalleri, B.; Casassa, S.; Maschio, L.; Ferrabone, M.; De La Pierre, M.; D'Arco, P.; Noël, Y.; Causà, M.; Rérat, M.; Kirtman, B. *Int. J. Quantum Chem.* **2014**, *114* (19), 1287.

(2) Orlando, R.; Delle Piane, M.; Bush, I. J.; Ugliengo, P.; Ferrabone, M.; Dovesi, R. *J. Comput. Chem.* **2012**, *33* (28), 2276.

(3) Becke, A. D. *J. Chem. Phys.* **1993**, *98* (7), 5648.

(4) Dovesi, R.; Saunders, V. R.; Roetti, C.; Orlando, R.; Zicovich-Wilson, C. M.; Pascale, F.; Civalleri, B.; Doll, K.; Harrison, N. M.; Bush, I. J.; D'Arco, P.; Llunell, M.; Causà, M.; Noël, Y. *CRYSTAL14, User's Manual*; Università di Torino: Torino, Italy, 2014.

(5) Monkhorst, H. J.; Pack, J. D. *Phys. Rev. B.* **1976**, *8*, 5188.

(6) Saunders, V. R.; Hillier, I. H. *Int. J. Quantum Chem.* **1973**, *7* (4), 699.

(7) Grimme, S. *J. Comput. Chem.* **2006**, *27* (15), 1787.

(8) Civalleri, B.; Zicovich-Wilson, C. M.; Valenzano, L.; Ugliengo, P. *CrystEngComm* **2008**, *10* (4), 405.

(9) Francl, M. M.; Pietro, W. J.; Hehre, W. J.; Binkley, J. S.; Gordon, M. S.; DeFrees, D. J.;

Pople, J. A. *J. Chem. Phys.* **1982**, *77* (7).

(10) Schäfer, A.; Horn, H.; Ahlrichs, R. *J. Chem. Phys.* **1992**, *97*, 2571.

(11) Broyden, C. G. *IMA J. Appl. Math.* **1970**, *6* (1), 76.

(12) Bernhard Schlegel, H. *Theor. Chim. Acta* **1984**, *66* (5), 333.

(13) Delle Piane, M.; Corno, M.; Ugliengo, P. *J. Chem. Theory Comput.* **2013**, *9* (5), 2404.

(14) Boys, S. F.; Bernardi, F. *Mol. Phys.* **1970**, *19* (4), 553.

(15) Martínez, L.; Andrade, R.; Birgin, E. G.; Martínez, J. M. *J. Comput. Chem.* **2009**, *30* (13), 2157.

(16) Hutter, J.; Iannuzzi, M.; Schiffmann, F.; Vandevondele, J. *Wiley Interdiscip. Rev. Comput. Mol. Sci.* **2014**, *4* (1), 15.

Supporting tables

Table S1. Energetics of the crambin crystal formation (HSE06-D*/6-31G(d,p)//B3LYP-D*/6-31G(d,p)).

Process	*n*	$\Delta E^{C\ a}$	$\Delta E^{CD\ b}$	disp [c]
2P + *n*W → CRY(*n*W)	0	+100 [+0.1]	-1350 [-1.1]	-1450 [-1.1]
	84	-4448 [-2.9]	-7380 [-4.8]	-2932 [-1.9]
CRY(0W) + *n*W → CRY(*n*W)	84	-4548 (-54)	-6029 (-72)	-1482 (-17)

[a] Reaction energies as in Table 3, without including dispersion, but at the HSE06/6-31G(d,p)//B3LYP-D*/6-31G(d,p) level of theory (in the *x//y* notation *x* is the level at which the energy is computed, *y* is the level at which the geometry has been optimized). [b] As [a], but obtained with dispersion (HSE06-D*/6-31G(d,p)//B3LYP-D*/6-31G(d,p)). In square brackets, the atom contribution to some formation energies. In parentheses, the contribution for each individual water molecule (when applicable) is reported. [c] Dispersion contribution evaluated as: $\Delta E^{CD} - \Delta E^{C}$. P: crambin molecule in gas phase. W: water molecule in gas phase. CRY: crambin crystal in the two 0W, 84W cases. All values are in kJ·mol^{-1}.

Table S2. Energetics of the crambin crystal formation (B3LYP-D*/6-31G//B3LYP-D*/6-31G(d,p)).

Process	*n*	$\Delta E^{C\ a}$	$\Delta E^{CD\ b}$	disp [c]
2P + *n*W → CRY(*n*W)	0	+353 [+0.3]	-1097 [-0.9]	-1450 [-1.1]
	84	-5416 [-3.5]	-8348 [-5.4]	-2932 [-1.9]
CRY(0W) + *n*W → CRY(*n*W)	84	-5769 (-69)	-7251 (-86)	-1482 (-17)

[a] Reaction energies as in Table 3, without including dispersion, but at the B3LYP/6-31G//B3LYP-D*/6-31G(d,p) level of theory (in the *x//y* notation *x* is the level at which the energy is computed, *y* is the level at which the geometry has been optimized). [b] As [a], but obtained with dispersion (B3LYP-D*/6-31G//B3LYP-D*/6-31G(d,p)). In square brackets, the atom contribution to some formation energies. In parentheses, the contribution for each individual water molecule (when applicable) is reported. [c] Dispersion contribution evaluated as: $\Delta E^{CD} - \Delta E^{C}$. P: crambin molecule in gas phase. W: water molecule in gas phase. CRY: crambin crystal in the two 0W, 84W cases. All values are in kJ·mol^{-}1.

Table S3. Energetics of the crambin crystal formation (B3LYP-D3/6-31G(d,p)//B3LYP-D*/6-31G(d,p)).

Process	*n*	$\Delta E^{C\ a}$	$\Delta E^{CD\ b}$	disp [c]
2P + *n*W → CRY(*n*W)	0	+442 [+0.3]	-1031 [-0.8]	-1473 [-1.1]
	84	-3400 [-2.2]	-6593 [-4.3]	-3193 [-2.1]
CRY(0W) + *n*W → CRY(*n*W)	84	-3842 (-46)	-5562 (-66)	-1719 (-20)

[a] Reaction energies as in Table 3, without including dispersion, but at the B3LYP/6-31G//B3LYP-D*/6-31G(d,p) level of theory (in the *x//y* notation *x* is the level at which the energy is computed, *y* is the level at which the geometry has been optimized). [b] As [a], but obtained with dispersion (B3LYP-D3/6-31G//B3LYP-D*/6-31G(d,p)). In square brackets, the atom contribution to some formation energies. In parentheses, the

contribution for each individual water molecule (when applicable) is reported. [c] Dispersion contribution evaluated as: $\Delta E^{CD} - \Delta E^{C}$. P: crambin molecule in gas phase. W: water molecule in gas phase. CRY: crambin crystal in the two 0W, 84W cases. All values are in kJ·mol^{-1}.

OPTIMIZED GEOMETRIES (PDB files)

1) CRAMBIN – optimized gas phase molecule

```
COMPND    CRAMBIN OPTIMIZED GAS PHASE GEOMETRY
AUTHOR    DELLE PIANE, CORNO, ORLANDO, DOVESI, UGLIENGO
ATOM      1  N   THR A   1      -2.493  -4.567   8.109  1.00  0.00           N
ATOM      2  CA  THR A   1      -2.060  -3.222   7.634  1.00  0.00           C
ATOM      3  C   THR A   1      -1.713  -3.328   6.152  1.00  0.00           C
ATOM      4  O   THR A   1      -1.368  -4.430   5.706  1.00  0.00           O
ATOM      5  CB  THR A   1      -0.845  -2.831   8.536  1.00  0.00           C
ATOM      6  OG1 THR A   1      -1.235  -3.236   9.845  1.00  0.00           O
ATOM      7  CG2 THR A   1      -0.504  -1.352   8.479  1.00  0.00           C
ATOM      8  H1  THR A   1      -1.902  -5.349   7.727  1.00  0.00           H
ATOM      9  H2  THR A   1      -3.471  -4.785   7.905  1.00  0.00           H
ATOM     10  H3  THR A   1      -2.314  -4.520   9.139  1.00  0.00           H
ATOM     11  HA  THR A   1      -2.888  -2.520   7.818  1.00  0.00           H
ATOM     12  HB  THR A   1       0.022  -3.430   8.203  1.00  0.00           H
ATOM     13  HG1 THR A   1      -0.454  -3.685  10.265  1.00  0.00           H
ATOM     14 HG21 THR A   1       0.313  -1.153   9.186  1.00  0.00           H
ATOM     15 HG22 THR A   1      -1.369  -0.741   8.785  1.00  0.00           H
ATOM     16 HG23 THR A   1      -0.170  -1.043   7.478  1.00  0.00           H
ATOM     17  N   THR A   2      -1.793  -2.206   5.427  1.00  0.00           N
ATOM     18  CA  THR A   2      -1.137  -2.097   4.124  1.00  0.00           C
ATOM     19  C   THR A   2       0.122  -1.250   4.368  1.00  0.00           C
ATOM     20  O   THR A   2       0.075  -0.294   5.161  1.00  0.00           O
ATOM     21  CB  THR A   2      -2.063  -1.552   3.023  1.00  0.00           C
ATOM     22  OG1 THR A   2      -2.368  -0.190   3.328  1.00  0.00           O
ATOM     23  CG2 THR A   2      -3.331  -2.392   2.887  1.00  0.00           C
ATOM     24  H   THR A   2      -1.960  -1.315   5.892  1.00  0.00           H
ATOM     25  HA  THR A   2      -0.839  -3.106   3.821  1.00  0.00           H
ATOM     26  HB  THR A   2      -1.482  -1.607   2.083  1.00  0.00           H
ATOM     27  HG1 THR A   2      -2.944   0.139   2.612  1.00  0.00           H
ATOM     28 HG21 THR A   2      -3.077  -3.443   2.686  1.00  0.00           H
ATOM     29 HG22 THR A   2      -3.976  -2.032   2.073  1.00  0.00           H
ATOM     30 HG23 THR A   2      -3.919  -2.349   3.817  1.00  0.00           H
ATOM     31  N   CYS A   3       1.213  -1.660   3.715  1.00  0.00           N
ATOM     32  CA  CYS A   3       2.550  -1.144   3.967  1.00  0.00           C
ATOM     33  C   CYS A   3       3.195  -0.865   2.610  1.00  0.00           C
ATOM     34  O   CYS A   3       3.005  -1.676   1.688  1.00  0.00           O
ATOM     35  CB  CYS A   3       3.420  -2.169   4.709  1.00  0.00           C
ATOM     36  SG  CYS A   3       2.800  -2.673   6.361  1.00  0.00           S
ATOM     37  H   CYS A   3       1.135  -2.464   3.081  1.00  0.00           H
ATOM     38  HA  CYS A   3       2.476  -0.232   4.567  1.00  0.00           H
ATOM     39  HB1 CYS A   3       3.478  -3.108   4.140  1.00  0.00           H
ATOM     40  HB2 CYS A   3       4.429  -1.750   4.826  1.00  0.00           H
ATOM     41  N   CYS A   4       3.971   0.214   2.486  1.00  0.00           N
ATOM     42  CA  CYS A   4       4.538   0.541   1.190  1.00  0.00           C
ATOM     43  C   CYS A   4       6.042   0.804   1.323  1.00  0.00           C
ATOM     44  O   CYS A   4       6.498   1.386   2.314  1.00  0.00           O
ATOM     45  CB  CYS A   4       3.831   1.760   0.576  1.00  0.00           C
ATOM     46  SG  CYS A   4       2.031   1.514   0.294  1.00  0.00           S
ATOM     47  H   CYS A   4       4.102   0.892   3.252  1.00  0.00           H
ATOM     48  HA  CYS A   4       4.366  -0.328   0.549  1.00  0.00           H
ATOM     49  HB1 CYS A   4       3.888   2.622   1.256  1.00  0.00           H
ATOM     50  HB2 CYS A   4       4.289   2.055  -0.378  1.00  0.00           H
ATOM     51  N   PRO A   5       6.817   0.438   0.277  1.00  0.00           N
```

```
ATOM     52  CA  PRO A   5       8.281   0.590   0.341  1.00  0.00           C
ATOM     53  C   PRO A   5       8.756   1.967  -0.134  1.00  0.00           C
ATOM     54  O   PRO A   5       9.945   2.246  -0.209  1.00  0.00           O
ATOM     55  CB  PRO A   5       8.778  -0.561  -0.543  1.00  0.00           C
ATOM     56  CG  PRO A   5       7.709  -0.659  -1.641  1.00  0.00           C
ATOM     57  CD  PRO A   5       6.392  -0.309  -0.930  1.00  0.00           C
ATOM     58  HA  PRO A   5       8.647   0.480   1.372  1.00  0.00           H
ATOM     59  HB1 PRO A   5       9.789  -0.365  -0.920  1.00  0.00           H
ATOM     60  HB2 PRO A   5       8.812  -1.476   0.066  1.00  0.00           H
ATOM     61  HG1 PRO A   5       7.912   0.067  -2.447  1.00  0.00           H
ATOM     62  HG2 PRO A   5       7.653  -1.657  -2.096  1.00  0.00           H
ATOM     63  HD1 PRO A   5       5.719   0.296  -1.556  1.00  0.00           H
ATOM     64  HD2 PRO A   5       5.863  -1.220  -0.635  1.00  0.00           H
ATOM     65  N   SER A   6       7.776   2.869  -0.478  1.00  0.00           N
ATOM     66  CA  SER A   6       8.091   4.274  -0.631  1.00  0.00           C
ATOM     67  C   SER A   6       6.809   5.094  -0.551  1.00  0.00           C
ATOM     68  O   SER A   6       5.714   4.615  -0.869  1.00  0.00           O
ATOM     69  CB  SER A   6       8.765   4.550  -1.995  1.00  0.00           C
ATOM     70  OG  SER A   6       7.904   4.281  -3.102  1.00  0.00           O
ATOM     71  H   SER A   6       6.806   2.633  -0.305  1.00  0.00           H
ATOM     72  HA  SER A   6       8.790   4.596   0.158  1.00  0.00           H
ATOM     73  HB1 SER A   6       9.045   5.611  -2.064  1.00  0.00           H
ATOM     74  HB2 SER A   6       9.682   3.945  -2.047  1.00  0.00           H
ATOM     75  H   SER A   6       7.762   3.319  -3.126  1.00  0.00           H
ATOM     76  N   ILE A   7       7.009   6.405  -0.250  1.00  0.00           N
ATOM     77  CA  ILE A   7       5.904   7.360  -0.292  1.00  0.00           C
ATOM     78  C   ILE A   7       5.288   7.414  -1.690  1.00  0.00           C
ATOM     79  O   ILE A   7       4.077   7.654  -1.816  1.00  0.00           O
ATOM     80  CB  ILE A   7       6.385   8.770   0.169  1.00  0.00           C
ATOM     81  CG1 ILE A   7       6.611   8.774   1.697  1.00  0.00           C
ATOM     82  CG2 ILE A   7       5.429   9.888  -0.269  1.00  0.00           C
ATOM     83  CD1 ILE A   7       7.327  10.014   2.236  1.00  0.00           C
ATOM     84  H   ILE A   7       7.737   6.533   0.474  1.00  0.00           H
ATOM     85  HA  ILE A   7       5.077   7.032   0.359  1.00  0.00           H
ATOM     86  HB  ILE A   7       7.359   8.939  -0.327  1.00  0.00           H
ATOM     87 HG11 ILE A   7       5.630   8.660   2.195  1.00  0.00           H
ATOM     88 HG12 ILE A   7       7.204   7.895   1.981  1.00  0.00           H
ATOM     89 HG21 ILE A   7       5.365   9.985  -1.362  1.00  0.00           H
ATOM     90 HG22 ILE A   7       5.776  10.857   0.116  1.00  0.00           H
ATOM     91 HG23 ILE A   7       4.409   9.719   0.110  1.00  0.00           H
ATOM     92 HD11 ILE A   7       8.303  10.157   1.743  1.00  0.00           H
ATOM     93 HD12 ILE A   7       7.518   9.906   3.315  1.00  0.00           H
ATOM     94 HD13 ILE A   7       6.744  10.938   2.099  1.00  0.00           H
ATOM     95  N   VAL A   8       6.093   7.247  -2.756  1.00  0.00           N
ATOM     96  CA  VAL A   8       5.562   7.224  -4.121  1.00  0.00           C
ATOM     97  C   VAL A   8       4.579   6.052  -4.308  1.00  0.00           C
ATOM     98  O   VAL A   8       3.460   6.240  -4.797  1.00  0.00           O
ATOM     99  CB  VAL A   8       6.704   7.195  -5.164  1.00  0.00           C
ATOM    100  CG1 VAL A   8       6.142   6.973  -6.576  1.00  0.00           C
ATOM    101  CG2 VAL A   8       7.545   8.477  -5.095  1.00  0.00           C
ATOM    102  H   VAL A   8       7.058   6.982  -2.586  1.00  0.00           H
ATOM    103  HA  VAL A   8       4.950   8.127  -4.266  1.00  0.00           H
ATOM    104  HB  VAL A   8       7.352   6.334  -4.914  1.00  0.00           H
ATOM    105 HG11 VAL A   8       5.634   6.003  -6.674  1.00  0.00           H
ATOM    106 HG12 VAL A   8       6.955   7.002  -7.316  1.00  0.00           H
ATOM    107 HG13 VAL A   8       5.410   7.753  -6.843  1.00  0.00           H
ATOM    108 HG21 VAL A   8       8.394   8.421  -5.794  1.00  0.00           H
ATOM    109 HG22 VAL A   8       7.958   8.659  -4.091  1.00  0.00           H
ATOM    110 HG23 VAL A   8       6.942   9.359  -5.370  1.00  0.00           H
ATOM    111  N   ALA A   9       5.025   4.839  -3.922  1.00  0.00           N
ATOM    112  CA  ALA A   9       4.155   3.667  -3.958  1.00  0.00           C
ATOM    113  C   ALA A   9       2.876   3.909  -3.135  1.00  0.00           C
ATOM    114  O   ALA A   9       1.777   3.569  -3.590  1.00  0.00           O
```

ATOM	115	CB	ALA	A	9	4.915	2.420	-3.502	1.00	0.00	C
ATOM	116	H	ALA	A	9	5.948	4.753	-3.492	1.00	0.00	H
ATOM	117	HA	ALA	A	9	3.792	3.526	-4.986	1.00	0.00	H
ATOM	118	HB1	ALA	A	9	5.702	2.166	-4.228	1.00	0.00	H
ATOM	119	HB2	ALA	A	9	4.247	1.551	-3.454	1.00	0.00	H
ATOM	120	HB3	ALA	A	9	5.367	2.584	-2.513	1.00	0.00	H
ATOM	121	N	ARG	A	10	3.026	4.500	-1.933	1.00	0.00	N
ATOM	122	CA	ARG	A	10	1.881	4.824	-1.093	1.00	0.00	C
ATOM	123	C	ARG	A	10	0.913	5.790	-1.775	1.00	0.00	C
ATOM	124	O	ARG	A	10	-0.312	5.636	-1.644	1.00	0.00	O
ATOM	125	CB	ARG	A	10	2.345	5.423	0.244	1.00	0.00	C
ATOM	126	CG	ARG	A	10	1.194	5.798	1.187	1.00	0.00	C
ATOM	127	CD	ARG	A	10	0.304	4.642	1.649	1.00	0.00	C
ATOM	128	NE	ARG	A	10	1.041	3.785	2.569	1.00	0.00	N
ATOM	129	CZ	ARG	A	10	0.518	2.740	3.216	1.00	0.00	C
ATOM	130	NH1	ARG	A	10	-0.754	2.348	2.975	1.00	0.00	N
ATOM	131	NH2	ARG	A	10	1.246	2.115	4.138	1.00	0.00	N
ATOM	132	H	ARG	A	10	3.966	4.703	-1.584	1.00	0.00	H
ATOM	133	HA	ARG	A	10	1.310	3.905	-0.915	1.00	0.00	H
ATOM	134	HB1	ARG	A	10	3.028	4.721	0.740	1.00	0.00	H
ATOM	135	HB2	ARG	A	10	2.929	6.331	0.034	1.00	0.00	H
ATOM	136	HG1	ARG	A	10	1.628	6.287	2.074	1.00	0.00	H
ATOM	137	HG2	ARG	A	10	0.545	6.542	0.703	1.00	0.00	H
ATOM	138	HD1	ARG	A	10	-0.598	5.044	2.139	1.00	0.00	H
ATOM	139	HD2	ARG	A	10	-0.057	4.066	0.780	1.00	0.00	H
ATOM	140	HE	ARG	A	10	2.032	4.043	2.787	1.00	0.00	H
ATOM	141	HH11	ARG	A	10	-1.209	2.661	2.125	1.00	0.00	H
ATOM	142	HH12	ARG	A	10	-1.076	1.446	3.329	1.00	0.00	H
ATOM	143	HH21	ARG	A	10	2.255	2.356	4.230	1.00	0.00	H
ATOM	144	HH22	ARG	A	10	0.858	1.303	4.624	1.00	0.00	H
ATOM	145	N	SER	A	11	1.453	6.823	-2.436	1.00	0.00	N
ATOM	146	CA	SER	A	11	0.641	7.816	-3.122	1.00	0.00	C
ATOM	147	C	SER	A	11	-0.217	7.140	-4.201	1.00	0.00	C
ATOM	148	O	SER	A	11	-1.428	7.376	-4.300	1.00	0.00	O
ATOM	149	CB	SER	A	11	1.539	8.925	-3.706	1.00	0.00	C
ATOM	150	OG	SER	A	11	2.213	9.657	-2.706	1.00	0.00	O
ATOM	151	H	SER	A	11	2.469	6.912	-2.455	1.00	0.00	H
ATOM	152	HA	SER	A	11	-0.066	8.268	-2.411	1.00	0.00	H
ATOM	153	HB1	SER	A	11	2.235	8.480	-4.439	1.00	0.00	H
ATOM	154	HB2	SER	A	11	0.890	9.630	-4.247	1.00	0.00	H
ATOM	155	H	SER	A	11	2.930	9.096	-2.353	1.00	0.00	H
ATOM	156	N	ASN	A	12	0.445	6.281	-4.997	1.00	0.00	N
ATOM	157	CA	ASN	A	12	-0.229	5.506	-6.028	1.00	0.00	C
ATOM	158	C	ASN	A	12	-1.317	4.603	-5.412	1.00	0.00	C
ATOM	159	O	ASN	A	12	-2.441	4.518	-5.926	1.00	0.00	O
ATOM	160	CB	ASN	A	12	0.792	4.666	-6.797	1.00	0.00	C
ATOM	161	CG	ASN	A	12	1.784	5.505	-7.600	1.00	0.00	C
ATOM	162	OD1	ASN	A	12	1.639	6.698	-7.818	1.00	0.00	O
ATOM	163	ND2	ASN	A	12	2.870	4.800	-8.049	1.00	0.00	N
ATOM	164	H	ASN	A	12	1.454	6.166	-4.878	1.00	0.00	H
ATOM	165	HA	ASN	A	12	-0.750	6.182	-6.719	1.00	0.00	H
ATOM	166	HB1	ASN	A	12	1.339	4.010	-6.103	1.00	0.00	H
ATOM	167	HB2	ASN	A	12	0.253	4.016	-7.503	1.00	0.00	H
ATOM	168	HD21	ASN	A	12	2.881	3.786	-8.037	1.00	0.00	H
ATOM	169	HD22	ASN	A	12	3.479	5.267	-8.713	1.00	0.00	H
ATOM	170	N	PHE	A	13	-0.957	3.920	-4.310	1.00	0.00	N
ATOM	171	CA	PHE	A	13	-1.876	3.059	-3.577	1.00	0.00	C
ATOM	172	C	PHE	A	13	-3.116	3.848	-3.135	1.00	0.00	C
ATOM	173	O	PHE	A	13	-4.253	3.380	-3.296	1.00	0.00	O
ATOM	174	CB	PHE	A	13	-1.113	2.426	-2.384	1.00	0.00	C
ATOM	175	CG	PHE	A	13	-2.004	1.778	-1.352	1.00	0.00	C
ATOM	176	CD2	PHE	A	13	-2.581	2.556	-0.333	1.00	0.00	C
ATOM	177	CD1	PHE	A	13	-2.306	0.410	-1.406	1.00	0.00	C

```
ATOM    178  CE2 PHE A  13      -3.475   1.985   0.574  1.00  0.00           C
ATOM    179  CE1 PHE A  13      -3.205  -0.160  -0.501  1.00  0.00           C
ATOM    180  CZ  PHE A  13      -3.809   0.629   0.485  1.00  0.00           C
ATOM    181  H   PHE A  13       0.005   3.990  -3.962  1.00  0.00           H
ATOM    182  HA  PHE A  13      -2.254   2.266  -4.240  1.00  0.00           H
ATOM    183  HB1 PHE A  13      -0.396   1.699  -2.787  1.00  0.00           H
ATOM    184  HB2 PHE A  13      -0.532   3.223  -1.906  1.00  0.00           H
ATOM    185  HD2 PHE A  13      -2.368   3.623  -0.263  1.00  0.00           H
ATOM    186  HD1 PHE A  13      -1.842  -0.203  -2.181  1.00  0.00           H
ATOM    187  HE2 PHE A  13      -3.938   2.619   1.335  1.00  0.00           H
ATOM    188  HE1 PHE A  13      -3.467  -1.218  -0.577  1.00  0.00           H
ATOM    189  HZ  PHE A  13      -4.582   0.184   1.117  1.00  0.00           H
ATOM    190  N   ASN A  14      -2.871   5.037  -2.564  1.00  0.00           N
ATOM    191  CA  ASN A  14      -3.932   5.874  -2.037  1.00  0.00           C
ATOM    192  C   ASN A  14      -4.902   6.327  -3.141  1.00  0.00           C
ATOM    193  O   ASN A  14      -6.121   6.295  -2.947  1.00  0.00           O
ATOM    194  CB  ASN A  14      -3.347   7.099  -1.332  1.00  0.00           C
ATOM    195  CG  ASN A  14      -2.639   6.785  -0.015  1.00  0.00           C
ATOM    196  OD1 ASN A  14      -2.770   5.726   0.590  1.00  0.00           O
ATOM    197  ND2 ASN A  14      -1.902   7.818   0.481  1.00  0.00           N
ATOM    198  H   ASN A  14      -1.899   5.323  -2.406  1.00  0.00           H
ATOM    199  HA  ASN A  14      -4.539   5.296  -1.326  1.00  0.00           H
ATOM    200  HB1 ASN A  14      -2.668   7.636  -2.014  1.00  0.00           H
ATOM    201  HB2 ASN A  14      -4.177   7.786  -1.098  1.00  0.00           H
ATOM    202 HD21 ASN A  14      -1.680   8.633  -0.078  1.00  0.00           H
ATOM    203 HD22 ASN A  14      -1.384   7.670   1.339  1.00  0.00           H
ATOM    204  N   VAL A  15      -4.338   6.764  -4.288  1.00  0.00           N
ATOM    205  CA  VAL A  15      -5.155   7.132  -5.440  1.00  0.00           C
ATOM    206  C   VAL A  15      -6.014   5.939  -5.895  1.00  0.00           C
ATOM    207  O   VAL A  15      -7.222   6.056  -6.117  1.00  0.00           O
ATOM    208  CB  VAL A  15      -4.269   7.692  -6.581  1.00  0.00           C
ATOM    209  CG1 VAL A  15      -5.059   7.815  -7.889  1.00  0.00           C
ATOM    210  CG2 VAL A  15      -3.650   9.038  -6.179  1.00  0.00           C
ATOM    211  H   VAL A  15      -3.319   6.760  -4.383  1.00  0.00           H
ATOM    212  HA  VAL A  15      -5.886   7.896  -5.139  1.00  0.00           H
ATOM    213  HB  VAL A  15      -3.453   6.964  -6.740  1.00  0.00           H
ATOM    214 HG11 VAL A  15      -4.427   8.257  -8.674  1.00  0.00           H
ATOM    215 HG12 VAL A  15      -5.945   8.460  -7.766  1.00  0.00           H
ATOM    216 HG13 VAL A  15      -5.410   6.840  -8.258  1.00  0.00           H
ATOM    217 HG21 VAL A  15      -2.978   9.402  -6.972  1.00  0.00           H
ATOM    218 HG22 VAL A  15      -3.056   8.963  -5.259  1.00  0.00           H
ATOM    219 HG23 VAL A  15      -4.434   9.799  -6.028  1.00  0.00           H
ATOM    220  N   CYS A  16      -5.354   4.765  -6.027  1.00  0.00           N
ATOM    221  CA  CYS A  16      -6.041   3.529  -6.365  1.00  0.00           C
ATOM    222  C   CYS A  16      -7.206   3.233  -5.407  1.00  0.00           C
ATOM    223  O   CYS A  16      -8.273   2.763  -5.830  1.00  0.00           O
ATOM    224  CB  CYS A  16      -4.980   2.415  -6.377  1.00  0.00           C
ATOM    225  SG  CYS A  16      -5.593   0.707  -6.616  1.00  0.00           S
ATOM    226  H   CYS A  16      -4.350   4.723  -5.832  1.00  0.00           H
ATOM    227  HA  CYS A  16      -6.517   3.609  -7.354  1.00  0.00           H
ATOM    228  HB1 CYS A  16      -4.296   2.582  -7.220  1.00  0.00           H
ATOM    229  HB2 CYS A  16      -4.393   2.453  -5.451  1.00  0.00           H
ATOM    230  N   ARG A  17      -7.018   3.513  -4.108  1.00  0.00           N
ATOM    231  CA  ARG A  17      -8.056   3.221  -3.141  1.00  0.00           C
ATOM    232  C   ARG A  17      -9.307   4.092  -3.278  1.00  0.00           C
ATOM    233  O   ARG A  17     -10.370   3.648  -2.798  1.00  0.00           O
ATOM    234  CB  ARG A  17      -7.530   3.340  -1.699  1.00  0.00           C
ATOM    235  CG  ARG A  17      -6.622   2.187  -1.269  1.00  0.00           C
ATOM    236  CD  ARG A  17      -7.306   0.824  -1.245  1.00  0.00           C
ATOM    237  NE  ARG A  17      -8.323   0.670  -0.186  1.00  0.00           N
ATOM    238  CZ  ARG A  17      -9.608   0.349  -0.396  1.00  0.00           C
ATOM    239  NH1 ARG A  17     -10.359   0.895  -1.357  1.00  0.00           N
ATOM    240  NH2 ARG A  17     -10.170  -0.566   0.423  1.00  0.00           N
```

ATOM	241	H	ARG	A	17	-6.087	3.784	-3.780	1.00	0.00	H
ATOM	242	HA	ARG	A	17	-8.429	2.204	-3.339	1.00	0.00	H
ATOM	243	HB1	ARG	A	17	-7.010	4.306	-1.603	1.00	0.00	H
ATOM	244	HB2	ARG	A	17	-8.402	3.387	-1.028	1.00	0.00	H
ATOM	245	HG1	ARG	A	17	-5.746	2.119	-1.932	1.00	0.00	H
ATOM	246	HG2	ARG	A	17	-6.220	2.404	-0.267	1.00	0.00	H
ATOM	247	HD1	ARG	A	17	-7.774	0.577	-2.207	1.00	0.00	H
ATOM	248	HD2	ARG	A	17	-6.548	0.051	-1.044	1.00	0.00	H
ATOM	249	HE	ARG	A	17	-7.967	0.284	0.705	1.00	0.00	H
ATOM	250	HH11	ARG	A	17	-10.081	1.728	-1.878	1.00	0.00	H
ATOM	251	HH12	ARG	A	17	-11.110	0.331	-1.759	1.00	0.00	H
ATOM	252	HH21	ARG	A	17	-9.554	-0.949	1.166	1.00	0.00	H
ATOM	253	HH22	ARG	A	17	-11.172	-0.517	0.572	1.00	0.00	H
ATOM	254	N	LEU	A	18	-9.185	5.300	-3.842	1.00	0.00	N
ATOM	255	CA	LEU	A	18	-10.252	6.301	-3.843	1.00	0.00	C
ATOM	256	C	LEU	A	18	-11.582	5.834	-4.466	1.00	0.00	C
ATOM	257	O	LEU	A	18	-12.628	6.095	-3.875	1.00	0.00	O
ATOM	258	CB	LEU	A	18	-9.782	7.614	-4.487	1.00	0.00	C
ATOM	259	CG	LEU	A	18	-8.632	8.346	-3.768	1.00	0.00	C
ATOM	260	CD1	LEU	A	18	-8.214	9.573	-4.588	1.00	0.00	C
ATOM	261	CD2	LEU	A	18	-8.979	8.729	-2.325	1.00	0.00	C
ATOM	262	H	LEU	A	18	-8.284	5.554	-4.246	1.00	0.00	H
ATOM	263	HA	LEU	A	18	-10.547	6.486	-2.800	1.00	0.00	H
ATOM	264	HB1	LEU	A	18	-9.470	7.416	-5.524	1.00	0.00	H
ATOM	265	HB2	LEU	A	18	-10.665	8.274	-4.536	1.00	0.00	H
ATOM	266	HG	LEU	A	18	-7.766	7.668	-3.724	1.00	0.00	H
ATOM	267	HD11	LEU	A	18	-7.938	9.289	-5.617	1.00	0.00	H
ATOM	268	HD12	LEU	A	18	-7.345	10.079	-4.136	1.00	0.00	H
ATOM	269	HD13	LEU	A	18	-9.029	10.314	-4.656	1.00	0.00	H
ATOM	270	HD21	LEU	A	18	-8.144	9.274	-1.854	1.00	0.00	H
ATOM	271	HD22	LEU	A	18	-9.173	7.844	-1.699	1.00	0.00	H
ATOM	272	HD23	LEU	A	18	-9.871	9.377	-2.277	1.00	0.00	H
ATOM	273	N	PRO	A	19	-11.575	5.129	-5.638	1.00	0.00	N
ATOM	274	CA	PRO	A	19	-12.855	4.637	-6.169	1.00	0.00	C
ATOM	275	C	PRO	A	19	-13.315	3.312	-5.533	1.00	0.00	C
ATOM	276	O	PRO	A	19	-14.281	2.706	-5.989	1.00	0.00	O
ATOM	277	CB	PRO	A	19	-12.592	4.483	-7.675	1.00	0.00	C
ATOM	278	CG	PRO	A	19	-11.099	4.142	-7.728	1.00	0.00	C
ATOM	279	CD	PRO	A	19	-10.490	5.030	-6.640	1.00	0.00	C
ATOM	280	HA	PRO	A	19	-13.654	5.359	-5.948	1.00	0.00	H
ATOM	281	HB1	PRO	A	19	-13.246	3.722	-8.118	1.00	0.00	H
ATOM	282	HB2	PRO	A	19	-12.789	5.442	-8.181	1.00	0.00	H
ATOM	283	HG1	PRO	A	19	-10.941	3.078	-7.488	1.00	0.00	H
ATOM	284	HG2	PRO	A	19	-10.641	4.325	-8.710	1.00	0.00	H
ATOM	285	HD1	PRO	A	19	-9.576	4.610	-6.217	1.00	0.00	H
ATOM	286	HD2	PRO	A	19	-10.254	6.031	-7.040	1.00	0.00	H
ATOM	287	N	GLY	A	20	-12.593	2.869	-4.464	1.00	0.00	N
ATOM	288	CA	GLY	A	20	-13.001	1.719	-3.703	1.00	0.00	C
ATOM	289	C	GLY	A	20	-12.147	0.471	-3.904	1.00	0.00	C
ATOM	290	O	GLY	A	20	-12.325	-0.534	-3.193	1.00	0.00	O
ATOM	291	H	GLY	A	20	-11.856	3.444	-4.053	1.00	0.00	H
ATOM	292	HA1	GLY	A	20	-13.007	1.947	-2.626	1.00	0.00	H
ATOM	293	HA2	GLY	A	20	-14.025	1.445	-3.999	1.00	0.00	H
ATOM	294	N	THR	A	21	-11.205	0.534	-4.861	1.00	0.00	N
ATOM	295	CA	THR	A	21	-10.501	-0.628	-5.359	1.00	0.00	C
ATOM	296	C	THR	A	21	-9.988	-1.505	-4.211	1.00	0.00	C
ATOM	297	O	THR	A	21	-9.243	-1.066	-3.334	1.00	0.00	O
ATOM	298	CB	THR	A	21	-9.294	-0.175	-6.218	1.00	0.00	C
ATOM	299	OG1	THR	A	21	-9.714	0.851	-7.101	1.00	0.00	O
ATOM	300	CG2	THR	A	21	-8.705	-1.351	-6.987	1.00	0.00	C
ATOM	301	H	THR	A	21	-11.133	1.370	-5.437	1.00	0.00	H
ATOM	302	HA	THR	A	21	-11.184	-1.200	-6.006	1.00	0.00	H
ATOM	303	HB	THR	A	21	-8.550	0.228	-5.518	1.00	0.00	H

```
ATOM    304  HG1 THR A  21      -9.134   1.620  -6.909  1.00  0.00           H
ATOM    305 HG21 THR A  21      -8.398  -2.157  -6.304  1.00  0.00           H
ATOM    306 HG22 THR A  21      -7.823  -1.024  -7.554  1.00  0.00           H
ATOM    307 HG23 THR A  21      -9.439  -1.749  -7.707  1.00  0.00           H
ATOM    308  N   PRO A  22     -10.290  -2.834  -4.267  1.00  0.00           N
ATOM    309  CA  PRO A  22      -9.824  -3.699  -3.195  1.00  0.00           C
ATOM    310  C   PRO A  22      -8.313  -3.624  -2.964  1.00  0.00           C
ATOM    311  O   PRO A  22      -7.493  -3.619  -3.888  1.00  0.00           O
ATOM    312  CB  PRO A  22     -10.258  -5.098  -3.664  1.00  0.00           C
ATOM    313  CG  PRO A  22     -11.583  -4.814  -4.379  1.00  0.00           C
ATOM    314  CD  PRO A  22     -11.335  -3.475  -5.087  1.00  0.00           C
ATOM    315  HA  PRO A  22     -10.336  -3.427  -2.252  1.00  0.00           H
ATOM    316  HB1 PRO A  22      -9.509  -5.486  -4.375  1.00  0.00           H
ATOM    317  HB2 PRO A  22     -10.347  -5.808  -2.830  1.00  0.00           H
ATOM    318  HG1 PRO A  22     -11.875  -5.609  -5.079  1.00  0.00           H
ATOM    319  HG2 PRO A  22     -12.393  -4.704  -3.640  1.00  0.00           H
ATOM    320  HD1 PRO A  22     -10.977  -3.622  -6.118  1.00  0.00           H
ATOM    321  HD2 PRO A  22     -12.240  -2.853  -5.112  1.00  0.00           H
ATOM    322  N   GLU A  23      -7.972  -3.743  -1.655  1.00  0.00           N
ATOM    323  CA  GLU A  23      -6.615  -3.615  -1.127  1.00  0.00           C
ATOM    324  C   GLU A  23      -5.588  -4.395  -1.941  1.00  0.00           C
ATOM    325  O   GLU A  23      -4.486  -3.904  -2.195  1.00  0.00           O
ATOM    326  CB  GLU A  23      -6.558  -4.113   0.335  1.00  0.00           C
ATOM    327  CG  GLU A  23      -7.444  -3.346   1.316  1.00  0.00           C
ATOM    328  CD  GLU A  23      -7.094  -1.847   1.443  1.00  0.00           C
ATOM    329  OE1 GLU A  23      -5.968  -1.484   1.029  1.00  0.00           O
ATOM    330  OE2 GLU A  23      -7.991  -1.104   1.948  1.00  0.00           O
ATOM    331  H   GLU A  23      -8.713  -3.546  -0.988  1.00  0.00           H
ATOM    332  HA  GLU A  23      -6.286  -2.567  -1.155  1.00  0.00           H
ATOM    333  HB1 GLU A  23      -6.812  -5.186   0.357  1.00  0.00           H
ATOM    334  HB2 GLU A  23      -5.513  -4.013   0.655  1.00  0.00           H
ATOM    335  HG1 GLU A  23      -8.519  -3.441   1.082  1.00  0.00           H
ATOM    336  HG2 GLU A  23      -7.341  -3.798   2.317  1.00  0.00           H
ATOM    337  N   ALA A  24      -5.903  -5.672  -2.278  1.00  0.00           N
ATOM    338  CA  ALA A  24      -4.950  -6.544  -2.951  1.00  0.00           C
ATOM    339  C   ALA A  24      -4.503  -5.993  -4.315  1.00  0.00           C
ATOM    340  O   ALA A  24      -3.342  -6.176  -4.692  1.00  0.00           O
ATOM    341  CB  ALA A  24      -5.529  -7.950  -3.107  1.00  0.00           C
ATOM    342  H   ALA A  24      -6.833  -6.014  -2.060  1.00  0.00           H
ATOM    343  HA  ALA A  24      -4.029  -6.590  -2.355  1.00  0.00           H
ATOM    344  HB1 ALA A  24      -5.756  -8.390  -2.124  1.00  0.00           H
ATOM    345  HB2 ALA A  24      -4.797  -8.596  -3.612  1.00  0.00           H
ATOM    346  HB3 ALA A  24      -6.449  -7.938  -3.713  1.00  0.00           H
ATOM    347  N   LEU A  25      -5.451  -5.391  -5.066  1.00  0.00           N
ATOM    348  CA  LEU A  25      -5.115  -4.798  -6.357  1.00  0.00           C
ATOM    349  C   LEU A  25      -4.272  -3.526  -6.201  1.00  0.00           C
ATOM    350  O   LEU A  25      -3.357  -3.274  -6.984  1.00  0.00           O
ATOM    351  CB  LEU A  25      -6.381  -4.497  -7.173  1.00  0.00           C
ATOM    352  CG  LEU A  25      -7.313  -5.696  -7.430  1.00  0.00           C
ATOM    353  CD1 LEU A  25      -8.547  -5.234  -8.216  1.00  0.00           C
ATOM    354  CD2 LEU A  25      -6.600  -6.860  -8.128  1.00  0.00           C
ATOM    355  H   LEU A  25      -6.307  -5.075  -4.606  1.00  0.00           H
ATOM    356  HA  LEU A  25      -4.452  -5.494  -6.886  1.00  0.00           H
ATOM    357  HB1 LEU A  25      -6.949  -3.703  -6.664  1.00  0.00           H
ATOM    358  HB2 LEU A  25      -6.048  -4.069  -8.135  1.00  0.00           H
ATOM    359  HG  LEU A  25      -7.668  -6.063  -6.448  1.00  0.00           H
ATOM    360 HD11 LEU A  25      -9.067  -4.412  -7.699  1.00  0.00           H
ATOM    361 HD12 LEU A  25      -9.267  -6.057  -8.353  1.00  0.00           H
ATOM    362 HD13 LEU A  25      -8.269  -4.867  -9.218  1.00  0.00           H
ATOM    363 HD21 LEU A  25      -7.299  -7.687  -8.333  1.00  0.00           H
ATOM    364 HD22 LEU A  25      -5.782  -7.270  -7.516  1.00  0.00           H
ATOM    365 HD23 LEU A  25      -6.170  -6.542  -9.093  1.00  0.00           H
ATOM    366  N   CYS A  26      -4.586  -2.706  -5.167  1.00  0.00           N
```

ATOM	367	CA	CYS	A	26	-3.778	-1.525	-4.934	1.00	0.00	C
ATOM	368	C	CYS	A	26	-2.362	-1.879	-4.448	1.00	0.00	C
ATOM	369	O	CYS	A	26	-1.430	-1.101	-4.705	1.00	0.00	O
ATOM	370	CB	CYS	A	26	-4.476	-0.525	-4.018	1.00	0.00	C
ATOM	371	SG	CYS	A	26	-6.085	0.064	-4.693	1.00	0.00	S
ATOM	372	H	CYS	A	26	-5.403	-2.889	-4.588	1.00	0.00	H
ATOM	373	HA	CYS	A	26	-3.600	-1.034	-5.903	1.00	0.00	H
ATOM	374	HB1	CYS	A	26	-4.736	-0.972	-3.044	1.00	0.00	H
ATOM	375	HB2	CYS	A	26	-3.822	0.334	-3.824	1.00	0.00	H
ATOM	376	N	ALA	A	27	-2.219	-3.038	-3.789	1.00	0.00	N
ATOM	377	CA	ALA	A	27	-0.925	-3.611	-3.434	1.00	0.00	C
ATOM	378	C	ALA	A	27	-0.139	-4.032	-4.694	1.00	0.00	C
ATOM	379	O	ALA	A	27	0.999	-3.594	-4.890	1.00	0.00	O
ATOM	380	CB	ALA	A	27	-1.113	-4.769	-2.453	1.00	0.00	C
ATOM	381	H	ALA	A	27	-3.059	-3.550	-3.521	1.00	0.00	H
ATOM	382	HA	ALA	A	27	-0.298	-2.835	-2.978	1.00	0.00	H
ATOM	383	HB1	ALA	A	27	-1.612	-4.409	-1.541	1.00	0.00	H
ATOM	384	HB2	ALA	A	27	-0.127	-5.168	-2.185	1.00	0.00	H
ATOM	385	HB3	ALA	A	27	-1.726	-5.566	-2.898	1.00	0.00	H
ATOM	386	N	THR	A	28	-0.739	-4.891	-5.549	1.00	0.00	N
ATOM	387	CA	THR	A	28	-0.010	-5.367	-6.720	1.00	0.00	C
ATOM	388	C	THR	A	28	0.355	-4.256	-7.732	1.00	0.00	C
ATOM	389	O	THR	A	28	1.386	-4.351	-8.390	1.00	0.00	O
ATOM	390	CB	THR	A	28	-0.727	-6.523	-7.428	1.00	0.00	C
ATOM	391	OG1	THR	A	28	-1.977	-6.014	-7.900	1.00	0.00	O
ATOM	392	CG2	THR	A	28	-0.912	-7.765	-6.556	1.00	0.00	C
ATOM	393	H	THR	A	28	-1.704	-5.193	-5.399	1.00	0.00	H
ATOM	394	HA	THR	A	28	0.973	-5.738	-6.392	1.00	0.00	H
ATOM	395	HB	THR	A	28	-0.077	-6.775	-8.286	1.00	0.00	H
ATOM	396	HG1	THR	A	28	-2.363	-6.673	-8.498	1.00	0.00	H
ATOM	397	HG21	THR	A	28	0.058	-8.135	-6.188	1.00	0.00	H
ATOM	398	HG22	THR	A	28	-1.373	-8.574	-7.147	1.00	0.00	H
ATOM	399	HG23	THR	A	28	-1.567	-7.559	-5.698	1.00	0.00	H
ATOM	400	N	TYR	A	29	-0.495	-3.202	-7.803	1.00	0.00	N
ATOM	401	CA	TYR	A	29	-0.228	-2.054	-8.659	1.00	0.00	C
ATOM	402	C	TYR	A	29	1.059	-1.300	-8.268	1.00	0.00	C
ATOM	403	O	TYR	A	29	1.664	-0.629	-9.097	1.00	0.00	O
ATOM	404	CB	TYR	A	29	-1.479	-1.137	-8.574	1.00	0.00	C
ATOM	405	CG	TYR	A	29	-1.366	0.294	-9.052	1.00	0.00	C
ATOM	406	CD1	TYR	A	29	-0.742	0.664	-10.249	1.00	0.00	C
ATOM	407	CD2	TYR	A	29	-1.907	1.318	-8.262	1.00	0.00	C
ATOM	408	CE1	TYR	A	29	-0.629	2.003	-10.622	1.00	0.00	C
ATOM	409	CE2	TYR	A	29	-1.811	2.661	-8.619	1.00	0.00	C
ATOM	410	CZ	TYR	A	29	-1.148	3.008	-9.800	1.00	0.00	C
ATOM	411	OH	TYR	A	29	-1.019	4.343	-10.076	1.00	0.00	O
ATOM	412	H	TYR	A	29	-1.426	-3.295	-7.393	1.00	0.00	H
ATOM	413	HA	TYR	A	29	-0.054	-2.381	-9.696	1.00	0.00	H
ATOM	414	HB1	TYR	A	29	-2.293	-1.658	-9.106	1.00	0.00	H
ATOM	415	HB2	TYR	A	29	-1.788	-1.116	-7.520	1.00	0.00	H
ATOM	416	HD1	TYR	A	29	-0.295	-0.096	-10.889	1.00	0.00	H
ATOM	417	HD2	TYR	A	29	-2.399	1.058	-7.322	1.00	0.00	H
ATOM	418	HE1	TYR	A	29	-0.114	2.269	-11.551	1.00	0.00	H
ATOM	419	HE2	TYR	A	29	-2.211	3.448	-7.980	1.00	0.00	H
ATOM	420	HH	TYR	A	29	-0.521	4.451	-10.901	1.00	0.00	H
ATOM	421	N	THR	A	30	1.415	-1.371	-6.954	1.00	0.00	N
ATOM	422	CA	THR	A	30	2.351	-0.433	-6.371	1.00	0.00	C
ATOM	423	C	THR	A	30	3.598	-1.036	-5.723	1.00	0.00	C
ATOM	424	O	THR	A	30	4.501	-0.298	-5.324	1.00	0.00	O
ATOM	425	CB	THR	A	30	1.632	0.432	-5.308	1.00	0.00	C
ATOM	426	OG1	THR	A	30	1.204	-0.402	-4.224	1.00	0.00	O
ATOM	427	CG2	THR	A	30	0.476	1.211	-5.909	1.00	0.00	C
ATOM	428	H	THR	A	30	0.845	-1.936	-6.329	1.00	0.00	H
ATOM	429	HA	THR	A	30	2.706	0.211	-7.187	1.00	0.00	H

```
ATOM    430  HB  THR A  30       2.373   1.123  -4.883  1.00  0.00           H
ATOM    431  HG1 THR A  30       0.286  -0.712  -4.393  1.00  0.00           H
ATOM    432 HG21 THR A  30       0.780   1.713  -6.837  1.00  0.00           H
ATOM    433 HG22 THR A  30       0.119   1.966  -5.205  1.00  0.00           H
ATOM    434 HG23 THR A  30      -0.356   0.544  -6.156  1.00  0.00           H
ATOM    435  N   GLY A  31       3.605  -2.380  -5.539  1.00  0.00           N
ATOM    436  CA  GLY A  31       4.569  -2.985  -4.655  1.00  0.00           C
ATOM    437  C   GLY A  31       4.290  -2.809  -3.163  1.00  0.00           C
ATOM    438  O   GLY A  31       5.093  -3.257  -2.329  1.00  0.00           O
ATOM    439  H   GLY A  31       2.781  -2.919  -5.797  1.00  0.00           H
ATOM    440  HA1 GLY A  31       4.612  -4.067  -4.840  1.00  0.00           H
ATOM    441  HA2 GLY A  31       5.569  -2.569  -4.844  1.00  0.00           H
ATOM    442  N   CYS A  32       3.163  -2.171  -2.801  1.00  0.00           N
ATOM    443  CA  CYS A  32       2.723  -2.212  -1.424  1.00  0.00           C
ATOM    444  C   CYS A  32       2.300  -3.669  -1.071  1.00  0.00           C
ATOM    445  O   CYS A  32       1.984  -4.475  -1.943  1.00  0.00           O
ATOM    446  CB  CYS A  32       1.567  -1.240  -1.157  1.00  0.00           C
ATOM    447  SG  CYS A  32       1.910   0.538  -1.547  1.00  0.00           S
ATOM    448  H   CYS A  32       2.478  -1.916  -3.514  1.00  0.00           H
ATOM    449  HA  CYS A  32       3.562  -1.943  -0.773  1.00  0.00           H
ATOM    450  HB1 CYS A  32       0.693  -1.494  -1.773  1.00  0.00           H
ATOM    451  HB2 CYS A  32       1.274  -1.298  -0.102  1.00  0.00           H
ATOM    452  N   ILE A  33       2.313  -3.957   0.252  1.00  0.00           N
ATOM    453  CA  ILE A  33       1.980  -5.270   0.747  1.00  0.00           C
ATOM    454  C   ILE A  33       0.868  -5.165   1.796  1.00  0.00           C
ATOM    455  O   ILE A  33       0.483  -4.087   2.269  1.00  0.00           O
ATOM    456  CB  ILE A  33       3.224  -6.052   1.276  1.00  0.00           C
ATOM    457  CG1 ILE A  33       3.823  -5.350   2.510  1.00  0.00           C
ATOM    458  CG2 ILE A  33       4.214  -6.293   0.130  1.00  0.00           C
ATOM    459  CD1 ILE A  33       5.067  -6.007   3.106  1.00  0.00           C
ATOM    460  H   ILE A  33       2.566  -3.222   0.917  1.00  0.00           H
ATOM    461  HA  ILE A  33       1.574  -5.825  -0.112  1.00  0.00           H
ATOM    462  HB  ILE A  33       2.860  -7.045   1.605  1.00  0.00           H
ATOM    463 HG11 ILE A  33       4.072  -4.313   2.246  1.00  0.00           H
ATOM    464 HG12 ILE A  33       3.037  -5.291   3.285  1.00  0.00           H
ATOM    465 HG21 ILE A  33       5.068  -6.898   0.462  1.00  0.00           H
ATOM    466 HG22 ILE A  33       4.613  -5.356  -0.276  1.00  0.00           H
ATOM    467 HG23 ILE A  33       3.723  -6.827  -0.699  1.00  0.00           H
ATOM    468 HD11 ILE A  33       4.898  -7.075   3.328  1.00  0.00           H
ATOM    469 HD12 ILE A  33       5.353  -5.510   4.045  1.00  0.00           H
ATOM    470 HD13 ILE A  33       5.932  -5.926   2.433  1.00  0.00           H
ATOM    471  N   ILE A  34       0.326  -6.348   2.165  1.00  0.00           N
ATOM    472  CA  ILE A  34      -0.724  -6.479   3.161  1.00  0.00           C
ATOM    473  C   ILE A  34      -0.215  -7.497   4.185  1.00  0.00           C
ATOM    474  O   ILE A  34       0.159  -8.614   3.826  1.00  0.00           O
ATOM    475  CB  ILE A  34      -2.082  -6.919   2.553  1.00  0.00           C
ATOM    476  CG1 ILE A  34      -2.408  -6.085   1.300  1.00  0.00           C
ATOM    477  CG2 ILE A  34      -3.172  -6.838   3.632  1.00  0.00           C
ATOM    478  CD1 ILE A  34      -3.715  -6.446   0.597  1.00  0.00           C
ATOM    479  H   ILE A  34       0.702  -7.213   1.787  1.00  0.00           H
ATOM    480  HA  ILE A  34      -0.838  -5.494   3.625  1.00  0.00           H
ATOM    481  HB  ILE A  34      -1.973  -7.976   2.248  1.00  0.00           H
ATOM    482 HG11 ILE A  34      -2.414  -5.015   1.568  1.00  0.00           H
ATOM    483 HG12 ILE A  34      -1.584  -6.199   0.578  1.00  0.00           H
ATOM    484 HG21 ILE A  34      -2.889  -7.396   4.538  1.00  0.00           H
ATOM    485 HG22 ILE A  34      -4.125  -7.257   3.278  1.00  0.00           H
ATOM    486 HG23 ILE A  34      -3.350  -5.792   3.934  1.00  0.00           H
ATOM    487 HD11 ILE A  34      -3.725  -7.504   0.281  1.00  0.00           H
ATOM    488 HD12 ILE A  34      -3.838  -5.822  -0.298  1.00  0.00           H
ATOM    489 HD13 ILE A  34      -4.596  -6.283   1.235  1.00  0.00           H
ATOM    490  N   ILE A  35      -0.187  -7.066   5.472  1.00  0.00           N
ATOM    491  CA  ILE A  35       0.406  -7.876   6.511  1.00  0.00           C
ATOM    492  C   ILE A  35      -0.486  -7.896   7.748  1.00  0.00           C
```

```
ATOM    493  O   ILE A  35      -1.280  -6.979   8.016  1.00  0.00           O
ATOM    494  CB  ILE A  35       1.849  -7.408   6.903  1.00  0.00           C
ATOM    495  CG1 ILE A  35       1.857  -6.057   7.644  1.00  0.00           C
ATOM    496  CG2 ILE A  35       2.769  -7.422   5.676  1.00  0.00           C
ATOM    497  CD1 ILE A  35       3.196  -5.713   8.298  1.00  0.00           C
ATOM    498  H   ILE A  35      -0.505  -6.121   5.685  1.00  0.00           H
ATOM    499  HA  ILE A  35       0.482  -8.886   6.084  1.00  0.00           H
ATOM    500  HB  ILE A  35       2.233  -8.173   7.606  1.00  0.00           H
ATOM    501 HG11 ILE A  35       1.572  -5.254   6.943  1.00  0.00           H
ATOM    502 HG12 ILE A  35       1.085  -6.050   8.429  1.00  0.00           H
ATOM    503 HG21 ILE A  35       3.811  -7.220   5.962  1.00  0.00           H
ATOM    504 HG22 ILE A  35       2.474  -6.650   4.951  1.00  0.00           H
ATOM    505 HG23 ILE A  35       2.740  -8.390   5.155  1.00  0.00           H
ATOM    506 HD11 ILE A  35       3.575  -6.556   8.906  1.00  0.00           H
ATOM    507 HD12 ILE A  35       3.095  -4.854   8.972  1.00  0.00           H
ATOM    508 HD13 ILE A  35       3.973  -5.474   7.557  1.00  0.00           H
ATOM    509  N   PRO A  36      -0.298  -8.914   8.627  1.00  0.00           N
ATOM    510  CA  PRO A  36      -0.998  -8.907   9.922  1.00  0.00           C
ATOM    511  C   PRO A  36      -0.332  -8.000  10.966  1.00  0.00           C
ATOM    512  O   PRO A  36      -0.944  -7.655  11.972  1.00  0.00           O
ATOM    513  CB  PRO A  36      -0.967 -10.386  10.353  1.00  0.00           C
ATOM    514  CG  PRO A  36       0.326 -10.914   9.719  1.00  0.00           C
ATOM    515  CD  PRO A  36       0.371 -10.209   8.359  1.00  0.00           C
ATOM    516  HA  PRO A  36      -2.018  -8.525   9.805  1.00  0.00           H
ATOM    517  HB1 PRO A  36      -1.005 -10.492  11.445  1.00  0.00           H
ATOM    518  HB2 PRO A  36      -1.845 -10.903   9.934  1.00  0.00           H
ATOM    519  HG1 PRO A  36       1.196 -10.619  10.326  1.00  0.00           H
ATOM    520  HG2 PRO A  36       0.346 -12.007   9.616  1.00  0.00           H
ATOM    521  HD1 PRO A  36       1.396 -10.067   7.993  1.00  0.00           H
ATOM    522  HD2 PRO A  36      -0.191 -10.767   7.592  1.00  0.00           H
ATOM    523  N   GLY A  37       0.973  -7.662  10.759  1.00  0.00           N
ATOM    524  CA  GLY A  37       1.624  -6.747  11.673  1.00  0.00           C
ATOM    525  C   GLY A  37       0.919  -5.393  11.714  1.00  0.00           C
ATOM    526  O   GLY A  37       0.728  -4.762  10.669  1.00  0.00           O
ATOM    527  H   GLY A  37       1.379  -7.807   9.844  1.00  0.00           H
ATOM    528  HA1 GLY A  37       1.678  -7.193  12.678  1.00  0.00           H
ATOM    529  HA2 GLY A  37       2.646  -6.552  11.321  1.00  0.00           H
ATOM    530  N   ALA A  38       0.586  -4.922  12.935  1.00  0.00           N
ATOM    531  CA  ALA A  38      -0.016  -3.601  13.129  1.00  0.00           C
ATOM    532  C   ALA A  38       0.972  -2.470  12.764  1.00  0.00           C
ATOM    533  O   ALA A  38       0.564  -1.415  12.305  1.00  0.00           O
ATOM    534  CB  ALA A  38      -0.498  -3.445  14.571  1.00  0.00           C
ATOM    535  H   ALA A  38       0.563  -5.587  13.702  1.00  0.00           H
ATOM    536  HA  ALA A  38      -0.866  -3.474  12.443  1.00  0.00           H
ATOM    537  HB1 ALA A  38      -1.276  -4.186  14.809  1.00  0.00           H
ATOM    538  HB2 ALA A  38      -0.926  -2.442  14.704  1.00  0.00           H
ATOM    539  HB3 ALA A  38       0.334  -3.560  15.284  1.00  0.00           H
ATOM    540  N   THR A  39       2.277  -2.730  13.098  1.00  0.00           N
ATOM    541  CA  THR A  39       3.371  -1.925  12.570  1.00  0.00           C
ATOM    542  C   THR A  39       3.934  -2.645  11.333  1.00  0.00           C
ATOM    543  O   THR A  39       3.895  -3.877  11.251  1.00  0.00           O
ATOM    544  CB  THR A  39       4.439  -1.731  13.681  1.00  0.00           C
ATOM    545  OG1 THR A  39       3.816  -1.197  14.836  1.00  0.00           O
ATOM    546  CG2 THR A  39       5.567  -0.789  13.295  1.00  0.00           C
ATOM    547  H   THR A  39       2.514  -3.718  13.162  1.00  0.00           H
ATOM    548  HA  THR A  39       2.951  -0.946  12.300  1.00  0.00           H
ATOM    549  HB  THR A  39       4.864  -2.736  13.899  1.00  0.00           H
ATOM    550  HG1 THR A  39       2.995  -1.701  14.967  1.00  0.00           H
ATOM    551 HG21 THR A  39       6.169  -1.167  12.456  1.00  0.00           H
ATOM    552 HG22 THR A  39       6.229  -0.651  14.162  1.00  0.00           H
ATOM    553 HG23 THR A  39       5.164   0.202  13.028  1.00  0.00           H
ATOM    554  N   CYS A  40       4.444  -1.827  10.390  1.00  0.00           N
ATOM    555  CA  CYS A  40       5.172  -2.275   9.216  1.00  0.00           C
```

```
ATOM    556  C   CYS A  40       6.683  -2.321   9.538  1.00  0.00           C
ATOM    557  O   CYS A  40       7.162  -1.470  10.295  1.00  0.00           O
ATOM    558  CB  CYS A  40       4.959  -1.280   8.067  1.00  0.00           C
ATOM    559  SG  CYS A  40       3.207  -1.024   7.567  1.00  0.00           S
ATOM    560  H   CYS A  40       4.559  -0.849  10.636  1.00  0.00           H
ATOM    561  HA  CYS A  40       4.785  -3.264   8.948  1.00  0.00           H
ATOM    562  HB1 CYS A  40       5.303  -0.279   8.377  1.00  0.00           H
ATOM    563  HB2 CYS A  40       5.552  -1.535   7.181  1.00  0.00           H
ATOM    564  N   PRO A  41       7.432  -3.276   8.943  1.00  0.00           N
ATOM    565  CA  PRO A  41       8.892  -3.223   9.066  1.00  0.00           C
ATOM    566  C   PRO A  41       9.392  -1.983   8.298  1.00  0.00           C
ATOM    567  O   PRO A  41       8.691  -1.455   7.431  1.00  0.00           O
ATOM    568  CB  PRO A  41       9.351  -4.569   8.478  1.00  0.00           C
ATOM    569  CG  PRO A  41       8.296  -4.872   7.405  1.00  0.00           C
ATOM    570  CD  PRO A  41       6.989  -4.262   7.937  1.00  0.00           C
ATOM    571  HA  PRO A  41       9.165  -3.107  10.125  1.00  0.00           H
ATOM    572  HB1 PRO A  41      10.351  -4.511   8.023  1.00  0.00           H
ATOM    573  HB2 PRO A  41       9.340  -5.323   9.283  1.00  0.00           H
ATOM    574  HG1 PRO A  41       8.613  -4.422   6.454  1.00  0.00           H
ATOM    575  HG2 PRO A  41       8.194  -5.950   7.215  1.00  0.00           H
ATOM    576  HD1 PRO A  41       6.409  -3.777   7.137  1.00  0.00           H
ATOM    577  HD2 PRO A  41       6.345  -5.013   8.423  1.00  0.00           H
ATOM    578  N   GLY A  42      10.598  -1.484   8.650  1.00  0.00           N
ATOM    579  CA  GLY A  42      11.088  -0.201   8.155  1.00  0.00           C
ATOM    580  C   GLY A  42      11.163  -0.050   6.621  1.00  0.00           C
ATOM    581  O   GLY A  42      10.791   1.002   6.088  1.00  0.00           O
ATOM    582  H   GLY A  42      11.166  -2.005   9.306  1.00  0.00           H
ATOM    583  HA1 GLY A  42      10.447   0.615   8.512  1.00  0.00           H
ATOM    584  HA2 GLY A  42      12.094  -0.052   8.573  1.00  0.00           H
ATOM    585  N   ASP A  43      11.659  -1.118   5.977  1.00  0.00           N
ATOM    586  CA  ASP A  43      11.635  -1.319   4.528  1.00  0.00           C
ATOM    587  C   ASP A  43      10.282  -0.999   3.862  1.00  0.00           C
ATOM    588  O   ASP A  43      10.216  -0.620   2.695  1.00  0.00           O
ATOM    589  CB  ASP A  43      11.973  -2.815   4.262  1.00  0.00           C
ATOM    590  CG  ASP A  43      11.174  -3.755   5.211  1.00  0.00           C
ATOM    591  OD2 ASP A  43      11.526  -3.683   6.440  1.00  0.00           O
ATOM    592  OD1 ASP A  43      10.247  -4.446   4.743  1.00  0.00           O
ATOM    593  H   ASP A  43      11.768  -2.030   6.485  1.00  0.00           H
ATOM    594  HA  ASP A  43      12.365  -0.666   4.028  1.00  0.00           H
ATOM    595  HB1 ASP A  43      11.760  -3.038   3.209  1.00  0.00           H
ATOM    596  HB2 ASP A  43      13.049  -2.965   4.444  1.00  0.00           H
ATOM    597  N   TYR A  44       9.214  -1.245   4.652  1.00  0.00           N
ATOM    598  CA  TYR A  44       7.839  -1.181   4.240  1.00  0.00           C
ATOM    599  C   TYR A  44       7.037  -0.202   5.110  1.00  0.00           C
ATOM    600  O   TYR A  44       5.804  -0.302   5.224  1.00  0.00           O
ATOM    601  CB  TYR A  44       7.226  -2.603   4.173  1.00  0.00           C
ATOM    602  CG  TYR A  44       7.249  -3.155   2.771  1.00  0.00           C
ATOM    603  CD2 TYR A  44       8.321  -3.910   2.271  1.00  0.00           C
ATOM    604  CD1 TYR A  44       6.196  -2.853   1.904  1.00  0.00           C
ATOM    605  CE2 TYR A  44       8.330  -4.337   0.942  1.00  0.00           C
ATOM    606  CE1 TYR A  44       6.181  -3.298   0.587  1.00  0.00           C
ATOM    607  CZ  TYR A  44       7.266  -4.029   0.082  1.00  0.00           C
ATOM    608  OH  TYR A  44       7.320  -4.456  -1.208  1.00  0.00           O
ATOM    609  H   TYR A  44       9.368  -1.572   5.603  1.00  0.00           H
ATOM    610  HA  TYR A  44       7.823  -0.734   3.236  1.00  0.00           H
ATOM    611  HB1 TYR A  44       7.798  -3.250   4.851  1.00  0.00           H
ATOM    612  HB2 TYR A  44       6.194  -2.564   4.538  1.00  0.00           H
ATOM    613  HD2 TYR A  44       9.149  -4.167   2.939  1.00  0.00           H
ATOM    614  HD1 TYR A  44       5.352  -2.270   2.267  1.00  0.00           H
ATOM    615  HE2 TYR A  44       9.168  -4.918   0.550  1.00  0.00           H
ATOM    616  HE1 TYR A  44       5.330  -3.100  -0.058  1.00  0.00           H
ATOM    617  HH  TYR A  44       6.545  -4.108  -1.702  1.00  0.00           H
ATOM    618  N   ALA A  45       7.738   0.805   5.668  1.00  0.00           N
```

```
ATOM    619  CA  ALA A  45       7.139   1.767   6.580  1.00  0.00           C
ATOM    620  C   ALA A  45       6.936   3.131   5.902  1.00  0.00           C
ATOM    621  O   ALA A  45       7.095   4.202   6.511  1.00  0.00           O
ATOM    622  CB  ALA A  45       7.905   1.873   7.893  1.00  0.00           C
ATOM    623  H   ALA A  45       8.759   0.799   5.597  1.00  0.00           H
ATOM    624  HA  ALA A  45       6.113   1.403   6.759  1.00  0.00           H
ATOM    625  HB1 ALA A  45       7.906   0.901   8.405  1.00  0.00           H
ATOM    626  HB2 ALA A  45       7.438   2.630   8.537  1.00  0.00           H
ATOM    627  HB3 ALA A  45       8.945   2.179   7.705  1.00  0.00           H
ATOM    628  N   ASN A  46       6.518   3.078   4.623  1.00  0.00           N
ATOM    629  CA  ASN A  46       5.803   4.167   3.994  1.00  0.00           C
ATOM    630  C   ASN A  46       4.359   3.720   3.637  1.00  0.00           C
ATOM    631  OXT ASN A  46       3.955   2.618   4.107  1.00  0.00           O
ATOM    632  CB  ASN A  46       6.552   4.761   2.795  1.00  0.00           C
ATOM    633  CG  ASN A  46       7.847   5.520   3.063  1.00  0.00           C
ATOM    634  OD1 ASN A  46       8.496   6.006   2.111  1.00  0.00           O
ATOM    635  ND2 ASN A  46       8.252   5.688   4.330  1.00  0.00           N
ATOM    636  O   ASN A  46       3.682   4.523   2.939  1.00  0.00           O
ATOM    637  H   ASN A  46       6.406   2.173   4.170  1.00  0.00           H
ATOM    638  HA  ASN A  46       5.671   4.949   4.759  1.00  0.00           H
ATOM    639  HB1 ASN A  46       6.799   3.954   2.089  1.00  0.00           H
ATOM    640  HB2 ASN A  46       5.875   5.451   2.271  1.00  0.00           H
ATOM    641 HD21 ASN A  46       7.812   5.253   5.154  1.00  0.00           H
ATOM    642 HD22 ASN A  46       9.139   6.166   4.455  1.00  0.00           H
END
```

2) CRAMBIN – molecule as in the optimized 0W crystal

```
COMPND    CRAMBIN OPTIMIZED CRYSTAL 0W GEOMETRY
AUTHOR    DELLE PIANE, CORNO, ORLANDO, DOVESI, UGLIENGO
ATOM      1  N   THR N   1      -7.130  -5.367  -3.415  1.00  0.00           N
ATOM      2  H1  THR N   1      -7.808  -5.844  -4.016  1.00  0.00           H
ATOM      3  H2  THR N   1      -7.455  -5.409  -2.434  1.00  0.00           H
ATOM      4  H3  THR N   1      -6.249  -5.927  -3.476  1.00  0.00           H
ATOM      5  CA  THR N   1      -6.943  -3.934  -3.779  1.00  0.00           C
ATOM      6  HA  THR N   1      -7.907  -3.544  -4.134  1.00  0.00           H
ATOM      7  C   THR N   1      -5.859  -3.842  -4.847  1.00  0.00           C
ATOM      8  CB  THR N   1      -6.480  -3.169  -2.492  1.00  0.00           C
ATOM      9  O   THR N   1      -4.905  -4.641  -4.820  1.00  0.00           O
ATOM     10  N   THR N   2      -5.980  -2.809  -5.680  1.00  0.00           N
ATOM     11  HB  THR N   1      -5.416  -3.420  -2.342  1.00  0.00           H
ATOM     12  OG1 THR N   1      -7.240  -3.648  -1.392  1.00  0.00           O
ATOM     13  CG2 THR N   1      -6.639  -1.665  -2.642  1.00  0.00           C
ATOM     14  H   THR N   2      -6.887  -2.331  -5.781  1.00  0.00           H
ATOM     15  CA  THR N   2      -4.922  -2.459  -6.603  1.00  0.00           C
ATOM     16  HG1 THR N   1      -6.662  -4.306  -0.939  1.00  0.00           H
ATOM     17 HG21 THR N   1      -6.323  -1.179  -1.708  1.00  0.00           H
ATOM     18 HG22 THR N   1      -6.018  -1.275  -3.461  1.00  0.00           H
ATOM     19 HG23 THR N   1      -7.693  -1.400  -2.825  1.00  0.00           H
ATOM     20  HA  THR N   2      -4.511  -3.379  -7.022  1.00  0.00           H
ATOM     21  C   THR N   2      -3.775  -1.719  -5.910  1.00  0.00           C
ATOM     22  CB  THR N   2      -5.496  -1.627  -7.782  1.00  0.00           C
ATOM     23  O   THR N   2      -3.948  -0.892  -5.009  1.00  0.00           O
ATOM     24  N   CYS N   3      -2.574  -2.037  -6.444  1.00  0.00           N
ATOM     25  HB  THR N   2      -4.637  -1.175  -8.308  1.00  0.00           H
ATOM     26  OG1 THR N   2      -6.278  -0.558  -7.265  1.00  0.00           O
ATOM     27  CG2 THR N   2      -6.217  -2.562  -8.755  1.00  0.00           C
ATOM     28  H   CYS N   3      -2.562  -2.704  -7.215  1.00  0.00           H
```

```
ATOM     29  CA  CYS N   3      -1.309  -1.392  -6.145  1.00  0.00           C
ATOM     30  HG1 THR N   2      -7.238  -0.655  -7.511  1.00  0.00           H
ATOM     31 HG21 THR N   2      -6.835  -2.012  -9.466  1.00  0.00           H
ATOM     32 HG22 THR N   2      -5.471  -3.141  -9.310  1.00  0.00           H
ATOM     33 HG23 THR N   2      -6.866  -3.258  -8.211  1.00  0.00           H
ATOM     34  HA  CYS N   3      -1.504  -0.436  -5.645  1.00  0.00           H
ATOM     35  C   CYS N   3      -0.600  -1.155  -7.488  1.00  0.00           C
ATOM     36  CB  CYS N   3      -0.392  -2.240  -5.247  1.00  0.00           C
ATOM     37  O   CYS N   3      -0.759  -1.963  -8.421  1.00  0.00           O
ATOM     38  N   CYS N   4       0.201  -0.082  -7.559  1.00  0.00           N
ATOM     39  HB1 CYS N   3      -0.198  -3.216  -5.713  1.00  0.00           H
ATOM     40  HB2 CYS N   3       0.582  -1.746  -5.106  1.00  0.00           H
ATOM     41  SG  CYS N   3      -1.101  -2.668  -3.608  1.00  0.00           S
ATOM     42  H   CYS N   4       0.186   0.617  -6.791  1.00  0.00           H
ATOM     43  CA  CYS N   4       0.708   0.360  -8.843  1.00  0.00           C
ATOM     44  SG  CYS N  40      -0.971  -0.887  -2.536  1.00  0.00           S
ATOM     45  HA  CYS N   4       0.448  -0.422  -9.561  1.00  0.00           H
ATOM     46  C   CYS N   4       2.226   0.537  -8.829  1.00  0.00           C
ATOM     47  CB  CYS N   4       0.031   1.676  -9.259  1.00  0.00           C
ATOM     48  CB  CYS N  40       0.807  -0.801  -2.025  1.00  0.00           C
ATOM     49  O   CYS N   4       2.817   1.042  -7.866  1.00  0.00           O
ATOM     50  N   PRO N   5       2.882   0.167  -9.952  1.00  0.00           N
ATOM     51  HB1 CYS N   4      -0.017   2.359  -8.399  1.00  0.00           H
ATOM     52  HB2 CYS N   4       0.574   2.187 -10.065  1.00  0.00           H
ATOM     53  SG  CYS N   4      -1.708   1.443  -9.792  1.00  0.00           S
ATOM     54  HB1 CYS N  40       1.412  -0.460  -2.877  1.00  0.00           H
ATOM     55  HB2 CYS N  40       0.827  -0.014  -1.256  1.00  0.00           H
ATOM     56  CA  CYS N  40       1.374  -2.122  -1.511  1.00  0.00           C
ATOM     57  CA  PRO N   5       4.354   0.202 -10.031  1.00  0.00           C
ATOM     58  CD  PRO N   5       2.271  -0.355 -11.196  1.00  0.00           C
ATOM     59  SG  CYS N  32      -1.453   0.620 -11.699  1.00  0.00           S
ATOM     60  HA  CYS N  40       1.253  -2.866  -2.301  1.00  0.00           H
ATOM     61  N   CYS N  40       0.669  -2.650  -0.346  1.00  0.00           N
ATOM     62  C   CYS N  40       2.857  -1.973  -1.129  1.00  0.00           C
ATOM     63  HA  PRO N   5       4.815  -0.332  -9.190  1.00  0.00           H
ATOM     64  C   PRO N   5       4.950   1.602  -9.973  1.00  0.00           C
ATOM     65  CB  PRO N   5       4.653  -0.496 -11.378  1.00  0.00           C
ATOM     66  HD1 PRO N   5       1.381   0.216 -11.481  1.00  0.00           H
ATOM     67  HD2 PRO N   5       1.990  -1.408 -11.067  1.00  0.00           H
ATOM     68  CG  PRO N   5       3.395  -0.230 -12.208  1.00  0.00           C
ATOM     69  CB  CYS N  32      -1.841  -1.187 -11.524  1.00  0.00           C
ATOM     70  H   CYS N  40       0.318  -2.006   0.355  1.00  0.00           H
ATOM     71  C   THR N  39       0.652  -3.985  -0.104  1.00  0.00           C
ATOM     72  O   CYS N  40       3.151  -1.162  -0.228  1.00  0.00           O
ATOM     73  N   PRO N  41       3.753  -2.778  -1.730  1.00  0.00           N
ATOM     74  O   PRO N   5       6.163   1.751  -9.762  1.00  0.00           O
ATOM     75  N   SER N   6       4.143   2.647 -10.207  1.00  0.00           N
ATOM     76  HB1 PRO N   5       5.567  -0.120 -11.851  1.00  0.00           H
ATOM     77  HB2 PRO N   5       4.783  -1.573 -11.197  1.00  0.00           H
ATOM     78  HG1 PRO N   5       3.281  -0.902 -13.061  1.00  0.00           H
ATOM     79  HG2 PRO N   5       3.417   0.789 -12.609  1.00  0.00           H
ATOM     80  HB1 CYS N  32      -2.260  -1.321 -10.520  1.00  0.00           H
ATOM     81  HB2 CYS N  32      -2.649  -1.360 -12.245  1.00  0.00           H
ATOM     82  CA  CYS N  32      -0.689  -2.187 -11.755  1.00  0.00           C
ATOM     83  O   THR N  39       1.141  -4.805  -0.882  1.00  0.00           O
ATOM     84  CA  THR N  39      -0.068  -4.441   1.179  1.00  0.00           C
ATOM     85  CA  PRO N  41       5.178  -2.750  -1.405  1.00  0.00           C
ATOM     86  CD  PRO N  41       3.530  -3.848  -2.724  1.00  0.00           C
ATOM     87  H   SER N   6       3.138   2.529 -10.271  1.00  0.00           H
ATOM     88  CA  SER N   6       4.631   4.003 -10.107  1.00  0.00           C
ATOM     89  HA  CYS N  32       0.069  -2.035 -10.976  1.00  0.00           H
ATOM     90  N   CYS N  32      -0.075  -2.031 -13.053  1.00  0.00           N
ATOM     91  C   CYS N  32      -1.152  -3.666 -11.558  1.00  0.00           C
```

ATOM	92	HA	THR	N	39	-0.037	-3.660	1.951	1.00	0.00	H
ATOM	93	N	THR	N	39	-1.480	-4.619	0.851	1.00	0.00	N
ATOM	94	CB	THR	N	39	0.581	-5.736	1.730	1.00	0.00	C
ATOM	95	HA	PRO	N	41	5.298	-2.363	-0.391	1.00	0.00	H
ATOM	96	C	PRO	N	41	5.918	-1.796	-2.355	1.00	0.00	C
ATOM	97	CB	PRO	N	41	5.638	-4.227	-1.546	1.00	0.00	C
ATOM	98	HD1	PRO	N	41	2.489	-4.173	-2.725	1.00	0.00	H
ATOM	99	HD2	PRO	N	41	3.809	-3.460	-3.712	1.00	0.00	H
ATOM	100	CG	PRO	N	41	4.472	-4.957	-2.251	1.00	0.00	C
ATOM	101	HA	SER	N	6	5.304	4.071	-9.239	1.00	0.00	H
ATOM	102	C	SER	N	6	3.436	4.920	-9.895	1.00	0.00	C
ATOM	103	CB	SER	N	6	5.383	4.440	-11.368	1.00	0.00	C
ATOM	104	H	CYS	N	32	-0.671	-1.687	-13.806	1.00	0.00	H
ATOM	105	C	GLY	N	31	1.054	-2.707	-13.384	1.00	0.00	C
ATOM	106	O	CYS	N	32	-0.995	-4.508	-12.449	1.00	0.00	O
ATOM	107	N	ILE	N	33	-1.688	-3.928	-10.350	1.00	0.00	N
ATOM	108	H	THR	N	39	-1.712	-5.288	0.122	1.00	0.00	H
ATOM	109	C	ALA	N	38	-2.480	-3.890	1.404	1.00	0.00	C
ATOM	110	HB	THR	N	39	0.612	-6.460	0.892	1.00	0.00	H
ATOM	111	OG1	THR	N	39	-0.133	-6.261	2.823	1.00	0.00	O
ATOM	112	CG2	THR	N	39	2.004	-5.482	2.217	1.00	0.00	C
ATOM	113	O	PRO	N	41	5.354	-1.256	-3.298	1.00	0.00	O
ATOM	114	N	GLY	N	42	7.249	-1.605	-2.120	1.00	0.00	N
ATOM	115	HB1	PRO	N	41	6.552	-4.297	-2.147	1.00	0.00	H
ATOM	116	HB2	PRO	N	41	5.842	-4.656	-0.554	1.00	0.00	H
ATOM	117	HG1	PRO	N	41	4.840	-5.545	-3.100	1.00	0.00	H
ATOM	118	HG2	PRO	N	41	3.935	-5.607	-1.544	1.00	0.00	H
ATOM	119	O	SER	N	6	2.271	4.543	-10.077	1.00	0.00	O
ATOM	120	N	ILE	N	7	3.757	6.199	-9.604	1.00	0.00	N
ATOM	121	HB1	SER	N	6	6.149	3.691	-11.614	1.00	0.00	H
ATOM	122	HB2	SER	N	6	5.907	5.392	-11.172	1.00	0.00	H
ATOM	123	OG	SER	N	6	4.451	4.590	-12.428	1.00	0.00	O
ATOM	124	O	GLY	N	31	1.793	-3.244	-12.553	1.00	0.00	O
ATOM	125	CA	GLY	N	31	1.418	-2.786	-14.874	1.00	0.00	C
ATOM	126	H	ILE	N	33	-1.631	-3.194	-9.641	1.00	0.00	H
ATOM	127	CA	ILE	N	33	-1.881	-5.273	-9.835	1.00	0.00	C
ATOM	128	O	ALA	N	38	-2.290	-3.044	2.280	1.00	0.00	O
ATOM	129	CA	ALA	N	38	-3.888	-4.156	0.811	1.00	0.00	C
ATOM	130	HG1	THR	N	39	-1.105	-6.218	2.685	1.00	0.00	H
ATOM	131	HG21	THR	N	39	2.653	-5.123	1.405	1.00	0.00	H
ATOM	132	HG22	THR	N	39	2.424	-6.419	2.611	1.00	0.00	H
ATOM	133	HG23	THR	N	39	1.995	-4.755	3.046	1.00	0.00	H
ATOM	134	H	GLY	N	42	7.625	-1.833	-1.205	1.00	0.00	H
ATOM	135	CA	GLY	N	42	7.982	-0.608	-2.887	1.00	0.00	C
ATOM	136	H	ILE	N	7	4.673	6.357	-9.198	1.00	0.00	H
ATOM	137	CA	ILE	N	7	2.731	7.196	-9.351	1.00	0.00	C
ATOM	138	H	SER	N	6	4.889	5.157	-13.109	1.00	0.00	H
ATOM	139	HA1	GLY	N	31	1.443	-3.857	-15.121	1.00	0.00	H
ATOM	140	HA2	GLY	N	31	2.435	-2.384	-14.982	1.00	0.00	H
ATOM	141	N	GLY	N	31	0.529	-2.105	-15.786	1.00	0.00	N
ATOM	142	HA	ILE	N	33	-1.930	-5.968	-10.679	1.00	0.00	H
ATOM	143	C	ILE	N	33	-3.183	-5.283	-9.061	1.00	0.00	C
ATOM	144	CB	ILE	N	33	-0.685	-5.738	-8.913	1.00	0.00	C
ATOM	145	HA	ALA	N	38	-3.829	-3.921	-0.265	1.00	0.00	H
ATOM	146	N	ALA	N	38	-4.211	-5.581	0.930	1.00	0.00	N
ATOM	147	CB	ALA	N	38	-4.948	-3.268	1.461	1.00	0.00	C
ATOM	148	HA1	GLY	N	42	8.991	-0.532	-2.460	1.00	0.00	H
ATOM	149	HA2	GLY	N	42	7.517	0.387	-2.803	1.00	0.00	H
ATOM	150	C	GLY	N	42	8.113	-0.876	-4.392	1.00	0.00	C
ATOM	151	HA	ILE	N	7	1.958	6.765	-8.697	1.00	0.00	H
ATOM	152	C	ILE	N	7	1.972	7.532	-10.645	1.00	0.00	C
ATOM	153	CB	ILE	N	7	3.348	8.430	-8.646	1.00	0.00	C
ATOM	154	H	GLY	N	31	-0.318	-2.594	-16.068	1.00	0.00	H

ATOM	155	C	THR	N	30	0.613	-0.760	-15.949	1.00	0.00	C
ATOM	156	O	ILE	N	33	-3.627	-4.232	-8.594	1.00	0.00	O
ATOM	157	N	ILE	N	34	-3.785	-6.485	-8.831	1.00	0.00	N
ATOM	158	HB	ILE	N	33	-0.644	-6.837	-9.014	1.00	0.00	H
ATOM	159	CG2	ILE	N	33	0.616	-5.147	-9.456	1.00	0.00	C
ATOM	160	CG1	ILE	N	33	-0.889	-5.388	-7.422	1.00	0.00	C
ATOM	161	H	ALA	N	38	-3.827	-6.047	1.775	1.00	0.00	H
ATOM	162	C	GLY	N	37	-5.087	-6.214	0.137	1.00	0.00	C
ATOM	163	HB1	ALA	N	38	-5.903	-3.360	0.929	1.00	0.00	H
ATOM	164	HB2	ALA	N	38	-4.634	-2.216	1.435	1.00	0.00	H
ATOM	165	HB3	ALA	N	38	-5.097	-3.557	2.510	1.00	0.00	H
ATOM	166	O	GLY	N	42	8.383	0.061	-5.149	1.00	0.00	O
ATOM	167	N	ASP	N	43	7.967	-2.155	-4.800	1.00	0.00	N
ATOM	168	O	ILE	N	7	0.745	7.671	-10.617	1.00	0.00	O
ATOM	169	N	VAL	N	8	2.697	7.684	-11.780	1.00	0.00	N
ATOM	170	HB	ILE	N	7	4.268	8.706	-9.198	1.00	0.00	H
ATOM	171	CG2	ILE	N	7	2.390	9.608	-8.723	1.00	0.00	C
ATOM	172	CG1	ILE	N	7	3.724	8.058	-7.194	1.00	0.00	C
ATOM	173	O	THR	N	30	1.553	-0.091	-15.517	1.00	0.00	O
ATOM	174	CA	THR	N	30	-0.585	-0.046	-16.594	1.00	0.00	C
ATOM	175	H	ILE	N	34	-3.277	-7.325	-9.101	1.00	0.00	H
ATOM	176	CA	ILE	N	34	-4.654	-6.626	-7.659	1.00	0.00	C
ATOM	177	HG21	ILE	N	33	1.478	-5.520	-8.906	1.00	0.00	H
ATOM	178	HG22	ILE	N	33	0.624	-4.057	-9.342	1.00	0.00	H
ATOM	179	HG23	ILE	N	33	0.775	-5.380	-10.517	1.00	0.00	H
ATOM	180	HG11	ILE	N	33	-1.048	-4.304	-7.340	1.00	0.00	H
ATOM	181	HG12	ILE	N	33	-1.810	-5.861	-7.047	1.00	0.00	H
ATOM	182	CD1	ILE	N	33	0.257	-5.796	-6.492	1.00	0.00	C
ATOM	183	O	GLY	N	37	-5.619	-5.695	-0.864	1.00	0.00	O
ATOM	184	CA	GLY	N	37	-5.454	-7.640	0.563	1.00	0.00	C
ATOM	185	H	ASP	N	43	7.497	-2.843	-4.202	1.00	0.00	H
ATOM	186	CA	ASP	N	43	7.834	-2.479	-6.217	1.00	0.00	C
ATOM	187	H	VAL	N	8	3.676	7.407	-11.772	1.00	0.00	H
ATOM	188	CA	VAL	N	8	2.025	7.902	-13.061	1.00	0.00	C
ATOM	189	HG21	ILE	N	7	2.784	10.462	-8.167	1.00	0.00	H
ATOM	190	HG22	ILE	N	7	2.240	9.935	-9.760	1.00	0.00	H
ATOM	191	HG23	ILE	N	7	1.405	9.344	-8.311	1.00	0.00	H
ATOM	192	HG11	ILE	N	7	4.412	7.200	-7.209	1.00	0.00	H
ATOM	193	HG12	ILE	N	7	2.810	7.688	-6.696	1.00	0.00	H
ATOM	194	CD1	ILE	N	7	4.347	9.162	-6.341	1.00	0.00	C
ATOM	195	HA	THR	N	30	-0.194	0.578	-17.409	1.00	0.00	H
ATOM	196	N	THR	N	30	-1.623	-0.895	-17.154	1.00	0.00	N
ATOM	197	CB	THR	N	30	-1.198	0.852	-15.491	1.00	0.00	C
ATOM	198	HA	ILE	N	34	-4.611	-5.665	-7.134	1.00	0.00	H
ATOM	199	C	ILE	N	34	-4.056	-7.736	-6.798	1.00	0.00	C
ATOM	200	CB	ILE	N	34	-6.152	-6.983	-7.917	1.00	0.00	C
ATOM	201	HD11	ILE	N	33	0.479	-6.871	-6.564	1.00	0.00	H
ATOM	202	HD12	ILE	N	33	1.188	-5.254	-6.714	1.00	0.00	H
ATOM	203	HD13	ILE	N	33	-0.004	-5.589	-5.442	1.00	0.00	H
ATOM	204	HA1	GLY	N	37	-4.762	-8.010	1.329	1.00	0.00	H
ATOM	205	HA2	GLY	N	37	-6.467	-7.590	0.988	1.00	0.00	H
ATOM	206	N	GLY	N	37	-5.479	-8.554	-0.552	1.00	0.00	N
ATOM	207	HA	ASP	N	43	8.654	-1.992	-6.752	1.00	0.00	H
ATOM	208	CB	ASP	N	43	7.921	-4.003	-6.415	1.00	0.00	C
ATOM	209	C	ASP	N	43	6.546	-1.863	-6.812	1.00	0.00	C
ATOM	210	HA	VAL	N	8	1.228	8.637	-12.881	1.00	0.00	H
ATOM	211	C	VAL	N	8	1.268	6.651	-13.520	1.00	0.00	C
ATOM	212	CB	VAL	N	8	2.985	8.471	-14.134	1.00	0.00	C
ATOM	213	HD11	ILE	N	7	5.286	9.557	-6.753	1.00	0.00	H
ATOM	214	HD12	ILE	N	7	3.668	10.015	-6.194	1.00	0.00	H
ATOM	215	HD13	ILE	N	7	4.586	8.770	-5.340	1.00	0.00	H
ATOM	216	H	THR	N	30	-2.154	-1.487	-16.517	1.00	0.00	H
ATOM	217	C	TYR	N	29	-2.127	-0.721	-18.403	1.00	0.00	C

ATOM	218	HB	THR	N	30	-0.368	1.395	-15.022	1.00	0.00	H
ATOM	219	OG1	THR	N	30	-1.745	0.009	-14.470	1.00	0.00	O
ATOM	220	CG2	THR	N	30	-2.224	1.840	-16.020	1.00	0.00	C
ATOM	221	O	ILE	N	34	-3.739	-8.824	-7.301	1.00	0.00	O
ATOM	222	N	ILE	N	35	-3.943	-7.452	-5.487	1.00	0.00	N
ATOM	223	HB	ILE	N	34	-6.215	-8.078	-7.998	1.00	0.00	H
ATOM	224	CG1	ILE	N	34	-6.710	-6.420	-9.230	1.00	0.00	C
ATOM	225	CG2	ILE	N	34	-7.004	-6.561	-6.708	1.00	0.00	C
ATOM	226	H	GLY	N	37	-4.581	-8.979	-0.785	1.00	0.00	H
ATOM	227	C	PRO	N	36	-6.613	-9.091	-1.054	1.00	0.00	C
ATOM	228	HB1	ASP	N	43	7.753	-4.246	-7.475	1.00	0.00	H
ATOM	229	HB2	ASP	N	43	8.949	-4.324	-6.179	1.00	0.00	H
ATOM	230	CG	ASP	N	43	6.973	-4.902	-5.565	1.00	0.00	C
ATOM	231	O	ASP	N	43	6.593	-1.032	-7.742	1.00	0.00	O
ATOM	232	N	TYR	N	44	5.403	-2.274	-6.247	1.00	0.00	N
ATOM	233	O	VAL	N	8	0.183	6.784	-14.100	1.00	0.00	O
ATOM	234	N	ALA	N	9	1.784	5.436	-13.248	1.00	0.00	N
ATOM	235	HB	VAL	N	8	3.902	7.856	-14.120	1.00	0.00	H
ATOM	236	CG2	VAL	N	8	3.359	9.912	-13.752	1.00	0.00	C
ATOM	237	CG1	VAL	N	8	2.380	8.390	-15.544	1.00	0.00	C
ATOM	238	O	TYR	N	29	-1.618	0.007	-19.257	1.00	0.00	O
ATOM	239	CA	TYR	N	29	-3.448	-1.446	-18.711	1.00	0.00	C
ATOM	240	HG1	THR	N	30	-2.654	-0.276	-14.706	1.00	0.00	H
ATOM	241	HG21	THR	N	30	-2.437	2.594	-15.258	1.00	0.00	H
ATOM	242	HG22	THR	N	30	-3.157	1.325	-16.284	1.00	0.00	H
ATOM	243	HG23	THR	N	30	-1.855	2.352	-16.921	1.00	0.00	H
ATOM	244	H	ILE	N	35	-4.268	-6.536	-5.166	1.00	0.00	H
ATOM	245	CA	ILE	N	35	-3.725	-8.490	-4.497	1.00	0.00	C
ATOM	246	HG11	ILE	N	34	-6.697	-5.319	-9.199	1.00	0.00	H
ATOM	247	HG12	ILE	N	34	-6.041	-6.697	-10.062	1.00	0.00	H
ATOM	248	CD1	ILE	N	34	-8.119	-6.939	-9.515	1.00	0.00	C
ATOM	249	HG21	ILE	N	34	-6.560	-6.914	-5.769	1.00	0.00	H
ATOM	250	HG22	ILE	N	34	-8.010	-7.001	-6.769	1.00	0.00	H
ATOM	251	HG23	ILE	N	34	-7.123	-5.466	-6.665	1.00	0.00	H
ATOM	252	O	PRO	N	36	-7.740	-8.902	-0.591	1.00	0.00	O
ATOM	253	CA	PRO	N	36	-6.464	-9.999	-2.293	1.00	0.00	C
ATOM	254	OD1	ASP	N	43	6.939	-6.102	-5.866	1.00	0.00	O
ATOM	255	OD2	ASP	N	43	6.317	-4.313	-4.627	1.00	0.00	O
ATOM	256	H	TYR	N	44	5.502	-2.973	-5.487	1.00	0.00	H
ATOM	257	CA	TYR	N	44	4.091	-1.789	-6.645	1.00	0.00	C
ATOM	258	H	ALA	N	9	2.687	5.311	-12.782	1.00	0.00	H
ATOM	259	CA	ALA	N	9	0.967	4.261	-13.525	1.00	0.00	C
ATOM	260	HG21	VAL	N	8	4.089	10.326	-14.463	1.00	0.00	H
ATOM	261	HG22	VAL	N	8	3.799	9.953	-12.744	1.00	0.00	H
ATOM	262	HG23	VAL	N	8	2.476	10.572	-13.762	1.00	0.00	H
ATOM	263	HG11	VAL	N	8	3.027	8.896	-16.273	1.00	0.00	H
ATOM	264	HG12	VAL	N	8	2.258	7.348	-15.878	1.00	0.00	H
ATOM	265	HG13	VAL	N	8	1.384	8.855	-15.603	1.00	0.00	H
ATOM	266	HA	TYR	N	29	-3.402	-1.658	-19.791	1.00	0.00	H
ATOM	267	N	TYR	N	29	-3.604	-2.682	-17.958	1.00	0.00	N
ATOM	268	CB	TYR	N	29	-4.659	-0.533	-18.387	1.00	0.00	C
ATOM	269	HA	ILE	N	35	-3.566	-9.414	-5.069	1.00	0.00	H
ATOM	270	C	ILE	N	35	-5.006	-8.636	-3.676	1.00	0.00	C
ATOM	271	CB	ILE	N	35	-2.471	-8.233	-3.613	1.00	0.00	C
ATOM	272	HD11	ILE	N	34	-8.501	-6.545	-10.460	1.00	0.00	H
ATOM	273	HD12	ILE	N	34	-8.837	-6.643	-8.737	1.00	0.00	H
ATOM	274	HD13	ILE	N	34	-8.131	-8.039	-9.569	1.00	0.00	H
ATOM	275	HA	PRO	N	36	-7.313	-9.698	-2.922	1.00	0.00	H
ATOM	276	N	PRO	N	36	-5.207	-9.807	-3.032	1.00	0.00	N
ATOM	277	CB	PRO	N	36	-6.496	-11.520	-2.020	1.00	0.00	C
ATOM	278	HA	TYR	N	44	4.240	-1.231	-7.576	1.00	0.00	H
ATOM	279	CB	TYR	N	44	3.125	-2.947	-6.927	1.00	0.00	C
ATOM	280	C	TYR	N	44	3.602	-0.781	-5.593	1.00	0.00	C

ATOM	281	HA	ALA	N	9	0.712	4.248	-14.594	1.00	0.00	H
ATOM	282	CB	ALA	N	9	1.682	2.965	-13.164	1.00	0.00	C
ATOM	283	C	ALA	N	9	-0.375	4.378	-12.794	1.00	0.00	C
ATOM	284	H	TYR	N	29	-4.521	-2.885	-17.555	1.00	0.00	H
ATOM	285	C	THR	N	28	-2.708	-3.696	-18.077	1.00	0.00	C
ATOM	286	HB1	TYR	N	29	-4.605	-0.268	-17.322	1.00	0.00	H
ATOM	287	HB2	TYR	N	29	-5.562	-1.151	-18.496	1.00	0.00	H
ATOM	288	CG	TYR	N	29	-4.809	0.719	-19.218	1.00	0.00	C
ATOM	289	O	ILE	N	35	-5.836	-7.718	-3.588	1.00	0.00	O
ATOM	290	HB	ILE	N	35	-2.556	-8.891	-2.730	1.00	0.00	H
ATOM	291	CG2	ILE	N	35	-1.217	-8.639	-4.397	1.00	0.00	C
ATOM	292	CG1	ILE	N	35	-2.438	-6.780	-3.102	1.00	0.00	C
ATOM	293	CD	PRO	N	36	-4.443	-11.058	-3.213	1.00	0.00	C
ATOM	294	HB1	PRO	N	36	-6.946	-11.757	-1.047	1.00	0.00	H
ATOM	295	HB2	PRO	N	36	-7.121	-12.007	-2.784	1.00	0.00	H
ATOM	296	CG	PRO	N	36	-5.037	-11.985	-2.151	1.00	0.00	C
ATOM	297	HB1	TYR	N	44	2.146	-2.523	-7.193	1.00	0.00	H
ATOM	298	HB2	TYR	N	44	2.976	-3.535	-6.009	1.00	0.00	H
ATOM	299	CG	TYR	N	44	3.666	-3.804	-8.048	1.00	0.00	C
ATOM	300	O	TYR	N	44	2.656	-0.983	-4.830	1.00	0.00	O
ATOM	301	N	ALA	N	45	4.357	0.343	-5.572	1.00	0.00	N
ATOM	302	HB1	ALA	N	9	2.570	2.862	-13.789	1.00	0.00	H
ATOM	303	HB2	ALA	N	9	1.985	2.959	-12.110	1.00	0.00	H
ATOM	304	HB3	ALA	N	9	1.026	2.106	-13.358	1.00	0.00	H
ATOM	305	O	ALA	N	9	-1.422	4.061	-13.372	1.00	0.00	O
ATOM	306	N	ARG	N	10	-0.349	4.817	-11.517	1.00	0.00	N
ATOM	307	O	THR	N	28	-1.711	-3.633	-18.795	1.00	0.00	O
ATOM	308	CA	THR	N	28	-2.958	-4.954	-17.222	1.00	0.00	C
ATOM	309	CD1	TYR	N	29	-5.050	0.659	-20.596	1.00	0.00	C
ATOM	310	CD2	TYR	N	29	-4.771	1.989	-18.626	1.00	0.00	C
ATOM	311	HG21	ILE	N	35	-0.296	-8.416	-3.840	1.00	0.00	H
ATOM	312	HG22	ILE	N	35	-1.166	-8.101	-5.355	1.00	0.00	H
ATOM	313	HG23	ILE	N	35	-1.217	-9.718	-4.621	1.00	0.00	H
ATOM	314	HG11	ILE	N	35	-2.235	-6.101	-3.948	1.00	0.00	H
ATOM	315	HG12	ILE	N	35	-3.436	-6.498	-2.726	1.00	0.00	H
ATOM	316	CD1	ILE	N	35	-1.410	-6.522	-2.004	1.00	0.00	C
ATOM	317	HD1	PRO	N	36	-3.368	-10.897	-3.063	1.00	0.00	H
ATOM	318	HD2	PRO	N	36	-4.593	-11.452	-4.233	1.00	0.00	H
ATOM	319	HG1	PRO	N	36	-4.950	-13.041	-2.436	1.00	0.00	H
ATOM	320	HG2	PRO	N	36	-4.485	-11.848	-1.211	1.00	0.00	H
ATOM	321	CD1	TYR	N	44	3.370	-3.510	-9.382	1.00	0.00	C
ATOM	322	CD2	TYR	N	44	4.565	-4.848	-7.791	1.00	0.00	C
ATOM	323	H	ALA	N	45	5.095	0.431	-6.265	1.00	0.00	H
ATOM	324	CA	ALA	N	45	4.362	1.255	-4.455	1.00	0.00	C
ATOM	325	H	ARG	N	10	0.546	5.022	-11.071	1.00	0.00	H
ATOM	326	CA	ARG	N	10	-1.582	5.064	-10.785	1.00	0.00	C
ATOM	327	HA	THR	N	28	-1.945	-5.297	-16.969	1.00	0.00	H
ATOM	328	N	THR	N	28	-3.678	-4.675	-15.981	1.00	0.00	N
ATOM	329	CB	THR	N	28	-3.628	-6.063	-18.044	1.00	0.00	C
ATOM	330	HD1	TYR	N	29	-5.119	-0.309	-21.098	1.00	0.00	H
ATOM	331	CE1	TYR	N	29	-5.206	1.817	-21.355	1.00	0.00	C
ATOM	332	HD2	TYR	N	29	-4.614	2.077	-17.551	1.00	0.00	H
ATOM	333	CE2	TYR	N	29	-4.923	3.160	-19.367	1.00	0.00	C
ATOM	334	HD11	ILE	N	35	-1.364	-5.447	-1.786	1.00	0.00	H
ATOM	335	HD12	ILE	N	35	-0.386	-6.804	-2.290	1.00	0.00	H
ATOM	336	HD13	ILE	N	35	-1.659	-7.078	-1.083	1.00	0.00	H
ATOM	337	HD1	TYR	N	44	2.676	-2.704	-9.616	1.00	0.00	H
ATOM	338	CE1	TYR	N	44	3.909	-4.252	-10.433	1.00	0.00	C
ATOM	339	HD2	TYR	N	44	4.819	-5.113	-6.767	1.00	0.00	H
ATOM	340	CE2	TYR	N	44	5.150	-5.574	-8.826	1.00	0.00	C
ATOM	341	HA	ALA	N	45	4.451	0.662	-3.538	1.00	0.00	H
ATOM	342	C	ALA	N	45	3.089	2.073	-4.294	1.00	0.00	C
ATOM	343	CB	ALA	N	45	5.559	2.210	-4.577	1.00	0.00	C

ATOM	344	HA	ARG	N	10	-2.199	4.160	-10.855	1.00	0.00	H
ATOM	345	CB	ARG	N	10	-1.305	5.363	-9.300	1.00	0.00	C
ATOM	346	C	ARG	N	10	-2.450	6.145	-11.463	1.00	0.00	C
ATOM	347	H	THR	N	28	-4.647	-4.982	-15.876	1.00	0.00	H
ATOM	348	C	ALA	N	27	-3.107	-3.861	-15.065	1.00	0.00	C
ATOM	349	HB	THR	N	28	-3.072	-6.102	-18.998	1.00	0.00	H
ATOM	350	OG1	THR	N	28	-4.973	-5.624	-18.286	1.00	0.00	O
ATOM	351	CG2	THR	N	28	-3.568	-7.426	-17.369	1.00	0.00	C
ATOM	352	HE1	TYR	N	29	-5.395	1.746	-22.428	1.00	0.00	H
ATOM	353	CZ	TYR	N	29	-5.116	3.075	-20.747	1.00	0.00	C
ATOM	354	HE2	TYR	N	29	-4.882	4.136	-18.879	1.00	0.00	H
ATOM	355	HE1	TYR	N	44	3.588	-4.038	-11.456	1.00	0.00	H
ATOM	356	CZ	TYR	N	44	4.802	-5.288	-10.148	1.00	0.00	C
ATOM	357	HE2	TYR	N	44	5.859	-6.371	-8.601	1.00	0.00	H
ATOM	358	O	ALA	N	45	2.831	2.503	-3.142	1.00	0.00	O
ATOM	359	N	ASN	N	46	2.375	2.396	-5.368	1.00	0.00	N
ATOM	360	HB1	ALA	N	45	5.630	2.845	-3.683	1.00	0.00	H
ATOM	361	HB2	ALA	N	45	6.491	1.633	-4.675	1.00	0.00	H
ATOM	362	HB3	ALA	N	45	5.454	2.863	-5.457	1.00	0.00	H
ATOM	363	HB1	ARG	N	10	-0.721	4.528	-8.884	1.00	0.00	H
ATOM	364	HB2	ARG	N	10	-0.668	6.257	-9.232	1.00	0.00	H
ATOM	365	CG	ARG	N	10	-2.577	5.592	-8.471	1.00	0.00	C
ATOM	366	O	ARG	N	10	-3.664	5.925	-11.606	1.00	0.00	O
ATOM	367	N	SER	N	11	-1.879	7.285	-11.895	1.00	0.00	N
ATOM	368	O	ALA	N	27	-1.983	-3.376	-15.232	1.00	0.00	O
ATOM	369	CA	ALA	N	27	-3.899	-3.519	-13.788	1.00	0.00	C
ATOM	370	HG1	THR	N	28	-5.391	-6.245	-18.903	1.00	0.00	H
ATOM	371	HG21	THR	N	28	-2.533	-7.770	-17.246	1.00	0.00	H
ATOM	372	HG22	THR	N	28	-4.052	-7.420	-16.381	1.00	0.00	H
ATOM	373	HG23	THR	N	28	-4.088	-8.175	-17.990	1.00	0.00	H
ATOM	374	OH	TYR	N	29	-5.222	4.242	-21.460	1.00	0.00	O
ATOM	375	OH	TYR	N	44	5.363	-6.061	-11.142	1.00	0.00	O
ATOM	376	H	ASN	N	46	2.589	1.920	-6.255	1.00	0.00	H
ATOM	377	CA	ASN	N	46	1.468	3.539	-5.397	1.00	0.00	C
ATOM	378	HG1	ARG	N	10	-3.166	6.389	-8.939	1.00	0.00	H
ATOM	379	HG2	ARG	N	10	-2.287	5.982	-7.484	1.00	0.00	H
ATOM	380	CD	ARG	N	10	-3.509	4.390	-8.278	1.00	0.00	C
ATOM	381	H	SER	N	11	-0.900	7.487	-11.660	1.00	0.00	H
ATOM	382	CA	SER	N	11	-2.657	8.268	-12.648	1.00	0.00	C
ATOM	383	HA	ALA	N	27	-3.262	-2.787	-13.286	1.00	0.00	H
ATOM	384	N	ALA	N	27	-5.167	-2.878	-14.122	1.00	0.00	N
ATOM	385	CB	ALA	N	27	-4.133	-4.723	-12.874	1.00	0.00	C
ATOM	386	HH	TYR	N	29	-5.017	4.054	-22.389	1.00	0.00	H
ATOM	387	HH	TYR	N	44	4.934	-5.840	-11.983	1.00	0.00	H
ATOM	388	HA	ASN	N	46	1.457	3.962	-4.386	1.00	0.00	H
ATOM	389	CB	ASN	N	46	2.013	4.579	-6.393	1.00	0.00	C
ATOM	390	C	ASN	N	46	-0.007	3.214	-5.708	1.00	0.00	C
ATOM	391	HD1	ARG	N	10	-3.656	3.858	-9.232	1.00	0.00	H
ATOM	392	HD2	ARG	N	10	-4.505	4.757	-7.978	1.00	0.00	H
ATOM	393	NE	ARG	N	10	-2.981	3.472	-7.269	1.00	0.00	N
ATOM	394	HA	SER	N	11	-3.438	8.700	-12.003	1.00	0.00	H
ATOM	395	CB	SER	N	11	-1.777	9.401	-13.195	1.00	0.00	C
ATOM	396	C	SER	N	11	-3.404	7.633	-13.841	1.00	0.00	C
ATOM	397	H	ALA	N	27	-6.020	-3.397	-13.932	1.00	0.00	H
ATOM	398	C	CYS	N	26	-5.276	-1.697	-14.747	1.00	0.00	C
ATOM	399	HB1	ALA	N	27	-3.171	-5.196	-12.653	1.00	0.00	H
ATOM	400	HB2	ALA	N	27	-4.797	-5.457	-13.349	1.00	0.00	H
ATOM	401	HB3	ALA	N	27	-4.589	-4.390	-11.929	1.00	0.00	H
ATOM	402	HB1	ASN	N	46	1.955	4.199	-7.422	1.00	0.00	H
ATOM	403	HB2	ASN	N	46	1.380	5.476	-6.327	1.00	0.00	H
ATOM	404	CG	ASN	N	46	3.469	4.938	-6.136	1.00	0.00	C
ATOM	405	OXT	ASN	N	46	-0.774	4.211	-5.764	1.00	0.00	O
ATOM	406	O	ASN	N	46	-0.344	2.010	-5.899	1.00	0.00	O

ATOM	407	HE	ARG	N	10	-2.167	3.786	-6.691	1.00	0.00	H
ATOM	408	CZ	ARG	N	10	-3.638	2.403	-6.798	1.00	0.00	C
ATOM	409	HB1	SER	N	11	-1.044	8.986	-13.905	1.00	0.00	H
ATOM	410	HB2	SER	N	11	-2.441	10.081	-13.757	1.00	0.00	H
ATOM	411	OG	SER	N	11	-1.132	10.066	-12.123	1.00	0.00	O
ATOM	412	O	SER	N	11	-4.600	7.885	-14.062	1.00	0.00	O
ATOM	413	N	ASN	N	12	-2.642	6.856	-14.623	1.00	0.00	N
ATOM	414	O	CYS	N	26	-4.323	-0.934	-14.976	1.00	0.00	O
ATOM	415	CA	CYS	N	26	-6.677	-1.312	-15.248	1.00	0.00	C
ATOM	416	OD1	ASN	N	46	4.341	4.771	-6.995	1.00	0.00	O
ATOM	417	ND2	ASN	N	46	3.762	5.458	-4.920	1.00	0.00	N
ATOM	418	NH2	ARG	N	10	-3.142	1.772	-5.723	1.00	0.00	N
ATOM	419	NH1	ARG	N	10	-4.797	2.004	-7.349	1.00	0.00	N
ATOM	420	H	SER	N	11	-0.958	11.001	-12.376	1.00	0.00	H
ATOM	421	H	ASN	N	12	-1.642	6.781	-14.413	1.00	0.00	H
ATOM	422	CA	ASN	N	12	-3.121	6.303	-15.882	1.00	0.00	C
ATOM	423	HA	CYS	N	26	-6.485	-0.887	-16.242	1.00	0.00	H
ATOM	424	N	CYS	N	26	-7.545	-2.456	-15.414	1.00	0.00	N
ATOM	425	CB	CYS	N	26	-7.357	-0.244	-14.401	1.00	0.00	C
ATOM	426	HD21	ASN	N	46	4.726	5.726	-4.756	1.00	0.00	H
ATOM	427	HD22	ASN	N	46	3.099	5.574	-4.153	1.00	0.00	H
ATOM	428	HH21	ARG	N	10	-3.544	0.876	-5.431	1.00	0.00	H
ATOM	429	HH22	ARG	N	10	-2.125	1.894	-5.569	1.00	0.00	H
ATOM	430	HH11	ARG	N	10	-5.087	2.399	-8.236	1.00	0.00	H
ATOM	431	HH12	ARG	N	10	-5.243	1.112	-7.107	1.00	0.00	H
ATOM	432	HA	ASN	N	12	-3.643	7.088	-16.445	1.00	0.00	H
ATOM	433	CB	ASN	N	12	-1.926	5.789	-16.702	1.00	0.00	C
ATOM	434	C	ASN	N	12	-4.184	5.214	-15.608	1.00	0.00	C
ATOM	435	H	CYS	N	26	-8.316	-2.596	-14.764	1.00	0.00	H
ATOM	436	C	LEU	N	25	-7.409	-3.270	-16.485	1.00	0.00	C
ATOM	437	HB1	CYS	N	26	-6.704	0.626	-14.259	1.00	0.00	H
ATOM	438	HB2	CYS	N	26	-7.619	-0.630	-13.404	1.00	0.00	H
ATOM	439	SG	CYS	N	26	-8.961	0.281	-15.125	1.00	0.00	S
ATOM	440	HB1	ASN	N	12	-2.297	5.288	-17.610	1.00	0.00	H
ATOM	441	HB2	ASN	N	12	-1.371	5.031	-16.125	1.00	0.00	H
ATOM	442	CG	ASN	N	12	-0.964	6.930	-17.094	1.00	0.00	C
ATOM	443	O	ASN	N	12	-5.213	5.112	-16.295	1.00	0.00	O
ATOM	444	N	PHE	N	13	-3.914	4.432	-14.550	1.00	0.00	N
ATOM	445	O	LEU	N	25	-6.567	-3.080	-17.365	1.00	0.00	O
ATOM	446	CA	LEU	N	25	-8.373	-4.458	-16.553	1.00	0.00	C
ATOM	447	SG	CYS	N	16	-8.419	0.856	-17.056	1.00	0.00	S
ATOM	448	OD1	ASN	N	12	-1.147	8.082	-16.693	1.00	0.00	O
ATOM	449	ND2	ASN	N	12	0.077	6.604	-17.882	1.00	0.00	N
ATOM	450	H	PHE	N	13	-3.024	4.556	-14.056	1.00	0.00	H
ATOM	451	CA	PHE	N	13	-4.835	3.463	-13.984	1.00	0.00	C
ATOM	452	HA	LEU	N	25	-7.817	-5.221	-17.110	1.00	0.00	H
ATOM	453	N	LEU	N	25	-8.673	-4.980	-15.220	1.00	0.00	N
ATOM	454	CB	LEU	N	25	-9.675	-4.076	-17.269	1.00	0.00	C
ATOM	455	CB	CYS	N	16	-7.826	2.567	-16.817	1.00	0.00	C
ATOM	456	HD21	ASN	N	12	0.272	5.681	-18.257	1.00	0.00	H
ATOM	457	HD22	ASN	N	12	0.684	7.367	-18.189	1.00	0.00	H
ATOM	458	HA	PHE	N	13	-5.105	2.703	-14.733	1.00	0.00	H
ATOM	459	C	PHE	N	13	-6.152	4.128	-13.574	1.00	0.00	C
ATOM	460	CB	PHE	N	13	-4.134	2.804	-12.773	1.00	0.00	C
ATOM	461	H	LEU	N	25	-9.553	-4.710	-14.783	1.00	0.00	H
ATOM	462	C	ALA	N	24	-7.680	-5.444	-14.426	1.00	0.00	C
ATOM	463	HB1	LEU	N	25	-10.260	-3.457	-16.576	1.00	0.00	H
ATOM	464	HB2	LEU	N	25	-9.425	-3.428	-18.125	1.00	0.00	H
ATOM	465	CG	LEU	N	25	-10.528	-5.263	-17.753	1.00	0.00	C
ATOM	466	HB1	CYS	N	16	-7.235	2.607	-15.893	1.00	0.00	H
ATOM	467	HB2	CYS	N	16	-7.150	2.753	-17.664	1.00	0.00	H
ATOM	468	CA	CYS	N	16	-8.910	3.649	-16.795	1.00	0.00	C
ATOM	469	O	PHE	N	13	-7.237	3.579	-13.826	1.00	0.00	O

```
ATOM    470  N   ASN N  14      -6.046   5.293 -12.919  1.00  0.00           N
ATOM    471  HB1 PHE N  13      -3.671   3.609 -12.195  1.00  0.00           H
ATOM    472  HB2 PHE N  13      -3.323   2.172 -13.152  1.00  0.00           H
ATOM    473  CG  PHE N  13      -5.034   2.009 -11.872  1.00  0.00           C
ATOM    474  O   ALA N  24      -6.510  -5.532 -14.804  1.00  0.00           O
ATOM    475  CA  ALA N  24      -8.085  -5.948 -13.033  1.00  0.00           C
ATOM    476  HG  LEU N  25     -10.658  -5.948 -16.901  1.00  0.00           H
ATOM    477  CD1 LEU N  25      -9.838  -6.038 -18.876  1.00  0.00           C
ATOM    478  CD2 LEU N  25     -11.923  -4.783 -18.177  1.00  0.00           C
ATOM    479  HA  CYS N  16      -9.364   3.734 -17.789  1.00  0.00           H
ATOM    480  N   CYS N  16      -8.265   4.900 -16.439  1.00  0.00           N
ATOM    481  C   CYS N  16     -10.115   3.340 -15.881  1.00  0.00           C
ATOM    482  H   ASN N  14      -5.116   5.628 -12.646  1.00  0.00           H
ATOM    483  CA  ASN N  14      -7.209   6.003 -12.421  1.00  0.00           C
ATOM    484  CD1 PHE N  13      -5.130   0.622 -12.016  1.00  0.00           C
ATOM    485  CD2 PHE N  13      -5.791   2.646 -10.875  1.00  0.00           C
ATOM    486  HA  ALA N  24      -7.147  -5.971 -12.460  1.00  0.00           H
ATOM    487  N   ALA N  24      -9.009  -5.040 -12.354  1.00  0.00           N
ATOM    488  CB  ALA N  24      -8.664  -7.357 -13.150  1.00  0.00           C
ATOM    489 HD11 LEU N  25      -8.869  -6.456 -18.569  1.00  0.00           H
ATOM    490 HD12 LEU N  25     -10.451  -6.884 -19.220  1.00  0.00           H
ATOM    491 HD13 LEU N  25      -9.656  -5.389 -19.746  1.00  0.00           H
ATOM    492 HD21 LEU N  25     -12.459  -4.295 -17.350  1.00  0.00           H
ATOM    493 HD22 LEU N  25     -12.550  -5.620 -18.521  1.00  0.00           H
ATOM    494 HD23 LEU N  25     -11.861  -4.050 -18.999  1.00  0.00           H
ATOM    495  H   CYS N  16      -7.275   4.900 -16.183  1.00  0.00           H
ATOM    496  C   VAL N  15      -8.971   6.052 -16.410  1.00  0.00           C
ATOM    497  O   CYS N  16     -11.188   2.966 -16.385  1.00  0.00           O
ATOM    498  N   ARG N  17      -9.977   3.500 -14.556  1.00  0.00           N
ATOM    499  HA  ASN N  14      -7.833   5.322 -11.824  1.00  0.00           H
ATOM    500  CB  ASN N  14      -6.783   7.188 -11.557  1.00  0.00           C
ATOM    501  C   ASN N  14      -8.107   6.495 -13.568  1.00  0.00           C
ATOM    502  HD1 PHE N  13      -4.534   0.132 -12.785  1.00  0.00           H
ATOM    503  CE1 PHE N  13      -5.984  -0.122 -11.203  1.00  0.00           C
ATOM    504  HD2 PHE N  13      -5.724   3.724 -10.728  1.00  0.00           H
ATOM    505  CE2 PHE N  13      -6.645   1.901 -10.064  1.00  0.00           C
ATOM    506  H   ALA N  24      -9.922  -5.378 -12.067  1.00  0.00           H
ATOM    507  C   GLU N  23      -8.607  -3.799 -11.966  1.00  0.00           C
ATOM    508  HB1 ALA N  24      -7.916  -8.018 -13.603  1.00  0.00           H
ATOM    509  HB2 ALA N  24      -9.556  -7.374 -13.785  1.00  0.00           H
ATOM    510  HB3 ALA N  24      -8.929  -7.767 -12.167  1.00  0.00           H
ATOM    511  O   VAL N  15     -10.145   6.126 -16.780  1.00  0.00           O
ATOM    512  CA  VAL N  15      -8.225   7.280 -15.881  1.00  0.00           C
ATOM    513  H   ARG N  17      -9.050   3.723 -14.179  1.00  0.00           H
ATOM    514  CA  ARG N  17     -11.083   3.166 -13.664  1.00  0.00           C
ATOM    515  HB1 ASN N  14      -6.121   7.853 -12.133  1.00  0.00           H
ATOM    516  HB2 ASN N  14      -7.690   7.758 -11.303  1.00  0.00           H
ATOM    517  CG  ASN N  14      -6.135   6.790 -10.229  1.00  0.00           C
ATOM    518  O   ASN N  14      -9.340   6.479 -13.445  1.00  0.00           O
ATOM    519  N   VAL N  15      -7.479   6.958 -14.668  1.00  0.00           N
ATOM    520  HE1 PHE N  13      -6.063  -1.202 -11.344  1.00  0.00           H
ATOM    521  CZ  PHE N  13      -6.750   0.519 -10.229  1.00  0.00           C
ATOM    522  HE2 PHE N  13      -7.235   2.406  -9.295  1.00  0.00           H
ATOM    523  O   GLU N  23      -7.506  -3.348 -12.290  1.00  0.00           O
ATOM    524  CA  GLU N  23      -9.515  -3.010 -11.008  1.00  0.00           C
ATOM    525  HA  VAL N  15      -9.026   7.990 -15.628  1.00  0.00           H
ATOM    526  CB  VAL N  15      -7.305   7.925 -16.943  1.00  0.00           C
ATOM    527  HA  ARG N  17     -11.483   2.177 -13.928  1.00  0.00           H
ATOM    528  C   ARG N  17     -12.267   4.129 -13.860  1.00  0.00           C
ATOM    529  CB  ARG N  17     -10.658   3.186 -12.181  1.00  0.00           C
ATOM    530  OD1 ASN N  14      -6.378   5.730  -9.659  1.00  0.00           O
ATOM    531  ND2 ASN N  14      -5.346   7.761  -9.704  1.00  0.00           N
ATOM    532  H   VAL N  15      -6.456   6.992 -14.681  1.00  0.00           H
```

ATOM	533	HZ	PHE	N	13	-7.422	-0.046	-9.590	1.00	0.00	H
ATOM	534	HA	GLU	N	23	-9.031	-2.029	-10.908	1.00	0.00	H
ATOM	535	N	GLU	N	23	-10.878	-2.855	-11.511	1.00	0.00	N
ATOM	536	CB	GLU	N	23	-9.566	-3.715	-9.634	1.00	0.00	C
ATOM	537	HB	VAL	N	15	-6.371	7.338	-16.968	1.00	0.00	H
ATOM	538	CG2	VAL	N	15	-6.995	9.357	-16.515	1.00	0.00	C
ATOM	539	CG1	VAL	N	15	-7.935	7.871	-18.337	1.00	0.00	C
ATOM	540	O	ARG	N	17	-13.428	3.728	-13.686	1.00	0.00	O
ATOM	541	N	LEU	N	18	-11.971	5.400	-14.194	1.00	0.00	N
ATOM	542	HB1	ARG	N	17	-11.566	3.004	-11.583	1.00	0.00	H
ATOM	543	HB2	ARG	N	17	-10.326	4.210	-11.940	1.00	0.00	H
ATOM	544	CG	ARG	N	17	-9.540	2.209	-11.775	1.00	0.00	C
ATOM	545	HD21	ASN	N	14	-5.060	8.542	-10.279	1.00	0.00	H
ATOM	546	HD22	ASN	N	14	-4.848	7.637	-8.823	1.00	0.00	H
ATOM	547	H	GLU	N	23	-11.583	-2.650	-10.808	1.00	0.00	H
ATOM	548	C	PRO	N	22	-11.266	-2.748	-12.807	1.00	0.00	C
ATOM	549	HB1	GLU	N	23	-8.548	-4.025	-9.366	1.00	0.00	H
ATOM	550	HB2	GLU	N	23	-10.143	-4.644	-9.765	1.00	0.00	H
ATOM	551	CG	GLU	N	23	-10.188	-2.918	-8.466	1.00	0.00	C
ATOM	552	HG21	VAL	N	15	-6.391	9.888	-17.258	1.00	0.00	H
ATOM	553	HG22	VAL	N	15	-6.442	9.398	-15.568	1.00	0.00	H
ATOM	554	HG23	VAL	N	15	-7.933	9.920	-16.391	1.00	0.00	H
ATOM	555	HG11	VAL	N	15	-7.344	8.448	-19.060	1.00	0.00	H
ATOM	556	HG12	VAL	N	15	-8.001	6.838	-18.707	1.00	0.00	H
ATOM	557	HG13	VAL	N	15	-8.953	8.291	-18.348	1.00	0.00	H
ATOM	558	H	LEU	N	18	-10.988	5.681	-14.230	1.00	0.00	H
ATOM	559	CA	LEU	N	18	-12.955	6.477	-14.196	1.00	0.00	C
ATOM	560	HG1	ARG	N	17	-8.694	2.305	-12.471	1.00	0.00	H
ATOM	561	HG2	ARG	N	17	-9.147	2.538	-10.803	1.00	0.00	H
ATOM	562	CD	ARG	N	17	-9.886	0.713	-11.659	1.00	0.00	C
ATOM	563	O	PRO	N	22	-10.497	-2.840	-13.767	1.00	0.00	O
ATOM	564	CA	PRO	N	22	-12.790	-2.652	-12.951	1.00	0.00	C
ATOM	565	HG1	GLU	N	23	-10.611	-3.644	-7.764	1.00	0.00	H
ATOM	566	HG2	GLU	N	23	-11.016	-2.282	-8.815	1.00	0.00	H
ATOM	567	CD	GLU	N	23	-9.173	-2.069	-7.678	1.00	0.00	C
ATOM	568	HA	LEU	N	18	-13.198	6.717	-13.150	1.00	0.00	H
ATOM	569	C	LEU	N	18	-14.314	6.107	-14.797	1.00	0.00	C
ATOM	570	CB	LEU	N	18	-12.415	7.742	-14.913	1.00	0.00	C
ATOM	571	HD1	ARG	N	17	-8.963	0.140	-11.806	1.00	0.00	H
ATOM	572	HD2	ARG	N	17	-10.575	0.389	-12.450	1.00	0.00	H
ATOM	573	NE	ARG	N	17	-10.395	0.297	-10.346	1.00	0.00	N
ATOM	574	HA	PRO	N	22	-13.183	-2.006	-12.149	1.00	0.00	H
ATOM	575	N	PRO	N	22	-13.195	-2.121	-14.246	1.00	0.00	N
ATOM	576	CB	PRO	N	22	-13.433	-4.055	-12.892	1.00	0.00	C
ATOM	577	OE2	GLU	N	23	-8.667	-2.593	-6.660	1.00	0.00	O
ATOM	578	OE1	GLU	N	23	-8.875	-0.914	-8.148	1.00	0.00	O
ATOM	579	O	LEU	N	18	-15.343	6.385	-14.141	1.00	0.00	O
ATOM	580	N	PRO	N	19	-14.398	5.598	-16.037	1.00	0.00	N
ATOM	581	HB1	LEU	N	18	-11.389	7.544	-15.249	1.00	0.00	H
ATOM	582	HB2	LEU	N	18	-12.990	7.912	-15.838	1.00	0.00	H
ATOM	583	CG	LEU	N	18	-12.458	9.033	-14.080	1.00	0.00	C
ATOM	584	HE	ARG	N	17	-9.722	0.014	-9.617	1.00	0.00	H
ATOM	585	CZ	ARG	N	17	-11.679	0.294	-9.955	1.00	0.00	C
ATOM	586	C	THR	N	21	-12.955	-0.822	-14.526	1.00	0.00	C
ATOM	587	CD	PRO	N	22	-14.097	-3.031	-14.968	1.00	0.00	C
ATOM	588	HB1	PRO	N	22	-13.725	-4.340	-11.875	1.00	0.00	H
ATOM	589	HB2	PRO	N	22	-12.717	-4.805	-13.260	1.00	0.00	H
ATOM	590	CG	PRO	N	22	-14.617	-3.948	-13.858	1.00	0.00	C
ATOM	591	CA	PRO	N	19	-15.720	5.360	-16.644	1.00	0.00	C
ATOM	592	CD	PRO	N	19	-13.290	5.300	-16.984	1.00	0.00	C
ATOM	593	HG	LEU	N	18	-11.788	8.886	-13.211	1.00	0.00	H
ATOM	594	CD1	LEU	N	18	-11.902	10.181	-14.932	1.00	0.00	C
ATOM	595	CD2	LEU	N	18	-13.857	9.382	-13.555	1.00	0.00	C

```
ATOM    596  NH1 ARG N  17     -12.688   0.334 -10.835  1.00  0.00           N
ATOM    597  NH2 ARG N  17     -11.967   0.212  -8.650  1.00  0.00           N
ATOM    598  O   THR N  21     -12.370  -0.089 -13.712  1.00  0.00           O
ATOM    599  CA  THR N  21     -13.378  -0.259 -15.890  1.00  0.00           C
ATOM    600  HD1 PRO N  22     -14.900  -2.462 -15.446  1.00  0.00           H
ATOM    601  HD2 PRO N  22     -13.540  -3.594 -15.732  1.00  0.00           H
ATOM    602  HG1 PRO N  22     -14.932  -4.933 -14.237  1.00  0.00           H
ATOM    603  HG2 PRO N  22     -15.478  -3.475 -13.360  1.00  0.00           H
ATOM    604  HA  PRO N  19     -16.394   6.201 -16.437  1.00  0.00           H
ATOM    605  CB  PRO N  19     -15.384   5.212 -18.134  1.00  0.00           C
ATOM    606  C   PRO N  19     -16.440   4.099 -16.137  1.00  0.00           C
ATOM    607  HD1 PRO N  19     -12.508   4.705 -16.514  1.00  0.00           H
ATOM    608  HD2 PRO N  19     -12.832   6.240 -17.327  1.00  0.00           H
ATOM    609  CG  PRO N  19     -13.996   4.574 -18.129  1.00  0.00           C
ATOM    610 HD11 LEU N  18     -10.934   9.919 -15.383  1.00  0.00           H
ATOM    611 HD12 LEU N  18     -11.757  11.102 -14.346  1.00  0.00           H
ATOM    612 HD13 LEU N  18     -12.588  10.427 -15.760  1.00  0.00           H
ATOM    613 HD21 LEU N  18     -14.293   8.580 -12.943  1.00  0.00           H
ATOM    614 HD22 LEU N  18     -13.838  10.287 -12.932  1.00  0.00           H
ATOM    615 HD23 LEU N  18     -14.556   9.554 -14.390  1.00  0.00           H
ATOM    616 HH11 ARG N  17     -12.506   0.335 -11.840  1.00  0.00           H
ATOM    617 HH12 ARG N  17     -13.590   0.721 -10.504  1.00  0.00           H
ATOM    618 HH21 ARG N  17     -11.218   0.221  -7.965  1.00  0.00           H
ATOM    619 HH22 ARG N  17     -12.903  -0.100  -8.341  1.00  0.00           H
ATOM    620  HA  THR N  21     -13.962  -1.001 -16.452  1.00  0.00           H
ATOM    621  N   THR N  21     -14.199   0.906 -15.672  1.00  0.00           N
ATOM    622  CB  THR N  21     -12.125   0.111 -16.711  1.00  0.00           C
ATOM    623  HB1 PRO N  19     -16.139   4.640 -18.677  1.00  0.00           H
ATOM    624  HB2 PRO N  19     -15.318   6.207 -18.591  1.00  0.00           H
ATOM    625  O   PRO N  19     -17.608   3.903 -16.458  1.00  0.00           O
ATOM    626  N   GLY N  20     -15.679   3.220 -15.427  1.00  0.00           N
ATOM    627  HG1 PRO N  19     -13.484   4.702 -19.089  1.00  0.00           H
ATOM    628  HG2 PRO N  19     -14.054   3.494 -17.919  1.00  0.00           H
ATOM    629  H   THR N  21     -13.886   1.799 -16.035  1.00  0.00           H
ATOM    630  C   GLY N  20     -15.404   0.758 -15.092  1.00  0.00           C
ATOM    631  HB  THR N  21     -11.434   0.622 -16.030  1.00  0.00           H
ATOM    632  OG1 THR N  21     -12.489   1.015 -17.743  1.00  0.00           O
ATOM    633  CG2 THR N  21     -11.472  -1.146 -17.252  1.00  0.00           C
ATOM    634  H   GLY N  20     -14.832   3.563 -14.964  1.00  0.00           H
ATOM    635  CA  GLY N  20     -16.247   2.004 -14.888  1.00  0.00           C
ATOM    636  O   GLY N  20     -15.802  -0.350 -14.712  1.00  0.00           O
ATOM    637  HG1 THR N  21     -11.987   1.833 -17.545  1.00  0.00           H
ATOM    638 HG21 THR N  21     -11.151  -1.776 -16.412  1.00  0.00           H
ATOM    639 HG22 THR N  21     -10.590  -0.898 -17.855  1.00  0.00           H
ATOM    640 HG23 THR N  21     -12.166  -1.722 -17.879  1.00  0.00           H
ATOM    641  HA1 GLY N  20     -17.223   1.821 -15.358  1.00  0.00           H
ATOM    642  HA2 GLY N  20     -16.411   2.111 -13.805  1.00  0.00           H
END
```

3) CRAMBIN – molecule as in the optimized 84W crystal

```
COMPND    CRAMBIN OPTIMIZED CRYSTAL 84W GEOMETRY
AUTHOR    DELLE PIANE, CORNO, ORLANDO, DOVESI, UGLIENGO
ATOM      1  N   THR N   1      -7.330   3.687  -4.789  1.00  0.00           N
ATOM      2  H1  THR N   1      -7.752   2.757  -4.913  1.00  0.00           H
ATOM      3  H2  THR N   1      -6.627   3.803  -5.557  1.00  0.00           H
ATOM      4  H3  THR N   1      -8.043   4.470  -4.836  1.00  0.00           H
ATOM      5  CA  THR N   1      -6.680   3.696  -3.453  1.00  0.00           C
```

ATOM	6	HA	THR	N	1	-7.478	3.693	-2.697	1.00	0.00	H
ATOM	7	C	THR	N	1	-5.830	4.954	-3.364	1.00	0.00	C
ATOM	8	CB	THR	N	1	-5.817	2.413	-3.308	1.00	0.00	C
ATOM	9	O	THR	N	1	-5.269	5.378	-4.383	1.00	0.00	O
ATOM	10	N	THR	N	2	-5.708	5.501	-2.151	1.00	0.00	N
ATOM	11	HB	THR	N	1	-4.968	2.514	-4.006	1.00	0.00	H
ATOM	12	OG1	THR	N	1	-6.631	1.324	-3.713	1.00	0.00	O
ATOM	13	CG2	THR	N	1	-5.286	2.221	-1.892	1.00	0.00	C
ATOM	14	H	THR	N	2	-6.142	5.059	-1.322	1.00	0.00	H
ATOM	15	CA	THR	N	2	-4.777	6.584	-1.921	1.00	0.00	C
ATOM	16	HG1	THR	N	1	-6.142	0.904	-4.465	1.00	0.00	H
ATOM	17	HG21	THR	N	1	-4.752	1.262	-1.834	1.00	0.00	H
ATOM	18	HG22	THR	N	1	-4.582	3.016	-1.607	1.00	0.00	H
ATOM	19	HG23	THR	N	1	-6.103	2.204	-1.154	1.00	0.00	H
ATOM	20	HA	THR	N	2	-4.418	6.907	-2.903	1.00	0.00	H
ATOM	21	C	THR	N	2	-3.548	6.115	-1.146	1.00	0.00	C
ATOM	22	CB	THR	N	2	-5.465	7.817	-1.269	1.00	0.00	C
ATOM	23	O	THR	N	2	-3.606	5.269	-0.228	1.00	0.00	O
ATOM	24	N	CYS	N	3	-2.428	6.734	-1.541	1.00	0.00	N
ATOM	25	HB	THR	N	2	-4.668	8.450	-0.842	1.00	0.00	H
ATOM	26	OG1	THR	N	2	-6.308	7.424	-0.182	1.00	0.00	O
ATOM	27	CG2	THR	N	2	-6.186	8.601	-2.355	1.00	0.00	C
ATOM	28	H	CYS	N	3	-2.498	7.387	-2.333	1.00	0.00	H
ATOM	29	CA	CYS	N	3	-1.096	6.499	-1.020	1.00	0.00	C
ATOM	30	HG1	THR	N	2	-7.255	7.613	-0.399	1.00	0.00	H
ATOM	31	HG21	THR	N	2	-6.673	9.485	-1.933	1.00	0.00	H
ATOM	32	HG22	THR	N	2	-5.458	8.940	-3.103	1.00	0.00	H
ATOM	33	HG23	THR	N	2	-6.945	7.987	-2.859	1.00	0.00	H
ATOM	34	HA	CYS	N	3	-1.166	5.864	-0.130	1.00	0.00	H
ATOM	35	C	CYS	N	3	-0.480	7.871	-0.707	1.00	0.00	C
ATOM	36	CB	CYS	N	3	-0.187	5.827	-2.071	1.00	0.00	C
ATOM	37	O	CYS	N	3	-0.670	8.807	-1.499	1.00	0.00	O
ATOM	38	N	CYS	N	4	0.301	7.984	0.376	1.00	0.00	N
ATOM	39	HB1	CYS	N	3	0.780	5.578	-1.615	1.00	0.00	H
ATOM	40	HB2	CYS	N	3	-0.008	6.522	-2.903	1.00	0.00	H
ATOM	41	SG	CYS	N	3	-0.874	4.341	-2.889	1.00	0.00	S
ATOM	42	H	CYS	N	4	0.398	7.221	1.058	1.00	0.00	H
ATOM	43	CA	CYS	N	4	0.852	9.279	0.732	1.00	0.00	C
ATOM	44	SG	CYS	N	40	-0.642	2.875	-1.434	1.00	0.00	S
ATOM	45	HA	CYS	N	4	0.601	9.947	-0.096	1.00	0.00	H
ATOM	46	C	CYS	N	4	2.372	9.194	0.862	1.00	0.00	C
ATOM	47	CB	CYS	N	4	0.216	9.836	2.014	1.00	0.00	C
ATOM	48	CB	CYS	N	40	1.140	2.454	-1.626	1.00	0.00	C
ATOM	49	O	CYS	N	4	2.923	8.200	1.355	1.00	0.00	O
ATOM	50	N	PRO	N	5	3.069	10.269	0.445	1.00	0.00	N
ATOM	51	HB1	CYS	N	4	0.237	9.091	2.821	1.00	0.00	H
ATOM	52	HB2	CYS	N	4	0.749	10.721	2.386	1.00	0.00	H
ATOM	53	SG	CYS	N	4	-1.556	10.271	1.813	1.00	0.00	S
ATOM	54	HB1	CYS	N	40	1.750	3.364	-1.567	1.00	0.00	H
ATOM	55	HB2	CYS	N	40	1.385	1.850	-0.739	1.00	0.00	H
ATOM	56	CA	CYS	N	40	1.438	1.661	-2.901	1.00	0.00	C
ATOM	57	CA	PRO	N	5	4.542	10.265	0.469	1.00	0.00	C
ATOM	58	CD	PRO	N	5	2.525	11.485	-0.212	1.00	0.00	C
ATOM	59	SG	CYS	N	32	-1.485	12.169	0.957	1.00	0.00	S
ATOM	60	HA	CYS	N	40	1.092	2.219	-3.781	1.00	0.00	H
ATOM	61	N	CYS	N	40	0.748	0.396	-2.846	1.00	0.00	N
ATOM	62	C	CYS	N	40	2.953	1.422	-2.997	1.00	0.00	C
ATOM	63	HA	PRO	N	5	4.937	9.295	0.150	1.00	0.00	H
ATOM	64	C	PRO	N	5	5.129	10.510	1.855	1.00	0.00	C
ATOM	65	CB	PRO	N	5	4.899	11.385	-0.516	1.00	0.00	C
ATOM	66	HD1	PRO	N	5	1.732	11.945	0.393	1.00	0.00	H
ATOM	67	HD2	PRO	N	5	2.109	11.221	-1.193	1.00	0.00	H
ATOM	68	CG	PRO	N	5	3.751	12.381	-0.362	1.00	0.00	C

ATOM	69	CB	CYS	N	32	-1.904	11.894	-0.820	1.00	0.00	C
ATOM	70	H	CYS	N	40	0.330	0.114	-1.950	1.00	0.00	H
ATOM	71	C	THR	N	39	0.635	-0.424	-3.898	1.00	0.00	C
ATOM	72	O	CYS	N	40	3.483	0.489	-2.371	1.00	0.00	O
ATOM	73	N	PRO	N	41	3.664	2.274	-3.748	1.00	0.00	N
ATOM	74	O	PRO	N	5	6.340	10.314	2.052	1.00	0.00	O
ATOM	75	N	SER	N	6	4.293	10.984	2.790	1.00	0.00	N
ATOM	76	HB1	PRO	N	5	5.886	11.812	-0.322	1.00	0.00	H
ATOM	77	HB2	PRO	N	5	4.896	10.967	-1.528	1.00	0.00	H
ATOM	78	HG1	PRO	N	5	3.660	13.066	-1.211	1.00	0.00	H
ATOM	79	HG2	PRO	N	5	3.875	13.000	0.532	1.00	0.00	H
ATOM	80	HB1	CYS	N	32	-2.298	10.875	-0.909	1.00	0.00	H
ATOM	81	HB2	CYS	N	32	-2.728	12.589	-1.040	1.00	0.00	H
ATOM	82	CA	CYS	N	32	-0.751	12.100	-1.810	1.00	0.00	C
ATOM	83	O	THR	N	39	1.085	-0.168	-5.027	1.00	0.00	O
ATOM	84	CA	THR	N	39	-0.173	-1.683	-3.597	1.00	0.00	C
ATOM	85	CA	PRO	N	41	5.115	2.137	-3.847	1.00	0.00	C
ATOM	86	CD	PRO	N	41	3.158	3.448	-4.489	1.00	0.00	C
ATOM	87	H	SER	N	6	3.301	11.068	2.595	1.00	0.00	H
ATOM	88	CA	SER	N	6	4.687	11.130	4.171	1.00	0.00	C
ATOM	89	HA	CYS	N	32	0.045	11.376	-1.595	1.00	0.00	H
ATOM	90	N	CYS	N	32	-0.218	13.450	-1.719	1.00	0.00	N
ATOM	91	C	CYS	N	32	-1.226	11.808	-3.261	1.00	0.00	C
ATOM	92	HA	THR	N	39	-0.205	-1.856	-2.515	1.00	0.00	H
ATOM	93	N	THR	N	39	-1.542	-1.407	-4.008	1.00	0.00	N
ATOM	94	CB	THR	N	39	0.380	-2.962	-4.254	1.00	0.00	C
ATOM	95	HA	PRO	N	41	5.384	1.092	-4.042	1.00	0.00	H
ATOM	96	C	PRO	N	41	5.784	2.584	-2.534	1.00	0.00	C
ATOM	97	CB	PRO	N	41	5.470	3.072	-5.023	1.00	0.00	C
ATOM	98	HD1	PRO	N	41	2.491	4.056	-3.862	1.00	0.00	H
ATOM	99	HD2	PRO	N	41	2.591	3.116	-5.375	1.00	0.00	H
ATOM	100	CG	PRO	N	41	4.433	4.190	-4.889	1.00	0.00	C
ATOM	101	HA	SER	N	6	5.405	10.334	4.399	1.00	0.00	H
ATOM	102	C	SER	N	6	3.462	10.940	5.051	1.00	0.00	C
ATOM	103	CB	SER	N	6	5.355	12.466	4.469	1.00	0.00	C
ATOM	104	H	CYS	N	32	-0.796	14.164	-1.262	1.00	0.00	H
ATOM	105	C	GLY	N	31	0.864	13.846	-2.414	1.00	0.00	C
ATOM	106	O	CYS	N	32	-1.251	12.708	-4.103	1.00	0.00	O
ATOM	107	N	ILE	N	33	-1.604	10.542	-3.520	1.00	0.00	N
ATOM	108	H	THR	N	39	-1.698	-1.126	-4.975	1.00	0.00	H
ATOM	109	C	ALA	N	38	-2.607	-1.567	-3.195	1.00	0.00	C
ATOM	110	HB	THR	N	39	0.385	-2.849	-5.346	1.00	0.00	H
ATOM	111	OG1	THR	N	39	-0.477	-4.040	-3.891	1.00	0.00	O
ATOM	112	CG2	THR	N	39	1.789	-3.294	-3.800	1.00	0.00	C
ATOM	113	O	PRO	N	41	5.201	3.290	-1.702	1.00	0.00	O
ATOM	114	N	GLY	N	42	7.078	2.214	-2.408	1.00	0.00	N
ATOM	115	HB1	PRO	N	41	6.498	3.450	-4.994	1.00	0.00	H
ATOM	116	HB2	PRO	N	41	5.338	2.531	-5.974	1.00	0.00	H
ATOM	117	HG1	PRO	N	41	4.292	4.757	-5.812	1.00	0.00	H
ATOM	118	HG2	PRO	N	41	4.742	4.878	-4.093	1.00	0.00	H
ATOM	119	O	SER	N	6	2.306	11.046	4.605	1.00	0.00	O
ATOM	120	N	ILE	N	7	3.758	10.679	6.329	1.00	0.00	N
ATOM	121	HB1	SER	N	6	5.848	12.406	5.443	1.00	0.00	H
ATOM	122	HB2	SER	N	6	6.142	12.621	3.724	1.00	0.00	H
ATOM	123	OG	SER	N	6	4.405	13.524	4.450	1.00	0.00	O
ATOM	124	O	GLY	N	31	1.598	13.050	-3.028	1.00	0.00	O
ATOM	125	CA	GLY	N	31	1.204	15.338	-2.451	1.00	0.00	C
ATOM	126	H	ILE	N	33	-1.442	9.819	-2.814	1.00	0.00	H
ATOM	127	CA	ILE	N	33	-1.872	10.088	-4.879	1.00	0.00	C
ATOM	128	O	ALA	N	38	-2.518	-1.960	-2.022	1.00	0.00	O
ATOM	129	CA	ALA	N	38	-3.968	-1.174	-3.802	1.00	0.00	C
ATOM	130	HG1	THR	N	39	-1.353	-3.907	-4.321	1.00	0.00	H
ATOM	131	HG21	THR	N	39	2.490	-2.506	-4.108	1.00	0.00	H

ATOM	132	HG22	THR	N	39	2.109	-4.233	-4.270	1.00	0.00	H
ATOM	133	HG23	THR	N	39	1.834	-3.386	-2.704	1.00	0.00	H
ATOM	134	H	GLY	N	42	7.484	1.614	-3.131	1.00	0.00	H
ATOM	135	CA	GLY	N	42	7.853	2.541	-1.224	1.00	0.00	C
ATOM	136	H	ILE	N	7	4.750	10.596	6.595	1.00	0.00	H
ATOM	137	CA	ILE	N	7	2.749	10.553	7.363	1.00	0.00	C
ATOM	138	H	SER	N	6	4.823	14.251	4.957	1.00	0.00	H
ATOM	139	HA1	GLY	N	31	1.359	15.576	-3.512	1.00	0.00	H
ATOM	140	HA2	GLY	N	31	2.160	15.482	-1.926	1.00	0.00	H
ATOM	141	N	GLY	N	31	0.196	16.216	-1.904	1.00	0.00	N
ATOM	142	HA	ILE	N	33	-2.189	10.960	-5.464	1.00	0.00	H
ATOM	143	C	ILE	N	33	-2.985	9.049	-4.861	1.00	0.00	C
ATOM	144	CB	ILE	N	33	-0.598	9.504	-5.576	1.00	0.00	C
ATOM	145	HA	ALA	N	38	-4.101	-0.107	-3.566	1.00	0.00	H
ATOM	146	N	ALA	N	38	-3.916	-1.278	-5.257	1.00	0.00	N
ATOM	147	CB	ALA	N	38	-5.113	-1.972	-3.178	1.00	0.00	C
ATOM	148	HA1	GLY	N	42	8.828	2.040	-1.306	1.00	0.00	H
ATOM	149	HA2	GLY	N	42	7.376	2.139	-0.319	1.00	0.00	H
ATOM	150	C	GLY	N	42	8.110	4.028	-0.984	1.00	0.00	C
ATOM	151	HA	ILE	N	7	2.005	9.801	7.058	1.00	0.00	H
ATOM	152	C	ILE	N	7	1.929	11.841	7.524	1.00	0.00	C
ATOM	153	CB	ILE	N	7	3.420	10.115	8.679	1.00	0.00	C
ATOM	154	H	GLY	N	31	-0.621	16.403	-2.490	1.00	0.00	H
ATOM	155	C	THR	N	30	0.193	16.648	-0.633	1.00	0.00	C
ATOM	156	O	ILE	N	33	-3.104	8.253	-3.912	1.00	0.00	O
ATOM	157	N	ILE	N	34	-3.750	9.001	-5.965	1.00	0.00	N
ATOM	158	HB	ILE	N	33	-0.903	9.302	-6.613	1.00	0.00	H
ATOM	159	CG2	ILE	N	33	0.488	10.578	-5.598	1.00	0.00	C
ATOM	160	CG1	ILE	N	33	-0.105	8.178	-4.961	1.00	0.00	C
ATOM	161	H	ALA	N	38	-3.570	-2.190	-5.588	1.00	0.00	H
ATOM	162	C	GLY	N	37	-4.464	-0.382	-6.101	1.00	0.00	C
ATOM	163	HB1	ALA	N	38	-6.075	-1.600	-3.555	1.00	0.00	H
ATOM	164	HB2	ALA	N	38	-5.098	-1.860	-2.086	1.00	0.00	H
ATOM	165	HB3	ALA	N	38	-5.014	-3.041	-3.416	1.00	0.00	H
ATOM	166	O	GLY	N	42	8.478	4.392	0.142	1.00	0.00	O
ATOM	167	N	ASP	N	43	7.932	4.869	-2.026	1.00	0.00	N
ATOM	168	O	ILE	N	7	0.693	11.766	7.610	1.00	0.00	O
ATOM	169	N	VAL	N	8	2.591	13.008	7.578	1.00	0.00	N
ATOM	170	HB	ILE	N	7	4.225	10.841	8.892	1.00	0.00	H
ATOM	171	CG2	ILE	N	7	2.427	10.182	9.831	1.00	0.00	C
ATOM	172	CG1	ILE	N	7	4.073	8.727	8.535	1.00	0.00	C
ATOM	173	O	THR	N	30	1.108	16.437	0.182	1.00	0.00	O
ATOM	174	CA	THR	N	30	-1.074	17.314	-0.118	1.00	0.00	C
ATOM	175	H	ILE	N	34	-3.692	9.750	-6.668	1.00	0.00	H
ATOM	176	CA	ILE	N	34	-4.645	7.891	-6.245	1.00	0.00	C
ATOM	177	HG21	ILE	N	33	1.419	10.176	-5.992	1.00	0.00	H
ATOM	178	HG22	ILE	N	33	0.715	10.953	-4.594	1.00	0.00	H
ATOM	179	HG23	ILE	N	33	0.187	11.431	-6.221	1.00	0.00	H
ATOM	180	HG11	ILE	N	33	-0.950	7.473	-4.901	1.00	0.00	H
ATOM	181	HG12	ILE	N	33	0.214	8.359	-3.921	1.00	0.00	H
ATOM	182	CD1	ILE	N	33	1.034	7.500	-5.733	1.00	0.00	C
ATOM	183	O	GLY	N	37	-5.052	0.640	-5.725	1.00	0.00	O
ATOM	184	CA	GLY	N	37	-4.301	-0.686	-7.601	1.00	0.00	C
ATOM	185	H	ASP	N	43	7.652	4.524	-2.943	1.00	0.00	H
ATOM	186	CA	ASP	N	43	8.021	6.314	-1.910	1.00	0.00	C
ATOM	187	H	VAL	N	8	3.620	13.011	7.607	1.00	0.00	H
ATOM	188	CA	VAL	N	8	1.842	14.254	7.709	1.00	0.00	C
ATOM	189	HG21	ILE	N	7	2.909	9.876	10.766	1.00	0.00	H
ATOM	190	HG22	ILE	N	7	2.056	11.203	9.985	1.00	0.00	H
ATOM	191	HG23	ILE	N	7	1.555	9.531	9.656	1.00	0.00	H
ATOM	192	HG11	ILE	N	7	4.743	8.724	7.663	1.00	0.00	H
ATOM	193	HG12	ILE	N	7	3.292	7.974	8.320	1.00	0.00	H
ATOM	194	CD1	ILE	N	7	4.876	8.325	9.766	1.00	0.00	C

```
ATOM    195  HA  THR N  30      -0.740  18.152   0.510  1.00  0.00           H
ATOM    196  N   THR N  30      -1.967  17.834  -1.133  1.00  0.00           N
ATOM    197  CB  THR N  30      -1.768  16.253   0.782  1.00  0.00           C
ATOM    198  HA  ILE N  34      -4.852   7.380  -5.299  1.00  0.00           H
ATOM    199  C   ILE N  34      -3.966   6.923  -7.215  1.00  0.00           C
ATOM    200  CB  ILE N  34      -5.974   8.340  -6.910  1.00  0.00           C
ATOM    201 HD11 ILE N  33       1.966   8.080  -5.690  1.00  0.00           H
ATOM    202 HD12 ILE N  33       0.757   7.356  -6.790  1.00  0.00           H
ATOM    203 HD13 ILE N  33       1.255   6.510  -5.302  1.00  0.00           H
ATOM    204  HA1 GLY N  37      -3.258  -0.967  -7.806  1.00  0.00           H
ATOM    205  HA2 GLY N  37      -4.950  -1.540  -7.851  1.00  0.00           H
ATOM    206  N   GLY N  37      -4.657   0.445  -8.410  1.00  0.00           N
ATOM    207  HA  ASP N  43       8.591   6.521  -0.997  1.00  0.00           H
ATOM    208  CB  ASP N  43       8.797   6.931  -3.078  1.00  0.00           C
ATOM    209  C   ASP N  43       6.660   6.985  -1.651  1.00  0.00           C
ATOM    210  HA  VAL N   8       0.988  14.045   8.366  1.00  0.00           H
ATOM    211  C   VAL N   8       1.161  14.658   6.399  1.00  0.00           C
ATOM    212  CB  VAL N   8       2.688  15.352   8.395  1.00  0.00           C
ATOM    213 HD11 ILE N   7       5.485   7.432   9.593  1.00  0.00           H
ATOM    214 HD12 ILE N   7       5.554   9.137  10.060  1.00  0.00           H
ATOM    215 HD13 ILE N   7       4.233   8.115  10.631  1.00  0.00           H
ATOM    216  H   THR N  30      -2.764  17.265  -1.418  1.00  0.00           H
ATOM    217  C   TYR N  29      -1.931  19.143  -1.521  1.00  0.00           C
ATOM    218  HB  THR N  30      -1.025  15.920   1.515  1.00  0.00           H
ATOM    219  OG1 THR N  30      -2.093  15.082   0.014  1.00  0.00           O
ATOM    220  CG2 THR N  30      -2.962  16.813   1.537  1.00  0.00           C
ATOM    221  O   ILE N  34      -3.439   7.348  -8.272  1.00  0.00           O
ATOM    222  N   ILE N  35      -4.076   5.628  -6.918  1.00  0.00           N
ATOM    223  HB  ILE N  34      -5.696   8.781  -7.880  1.00  0.00           H
ATOM    224  CG1 ILE N  34      -6.712   9.425  -6.109  1.00  0.00           C
ATOM    225  CG2 ILE N  34      -6.902   7.140  -7.162  1.00  0.00           C
ATOM    226  H   GLY N  37      -3.894   1.057  -8.712  1.00  0.00           H
ATOM    227  C   PRO N  36      -5.947   0.722  -8.706  1.00  0.00           C
ATOM    228  HB1 ASP N  43       8.832   8.024  -2.981  1.00  0.00           H
ATOM    229  HB2 ASP N  43       9.845   6.600  -3.012  1.00  0.00           H
ATOM    230  CG  ASP N  43       8.287   6.619  -4.491  1.00  0.00           C
ATOM    231  O   ASP N  43       6.610   8.212  -1.434  1.00  0.00           O
ATOM    232  N   TYR N  44       5.578   6.195  -1.608  1.00  0.00           N
ATOM    233  O   VAL N   8       0.096  15.290   6.456  1.00  0.00           O
ATOM    234  N   ALA N   9       1.682  14.253   5.226  1.00  0.00           N
ATOM    235  HB  VAL N   8       3.710  15.279   7.985  1.00  0.00           H
ATOM    236  CG1 VAL N   8       2.192  16.774   8.116  1.00  0.00           C
ATOM    237  CG2 VAL N   8       2.724  15.040   9.898  1.00  0.00           C
ATOM    238  O   TYR N  29      -1.020  19.905  -1.220  1.00  0.00           O
ATOM    239  CA  TYR N  29      -3.167  19.609  -2.319  1.00  0.00           C
ATOM    240  HG1 THR N  30      -2.898  15.278  -0.517  1.00  0.00           H
ATOM    241 HG21 THR N  30      -3.291  16.082   2.277  1.00  0.00           H
ATOM    242 HG22 THR N  30      -3.803  17.053   0.872  1.00  0.00           H
ATOM    243 HG23 THR N  30      -2.662  17.721   2.080  1.00  0.00           H
ATOM    244  H   ILE N  35      -4.547   5.369  -6.045  1.00  0.00           H
ATOM    245  CA  ILE N  35      -3.750   4.570  -7.857  1.00  0.00           C
ATOM    246 HG11 ILE N  34      -5.988  10.089  -5.613  1.00  0.00           H
ATOM    247 HG12 ILE N  34      -7.308   8.952  -5.311  1.00  0.00           H
ATOM    248  CD1 ILE N  34      -7.613  10.260  -7.012  1.00  0.00           C
ATOM    249 HG21 ILE N  34      -7.865   7.480  -7.568  1.00  0.00           H
ATOM    250 HG22 ILE N  34      -7.114   6.606  -6.225  1.00  0.00           H
ATOM    251 HG23 ILE N  34      -6.483   6.402  -7.864  1.00  0.00           H
ATOM    252  O   PRO N  36      -6.884  -0.044  -8.480  1.00  0.00           O
ATOM    253  CA  PRO N  36      -6.239   2.078  -9.379  1.00  0.00           C
ATOM    254  OD2 ASP N  43       7.283   5.863  -4.631  1.00  0.00           O
ATOM    255  OD1 ASP N  43       8.940   7.166  -5.429  1.00  0.00           O
ATOM    256  H   TYR N  44       5.689   5.188  -1.744  1.00  0.00           H
ATOM    257  CA  TYR N  44       4.260   6.646  -1.176  1.00  0.00           C
```

ATOM	258	H	ALA	N	9	2.572	13.755	5.179	1.00	0.00	H
ATOM	259	CA	ALA	N	9	0.878	14.396	4.014	1.00	0.00	C
ATOM	260	HG11	VAL	N	8	2.813	17.499	8.658	1.00	0.00	H
ATOM	261	HG12	VAL	N	8	2.252	17.028	7.048	1.00	0.00	H
ATOM	262	HG13	VAL	N	8	1.146	16.926	8.424	1.00	0.00	H
ATOM	263	HG21	VAL	N	8	3.291	15.802	10.443	1.00	0.00	H
ATOM	264	HG22	VAL	N	8	3.189	14.063	10.102	1.00	0.00	H
ATOM	265	HG23	VAL	N	8	1.707	15.020	10.318	1.00	0.00	H
ATOM	266	HA	TYR	N	29	-2.913	20.618	-2.673	1.00	0.00	H
ATOM	267	N	TYR	N	29	-3.413	18.734	-3.464	1.00	0.00	N
ATOM	268	CB	TYR	N	29	-4.430	19.663	-1.425	1.00	0.00	C
ATOM	269	HA	ILE	N	35	-3.516	5.062	-8.807	1.00	0.00	H
ATOM	270	C	ILE	N	35	-4.994	3.686	-8.026	1.00	0.00	C
ATOM	271	CB	ILE	N	35	-2.496	3.752	-7.430	1.00	0.00	C
ATOM	272	HD11	ILE	N	34	-8.249	10.922	-6.422	1.00	0.00	H
ATOM	273	HD12	ILE	N	34	-7.011	10.871	-7.702	1.00	0.00	H
ATOM	274	HD13	ILE	N	34	-8.295	9.642	-7.615	1.00	0.00	H
ATOM	275	HA	PRO	N	36	-7.153	2.431	-8.885	1.00	0.00	H
ATOM	276	N	PRO	N	36	-5.161	3.063	-9.213	1.00	0.00	N
ATOM	277	CB	PRO	N	36	-6.412	1.980	-10.907	1.00	0.00	C
ATOM	278	HA	TYR	N	44	4.409	7.602	-0.659	1.00	0.00	H
ATOM	279	C	TYR	N	44	3.745	5.643	-0.138	1.00	0.00	C
ATOM	280	CB	TYR	N	44	3.267	6.867	-2.324	1.00	0.00	C
ATOM	281	HA	ALA	N	9	0.558	15.440	3.918	1.00	0.00	H
ATOM	282	CB	ALA	N	9	1.660	14.018	2.758	1.00	0.00	C
ATOM	283	C	ALA	N	9	-0.436	13.607	4.171	1.00	0.00	C
ATOM	284	H	TYR	N	29	-4.321	18.268	-3.540	1.00	0.00	H
ATOM	285	C	THR	N	28	-2.584	18.733	-4.528	1.00	0.00	C
ATOM	286	HB1	TYR	N	29	-4.586	18.678	-0.963	1.00	0.00	H
ATOM	287	HB2	TYR	N	29	-5.297	19.818	-2.084	1.00	0.00	H
ATOM	288	CG	TYR	N	29	-4.393	20.739	-0.365	1.00	0.00	C
ATOM	289	O	ILE	N	35	-5.793	3.512	-7.091	1.00	0.00	O
ATOM	290	HB	ILE	N	35	-2.386	2.968	-8.193	1.00	0.00	H
ATOM	291	CG2	ILE	N	35	-1.251	4.647	-7.492	1.00	0.00	C
ATOM	292	CG1	ILE	N	35	-2.684	3.061	-6.063	1.00	0.00	C
ATOM	293	CD	PRO	N	36	-4.493	3.406	-10.490	1.00	0.00	C
ATOM	294	HB1	PRO	N	36	-6.745	0.977	-11.206	1.00	0.00	H
ATOM	295	HB2	PRO	N	36	-7.180	2.702	-11.230	1.00	0.00	H
ATOM	296	CG	PRO	N	36	-5.032	2.356	-11.468	1.00	0.00	C
ATOM	297	O	TYR	N	44	2.682	5.015	-0.291	1.00	0.00	O
ATOM	298	N	ALA	N	45	4.564	5.490	0.913	1.00	0.00	N
ATOM	299	HB1	TYR	N	44	2.296	7.086	-1.864	1.00	0.00	H
ATOM	300	HB2	TYR	N	44	3.150	5.930	-2.886	1.00	0.00	H
ATOM	301	CG	TYR	N	44	3.669	8.014	-3.211	1.00	0.00	C
ATOM	302	HB1	ALA	N	9	2.510	14.699	2.618	1.00	0.00	H
ATOM	303	HB2	ALA	N	9	1.010	14.109	1.877	1.00	0.00	H
ATOM	304	HB3	ALA	N	9	2.050	12.993	2.816	1.00	0.00	H
ATOM	305	O	ALA	N	9	-1.509	14.115	3.816	1.00	0.00	O
ATOM	306	N	ARG	N	10	-0.365	12.374	4.723	1.00	0.00	N
ATOM	307	O	THR	N	28	-1.608	19.488	-4.627	1.00	0.00	O
ATOM	308	CA	THR	N	28	-2.852	17.722	-5.657	1.00	0.00	C
ATOM	309	CD1	TYR	N	29	-4.596	22.083	-0.704	1.00	0.00	C
ATOM	310	CD2	TYR	N	29	-4.154	20.444	0.983	1.00	0.00	C
ATOM	311	HG21	ILE	N	35	-0.341	4.079	-7.248	1.00	0.00	H
ATOM	312	HG22	ILE	N	35	-1.106	5.070	-8.498	1.00	0.00	H
ATOM	313	HG23	ILE	N	35	-1.313	5.488	-6.783	1.00	0.00	H
ATOM	314	HG11	ILE	N	35	-3.658	2.552	-6.053	1.00	0.00	H
ATOM	315	HG12	ILE	N	35	-2.722	3.825	-5.267	1.00	0.00	H
ATOM	316	CD1	ILE	N	35	-1.604	2.028	-5.731	1.00	0.00	C
ATOM	317	HD1	PRO	N	36	-3.407	3.333	-10.387	1.00	0.00	H
ATOM	318	HD2	PRO	N	36	-4.771	4.432	-10.782	1.00	0.00	H
ATOM	319	HG1	PRO	N	36	-4.380	1.473	-11.479	1.00	0.00	H
ATOM	320	HG2	PRO	N	36	-5.080	2.750	-12.493	1.00	0.00	H

ATOM	321	H	ALA	N	45	5.333	6.170	1.057	1.00	0.00	H
ATOM	322	CA	ALA	N	45	4.414	4.427	1.884	1.00	0.00	C
ATOM	323	CD2	TYR	N	44	3.017	9.244	-3.099	1.00	0.00	C
ATOM	324	CD1	TYR	N	44	4.750	7.918	-4.100	1.00	0.00	C
ATOM	325	H	ARG	N	10	0.548	11.991	4.971	1.00	0.00	H
ATOM	326	CA	ARG	N	10	-1.562	11.627	5.095	1.00	0.00	C
ATOM	327	HA	THR	N	28	-1.844	17.409	-5.959	1.00	0.00	H
ATOM	328	N	THR	N	28	-3.579	16.544	-5.188	1.00	0.00	N
ATOM	329	CB	THR	N	28	-3.511	18.393	-6.873	1.00	0.00	C
ATOM	330	HD1	TYR	N	29	-4.776	22.357	-1.744	1.00	0.00	H
ATOM	331	CE1	TYR	N	29	-4.564	23.092	0.253	1.00	0.00	C
ATOM	332	HD2	TYR	N	29	-3.992	19.408	1.282	1.00	0.00	H
ATOM	333	CE2	TYR	N	29	-4.115	21.442	1.956	1.00	0.00	C
ATOM	334	HD11	ILE	N	35	-0.600	2.474	-5.658	1.00	0.00	H
ATOM	335	HD12	ILE	N	35	-1.550	1.233	-6.491	1.00	0.00	H
ATOM	336	HD13	ILE	N	35	-1.807	1.547	-4.760	1.00	0.00	H
ATOM	337	HA	ALA	N	45	4.014	3.551	1.352	1.00	0.00	H
ATOM	338	C	ALA	N	45	3.403	4.750	2.986	1.00	0.00	C
ATOM	339	CB	ALA	N	45	5.786	4.103	2.495	1.00	0.00	C
ATOM	340	HD2	TYR	N	44	2.167	9.341	-2.419	1.00	0.00	H
ATOM	341	CE2	TYR	N	44	3.410	10.353	-3.848	1.00	0.00	C
ATOM	342	HD1	TYR	N	44	5.304	6.988	-4.213	1.00	0.00	H
ATOM	343	CE1	TYR	N	44	5.169	9.019	-4.836	1.00	0.00	C
ATOM	344	HA	ARG	N	10	-2.195	11.530	4.206	1.00	0.00	H
ATOM	345	C	ARG	N	10	-2.434	12.384	6.105	1.00	0.00	C
ATOM	346	CB	ARG	N	10	-1.189	10.228	5.619	1.00	0.00	C
ATOM	347	H	THR	N	28	-4.569	16.433	-5.421	1.00	0.00	H
ATOM	348	C	ALA	N	27	-2.962	15.703	-4.337	1.00	0.00	C
ATOM	349	HB	THR	N	28	-2.907	19.300	-7.062	1.00	0.00	H
ATOM	350	OG1	THR	N	28	-4.834	18.757	-6.496	1.00	0.00	O
ATOM	351	CG2	THR	N	28	-3.480	17.526	-8.128	1.00	0.00	C
ATOM	352	HE1	TYR	N	29	-4.710	24.130	-0.038	1.00	0.00	H
ATOM	353	CZ	TYR	N	29	-4.315	22.782	1.599	1.00	0.00	C
ATOM	354	HE2	TYR	N	29	-3.919	21.200	3.002	1.00	0.00	H
ATOM	355	O	ALA	N	45	3.039	3.850	3.778	1.00	0.00	O
ATOM	356	N	ASN	N	46	2.986	6.007	3.080	1.00	0.00	N
ATOM	357	HB1	ALA	N	45	5.720	3.248	3.177	1.00	0.00	H
ATOM	358	HB2	ALA	N	45	6.506	3.875	1.698	1.00	0.00	H
ATOM	359	HB3	ALA	N	45	6.171	4.968	3.055	1.00	0.00	H
ATOM	360	HE2	TYR	N	44	2.874	11.292	-3.742	1.00	0.00	H
ATOM	361	CZ	TYR	N	44	4.487	10.238	-4.731	1.00	0.00	C
ATOM	362	HE1	TYR	N	44	6.036	8.939	-5.490	1.00	0.00	H
ATOM	363	O	ARG	N	10	-3.672	12.383	5.963	1.00	0.00	O
ATOM	364	N	SER	N	11	-1.823	13.017	7.117	1.00	0.00	N
ATOM	365	HB1	ARG	N	10	-0.521	10.348	6.481	1.00	0.00	H
ATOM	366	HB2	ARG	N	10	-0.606	9.714	4.844	1.00	0.00	H
ATOM	367	CG	ARG	N	10	-2.387	9.376	6.054	1.00	0.00	C
ATOM	368	O	ALA	N	27	-1.766	15.853	-4.029	1.00	0.00	O
ATOM	369	CA	ALA	N	27	-3.752	14.511	-3.766	1.00	0.00	C
ATOM	370	HG1	THR	N	28	-5.131	19.467	-7.115	1.00	0.00	H
ATOM	371	HG21	THR	N	28	-2.451	17.342	-8.466	1.00	0.00	H
ATOM	372	HG22	THR	N	28	-3.986	18.058	-8.945	1.00	0.00	H
ATOM	373	HG23	THR	N	28	-4.002	16.573	-7.973	1.00	0.00	H
ATOM	374	OH	TYR	N	29	-4.238	23.733	2.564	1.00	0.00	O
ATOM	375	H	ASN	N	46	3.224	6.689	2.355	1.00	0.00	H
ATOM	376	CA	ASN	N	46	2.187	6.486	4.188	1.00	0.00	C
ATOM	377	OH	TYR	N	44	4.912	11.262	-5.521	1.00	0.00	O
ATOM	378	H	SER	N	11	-0.833	12.818	7.299	1.00	0.00	H
ATOM	379	CA	SER	N	11	-2.612	13.751	8.094	1.00	0.00	C
ATOM	380	HG1	ARG	N	10	-2.963	9.916	6.817	1.00	0.00	H
ATOM	381	HG2	ARG	N	10	-2.004	8.479	6.565	1.00	0.00	H
ATOM	382	CD	ARG	N	10	-3.355	8.952	4.953	1.00	0.00	C
ATOM	383	HA	ALA	N	27	-3.106	14.130	-2.966	1.00	0.00	H

ATOM	384	N	ALA	N	27	-5.037	14.924	-3.208	1.00	0.00	N
ATOM	385	CB	ALA	N	27	-3.980	13.432	-4.829	1.00	0.00	C
ATOM	386	HH	TYR	N	29	-4.426	24.633	2.172	1.00	0.00	H
ATOM	387	HA	ASN	N	46	2.167	5.687	4.936	1.00	0.00	H
ATOM	388	CB	ASN	N	46	2.873	7.713	4.815	1.00	0.00	C
ATOM	389	C	ASN	N	46	0.717	6.734	3.795	1.00	0.00	C
ATOM	390	HH	TYR	N	44	4.419	12.101	-5.333	1.00	0.00	H
ATOM	391	HA	SER	N	11	-3.360	13.077	8.522	1.00	0.00	H
ATOM	392	CB	SER	N	11	-1.759	14.339	9.221	1.00	0.00	C
ATOM	393	C	SER	N	11	-3.389	14.885	7.404	1.00	0.00	C
ATOM	394	HD1	ARG	N	10	-4.258	8.530	5.407	1.00	0.00	H
ATOM	395	HD2	ARG	N	10	-3.682	9.813	4.348	1.00	0.00	H
ATOM	396	NE	ARG	N	10	-2.743	7.950	4.075	1.00	0.00	N
ATOM	397	H	ALA	N	27	-5.883	14.557	-3.654	1.00	0.00	H
ATOM	398	C	CYS	N	26	-5.176	15.649	-2.100	1.00	0.00	C
ATOM	399	HB1	ALA	N	27	-4.764	13.743	-5.522	1.00	0.00	H
ATOM	400	HB2	ALA	N	27	-3.055	13.255	-5.386	1.00	0.00	H
ATOM	401	HB3	ALA	N	27	-4.288	12.490	-4.353	1.00	0.00	H
ATOM	402	HB1	ASN	N	46	2.783	8.578	4.151	1.00	0.00	H
ATOM	403	HB2	ASN	N	46	2.382	7.957	5.766	1.00	0.00	H
ATOM	404	CG	ASN	N	46	4.361	7.444	5.021	1.00	0.00	C
ATOM	405	OXT	ASN	N	46	0.008	7.360	4.630	1.00	0.00	O
ATOM	406	O	ASN	N	46	0.302	6.296	2.681	1.00	0.00	O
ATOM	407	HB1	SER	N	11	-2.449	14.869	9.900	1.00	0.00	H
ATOM	408	HB2	SER	N	11	-1.056	15.080	8.803	1.00	0.00	H
ATOM	409	OG	SER	N	11	-1.075	13.305	9.904	1.00	0.00	O
ATOM	410	O	SER	N	11	-4.611	15.066	7.616	1.00	0.00	O
ATOM	411	N	ASN	N	12	-2.673	15.657	6.591	1.00	0.00	N
ATOM	412	HE	ARG	N	10	-1.767	7.674	4.267	1.00	0.00	H
ATOM	413	CZ	ARG	N	10	-3.288	7.481	2.948	1.00	0.00	C
ATOM	414	O	CYS	N	26	-4.225	15.989	-1.367	1.00	0.00	O
ATOM	415	CA	CYS	N	26	-6.587	16.137	-1.733	1.00	0.00	C
ATOM	416	OD1	ASN	N	46	5.216	8.052	4.363	1.00	0.00	O
ATOM	417	ND2	ASN	N	46	4.688	6.490	5.914	1.00	0.00	N
ATOM	418	H	SER	N	11	-0.639	13.719	10.691	1.00	0.00	H
ATOM	419	H	ASN	N	12	-1.664	15.476	6.492	1.00	0.00	H
ATOM	420	CA	ASN	N	12	-3.253	16.786	5.880	1.00	0.00	C
ATOM	421	NH2	ARG	N	10	-4.572	7.728	2.654	1.00	0.00	N
ATOM	422	NH1	ARG	N	10	-2.507	6.805	2.086	1.00	0.00	N
ATOM	423	HA	CYS	N	26	-6.403	17.141	-1.322	1.00	0.00	H
ATOM	424	N	CYS	N	26	-7.439	16.256	-2.893	1.00	0.00	N
ATOM	425	CB	CYS	N	26	-7.238	15.288	-0.644	1.00	0.00	C
ATOM	426	HD21	ASN	N	46	5.677	6.316	6.066	1.00	0.00	H
ATOM	427	HD22	ASN	N	46	4.001	5.962	6.465	1.00	0.00	H
ATOM	428	HA	ASN	N	12	-3.753	17.446	6.600	1.00	0.00	H
ATOM	429	CB	ASN	N	12	-2.149	17.570	5.162	1.00	0.00	C
ATOM	430	C	ASN	N	12	-4.352	16.296	4.922	1.00	0.00	C
ATOM	431	HH21	ARG	N	10	-4.948	7.439	1.754	1.00	0.00	H
ATOM	432	HH22	ARG	N	10	-5.263	7.849	3.419	1.00	0.00	H
ATOM	433	HH11	ARG	N	10	-2.951	6.109	1.488	1.00	0.00	H
ATOM	434	HH12	ARG	N	10	-1.551	6.564	2.380	1.00	0.00	H
ATOM	435	H	CYS	N	26	-8.265	15.654	-2.988	1.00	0.00	H
ATOM	436	C	LEU	N	25	-7.166	17.207	-3.808	1.00	0.00	C
ATOM	437	HB1	CYS	N	26	-6.549	15.157	0.199	1.00	0.00	H
ATOM	438	HB2	CYS	N	26	-7.507	14.288	-1.017	1.00	0.00	H
ATOM	439	SG	CYS	N	26	-8.824	15.999	-0.041	1.00	0.00	S
ATOM	440	HB1	ASN	N	12	-1.598	16.930	4.457	1.00	0.00	H
ATOM	441	HB2	ASN	N	12	-2.618	18.370	4.566	1.00	0.00	H
ATOM	442	CG	ASN	N	12	-1.173	18.222	6.164	1.00	0.00	C
ATOM	443	O	ASN	N	12	-5.412	16.928	4.786	1.00	0.00	O
ATOM	444	N	PHE	N	13	-4.086	15.147	4.276	1.00	0.00	N
ATOM	445	O	LEU	N	25	-6.247	18.023	-3.664	1.00	0.00	O
ATOM	446	CA	LEU	N	25	-8.055	17.241	-5.051	1.00	0.00	C

```
ATOM    447  SG  CYS N  16      -8.233  17.870   0.666  1.00  0.00           S
ATOM    448  OD1 ASN N  12      -1.437  18.284   7.364  1.00  0.00           O
ATOM    449  ND2 ASN N  12      -0.054  18.735   5.611  1.00  0.00           N
ATOM    450  H   PHE N  13      -3.154  14.725   4.356  1.00  0.00           H
ATOM    451  CA  PHE N  13      -5.030  14.496   3.384  1.00  0.00           C
ATOM    452  HA  LEU N  25      -7.437  17.732  -5.812  1.00  0.00           H
ATOM    453  N   LEU N  25      -8.385  15.894  -5.520  1.00  0.00           N
ATOM    454  CB  LEU N  25      -9.341  18.037  -4.769  1.00  0.00           C
ATOM    455  CB  CYS N  16      -7.726  17.511   2.388  1.00  0.00           C
ATOM    456 HD21 ASN N  12       0.160  18.589   4.627  1.00  0.00           H
ATOM    457 HD22 ASN N  12       0.654  19.136   6.226  1.00  0.00           H
ATOM    458  HA  PHE N  13      -5.355  15.198   2.601  1.00  0.00           H
ATOM    459  C   PHE N  13      -6.316  14.106   4.114  1.00  0.00           C
ATOM    460  CB  PHE N  13      -4.330  13.279   2.745  1.00  0.00           C
ATOM    461  H   LEU N  25      -9.241  15.472  -5.156  1.00  0.00           H
ATOM    462  C   ALA N  24      -7.446  15.056  -6.000  1.00  0.00           C
ATOM    463  HB1 LEU N  25     -10.014  17.388  -4.187  1.00  0.00           H
ATOM    464  HB2 LEU N  25      -9.088  18.885  -4.110  1.00  0.00           H
ATOM    465  CG  LEU N  25     -10.059  18.554  -6.026  1.00  0.00           C
ATOM    466  HB1 CYS N  16      -7.274  16.511   2.420  1.00  0.00           H
ATOM    467  HB2 CYS N  16      -6.941  18.243   2.629  1.00  0.00           H
ATOM    468  CA  CYS N  16      -8.845  17.605   3.438  1.00  0.00           C
ATOM    469  O   PHE N  13      -7.419  14.385   3.621  1.00  0.00           O
ATOM    470  N   ASN N  14      -6.184  13.446   5.279  1.00  0.00           N
ATOM    471  HB1 PHE N  13      -3.514  13.655   2.116  1.00  0.00           H
ATOM    472  HB2 PHE N  13      -3.866  12.704   3.555  1.00  0.00           H
ATOM    473  CG  PHE N  13      -5.235  12.379   1.949  1.00  0.00           C
ATOM    474  O   ALA N  24      -6.270  15.412  -6.188  1.00  0.00           O
ATOM    475  CA  ALA N  24      -7.901  13.634  -6.379  1.00  0.00           C
ATOM    476  HG  LEU N  25     -10.101  17.719  -6.739  1.00  0.00           H
ATOM    477  CD1 LEU N  25      -9.312  19.703  -6.699  1.00  0.00           C
ATOM    478  CD2 LEU N  25     -11.502  18.955  -5.700  1.00  0.00           C
ATOM    479  HA  CYS N  16      -9.220  18.636   3.494  1.00  0.00           H
ATOM    480  N   CYS N  16      -8.261  17.203   4.711  1.00  0.00           N
ATOM    481  C   CYS N  16     -10.093  16.760   3.097  1.00  0.00           C
ATOM    482  H   ASN N  14      -5.249  13.195   5.627  1.00  0.00           H
ATOM    483  CA  ASN N  14      -7.372  13.013   5.992  1.00  0.00           C
ATOM    484  CD1 PHE N  13      -5.420  12.578   0.575  1.00  0.00           C
ATOM    485  CD2 PHE N  13      -5.907  11.317   2.574  1.00  0.00           C
ATOM    486  HA  ALA N  24      -6.978  13.044  -6.436  1.00  0.00           H
ATOM    487  N   ALA N  24      -8.771  13.075  -5.350  1.00  0.00           N
ATOM    488  CB  ALA N  24      -8.587  13.631  -7.744  1.00  0.00           C
ATOM    489 HD11 LEU N  25      -8.277  19.443  -6.962  1.00  0.00           H
ATOM    490 HD12 LEU N  25      -9.811  20.004  -7.631  1.00  0.00           H
ATOM    491 HD13 LEU N  25      -9.280  20.590  -6.047  1.00  0.00           H
ATOM    492 HD21 LEU N  25     -12.094  18.091  -5.371  1.00  0.00           H
ATOM    493 HD22 LEU N  25     -12.009  19.381  -6.580  1.00  0.00           H
ATOM    494 HD23 LEU N  25     -11.538  19.709  -4.894  1.00  0.00           H
ATOM    495  H   CYS N  16      -7.265  16.954   4.735  1.00  0.00           H
ATOM    496  C   VAL N  15      -9.003  17.113   5.829  1.00  0.00           C
ATOM    497  O   CYS N  16     -11.099  17.276   2.580  1.00  0.00           O
ATOM    498  N   ARG N  17     -10.034  15.461   3.420  1.00  0.00           N
ATOM    499  HA  ASN N  14      -8.023  12.466   5.297  1.00  0.00           H
ATOM    500  CB  ASN N  14      -7.042  12.096   7.172  1.00  0.00           C
ATOM    501  C   ASN N  14      -8.219  14.208   6.453  1.00  0.00           C
ATOM    502  HD1 PHE N  13      -4.884  13.392   0.082  1.00  0.00           H
ATOM    503  CE1 PHE N  13      -6.271  11.749  -0.160  1.00  0.00           C
ATOM    504  HD2 PHE N  13      -5.777  11.144   3.643  1.00  0.00           H
ATOM    505  CE2 PHE N  13      -6.746  10.480   1.843  1.00  0.00           C
ATOM    506  H   ALA N  24      -9.610  12.551  -5.640  1.00  0.00           H
ATOM    507  C   GLU N  23      -8.290  12.896  -4.100  1.00  0.00           C
ATOM    508  HB1 ALA N  24      -7.907  14.046  -8.496  1.00  0.00           H
ATOM    509  HB2 ALA N  24      -9.503  14.232  -7.735  1.00  0.00           H
```

ATOM	510	HB3	ALA	N	24	-8.856	12.609	-8.040	1.00	0.00	H
ATOM	511	O	VAL	N	15	-10.198	17.443	5.864	1.00	0.00	O
ATOM	512	CA	VAL	N	15	-8.308	16.569	7.081	1.00	0.00	C
ATOM	513	H	ARG	N	17	-9.140	15.071	3.737	1.00	0.00	H
ATOM	514	CA	ARG	N	17	-11.127	14.541	3.168	1.00	0.00	C
ATOM	515	HB1	ASN	N	14	-6.360	12.573	7.888	1.00	0.00	H
ATOM	516	HB2	ASN	N	14	-7.986	11.903	7.707	1.00	0.00	H
ATOM	517	CG	ASN	N	14	-6.535	10.725	6.750	1.00	0.00	C
ATOM	518	O	ASN	N	14	-9.458	14.111	6.491	1.00	0.00	O
ATOM	519	N	VAL	N	15	-7.576	15.336	6.798	1.00	0.00	N
ATOM	520	HE1	PHE	N	13	-6.402	11.920	-1.232	1.00	0.00	H
ATOM	521	CZ	PHE	N	13	-6.937	10.699	0.478	1.00	0.00	C
ATOM	522	HE2	PHE	N	13	-7.259	9.655	2.340	1.00	0.00	H
ATOM	523	O	GLU	N	23	-7.140	13.222	-3.773	1.00	0.00	O
ATOM	524	CA	GLU	N	23	-9.216	12.270	-3.056	1.00	0.00	C
ATOM	525	HA	VAL	N	15	-9.137	16.331	7.759	1.00	0.00	H
ATOM	526	CB	VAL	N	15	-7.387	17.607	7.759	1.00	0.00	C
ATOM	527	HA	ARG	N	17	-11.563	14.762	2.180	1.00	0.00	H
ATOM	528	C	ARG	N	17	-12.301	14.698	4.131	1.00	0.00	C
ATOM	529	CB	ARG	N	17	-10.605	13.094	3.213	1.00	0.00	C
ATOM	530	OD1	ASN	N	14	-6.986	10.175	5.719	1.00	0.00	O
ATOM	531	ND2	ASN	N	14	-5.666	10.124	7.561	1.00	0.00	N
ATOM	532	H	VAL	N	15	-6.554	15.358	6.770	1.00	0.00	H
ATOM	533	HZ	PHE	N	13	-7.591	10.033	-0.078	1.00	0.00	H
ATOM	534	HA	GLU	N	23	-8.816	12.634	-2.097	1.00	0.00	H
ATOM	535	N	GLU	N	23	-10.584	12.717	-3.208	1.00	0.00	N
ATOM	536	CB	GLU	N	23	-9.095	10.740	-3.100	1.00	0.00	C
ATOM	537	HB	VAL	N	15	-6.429	17.606	7.211	1.00	0.00	H
ATOM	538	CG2	VAL	N	15	-7.142	17.167	9.200	1.00	0.00	C
ATOM	539	CG1	VAL	N	15	-7.968	19.021	7.683	1.00	0.00	C
ATOM	540	O	ARG	N	17	-13.397	14.177	3.818	1.00	0.00	O
ATOM	541	N	LEU	N	18	-12.155	15.365	5.278	1.00	0.00	N
ATOM	542	HB1	ARG	N	17	-10.043	12.965	4.144	1.00	0.00	H
ATOM	543	HB2	ARG	N	17	-11.461	12.408	3.274	1.00	0.00	H
ATOM	544	CG	ARG	N	17	-9.722	12.725	2.020	1.00	0.00	C
ATOM	545	HD21	ASN	N	14	-5.413	9.154	7.366	1.00	0.00	H
ATOM	546	HD22	ASN	N	14	-5.221	10.582	8.375	1.00	0.00	H
ATOM	547	H	GLU	N	23	-11.317	12.009	-3.457	1.00	0.00	H
ATOM	548	C	PRO	N	22	-10.874	14.021	-3.295	1.00	0.00	C
ATOM	549	HB1	GLU	N	23	-9.497	10.370	-4.050	1.00	0.00	H
ATOM	550	HB2	GLU	N	23	-8.020	10.514	-3.108	1.00	0.00	H
ATOM	551	CG	GLU	N	23	-9.780	10.027	-1.924	1.00	0.00	C
ATOM	552	HG21	VAL	N	15	-6.499	17.866	9.744	1.00	0.00	H
ATOM	553	HG22	VAL	N	15	-6.655	16.187	9.243	1.00	0.00	H
ATOM	554	HG23	VAL	N	15	-8.099	17.091	9.741	1.00	0.00	H
ATOM	555	HG11	VAL	N	15	-7.311	19.722	8.210	1.00	0.00	H
ATOM	556	HG12	VAL	N	15	-8.056	19.367	6.643	1.00	0.00	H
ATOM	557	HG13	VAL	N	15	-8.967	19.081	8.140	1.00	0.00	H
ATOM	558	H	LEU	N	18	-11.278	15.846	5.495	1.00	0.00	H
ATOM	559	CA	LEU	N	18	-13.256	15.461	6.230	1.00	0.00	C
ATOM	560	HG1	ARG	N	17	-9.180	11.802	2.265	1.00	0.00	H
ATOM	561	HG2	ARG	N	17	-8.949	13.488	1.856	1.00	0.00	H
ATOM	562	CD	ARG	N	17	-10.497	12.542	0.720	1.00	0.00	C
ATOM	563	O	PRO	N	22	-10.024	14.930	-3.199	1.00	0.00	O
ATOM	564	CA	PRO	N	22	-12.314	14.322	-3.674	1.00	0.00	C
ATOM	565	HG1	GLU	N	23	-9.553	10.537	-0.982	1.00	0.00	H
ATOM	566	HG2	GLU	N	23	-10.870	10.060	-2.068	1.00	0.00	H
ATOM	567	CD	GLU	N	23	-9.385	8.556	-1.794	1.00	0.00	C
ATOM	568	HA	LEU	N	18	-13.598	14.441	6.455	1.00	0.00	H
ATOM	569	CB	LEU	N	18	-12.826	16.158	7.521	1.00	0.00	C
ATOM	570	C	LEU	N	18	-14.507	16.125	5.651	1.00	0.00	C
ATOM	571	HD1	ARG	N	17	-11.104	13.428	0.502	1.00	0.00	H
ATOM	572	HD2	ARG	N	17	-9.786	12.445	-0.119	1.00	0.00	H

ATOM	573	NE	ARG	N	17	-11.353	11.357	0.791	1.00	0.00	N
ATOM	574	HA	PRO	N	22	-12.948	13.447	-3.475	1.00	0.00	H
ATOM	575	N	PRO	N	22	-12.848	15.490	-2.966	1.00	0.00	N
ATOM	576	CB	PRO	N	22	-12.429	14.728	-5.149	1.00	0.00	C
ATOM	577	OE1	GLU	N	23	-9.436	7.852	-2.839	1.00	0.00	O
ATOM	578	OE2	GLU	N	23	-9.023	8.122	-0.649	1.00	0.00	O
ATOM	579	HB1	LEU	N	18	-12.321	17.102	7.268	1.00	0.00	H
ATOM	580	HB2	LEU	N	18	-13.738	16.427	8.074	1.00	0.00	H
ATOM	581	CG	LEU	N	18	-11.911	15.339	8.445	1.00	0.00	C
ATOM	582	O	LEU	N	18	-15.626	15.630	5.919	1.00	0.00	O
ATOM	583	N	PRO	N	19	-14.411	17.209	4.864	1.00	0.00	N
ATOM	584	HE	ARG	N	17	-10.941	10.544	1.270	1.00	0.00	H
ATOM	585	CZ	ARG	N	17	-12.382	11.092	-0.033	1.00	0.00	C
ATOM	586	C	THR	N	21	-12.707	15.586	-1.639	1.00	0.00	C
ATOM	587	CD	PRO	N	22	-13.694	16.330	-3.836	1.00	0.00	C
ATOM	588	HB1	PRO	N	22	-12.491	13.852	-5.804	1.00	0.00	H
ATOM	589	HB2	PRO	N	22	-11.556	15.339	-5.425	1.00	0.00	H
ATOM	590	CG	PRO	N	22	-13.705	15.568	-5.166	1.00	0.00	C
ATOM	591	HG	LEU	N	18	-10.988	15.100	7.895	1.00	0.00	H
ATOM	592	CD2	LEU	N	18	-11.548	16.249	9.620	1.00	0.00	C
ATOM	593	CD1	LEU	N	18	-12.547	14.018	8.895	1.00	0.00	C
ATOM	594	CA	PRO	N	19	-15.617	17.740	4.209	1.00	0.00	C
ATOM	595	CD	PRO	N	19	-13.288	18.189	4.811	1.00	0.00	C
ATOM	596	NH1	ARG	N	17	-12.936	12.036	-0.790	1.00	0.00	N
ATOM	597	NH2	ARG	N	17	-12.869	9.844	-0.084	1.00	0.00	N
ATOM	598	O	THR	N	21	-12.119	14.721	-0.967	1.00	0.00	O
ATOM	599	CA	THR	N	21	-13.202	16.847	-0.921	1.00	0.00	C
ATOM	600	HD1	PRO	N	22	-14.702	16.426	-3.413	1.00	0.00	H
ATOM	601	HD2	PRO	N	22	-13.255	17.334	-3.928	1.00	0.00	H
ATOM	602	HG1	PRO	N	22	-13.773	16.241	-6.025	1.00	0.00	H
ATOM	603	HG2	PRO	N	22	-14.573	14.903	-5.183	1.00	0.00	H
ATOM	604	HD21	LEU	N	18	-10.868	15.763	10.330	1.00	0.00	H
ATOM	605	HD22	LEU	N	18	-11.053	17.165	9.261	1.00	0.00	H
ATOM	606	HD23	LEU	N	18	-12.444	16.560	10.180	1.00	0.00	H
ATOM	607	HD11	LEU	N	18	-13.522	14.184	9.374	1.00	0.00	H
ATOM	608	HD12	LEU	N	18	-11.906	13.494	9.619	1.00	0.00	H
ATOM	609	HD13	LEU	N	18	-12.692	13.326	8.051	1.00	0.00	H
ATOM	610	HA	PRO	N	19	-16.512	17.533	4.810	1.00	0.00	H
ATOM	611	CB	PRO	N	19	-15.335	19.233	4.104	1.00	0.00	C
ATOM	612	C	PRO	N	19	-15.882	17.087	2.847	1.00	0.00	C
ATOM	613	HD1	PRO	N	19	-12.370	17.734	4.436	1.00	0.00	H
ATOM	614	HD2	PRO	N	19	-13.106	18.566	5.828	1.00	0.00	H
ATOM	615	CG	PRO	N	19	-13.825	19.293	3.899	1.00	0.00	C
ATOM	616	HH11	ARG	N	17	-12.590	13.001	-0.805	1.00	0.00	H
ATOM	617	HH12	ARG	N	17	-13.616	11.772	-1.516	1.00	0.00	H
ATOM	618	HH21	ARG	N	17	-12.364	9.045	0.309	1.00	0.00	H
ATOM	619	HH22	ARG	N	17	-13.674	9.637	-0.685	1.00	0.00	H
ATOM	620	HA	THR	N	21	-13.820	17.480	-1.573	1.00	0.00	H
ATOM	621	N	THR	N	21	-13.981	16.445	0.230	1.00	0.00	N
ATOM	622	CB	THR	N	21	-11.964	17.635	-0.441	1.00	0.00	C
ATOM	623	HB1	PRO	N	19	-15.907	19.725	3.319	1.00	0.00	H
ATOM	624	HB2	PRO	N	19	-15.600	19.707	5.050	1.00	0.00	H
ATOM	625	O	PRO	N	19	-16.666	17.641	2.047	1.00	0.00	O
ATOM	626	N	GLY	N	20	-15.279	15.920	2.583	1.00	0.00	N
ATOM	627	HG1	PRO	N	19	-13.419	20.279	4.151	1.00	0.00	H
ATOM	628	HG2	PRO	N	19	-13.572	19.080	2.853	1.00	0.00	H
ATOM	629	H	THR	N	21	-13.714	16.817	1.135	1.00	0.00	H
ATOM	630	C	GLY	N	20	-14.934	15.502	0.144	1.00	0.00	C
ATOM	631	HB	THR	N	21	-11.302	16.907	0.046	1.00	0.00	H
ATOM	632	OG1	THR	N	21	-12.369	18.584	0.549	1.00	0.00	O
ATOM	633	CG2	THR	N	21	-11.232	18.291	-1.600	1.00	0.00	C
ATOM	634	H	GLY	N	20	-14.628	15.500	3.251	1.00	0.00	H
ATOM	635	CA	GLY	N	20	-15.621	15.103	1.434	1.00	0.00	C

```
ATOM    636   O   GLY N  20     -15.233  14.957  -0.922  1.00  0.00           O
ATOM    637  HG1 THR N  21     -11.845  18.372   1.353  1.00  0.00           H
ATOM    638 HG21 THR N  21     -10.815  17.518  -2.258  1.00  0.00           H
ATOM    639 HG22 THR N  21     -10.402  18.902  -1.222  1.00  0.00           H
ATOM    640 HG23 THR N  21     -11.898  18.941  -2.186  1.00  0.00           H
ATOM    641  HA1 GLY N  20     -16.700  15.141   1.242  1.00  0.00           H
ATOM    642  HA2 GLY N  20     -15.327  14.067   1.647  1.00  0.00           H
END
```

4) CRAMBIN – molecule as in the optimized 172W crystal

```
COMPND    CRAMBIN OPTIMIZED CRYSTAL 172W GEOMETRY
AUTHOR    DELLE PIANE, CORNO, ORLANDO, DOVESI, UGLIENGO
ATOM      1  N   THR N   1      -6.981  -4.273  -2.053  1.00  0.00           N
ATOM      2  H1  THR N   1      -6.366  -5.104  -2.013  1.00  0.00           H
ATOM      3  H2  THR N   1      -7.626  -4.384  -2.862  1.00  0.00           H
ATOM      4  H3  THR N   1      -7.578  -4.181  -1.184  1.00  0.00           H
ATOM      5  CA  THR N   1      -6.209  -3.015  -2.242  1.00  0.00           C
ATOM      6  HA  THR N   1      -6.902  -2.179  -2.074  1.00  0.00           H
ATOM      7  C   THR N   1      -5.659  -3.012  -3.656  1.00  0.00           C
ATOM      8  CB  THR N   1      -5.097  -2.937  -1.169  1.00  0.00           C
ATOM      9  O   THR N   1      -5.157  -4.037  -4.136  1.00  0.00           O
ATOM     10  N   THR N   2      -5.741  -1.835  -4.296  1.00  0.00           N
ATOM     11  HB  THR N   1      -4.492  -3.855  -1.261  1.00  0.00           H
ATOM     12  OG1 THR N   1      -5.747  -2.931   0.090  1.00  0.00           O
ATOM     13  CG2 THR N   1      -4.187  -1.732  -1.308  1.00  0.00           C
ATOM     14  H   THR N   2      -6.101  -1.014  -3.798  1.00  0.00           H
ATOM     15  CA  THR N   2      -5.062  -1.600  -5.559  1.00  0.00           C
ATOM     16  HG1 THR N   1      -5.694  -3.860   0.443  1.00  0.00           H
ATOM     17 HG21 THR N   1      -4.742  -0.798  -1.179  1.00  0.00           H
ATOM     18 HG22 THR N   1      -3.696  -1.712  -2.287  1.00  0.00           H
ATOM     19 HG23 THR N   1      -3.410  -1.777  -0.535  1.00  0.00           H
ATOM     20  HA  THR N   2      -4.867  -2.578  -6.015  1.00  0.00           H
ATOM     21  C   THR N   2      -3.725  -0.926  -5.260  1.00  0.00           C
ATOM     22  CB  THR N   2      -5.940  -0.775  -6.535  1.00  0.00           C
ATOM     23  O   THR N   2      -3.636   0.012  -4.433  1.00  0.00           O
ATOM     24  N   CYS N   3      -2.679  -1.411  -5.929  1.00  0.00           N
ATOM     25  HB  THR N   2      -5.389  -0.716  -7.489  1.00  0.00           H
ATOM     26  OG1 THR N   2      -6.095   0.559  -6.075  1.00  0.00           O
ATOM     27  CG2 THR N   2      -7.272  -1.482  -6.757  1.00  0.00           C
ATOM     28  H   CYS N   3      -2.825  -2.235  -6.533  1.00  0.00           H
ATOM     29  CA  CYS N   3      -1.310  -0.994  -5.669  1.00  0.00           C
ATOM     30  HG1 THR N   2      -6.964   0.684  -5.607  1.00  0.00           H
ATOM     31 HG21 THR N   2      -7.960  -0.833  -7.308  1.00  0.00           H
ATOM     32 HG22 THR N   2      -7.750  -1.746  -5.806  1.00  0.00           H
ATOM     33 HG23 THR N   2      -7.130  -2.411  -7.328  1.00  0.00           H
ATOM     34  HA  CYS N   3      -1.338  -0.095  -5.040  1.00  0.00           H
ATOM     35  C   CYS N   3      -0.662  -0.709  -7.023  1.00  0.00           C
ATOM     36  CB  CYS N   3      -0.512  -2.104  -4.968  1.00  0.00           C
ATOM     37  O   CYS N   3      -0.690  -1.584  -7.904  1.00  0.00           O
ATOM     38  N   CYS N   4      -0.075   0.479  -7.208  1.00  0.00           N
ATOM     39  HB1 CYS N   3       0.510  -1.753  -4.785  1.00  0.00           H
ATOM     40  HB2 CYS N   3      -0.469  -2.995  -5.609  1.00  0.00           H
ATOM     41  SG  CYS N   3      -1.226  -2.706  -3.398  1.00  0.00           S
ATOM     42  H   CYS N   4      -0.046   1.199  -6.479  1.00  0.00           H
ATOM     43  CA  CYS N   4       0.416   0.827  -8.528  1.00  0.00           C
ATOM     44  SG  CYS N  40      -0.820  -1.125  -2.107  1.00  0.00           S
ATOM     45  HA  CYS N   4       0.126  -0.001  -9.178  1.00  0.00           H
```

ATOM	46	C	CYS	N	4	1.939	0.916	-8.521	1.00	0.00	C
ATOM	47	CB	CYS	N	4	-0.255	2.101	-9.050	1.00	0.00	C
ATOM	48	CB	CYS	N	40	0.921	-1.474	-1.645	1.00	0.00	C
ATOM	49	O	CYS	N	4	2.563	1.414	-7.570	1.00	0.00	O
ATOM	50	N	PRO	N	5	2.575	0.437	-9.605	1.00	0.00	N
ATOM	51	HB1	CYS	N	4	0.222	2.474	-9.965	1.00	0.00	H
ATOM	52	HB2	CYS	N	4	-0.205	2.912	-8.311	1.00	0.00	H
ATOM	53	SG	CYS	N	4	-2.046	1.849	-9.375	1.00	0.00	S
ATOM	54	HB1	CYS	N	40	1.549	-1.517	-2.545	1.00	0.00	H
ATOM	55	HB2	CYS	N	40	1.246	-0.591	-1.073	1.00	0.00	H
ATOM	56	CA	CYS	N	40	1.075	-2.741	-0.801	1.00	0.00	C
ATOM	57	CA	PRO	N	5	4.046	0.443	-9.656	1.00	0.00	C
ATOM	58	CD	PRO	N	5	1.975	-0.229	-10.790	1.00	0.00	C
ATOM	59	SG	CYS	N	32	-2.058	0.916	-11.241	1.00	0.00	S
ATOM	60	HA	CYS	N	40	0.639	-3.595	-1.332	1.00	0.00	H
ATOM	61	N	CYS	N	40	0.402	-2.581	0.462	1.00	0.00	N
ATOM	62	C	CYS	N	40	2.571	-2.985	-0.593	1.00	0.00	C
ATOM	63	HA	PRO	N	5	4.471	0.091	-8.710	1.00	0.00	H
ATOM	64	C	PRO	N	5	4.631	1.837	-9.876	1.00	0.00	C
ATOM	65	CB	PRO	N	5	4.348	-0.520	-10.812	1.00	0.00	C
ATOM	66	HD1	PRO	N	5	1.147	0.359	-11.204	1.00	0.00	H
ATOM	67	HD2	PRO	N	5	1.596	-1.219	-10.504	1.00	0.00	H
ATOM	68	CG	PRO	N	5	3.154	-0.357	-11.749	1.00	0.00	C
ATOM	69	CB	CYS	N	32	-2.384	-0.863	-10.866	1.00	0.00	C
ATOM	70	H	CYS	N	40	0.018	-1.659	0.693	1.00	0.00	H
ATOM	71	C	THR	N	39	0.323	-3.584	1.354	1.00	0.00	C
ATOM	72	O	CYS	N	40	3.194	-2.409	0.317	1.00	0.00	O
ATOM	73	N	PRO	N	41	3.175	-3.780	-1.485	1.00	0.00	N
ATOM	74	O	PRO	N	5	5.836	2.032	-9.650	1.00	0.00	O
ATOM	75	N	SER	N	6	3.795	2.768	-10.365	1.00	0.00	N
ATOM	76	HB1	PRO	N	5	5.317	-0.329	-11.282	1.00	0.00	H
ATOM	77	HB2	PRO	N	5	4.368	-1.540	-10.414	1.00	0.00	H
ATOM	78	HG1	PRO	N	5	3.036	-1.193	-12.446	1.00	0.00	H
ATOM	79	HG2	PRO	N	5	3.239	0.552	-12.357	1.00	0.00	H
ATOM	80	HB1	CYS	N	32	-2.654	-0.929	-9.806	1.00	0.00	H
ATOM	81	HB2	CYS	N	32	-3.274	-1.131	-11.451	1.00	0.00	H
ATOM	82	CA	CYS	N	32	-1.238	-1.843	-11.165	1.00	0.00	C
ATOM	83	O	THR	N	39	0.778	-4.717	1.156	1.00	0.00	O
ATOM	84	CA	THR	N	39	-0.437	-3.215	2.626	1.00	0.00	C
ATOM	85	CA	PRO	N	41	4.624	-3.949	-1.455	1.00	0.00	C
ATOM	86	CD	PRO	N	41	2.538	-4.512	-2.606	1.00	0.00	C
ATOM	87	H	SER	N	6	2.810	2.554	-10.481	1.00	0.00	H
ATOM	88	CA	SER	N	6	4.181	4.146	-10.560	1.00	0.00	C
ATOM	89	HA	CYS	N	32	-0.391	-1.626	-10.505	1.00	0.00	H
ATOM	90	N	CYS	N	32	-0.808	-1.774	-12.548	1.00	0.00	N
ATOM	91	C	CYS	N	32	-1.711	-3.276	-10.827	1.00	0.00	C
ATOM	92	HA	THR	N	39	-0.504	-2.123	2.722	1.00	0.00	H
ATOM	93	N	THR	N	39	-1.795	-3.714	2.510	1.00	0.00	N
ATOM	94	CB	THR	N	39	0.232	-3.793	3.894	1.00	0.00	C
ATOM	95	HA	PRO	N	41	4.956	-4.207	-0.440	1.00	0.00	H
ATOM	96	CB	PRO	N	41	4.847	-5.111	-2.443	1.00	0.00	C
ATOM	97	C	PRO	N	41	5.313	-2.637	-1.883	1.00	0.00	C
ATOM	98	HD1	PRO	N	41	1.833	-3.870	-3.151	1.00	0.00	H
ATOM	99	HD2	PRO	N	41	1.987	-5.383	-2.219	1.00	0.00	H
ATOM	100	CG	PRO	N	41	3.724	-4.949	-3.459	1.00	0.00	C
ATOM	101	HA	SER	N	6	4.943	4.384	-9.810	1.00	0.00	H
ATOM	102	C	SER	N	6	2.974	5.047	-10.328	1.00	0.00	C
ATOM	103	CB	SER	N	6	4.783	4.402	-11.937	1.00	0.00	C
ATOM	104	H	CYS	N	32	-1.475	-1.383	-13.218	1.00	0.00	H
ATOM	105	C	GLY	N	31	0.220	-2.509	-13.020	1.00	0.00	C
ATOM	106	O	CYS	N	32	-1.912	-4.107	-11.718	1.00	0.00	O
ATOM	107	N	ILE	N	33	-1.907	-3.522	-9.524	1.00	0.00	N
ATOM	108	H	THR	N	39	-1.893	-4.738	2.549	1.00	0.00	H

ATOM	109	C	ALA	N	38	-2.879	-2.923	2.442	1.00	0.00	C
ATOM	110	HB	THR	N	39	0.420	-4.866	3.733	1.00	0.00	H
ATOM	111	OG1	THR	N	39	-0.649	-3.628	5.001	1.00	0.00	O
ATOM	112	CG2	THR	N	39	1.549	-3.110	4.215	1.00	0.00	C
ATOM	113	HB1	PRO	N	41	5.838	-5.087	-2.887	1.00	0.00	H
ATOM	114	HB2	PRO	N	41	4.746	-6.061	-1.896	1.00	0.00	H
ATOM	115	O	PRO	N	41	4.727	-1.770	-2.543	1.00	0.00	O
ATOM	116	N	GLY	N	42	6.616	-2.517	-1.519	1.00	0.00	N
ATOM	117	HG1	PRO	N	41	3.521	-5.874	-4.007	1.00	0.00	H
ATOM	118	HG2	PRO	N	41	3.993	-4.157	-4.172	1.00	0.00	H
ATOM	119	O	SER	N	6	1.811	4.608	-10.369	1.00	0.00	O
ATOM	120	N	ILE	N	7	3.282	6.333	-10.111	1.00	0.00	N
ATOM	121	HB1	SER	N	6	5.301	5.365	-11.917	1.00	0.00	H
ATOM	122	HB2	SER	N	6	5.540	3.633	-12.128	1.00	0.00	H
ATOM	123	OG	SER	N	6	3.776	4.396	-12.945	1.00	0.00	O
ATOM	124	O	GLY	N	31	1.007	-3.124	-12.278	1.00	0.00	O
ATOM	125	CA	GLY	N	31	0.424	-2.558	-14.535	1.00	0.00	C
ATOM	126	H	ILE	N	33	-1.614	-2.824	-8.835	1.00	0.00	H
ATOM	127	CA	ILE	N	33	-2.190	-4.860	-9.045	1.00	0.00	C
ATOM	128	O	ALA	N	38	-2.827	-1.694	2.236	1.00	0.00	O
ATOM	129	CA	ALA	N	38	-4.224	-3.601	2.746	1.00	0.00	C
ATOM	130	HG1	THR	N	39	-1.361	-4.311	4.940	1.00	0.00	H
ATOM	131	HG21	THR	N	39	2.264	-3.233	3.390	1.00	0.00	H
ATOM	132	HG22	THR	N	39	1.986	-3.551	5.124	1.00	0.00	H
ATOM	133	HG23	THR	N	39	1.403	-2.028	4.366	1.00	0.00	H
ATOM	134	H	GLY	N	42	7.086	-3.306	-1.085	1.00	0.00	H
ATOM	135	CA	GLY	N	42	7.403	-1.326	-1.795	1.00	0.00	C
ATOM	136	H	ILE	N	7	4.275	6.607	-10.065	1.00	0.00	H
ATOM	137	CA	ILE	N	7	2.262	7.357	-9.959	1.00	0.00	C
ATOM	138	H	SER	N	6	4.194	4.838	-13.710	1.00	0.00	H
ATOM	139	HA1	GLY	N	31	0.524	-3.613	-14.820	1.00	0.00	H
ATOM	140	HA2	GLY	N	31	1.375	-2.056	-14.768	1.00	0.00	H
ATOM	141	N	GLY	N	31	-0.650	-1.971	-15.301	1.00	0.00	N
ATOM	142	HA	ILE	N	33	-2.575	-5.434	-9.896	1.00	0.00	H
ATOM	143	C	ILE	N	33	-3.277	-4.788	-7.981	1.00	0.00	C
ATOM	144	CB	ILE	N	33	-0.907	-5.586	-8.545	1.00	0.00	C
ATOM	145	HA	ALA	N	38	-4.925	-3.273	1.985	1.00	0.00	H
ATOM	146	N	ALA	N	38	-4.144	-5.058	2.692	1.00	0.00	N
ATOM	147	CB	ALA	N	38	-4.718	-3.089	4.109	1.00	0.00	C
ATOM	148	HA1	GLY	N	42	8.334	-1.369	-1.222	1.00	0.00	H
ATOM	149	HA2	GLY	N	42	6.865	-0.434	-1.442	1.00	0.00	H
ATOM	150	C	GLY	N	42	7.757	-1.056	-3.253	1.00	0.00	C
ATOM	151	HA	ILE	N	7	1.515	7.000	-9.235	1.00	0.00	H
ATOM	152	C	ILE	N	7	1.427	7.554	-11.232	1.00	0.00	C
ATOM	153	CB	ILE	N	7	2.897	8.654	-9.409	1.00	0.00	C
ATOM	154	H	GLY	N	31	-1.448	-2.568	-15.512	1.00	0.00	H
ATOM	155	C	THR	N	30	-0.734	-0.660	-15.569	1.00	0.00	C
ATOM	156	O	ILE	N	33	-3.483	-3.756	-7.318	1.00	0.00	O
ATOM	157	N	ILE	N	34	-3.963	-5.932	-7.800	1.00	0.00	N
ATOM	158	HB	ILE	N	33	-1.210	-6.611	-8.296	1.00	0.00	H
ATOM	159	CG2	ILE	N	33	0.099	-5.653	-9.696	1.00	0.00	C
ATOM	160	CG1	ILE	N	33	-0.324	-4.968	-7.261	1.00	0.00	C
ATOM	161	H	ALA	N	38	-3.641	-5.487	3.479	1.00	0.00	H
ATOM	162	C	GLY	N	37	-4.583	-5.842	1.689	1.00	0.00	C
ATOM	163	HB1	ALA	N	38	-5.708	-3.502	4.336	1.00	0.00	H
ATOM	164	HB2	ALA	N	38	-4.026	-3.365	4.915	1.00	0.00	H
ATOM	165	HB3	ALA	N	38	-4.801	-1.994	4.074	1.00	0.00	H
ATOM	166	O	GLY	N	42	8.312	0.007	-3.562	1.00	0.00	O
ATOM	167	N	ASP	N	43	7.372	-1.991	-4.145	1.00	0.00	N
ATOM	168	O	ILE	N	7	0.196	7.663	-11.118	1.00	0.00	O
ATOM	169	N	VAL	N	8	2.046	7.582	-12.425	1.00	0.00	N
ATOM	170	HB	ILE	N	7	3.833	8.833	-9.969	1.00	0.00	H
ATOM	171	CG2	ILE	N	7	1.990	9.858	-9.628	1.00	0.00	C

ATOM	172	CG1	ILE	N	7	3.238	8.442	-7.920	1.00	0.00	C
ATOM	173	O	THR	N	30	0.137	0.170	-15.256	1.00	0.00	O
ATOM	174	CA	THR	N	30	-2.048	-0.155	-16.154	1.00	0.00	C
ATOM	175	H	ILE	N	34	-3.720	-6.734	-8.387	1.00	0.00	H
ATOM	176	CA	ILE	N	34	-4.896	-6.102	-6.699	1.00	0.00	C
ATOM	177	HG21	ILE	N	33	1.059	-6.031	-9.349	1.00	0.00	H
ATOM	178	HG22	ILE	N	33	0.294	-4.668	-10.135	1.00	0.00	H
ATOM	179	HG23	ILE	N	33	-0.259	-6.319	-10.494	1.00	0.00	H
ATOM	180	HG11	ILE	N	33	0.036	-3.948	-7.467	1.00	0.00	H
ATOM	181	HG12	ILE	N	33	-1.135	-4.867	-6.519	1.00	0.00	H
ATOM	182	CD1	ILE	N	33	0.806	-5.788	-6.634	1.00	0.00	C
ATOM	183	O	GLY	N	37	-5.192	-5.439	0.685	1.00	0.00	O
ATOM	184	CA	GLY	N	37	-4.277	-7.340	1.876	1.00	0.00	C
ATOM	185	H	ASP	N	43	7.115	-2.921	-3.822	1.00	0.00	H
ATOM	186	CA	ASP	N	43	7.540	-1.865	-5.575	1.00	0.00	C
ATOM	187	H	VAL	N	8	3.075	7.571	-12.474	1.00	0.00	H
ATOM	188	CA	VAL	N	8	1.231	7.642	-13.635	1.00	0.00	C
ATOM	189	HG21	ILE	N	7	2.486	10.761	-9.261	1.00	0.00	H
ATOM	190	HG22	ILE	N	7	1.774	10.026	-10.692	1.00	0.00	H
ATOM	191	HG23	ILE	N	7	1.030	9.743	-9.102	1.00	0.00	H
ATOM	192	HG11	ILE	N	7	3.912	7.578	-7.833	1.00	0.00	H
ATOM	193	HG12	ILE	N	7	2.305	8.163	-7.398	1.00	0.00	H
ATOM	194	CD1	ILE	N	7	3.882	9.623	-7.203	1.00	0.00	C
ATOM	195	HA	THR	N	30	-1.784	0.506	-16.991	1.00	0.00	H
ATOM	196	N	THR	N	30	-2.922	-1.203	-16.648	1.00	0.00	N
ATOM	197	CB	THR	N	30	-2.743	0.671	-15.042	1.00	0.00	C
ATOM	198	HA	ILE	N	34	-4.968	-5.129	-6.201	1.00	0.00	H
ATOM	199	C	ILE	N	34	-4.312	-7.131	-5.740	1.00	0.00	C
ATOM	200	CB	ILE	N	34	-6.308	-6.589	-7.113	1.00	0.00	C
ATOM	201	HD11	ILE	N	33	1.699	-5.811	-7.274	1.00	0.00	H
ATOM	202	HD12	ILE	N	33	1.115	-5.356	-5.670	1.00	0.00	H
ATOM	203	HD13	ILE	N	33	0.476	-6.826	-6.460	1.00	0.00	H
ATOM	204	HA1	GLY	N	37	-3.203	-7.445	2.083	1.00	0.00	H
ATOM	205	HA2	GLY	N	37	-4.844	-7.699	2.749	1.00	0.00	H
ATOM	206	N	GLY	N	37	-4.587	-8.140	0.714	1.00	0.00	N
ATOM	207	HA	ASP	N	43	8.092	-0.928	-5.740	1.00	0.00	H
ATOM	208	CB	ASP	N	43	8.362	-3.012	-6.185	1.00	0.00	C
ATOM	209	C	ASP	N	43	6.188	-1.674	-6.293	1.00	0.00	C
ATOM	210	HA	VAL	N	8	0.474	8.427	-13.495	1.00	0.00	H
ATOM	211	C	VAL	N	8	0.386	6.376	-13.797	1.00	0.00	C
ATOM	212	CB	VAL	N	8	2.063	7.992	-14.894	1.00	0.00	C
ATOM	213	HD11	ILE	N	7	4.830	9.910	-7.679	1.00	0.00	H
ATOM	214	HD12	ILE	N	7	4.099	9.366	-6.157	1.00	0.00	H
ATOM	215	HD13	ILE	N	7	3.236	10.512	-7.188	1.00	0.00	H
ATOM	216	H	THR	N	30	-3.621	-1.601	-16.018	1.00	0.00	H
ATOM	217	C	TYR	N	29	-3.032	-1.494	-17.965	1.00	0.00	C
ATOM	218	HB	THR	N	30	-2.024	1.426	-14.698	1.00	0.00	H
ATOM	219	OG1	THR	N	30	-2.982	-0.172	-13.904	1.00	0.00	O
ATOM	220	CG2	THR	N	30	-4.002	1.378	-15.522	1.00	0.00	C
ATOM	221	O	ILE	N	34	-3.969	-8.261	-6.165	1.00	0.00	O
ATOM	222	N	ILE	N	35	-4.250	-6.788	-4.452	1.00	0.00	N
ATOM	223	HB	ILE	N	34	-6.201	-7.637	-7.440	1.00	0.00	H
ATOM	224	CG1	ILE	N	34	-6.892	-5.815	-8.301	1.00	0.00	C
ATOM	225	CG2	ILE	N	34	-7.240	-6.541	-5.895	1.00	0.00	C
ATOM	226	H	GLY	N	37	-3.784	-8.445	0.152	1.00	0.00	H
ATOM	227	C	PRO	N	36	-5.817	-8.596	0.446	1.00	0.00	C
ATOM	228	HB1	ASP	N	43	8.484	-2.856	-7.262	1.00	0.00	H
ATOM	229	HB2	ASP	N	43	9.363	-2.978	-5.733	1.00	0.00	H
ATOM	230	CG	ASP	N	43	7.824	-4.422	-5.959	1.00	0.00	C
ATOM	231	O	ASP	N	43	6.149	-1.501	-7.525	1.00	0.00	O
ATOM	232	N	TYR	N	44	5.089	-1.662	-5.524	1.00	0.00	N
ATOM	233	O	VAL	N	8	-0.765	6.476	-14.243	1.00	0.00	O
ATOM	234	N	ALA	N	9	0.912	5.191	-13.434	1.00	0.00	N

```
ATOM    235  HB  VAL N   8       3.046   7.497 -14.789  1.00  0.00           H
ATOM    236  CG1 VAL N   8       1.426   7.475 -16.191  1.00  0.00           C
ATOM    237  CG2 VAL N   8       2.274   9.512 -14.939  1.00  0.00           C
ATOM    238  O   TYR N  29      -2.303  -1.017 -18.833  1.00  0.00           O
ATOM    239  CA  TYR N  29      -4.204  -2.404 -18.355  1.00  0.00           C
ATOM    240  HG1 THR N  30      -3.774  -0.727 -14.089  1.00  0.00           H
ATOM    241 HG21 THR N  30      -4.356   2.054 -14.738  1.00  0.00           H
ATOM    242 HG22 THR N  30      -4.803   0.669 -15.770  1.00  0.00           H
ATOM    243 HG23 THR N  30      -3.776   1.981 -16.412  1.00  0.00           H
ATOM    244  H   ILE N  35      -4.581  -5.860  -4.169  1.00  0.00           H
ATOM    245  CA  ILE N  35      -3.826  -7.743  -3.443  1.00  0.00           C
ATOM    246 HG11 ILE N  34      -6.137  -5.758  -9.097  1.00  0.00           H
ATOM    247 HG12 ILE N  34      -7.123  -4.780  -7.991  1.00  0.00           H
ATOM    248  CD1 ILE N  34      -8.139  -6.496  -8.862  1.00  0.00           C
ATOM    249 HG21 ILE N  34      -8.233  -6.922  -6.162  1.00  0.00           H
ATOM    250 HG22 ILE N  34      -6.879  -7.150  -5.055  1.00  0.00           H
ATOM    251 HG23 ILE N  34      -7.354  -5.508  -5.529  1.00  0.00           H
ATOM    252  O   PRO N  36      -6.810  -8.400   1.168  1.00  0.00           O
ATOM    253  CA  PRO N  36      -6.044  -9.432  -0.815  1.00  0.00           C
ATOM    254  OD2 ASP N  43       7.101  -4.644  -4.943  1.00  0.00           O
ATOM    255  OD1 ASP N  43       8.161  -5.311  -6.808  1.00  0.00           O
ATOM    256  H   TYR N  44       5.196  -1.753  -4.510  1.00  0.00           H
ATOM    257  CA  TYR N  44       3.775  -1.270  -6.020  1.00  0.00           C
ATOM    258  H   ALA N   9       1.867   5.122 -13.077  1.00  0.00           H
ATOM    259  CA  ALA N   9       0.073   3.995 -13.471  1.00  0.00           C
ATOM    260 HG11 VAL N   8       2.055   7.744 -17.049  1.00  0.00           H
ATOM    261 HG12 VAL N   8       1.319   6.380 -16.193  1.00  0.00           H
ATOM    262 HG13 VAL N   8       0.420   7.892 -16.353  1.00  0.00           H
ATOM    263 HG21 VAL N   8       2.835   9.804 -15.835  1.00  0.00           H
ATOM    264 HG22 VAL N   8       2.824   9.876 -14.059  1.00  0.00           H
ATOM    265 HG23 VAL N   8       1.303  10.033 -14.967  1.00  0.00           H
ATOM    266  HA  TYR N  29      -3.902  -2.810 -19.329  1.00  0.00           H
ATOM    267  N   TYR N  29      -4.407  -3.515 -17.429  1.00  0.00           N
ATOM    268  CB  TYR N  29      -5.489  -1.558 -18.523  1.00  0.00           C
ATOM    269  HA  ILE N  35      -3.648  -8.672  -3.990  1.00  0.00           H
ATOM    270  C   ILE N  35      -4.960  -7.961  -2.446  1.00  0.00           C
ATOM    271  CB  ILE N  35      -2.493  -7.342  -2.747  1.00  0.00           C
ATOM    272 HD11 ILE N  34      -8.495  -5.981  -9.756  1.00  0.00           H
ATOM    273 HD12 ILE N  34      -8.976  -6.511  -8.152  1.00  0.00           H
ATOM    274 HD13 ILE N  34      -7.915  -7.535  -9.145  1.00  0.00           H
ATOM    275  HA  PRO N  36      -7.053  -9.177  -1.156  1.00  0.00           H
ATOM    276  N   PRO N  36      -5.062  -9.177  -1.879  1.00  0.00           N
ATOM    277  CB  PRO N  36      -5.973 -10.957  -0.565  1.00  0.00           C
ATOM    278  HA  TYR N  44       3.938  -0.794  -6.995  1.00  0.00           H
ATOM    279  CB  TYR N  44       2.815  -2.446  -6.211  1.00  0.00           C
ATOM    280  C   TYR N  44       3.235  -0.200  -5.067  1.00  0.00           C
ATOM    281  HA  ALA N   9      -0.320   3.873 -14.489  1.00  0.00           H
ATOM    282  CB  ALA N   9       0.872   2.740 -13.121  1.00  0.00           C
ATOM    283  C   ALA N   9      -1.174   4.191 -12.584  1.00  0.00           C
ATOM    284  H   TYR N  29      -5.325  -3.628 -16.986  1.00  0.00           H
ATOM    285  C   THR N  28      -3.537  -4.542 -17.426  1.00  0.00           C
ATOM    286  HB1 TYR N  29      -5.759  -1.112 -17.555  1.00  0.00           H
ATOM    287  HB2 TYR N  29      -6.313  -2.239 -18.786  1.00  0.00           H
ATOM    288  CG  TYR N  29      -5.292  -0.491 -19.575  1.00  0.00           C
ATOM    289  O   ILE N  35      -5.720  -7.017  -2.136  1.00  0.00           O
ATOM    290  HB  ILE N  35      -2.276  -8.158  -2.040  1.00  0.00           H
ATOM    291  CG2 ILE N  35      -1.366  -7.297  -3.789  1.00  0.00           C
ATOM    292  CG1 ILE N  35      -2.628  -6.041  -1.932  1.00  0.00           C
ATOM    293  CD  PRO N  36      -4.429 -10.420  -2.370  1.00  0.00           C
ATOM    294  HB1 PRO N  36      -5.980 -11.179   0.509  1.00  0.00           H
ATOM    295  HB2 PRO N  36      -6.865 -11.420  -1.009  1.00  0.00           H
ATOM    296  CG  PRO N  36      -4.681 -11.427  -1.248  1.00  0.00           C
ATOM    297  HB1 TYR N  44       1.834  -2.023  -6.455  1.00  0.00           H
```

ATOM	298	HB2	TYR	N	44	2.702	-2.983	-5.260	1.00	0.00	H
ATOM	299	CG	TYR	N	44	3.240	-3.355	-7.331	1.00	0.00	C
ATOM	300	O	TYR	N	44	2.169	-0.335	-4.443	1.00	0.00	O
ATOM	301	N	ALA	N	45	4.050	0.858	-4.931	1.00	0.00	N
ATOM	302	HB1	ALA	N	9	0.215	1.862	-13.147	1.00	0.00	H
ATOM	303	HB2	ALA	N	9	1.673	2.580	-13.856	1.00	0.00	H
ATOM	304	HB3	ALA	N	9	1.330	2.825	-12.127	1.00	0.00	H
ATOM	305	O	ALA	N	9	-2.279	3.795	-12.985	1.00	0.00	O
ATOM	306	N	ARG	N	10	-1.010	4.807	-11.394	1.00	0.00	N
ATOM	307	O	THR	N	28	-2.536	-4.556	-18.164	1.00	0.00	O
ATOM	308	CA	THR	N	28	-3.762	-5.720	-16.464	1.00	0.00	C
ATOM	309	CD1	TYR	N	29	-5.055	-0.861	-20.904	1.00	0.00	C
ATOM	310	CD2	TYR	N	29	-5.213	0.870	-19.257	1.00	0.00	C
ATOM	311	HG21	ILE	N	35	-0.376	-7.209	-3.324	1.00	0.00	H
ATOM	312	HG22	ILE	N	35	-1.343	-8.212	-4.401	1.00	0.00	H
ATOM	313	HG23	ILE	N	35	-1.489	-6.451	-4.484	1.00	0.00	H
ATOM	314	HG11	ILE	N	35	-3.509	-6.115	-1.276	1.00	0.00	H
ATOM	315	HG12	ILE	N	35	-2.827	-5.200	-2.621	1.00	0.00	H
ATOM	316	CD1	ILE	N	35	-1.414	-5.709	-1.066	1.00	0.00	C
ATOM	317	HD1	PRO	N	36	-3.361	-10.264	-2.536	1.00	0.00	H
ATOM	318	HD2	PRO	N	36	-4.908	-10.711	-3.320	1.00	0.00	H
ATOM	319	HG1	PRO	N	36	-3.855	-11.401	-0.530	1.00	0.00	H
ATOM	320	HG2	PRO	N	36	-4.763	-12.455	-1.629	1.00	0.00	H
ATOM	321	CD1	TYR	N	44	2.664	-3.183	-8.592	1.00	0.00	C
ATOM	322	CD2	TYR	N	44	4.223	-4.344	-7.182	1.00	0.00	C
ATOM	323	H	ALA	N	45	4.840	0.978	-5.579	1.00	0.00	H
ATOM	324	CA	ALA	N	45	3.945	1.800	-3.839	1.00	0.00	C
ATOM	325	H	ARG	N	10	-0.067	5.076	-11.110	1.00	0.00	H
ATOM	326	CA	ARG	N	10	-2.135	5.212	-10.551	1.00	0.00	C
ATOM	327	HA	THR	N	28	-2.738	-6.005	-16.187	1.00	0.00	H
ATOM	328	N	THR	N	28	-4.470	-5.307	-15.261	1.00	0.00	N
ATOM	329	CB	THR	N	28	-4.390	-6.938	-17.163	1.00	0.00	C
ATOM	330	HD1	TYR	N	29	-5.086	-1.913	-21.185	1.00	0.00	H
ATOM	331	CE1	TYR	N	29	-4.737	0.074	-21.881	1.00	0.00	C
ATOM	332	HD2	TYR	N	29	-5.391	1.193	-18.229	1.00	0.00	H
ATOM	333	CE2	TYR	N	29	-4.889	1.824	-20.222	1.00	0.00	C
ATOM	334	HD11	ILE	N	35	-0.502	-5.564	-1.663	1.00	0.00	H
ATOM	335	HD12	ILE	N	35	-1.206	-6.505	-0.336	1.00	0.00	H
ATOM	336	HD13	ILE	N	35	-1.586	-4.783	-0.498	1.00	0.00	H
ATOM	337	HD1	TYR	N	44	1.888	-2.427	-8.724	1.00	0.00	H
ATOM	338	CE1	TYR	N	44	3.028	-3.972	-9.678	1.00	0.00	C
ATOM	339	HD2	TYR	N	44	4.703	-4.507	-6.218	1.00	0.00	H
ATOM	340	CE2	TYR	N	44	4.594	-5.150	-8.254	1.00	0.00	C
ATOM	341	HA	ALA	N	45	3.505	1.267	-2.983	1.00	0.00	H
ATOM	342	C	ALA	N	45	3.025	2.978	-4.134	1.00	0.00	C
ATOM	343	CB	ALA	N	45	5.343	2.326	-3.501	1.00	0.00	C
ATOM	344	HA	ARG	N	10	-2.747	4.328	-10.340	1.00	0.00	H
ATOM	345	C	ARG	N	10	-3.086	6.191	-11.248	1.00	0.00	C
ATOM	346	CB	ARG	N	10	-1.615	5.831	-9.242	1.00	0.00	C
ATOM	347	H	THR	N	28	-5.450	-5.571	-15.129	1.00	0.00	H
ATOM	348	C	ALA	N	27	-3.856	-4.419	-14.449	1.00	0.00	C
ATOM	349	HB	THR	N	28	-3.822	-7.046	-18.107	1.00	0.00	H
ATOM	350	OG1	THR	N	28	-5.749	-6.635	-17.442	1.00	0.00	O
ATOM	351	CG2	THR	N	28	-4.246	-8.241	-16.381	1.00	0.00	C
ATOM	352	HE1	TYR	N	29	-4.548	-0.248	-22.902	1.00	0.00	H
ATOM	353	CZ	TYR	N	29	-4.624	1.433	-21.543	1.00	0.00	C
ATOM	354	HE2	TYR	N	29	-4.809	2.881	-19.963	1.00	0.00	H
ATOM	355	HE1	TYR	N	44	2.548	-3.819	-10.640	1.00	0.00	H
ATOM	356	CZ	TYR	N	44	3.983	-4.980	-9.504	1.00	0.00	C
ATOM	357	HE2	TYR	N	44	5.355	-5.920	-8.129	1.00	0.00	H
ATOM	358	O	ALA	N	45	2.772	3.797	-3.219	1.00	0.00	O
ATOM	359	N	ASN	N	46	2.587	3.118	-5.376	1.00	0.00	N
ATOM	360	HB1	ALA	N	45	5.357	2.881	-2.562	1.00	0.00	H

ATOM	361	HB2	ALA	N	45	5.687	3.008	-4.288	1.00	0.00	H
ATOM	362	HB3	ALA	N	45	6.056	1.495	-3.422	1.00	0.00	H
ATOM	363	O	ARG	N	10	-4.316	6.018	-11.172	1.00	0.00	O
ATOM	364	N	SER	N	11	-2.540	7.241	-11.882	1.00	0.00	N
ATOM	365	HB1	ARG	N	10	-1.054	5.063	-8.693	1.00	0.00	H
ATOM	366	HB2	ARG	N	10	-0.895	6.620	-9.499	1.00	0.00	H
ATOM	367	CG	ARG	N	10	-2.694	6.456	-8.349	1.00	0.00	C
ATOM	368	O	ALA	N	27	-2.691	-4.047	-14.667	1.00	0.00	O
ATOM	369	CA	ALA	N	27	-4.626	-3.892	-13.226	1.00	0.00	C
ATOM	370	HG1	THR	N	28	-6.009	-7.269	-18.155	1.00	0.00	H
ATOM	371	HG21	THR	N	28	-4.661	-9.068	-16.975	1.00	0.00	H
ATOM	372	HG22	THR	N	28	-3.191	-8.493	-16.202	1.00	0.00	H
ATOM	373	HG23	THR	N	28	-4.786	-8.196	-15.425	1.00	0.00	H
ATOM	374	OH	TYR	N	29	-4.247	2.373	-22.441	1.00	0.00	O
ATOM	375	OH	TYR	N	44	4.345	-5.824	-10.507	1.00	0.00	O
ATOM	376	H	ASN	N	46	2.736	2.374	-6.067	1.00	0.00	H
ATOM	377	CA	ASN	N	46	1.877	4.292	-5.837	1.00	0.00	C
ATOM	378	H	SER	N	11	-1.529	7.409	-11.832	1.00	0.00	H
ATOM	379	CA	SER	N	11	-3.399	8.175	-12.593	1.00	0.00	C
ATOM	380	HG1	ARG	N	10	-2.188	6.966	-7.517	1.00	0.00	H
ATOM	381	HG2	ARG	N	10	-3.221	7.255	-8.892	1.00	0.00	H
ATOM	382	CD	ARG	N	10	-3.751	5.513	-7.782	1.00	0.00	C
ATOM	383	HA	ALA	N	27	-3.995	-3.081	-12.845	1.00	0.00	H
ATOM	384	N	ALA	N	27	-5.933	-3.360	-13.611	1.00	0.00	N
ATOM	385	CB	ALA	N	27	-4.789	-4.978	-12.158	1.00	0.00	C
ATOM	386	HH	TYR	N	29	-4.351	2.018	-23.368	1.00	0.00	H
ATOM	387	HH	TYR	N	44	3.841	-5.627	-11.336	1.00	0.00	H
ATOM	388	HA	ASN	N	46	1.963	5.059	-5.064	1.00	0.00	H
ATOM	389	CB	ASN	N	46	2.560	4.826	-7.105	1.00	0.00	C
ATOM	390	C	ASN	N	46	0.366	4.035	-5.977	1.00	0.00	C
ATOM	391	HA	SER	N	11	-4.129	8.593	-11.890	1.00	0.00	H
ATOM	392	CB	SER	N	11	-2.602	9.316	-13.231	1.00	0.00	C
ATOM	393	C	SER	N	11	-4.219	7.423	-13.662	1.00	0.00	C
ATOM	394	HD1	ARG	N	10	-4.481	6.108	-7.215	1.00	0.00	H
ATOM	395	HD2	ARG	N	10	-4.308	5.004	-8.584	1.00	0.00	H
ATOM	396	NE	ARG	N	10	-3.134	4.515	-6.906	1.00	0.00	N
ATOM	397	H	ALA	N	27	-6.769	-3.795	-13.204	1.00	0.00	H
ATOM	398	C	CYS	N	26	-6.086	-2.275	-14.369	1.00	0.00	C
ATOM	399	HB1	ALA	N	27	-5.501	-5.743	-12.480	1.00	0.00	H
ATOM	400	HB2	ALA	N	27	-3.820	-5.454	-11.972	1.00	0.00	H
ATOM	401	HB3	ALA	N	27	-5.152	-4.537	-11.218	1.00	0.00	H
ATOM	402	HB1	ASN	N	46	2.403	4.142	-7.942	1.00	0.00	H
ATOM	403	HB2	ASN	N	46	2.126	5.799	-7.363	1.00	0.00	H
ATOM	404	CG	ASN	N	46	4.066	4.934	-6.865	1.00	0.00	C
ATOM	405	O	ASN	N	46	-0.337	4.977	-6.432	1.00	0.00	O
ATOM	406	OXT	ASN	N	46	-0.093	2.909	-5.621	1.00	0.00	O
ATOM	407	HB1	SER	N	11	-3.327	9.978	-13.729	1.00	0.00	H
ATOM	408	HB2	SER	N	11	-1.919	8.905	-13.994	1.00	0.00	H
ATOM	409	OG	SER	N	11	-1.878	10.024	-12.237	1.00	0.00	O
ATOM	410	O	SER	N	11	-5.448	7.626	-13.806	1.00	0.00	O
ATOM	411	N	ASN	N	12	-3.552	6.548	-14.409	1.00	0.00	N
ATOM	412	HE	ARG	N	10	-2.146	4.659	-6.648	1.00	0.00	H
ATOM	413	CZ	ARG	N	10	-3.684	3.345	-6.567	1.00	0.00	C
ATOM	414	O	CYS	N	26	-5.133	-1.574	-14.768	1.00	0.00	O
ATOM	415	CA	CYS	N	26	-7.506	-1.891	-14.823	1.00	0.00	C
ATOM	416	OD1	ASN	N	46	4.849	4.176	-7.465	1.00	0.00	O
ATOM	417	ND2	ASN	N	46	4.480	5.829	-5.957	1.00	0.00	N
ATOM	418	H	SER	N	11	-1.438	10.800	-12.679	1.00	0.00	H
ATOM	419	H	ASN	N	12	-2.535	6.446	-14.290	1.00	0.00	H
ATOM	420	CA	ASN	N	12	-4.225	5.761	-15.431	1.00	0.00	C
ATOM	421	NH1	ARG	N	10	-4.978	3.099	-6.809	1.00	0.00	N
ATOM	422	NH2	ARG	N	10	-2.901	2.393	-6.032	1.00	0.00	N
ATOM	423	HA	CYS	N	26	-7.335	-1.462	-15.821	1.00	0.00	H

```
ATOM    424  N   CYS N  26      -8.369  -3.043 -14.955  1.00  0.00           N
ATOM    425  CB  CYS N  26      -8.132  -0.814 -13.941  1.00  0.00           C
ATOM    426 HD21 ASN N  46       3.830   6.466  -5.486  1.00  0.00           H
ATOM    427 HD22 ASN N  46       5.490   5.950  -5.792  1.00  0.00           H
ATOM    428  HA  ASN N  12      -4.766   6.438 -16.105  1.00  0.00           H
ATOM    429  CB  ASN N  12      -3.213   4.964 -16.247  1.00  0.00           C
ATOM    430  C   ASN N  12      -5.277   4.835 -14.817  1.00  0.00           C
ATOM    431 HH11 ARG N  10      -5.349   2.163  -6.604  1.00  0.00           H
ATOM    432 HH12 ARG N  10      -5.646   3.880  -6.770  1.00  0.00           H
ATOM    433 HH21 ARG N  10      -3.335   1.688  -5.438  1.00  0.00           H
ATOM    434 HH22 ARG N  10      -1.934   2.640  -5.785  1.00  0.00           H
ATOM    435  H   CYS N  26      -9.167  -3.159 -14.325  1.00  0.00           H
ATOM    436  C   LEU N  25      -8.125  -3.933 -15.939  1.00  0.00           C
ATOM    437  HB1 CYS N  26      -7.416  -0.003 -13.762  1.00  0.00           H
ATOM    438  HB2 CYS N  26      -8.429  -1.218 -12.962  1.00  0.00           H
ATOM    439  SG  CYS N  26      -9.688  -0.129 -14.642  1.00  0.00           S
ATOM    440  HB1 ASN N  12      -2.593   4.327 -15.599  1.00  0.00           H
ATOM    441  HB2 ASN N  12      -3.772   4.292 -16.918  1.00  0.00           H
ATOM    442  CG  ASN N  12      -2.348   5.877 -17.131  1.00  0.00           C
ATOM    443  O   ASN N  12      -6.363   4.657 -15.392  1.00  0.00           O
ATOM    444  N   PHE N  13      -4.951   4.232 -13.663  1.00  0.00           N
ATOM    445  O   LEU N  25      -7.233  -3.763 -16.779  1.00  0.00           O
ATOM    446  CA  LEU N  25      -8.993  -5.193 -15.973  1.00  0.00           C
ATOM    447  SG  CYS N  16      -9.064   0.644 -16.475  1.00  0.00           S
ATOM    448  OD1 ASN N  12      -2.702   7.028 -17.391  1.00  0.00           O
ATOM    449  ND2 ASN N  12      -1.237   5.286 -17.602  1.00  0.00           N
ATOM    450  H   PHE N  13      -3.990   4.301 -13.305  1.00  0.00           H
ATOM    451  CA  PHE N  13      -5.873   3.355 -12.962  1.00  0.00           C
ATOM    452  HA  LEU N  25      -8.343  -5.940 -16.447  1.00  0.00           H
ATOM    453  N   LEU N  25      -9.310  -5.666 -14.626  1.00  0.00           N
ATOM    454  CB  LEU N  25     -10.277  -4.981 -16.788  1.00  0.00           C
ATOM    455  CB  CYS N  16      -8.578   2.362 -16.060  1.00  0.00           C
ATOM    456 HD21 ASN N  12      -1.001   4.335 -17.319  1.00  0.00           H
ATOM    457 HD22 ASN N  12      -0.686   5.756 -18.331  1.00  0.00           H
ATOM    458  HA  PHE N  13      -6.181   2.529 -13.621  1.00  0.00           H
ATOM    459  C   PHE N  13      -7.163   4.097 -12.615  1.00  0.00           C
ATOM    460  CB  PHE N  13      -5.193   2.780 -11.701  1.00  0.00           C
ATOM    461  H   LEU N  25     -10.163  -5.321 -14.184  1.00  0.00           H
ATOM    462  C   ALA N  24      -8.344  -6.148 -13.822  1.00  0.00           C
ATOM    463  HB1 LEU N  25     -10.999  -4.442 -16.154  1.00  0.00           H
ATOM    464  HB2 LEU N  25     -10.047  -4.308 -17.631  1.00  0.00           H
ATOM    465  CG  LEU N  25     -10.902  -6.281 -17.326  1.00  0.00           C
ATOM    466  HB1 CYS N  16      -8.146   2.372 -15.050  1.00  0.00           H
ATOM    467  HB2 CYS N  16      -7.778   2.627 -16.768  1.00  0.00           H
ATOM    468  CA  CYS N  16      -9.702   3.408 -16.156  1.00  0.00           C
ATOM    469  O   PHE N  13      -8.271   3.608 -12.872  1.00  0.00           O
ATOM    470  N   ASN N  14      -7.016   5.290 -12.001  1.00  0.00           N
ATOM    471  HB1 PHE N  13      -4.220   2.376 -12.005  1.00  0.00           H
ATOM    472  HB2 PHE N  13      -4.993   3.603 -11.001  1.00  0.00           H
ATOM    473  CG  PHE N  13      -6.013   1.695 -11.050  1.00  0.00           C
ATOM    474  O   ALA N  24      -7.181  -6.319 -14.231  1.00  0.00           O
ATOM    475  CA  ALA N  24      -8.740  -6.528 -12.387  1.00  0.00           C
ATOM    476  HG  LEU N  25     -10.953  -6.995 -16.488  1.00  0.00           H
ATOM    477  CD1 LEU N  25     -10.043  -6.912 -18.427  1.00  0.00           C
ATOM    478  CD2 LEU N  25     -12.340  -6.034 -17.802  1.00  0.00           C
ATOM    479  HA  CYS N  16     -10.060   3.478 -17.192  1.00  0.00           H
ATOM    480  N   CYS N  16      -9.135   4.681 -15.733  1.00  0.00           N
ATOM    481  C   CYS N  16     -10.956   3.033 -15.341  1.00  0.00           C
ATOM    482  H   ASN N  14      -6.077   5.657 -11.807  1.00  0.00           H
ATOM    483  CA  ASN N  14      -8.202   6.023 -11.599  1.00  0.00           C
ATOM    484  CD2 PHE N  13      -6.975   1.984 -10.072  1.00  0.00           C
ATOM    485  CD1 PHE N  13      -5.842   0.364 -11.460  1.00  0.00           C
ATOM    486  HA  ALA N  24      -7.796  -6.550 -11.828  1.00  0.00           H
```

ATOM	487	N	ALA	N	24	-9.628	-5.528	-11.806	1.00	0.00	N
ATOM	488	CB	ALA	N	24	-9.387	-7.913	-12.325	1.00	0.00	C
ATOM	489	HD11	LEU	N	25	-10.492	-7.841	-18.803	1.00	0.00	H
ATOM	490	HD12	LEU	N	25	-9.031	-7.165	-18.081	1.00	0.00	H
ATOM	491	HD13	LEU	N	25	-9.932	-6.231	-19.286	1.00	0.00	H
ATOM	492	HD21	LEU	N	25	-12.986	-5.698	-16.977	1.00	0.00	H
ATOM	493	HD22	LEU	N	25	-12.792	-6.949	-18.214	1.00	0.00	H
ATOM	494	HD23	LEU	N	25	-12.377	-5.258	-18.587	1.00	0.00	H
ATOM	495	H	CYS	N	16	-8.144	4.702	-15.461	1.00	0.00	H
ATOM	496	C	VAL	N	15	-9.865	5.810	-15.687	1.00	0.00	C
ATOM	497	O	CYS	N	16	-11.927	2.475	-15.875	1.00	0.00	O
ATOM	498	N	ARG	N	17	-10.930	3.365	-14.040	1.00	0.00	N
ATOM	499	HA	ASN	N	14	-8.851	5.341	-11.035	1.00	0.00	H
ATOM	500	CB	ASN	N	14	-7.874	7.238	-10.729	1.00	0.00	C
ATOM	501	C	ASN	N	14	-9.052	6.432	-12.814	1.00	0.00	C
ATOM	502	HD2	PHE	N	13	-7.134	3.015	-9.746	1.00	0.00	H
ATOM	503	CE2	PHE	N	13	-7.754	0.963	-9.524	1.00	0.00	C
ATOM	504	HD1	PHE	N	13	-5.097	0.143	-12.227	1.00	0.00	H
ATOM	505	CE1	PHE	N	13	-6.616	-0.659	-10.913	1.00	0.00	C
ATOM	506	H	ALA	N	24	-10.452	-5.869	-11.276	1.00	0.00	H
ATOM	507	C	GLU	N	23	-9.180	-4.275	-11.592	1.00	0.00	C
ATOM	508	HB1	ALA	N	24	-8.727	-8.655	-12.787	1.00	0.00	H
ATOM	509	HB2	ALA	N	24	-9.555	-8.197	-11.278	1.00	0.00	H
ATOM	510	HB3	ALA	N	24	-10.355	-7.932	-12.841	1.00	0.00	H
ATOM	511	O	VAL	N	15	-11.053	5.854	-16.039	1.00	0.00	O
ATOM	512	CA	VAL	N	15	-9.155	7.071	-15.167	1.00	0.00	C
ATOM	513	H	ARG	N	17	-10.060	3.724	-13.637	1.00	0.00	H
ATOM	514	CA	ARG	N	17	-12.041	3.089	-13.150	1.00	0.00	C
ATOM	515	HB1	ASN	N	14	-7.230	7.954	-11.258	1.00	0.00	H
ATOM	516	HB2	ASN	N	14	-8.830	7.748	-10.522	1.00	0.00	H
ATOM	517	CG	ASN	N	14	-7.293	6.896	-9.362	1.00	0.00	C
ATOM	518	O	ASN	N	14	-10.293	6.410	-12.737	1.00	0.00	O
ATOM	519	N	VAL	N	15	-8.413	6.802	-13.936	1.00	0.00	N
ATOM	520	HE2	PHE	N	13	-8.506	1.204	-8.769	1.00	0.00	H
ATOM	521	CZ	PHE	N	13	-7.581	-0.355	-9.951	1.00	0.00	C
ATOM	522	HE1	PHE	N	13	-6.492	-1.692	-11.246	1.00	0.00	H
ATOM	523	O	GLU	N	23	-8.052	-3.892	-11.937	1.00	0.00	O
ATOM	524	CA	GLU	N	23	-10.113	-3.301	-10.863	1.00	0.00	C
ATOM	525	HA	VAL	N	15	-9.978	7.759	-14.933	1.00	0.00	H
ATOM	526	CB	VAL	N	15	-8.237	7.726	-16.224	1.00	0.00	C
ATOM	527	HA	ARG	N	17	-12.470	2.108	-13.412	1.00	0.00	H
ATOM	528	C	ARG	N	17	-13.210	4.059	-13.323	1.00	0.00	C
ATOM	529	CB	ARG	N	17	-11.564	3.087	-11.688	1.00	0.00	C
ATOM	530	OD1	ASN	N	14	-7.650	5.854	-8.756	1.00	0.00	O
ATOM	531	ND2	ASN	N	14	-6.466	7.789	-8.835	1.00	0.00	N
ATOM	532	H	VAL	N	15	-7.390	6.798	-13.962	1.00	0.00	H
ATOM	533	HZ	PHE	N	13	-8.192	-1.152	-9.531	1.00	0.00	H
ATOM	534	HA	GLU	N	23	-9.744	-2.305	-11.152	1.00	0.00	H
ATOM	535	N	GLU	N	23	-11.489	-3.450	-11.301	1.00	0.00	N
ATOM	536	CB	GLU	N	23	-9.956	-3.480	-9.342	1.00	0.00	C
ATOM	537	HB	VAL	N	15	-7.303	7.139	-16.254	1.00	0.00	H
ATOM	538	CG2	VAL	N	15	-7.910	9.158	-15.801	1.00	0.00	C
ATOM	539	CG1	VAL	N	15	-8.864	7.687	-17.619	1.00	0.00	C
ATOM	540	O	ARG	N	17	-14.324	3.736	-12.851	1.00	0.00	O
ATOM	541	N	LEU	N	18	-13.037	5.221	-13.961	1.00	0.00	N
ATOM	542	HB1	ARG	N	17	-11.080	4.048	-11.480	1.00	0.00	H
ATOM	543	HB2	ARG	N	17	-12.444	3.044	-11.030	1.00	0.00	H
ATOM	544	CG	ARG	N	17	-10.601	1.944	-11.360	1.00	0.00	C
ATOM	545	HD21	ASN	N	14	-6.183	7.683	-7.858	1.00	0.00	H
ATOM	546	HD22	ASN	N	14	-6.118	8.622	-9.359	1.00	0.00	H
ATOM	547	H	GLU	N	23	-12.214	-3.697	-10.603	1.00	0.00	H
ATOM	548	C	PRO	N	22	-11.794	-3.470	-12.610	1.00	0.00	C
ATOM	549	HB1	GLU	N	23	-10.331	-4.471	-9.063	1.00	0.00	H

ATOM	550	HB2	GLU	N	23	-8.876	-3.493	-9.132	1.00	0.00	H
ATOM	551	CG	GLU	N	23	-10.637	-2.411	-8.486	1.00	0.00	C
ATOM	552	HG21	VAL	N	15	-7.435	9.191	-14.815	1.00	0.00	H
ATOM	553	HG22	VAL	N	15	-7.223	9.645	-16.503	1.00	0.00	H
ATOM	554	HG23	VAL	N	15	-8.829	9.765	-15.750	1.00	0.00	H
ATOM	555	HG11	VAL	N	15	-8.228	8.221	-18.335	1.00	0.00	H
ATOM	556	HG12	VAL	N	15	-8.989	6.656	-17.983	1.00	0.00	H
ATOM	557	HG13	VAL	N	15	-9.858	8.162	-17.634	1.00	0.00	H
ATOM	558	H	LEU	N	18	-12.139	5.444	-14.396	1.00	0.00	H
ATOM	559	CA	LEU	N	18	-14.128	6.182	-14.096	1.00	0.00	C
ATOM	560	HG1	ARG	N	17	-9.809	1.869	-12.117	1.00	0.00	H
ATOM	561	HG2	ARG	N	17	-10.087	2.206	-10.424	1.00	0.00	H
ATOM	562	CD	ARG	N	17	-11.274	0.581	-11.219	1.00	0.00	C
ATOM	563	O	PRO	N	22	-10.949	-3.338	-13.516	1.00	0.00	O
ATOM	564	CA	PRO	N	22	-13.242	-3.821	-12.914	1.00	0.00	C
ATOM	565	HG1	GLU	N	23	-10.332	-1.399	-8.786	1.00	0.00	H
ATOM	566	HG2	GLU	N	23	-11.729	-2.454	-8.622	1.00	0.00	H
ATOM	567	CD	GLU	N	23	-10.382	-2.546	-6.985	1.00	0.00	C
ATOM	568	HA	LEU	N	18	-14.506	6.409	-13.089	1.00	0.00	H
ATOM	569	CB	LEU	N	18	-13.668	7.476	-14.776	1.00	0.00	C
ATOM	570	C	LEU	N	18	-15.369	5.618	-14.803	1.00	0.00	C
ATOM	571	HD1	ARG	N	17	-11.907	0.356	-12.086	1.00	0.00	H
ATOM	572	HD2	ARG	N	17	-10.501	-0.208	-11.188	1.00	0.00	H
ATOM	573	NE	ARG	N	17	-12.067	0.542	-9.997	1.00	0.00	N
ATOM	574	HA	PRO	N	22	-13.874	-3.601	-12.043	1.00	0.00	H
ATOM	575	N	PRO	N	22	-13.767	-3.116	-14.091	1.00	0.00	N
ATOM	576	CB	PRO	N	22	-13.375	-5.302	-13.297	1.00	0.00	C
ATOM	577	OE2	GLU	N	23	-10.531	-1.496	-6.287	1.00	0.00	O
ATOM	578	OE1	GLU	N	23	-10.066	-3.678	-6.501	1.00	0.00	O
ATOM	579	HB1	LEU	N	18	-14.571	8.034	-15.058	1.00	0.00	H
ATOM	580	HB2	LEU	N	18	-13.145	7.223	-15.710	1.00	0.00	H
ATOM	581	CG	LEU	N	18	-12.774	8.406	-13.935	1.00	0.00	C
ATOM	582	O	LEU	N	18	-16.498	5.929	-14.362	1.00	0.00	O
ATOM	583	N	PRO	N	19	-15.259	4.807	-15.872	1.00	0.00	N
ATOM	584	HE	ARG	N	17	-11.615	0.879	-9.135	1.00	0.00	H
ATOM	585	CZ	ARG	N	17	-13.204	-0.131	-9.764	1.00	0.00	C
ATOM	586	C	THR	N	21	-13.628	-1.786	-14.191	1.00	0.00	C
ATOM	587	CD	PRO	N	22	-14.570	-4.007	-14.955	1.00	0.00	C
ATOM	588	HB1	PRO	N	22	-13.456	-5.939	-12.410	1.00	0.00	H
ATOM	589	HB2	PRO	N	22	-12.499	-5.604	-13.894	1.00	0.00	H
ATOM	590	CG	PRO	N	22	-14.638	-5.311	-14.154	1.00	0.00	C
ATOM	591	HG	LEU	N	18	-11.847	7.867	-13.682	1.00	0.00	H
ATOM	592	CD1	LEU	N	18	-12.407	9.606	-14.817	1.00	0.00	C
ATOM	593	CD2	LEU	N	18	-13.440	8.839	-12.624	1.00	0.00	C
ATOM	594	CA	PRO	N	19	-16.468	4.173	-16.423	1.00	0.00	C
ATOM	595	CD	PRO	N	19	-14.109	4.716	-16.813	1.00	0.00	C
ATOM	596	NH1	ARG	N	17	-13.874	-0.775	-10.716	1.00	0.00	N
ATOM	597	NH2	ARG	N	17	-13.676	-0.116	-8.506	1.00	0.00	N
ATOM	598	O	THR	N	21	-13.034	-1.116	-13.330	1.00	0.00	O
ATOM	599	CA	THR	N	21	-14.110	-1.066	-15.459	1.00	0.00	C
ATOM	600	HD1	PRO	N	22	-15.565	-3.581	-15.131	1.00	0.00	H
ATOM	601	HD2	PRO	N	22	-14.064	-4.141	-15.925	1.00	0.00	H
ATOM	602	HG1	PRO	N	22	-14.721	-6.188	-14.802	1.00	0.00	H
ATOM	603	HG2	PRO	N	22	-15.517	-5.282	-13.501	1.00	0.00	H
ATOM	604	HD11	LEU	N	18	-11.735	10.305	-14.299	1.00	0.00	H
ATOM	605	HD12	LEU	N	18	-11.901	9.279	-15.738	1.00	0.00	H
ATOM	606	HD13	LEU	N	18	-13.303	10.172	-15.119	1.00	0.00	H
ATOM	607	HD21	LEU	N	18	-12.826	9.581	-12.093	1.00	0.00	H
ATOM	608	HD22	LEU	N	18	-13.578	7.994	-11.932	1.00	0.00	H
ATOM	609	HD23	LEU	N	18	-14.426	9.295	-12.802	1.00	0.00	H
ATOM	610	HA	PRO	N	19	-17.311	4.878	-16.413	1.00	0.00	H
ATOM	611	C	PRO	N	19	-16.945	2.943	-15.646	1.00	0.00	C
ATOM	612	CB	PRO	N	19	-16.067	3.769	-17.850	1.00	0.00	C

```
ATOM    613  HD1 PRO N  19      -13.179   4.475 -16.299  1.00  0.00            H
ATOM    614  HD2 PRO N  19      -13.988   5.692 -17.310  1.00  0.00            H
ATOM    615  CG  PRO N  19      -14.545   3.640 -17.802  1.00  0.00            C
ATOM    616 HH11 ARG N  17      -13.497  -0.887 -11.664  1.00  0.00            H
ATOM    617 HH12 ARG N  17      -14.640  -1.403 -10.448  1.00  0.00            H
ATOM    618 HH21 ARG N  17      -13.100   0.340  -7.795  1.00  0.00            H
ATOM    619 HH22 ARG N  17      -14.517  -0.645  -8.254  1.00  0.00            H
ATOM    620  HA  THR N  21      -14.712  -1.727 -16.100  1.00  0.00            H
ATOM    621  N   THR N  21      -14.893   0.096 -15.087  1.00  0.00            N
ATOM    622  CB  THR N  21      -12.848  -0.597 -16.220  1.00  0.00            C
ATOM    623  O   PRO N  19      -17.986   2.380 -16.021  1.00  0.00            O
ATOM    624  N   GLY N  20      -16.185   2.495 -14.631  1.00  0.00            N
ATOM    625  HB1 PRO N  19      -16.351   4.553 -18.552  1.00  0.00            H
ATOM    626  HB2 PRO N  19      -16.568   2.847 -18.156  1.00  0.00            H
ATOM    627  HG1 PRO N  19      -14.099   3.791 -18.792  1.00  0.00            H
ATOM    628  HG2 PRO N  19      -14.246   2.649 -17.437  1.00  0.00            H
ATOM    629  H   THR N  21      -14.723   0.953 -15.602  1.00  0.00            H
ATOM    630  C   GLY N  20      -15.853   0.056 -14.148  1.00  0.00            C
ATOM    631  HB  THR N  21      -12.221  -0.074 -15.488  1.00  0.00            H
ATOM    632  OG1 THR N  21      -13.238   0.349 -17.221  1.00  0.00            O
ATOM    633  CG2 THR N  21      -12.077  -1.769 -16.807  1.00  0.00            C
ATOM    634  H   GLY N  20      -15.418   3.072 -14.277  1.00  0.00            H
ATOM    635  CA  GLY N  20      -16.561   1.360 -13.811  1.00  0.00            C
ATOM    636  O   GLY N  20      -16.140  -0.980 -13.537  1.00  0.00            O
ATOM    637  HG1 THR N  21      -12.708   1.157 -17.050  1.00  0.00            H
ATOM    638 HG21 THR N  21      -11.237  -1.400 -17.411  1.00  0.00            H
ATOM    639 HG22 THR N  21      -11.670  -2.388 -15.997  1.00  0.00            H
ATOM    640 HG23 THR N  21      -12.713  -2.394 -17.452  1.00  0.00            H
ATOM    641  HA1 GLY N  20      -17.638   1.168 -13.902  1.00  0.00            H
ATOM    642  HA2 GLY N  20      -16.322   1.586 -12.763  1.00  0.00            H
END
```

OPTIMIZED GEOMETRIES (CIF files)

1) CRAMBIN CRYSTAL – optimized 0W

```
data_CSD_CIF_MOLDRAW
_audit_creation_date 09-04-2015
_audit_creation_method MOLDRAW
_chemical_formula_sum 'H630 C404 N110 O128 S12'
_chemical_formula_moiety
;
H630 C404 N110 O128 S12
;
loop_
_publ_author_name
Delle Piane, Corno, Orlando, Dovesi, Ugliengo
_cell_volume 14065.8965
_exptl_crystal_density_diffrn ?
_diffrn_ambient_temperature ?
_refine_ls_R_factor_gt ?
_refine_ls_wR_factor_gt ?
_symmetry_space_group_name_H-M 'P 1'
_symmetry_cell_setting monoclinic
_symmetry_Int_Tables_number 1
loop_
```

```
_symmetry_equiv_pos_site_id
_symmetry_equiv_pos_as_xyz
1 x,y,z
_cell_length_a  21.0266844
_cell_length_b  17.3555897
_cell_length_c  38.5449471
_cell_angle_alpha  90
_cell_angle_beta  89.607206
_cell_angle_gamma  90
_cell_formula_units_Z 1
loop_
_atom_type_symbol
_atom_type_radius_bond
H  .38
C  .77
N  .75
O  .73
S  1.04
loop_
_atom_site_label
_atom_site_type_symbol
_atom_site_fract_x
_atom_site_fract_y
_atom_site_fract_z
H1   H     0.702089974494   0.658514332352   0.911637597984
H2   H     0.297910025506   0.158514332349   0.088362402016
H3   H     0.627837929245   0.663252073808   0.898066075843
H4   H     0.372162070755   0.163252073805   0.101933924157
H5   H     0.644961898031   0.688339416351   0.938998811756
H6   H     0.355038101969   0.188339416347   0.061001188244
H7   H     0.623130253893   0.795824295119   0.895039771713
H8   H     0.376869746107   0.295824295116   0.104960228287
H9   H     0.741923723408   0.802921225082   0.940805182944
H10   H     0.258076276592   0.302921225079   0.059194817056
H11   H     0.682954779765   0.751895748011   0.977551583502
H12   H     0.317045220235   0.251895748007   0.022448416498
H13   H     0.698942718698   0.932039958019   0.957508456053
H14   H     0.301057281302   0.432039958015   0.042491543947
H15   H     0.633566660662   0.919362784610   0.928921049516
H16   H     0.366433339338   0.419362784606   0.071078950484
H17   H     0.713076160503   0.926509358220   0.911943020584
H18   H     0.286923839497   0.426509358217   0.088056979416
H19   H     0.671301203831   0.865669033012   0.852015959889
H20   H     0.328698796169   0.365669033009   0.147984040111
H21   H     0.784037698360   0.805288522767   0.819140325665
H22   H     0.215962301640   0.305288522763   0.180859674335
H23   H     0.777795542066   0.932292256499   0.785811837683
H24   H     0.222204457934   0.432292256495   0.214188162317
H25   H     0.654221506256   0.962237116478   0.807225076171
H26   H     0.345778493744   0.462237116475   0.192774923829
H27   H     0.737898780399   0.819007276608   0.760055851545
H28   H     0.262101219601   0.319007276605   0.239944148454
H29   H     0.673017587693   0.884063090905   0.756395784897
H30   H     0.326982412308   0.384063090902   0.243604215103
H31   H     0.671807305139   0.812280831363   0.788977859982
H32   H     0.328192694861   0.312280831360   0.211022140018
H33   H     0.876682299132   0.844206871697   0.813552363440
H34   H     0.123317700868   0.344206871693   0.186447636560
H35   H     0.927314335668   0.974885558677   0.853980752932
H36   H     0.072685664332   0.474885558673   0.146019247068
H37   H     0.989401259770   0.814684279278   0.851847011719
H38   H     0.010598740230   0.314684279275   0.148152988281
H39   H     0.026619745531   0.899372218440   0.867375400778
```

H40	H	0.973380254469	0.399372218437	0.132624599222
H41	H	0.007452363282	0.035558441926	0.823774920614
H42	H	0.992547636718	0.535558441922	0.176225079386
H43	H	0.019366716855	0.975706927239	0.751827531312
H44	H	0.980633283145	0.475706927236	0.248172468688
H45	H	0.997479122189	0.135930512453	0.782101554417
H46	H	0.002520877811	0.635930512450	0.217898445583
H47	H	0.025273456562	0.125997580393	0.738712544474
H48	H	0.974726543438	0.625997580390	0.261287455526
H49	H	0.227119751686	0.980854393039	0.760189445086
H50	H	0.772880248314	0.480854393036	0.239810554914
H51	H	0.262363667666	0.993067202489	0.690944857612
H52	H	0.737636332334	0.493067202485	0.309055142387
H53	H	0.225200445481	0.909345142061	0.708130820340
H54	H	0.774799554519	0.409345142058	0.291869179660
H55	H	0.159925333904	0.045489347242	0.671888846902
H56	H	0.840074666096	0.545489347239	0.328111153098
H57	H	0.153397050046	0.948020202604	0.660215636080
H58	H	0.846602949954	0.448020202601	0.339784363920
H59	H	0.063354961711	0.012470937854	0.701743181579
H60	H	0.936645038289	0.512470937850	0.298256818421
H61	H	0.092403389713	0.918853461722	0.712312721398
H62	H	0.907596610287	0.418853461719	0.287687278602
H63	H	0.147152186018	0.145731411393	0.732623384722
H64	H	0.852847813982	0.645731411390	0.267376615278
H65	H	0.250390512465	0.234585206295	0.758776722702
H66	H	0.749609487535	0.734585206292	0.241223277298
H67	H	0.278653732321	0.310705182286	0.708464120043
H68	H	0.721346267679	0.810705182282	0.291535879957
H69	H	0.290072928724	0.212656575976	0.696913552993
H70	H	0.709927071276	0.712656575972	0.303086447007
H71	H	0.229856868771	0.297134141488	0.658507717217
H72	H	0.770143131229	0.797134141485	0.341492282783
H73	H	0.220382211953	0.366280478295	0.760031222268
H74	H	0.779617788047	0.866280478292	0.239968777732
H75	H	0.091333991576	0.389778689521	0.773806082553
H76	H	0.908666008424	0.889778689518	0.226193917447
H77	H	0.201103119755	0.501650785149	0.760152130968
H78	H	0.798896880245	0.001650785146	0.239847869032
H79	H	0.132260920609	0.442970217967	0.825467909282
H80	H	0.867739079391	0.942970217963	0.174532090718
H81	H	0.208381480244	0.414836030112	0.811700059937
H82	H	0.791618519756	0.914836030109	0.188299940063
H83	H	0.104524903700	0.572409361716	0.746164180098
H84	H	0.895475096300	0.072409361713	0.253835819902
H85	H	0.130732648099	0.602827836650	0.787331626735
H86	H	0.869267351901	0.102827836646	0.212668373265
H87	H	0.065107061205	0.538380451943	0.783973014006
H88	H	0.934892938795	0.038380451940	0.216026985994
H89	H	0.250012810847	0.550672499188	0.823292355731
H90	H	0.749987189154	0.050672499185	0.176707644269
H91	H	0.217039354011	0.505327404046	0.860133551995
H92	H	0.782960645989	0.005327404043	0.139866448005
H93	H	0.173196668334	0.577040602798	0.838242577252
H94	H	0.826803331666	0.077040602794	0.161757422748
H95	H	0.172420441512	0.426790338609	0.693538756971
H96	H	0.827579558488	0.926790338606	0.306461243029
H97	H	0.055803579021	0.497669229171	0.665486017275
H98	H	0.944196420979	0.997669229168	0.334513982725
H99	H	0.182697258330	0.452660832467	0.632570878558
H100	H	0.817302741671	0.952660832464	0.367429121442
H101	H	0.104163449761	0.423402585249	0.587439632723
H102	H	0.895836550239	0.923402585245	0.412560367277

H103	H	0.140638780199	0.512600437362	0.576963966090
H104	H	0.859361219801	0.012600437359	0.423036033910
H105	H	0.062644732347	0.510227448076	0.594822082703
H106	H	0.937355267653	0.010227448072	0.405177917297
H107	H	0.191543650423	0.594947921766	0.623596426576
H108	H	0.808456349577	0.094947921763	0.376403573423
H109	H	0.178069110771	0.573484287874	0.668287627242
H110	H	0.821930889229	0.073484287870	0.331712372758
H111	H	0.114945158135	0.609132284720	0.642257965030
H112	H	0.885054841865	0.109132284717	0.357742034970
H113	H	0.125174424110	0.305991167845	0.667633687597
H114	H	0.874825575891	0.805991167842	0.332366312403
H115	H	0.030903432326	0.244736039723	0.621186744036
H116	H	0.969096567674	0.744736039719	0.378813255964
H117	H	0.119422216164	0.164909971009	0.641535389225
H118	H	0.880577783836	0.664909971006	0.358464610774
H119	H	0.046058826510	0.121357120856	0.653166555703
H120	H	0.953941173490	0.621357120853	0.346833444297
H121	H	0.091944772063	0.170509468476	0.685249923936
H122	H	0.908055227937	0.670509468472	0.314750076064
H123	H	0.023692944137	0.289348644076	0.712622862684
H124	H	0.976307055863	0.789348644073	0.287377137316
H125	H	0.893211960804	0.239683540478	0.719020461573
H126	H	0.106788039196	0.739683540475	0.280979538427
H127	H	0.963899380691	0.260877233203	0.769745507524
H128	H	0.036100619309	0.760877233200	0.230254492476
H129	H	0.966374106096	0.360512514649	0.760696662762
H130	H	0.033625893904	0.860512514645	0.239303337238
H131	H	0.889690098968	0.344687057343	0.806511383447
H132	H	0.110309901032	0.844687057340	0.193488616553
H133	H	0.847589207723	0.368149567019	0.769015323359
H134	H	0.152410792277	0.868149567015	0.230984676641
H135	H	0.784107676478	0.274081350096	0.794338437348
H136	H	0.215892323522	0.774081350093	0.205661562651
H137	H	0.824223388556	0.222314823815	0.761547274469
H138	H	0.175776611445	0.722314823811	0.238452725531
H139	H	0.895591025170	0.218146776067	0.827047797167
H140	H	0.104408974830	0.718146776064	0.172952202833
H141	H	0.756379572214	0.138198254347	0.787803504591
H142	H	0.243620427786	0.638198254343	0.212196495409
H143	H	0.749196218990	0.064056315557	0.817147464323
H144	H	0.250803781010	0.564056315554	0.182852535676
H145	H	0.897813261864	0.109123819253	0.856143562278
H146	H	0.102186738136	0.609123819249	0.143856437722
H147	H	0.830339907935	0.050498538679	0.860130809014
H148	H	0.169660092065	0.550498538675	0.139869190986
H149	H	0.954810921853	0.431384814413	0.697761301870
H150	H	0.045189078147	0.931384814409	0.302238698130
H151	H	0.834042504403	0.501307395242	0.689614982396
H152	H	0.165957495597	0.001307395239	0.310385017604
H153	H	0.947515215526	0.517741149688	0.639556148517
H154	H	0.052484784474	0.017741149684	0.360443851483
H155	H	0.881122828810	0.580834483583	0.643798884235
H156	H	0.118877171190	0.080834483580	0.356201115764
H157	H	0.951897250758	0.633838060959	0.679211446136
H158	H	0.048102749242	0.133838060956	0.320788553864
H159	H	0.918994404562	0.390690595287	0.626554086753
H160	H	0.081005595438	0.890690595284	0.373445913247
H161	H	0.823374968718	0.408404720217	0.574426467183
H162	H	0.176625031282	0.908404720214	0.425573532817
H163	H	0.931532680371	0.289871653959	0.582078515213
H164	H	0.068467319629	0.789871653955	0.417921484786
H165	H	0.887176070637	0.304667032293	0.543812149247

H166	H	0.112823929363	0.804667032289	0.456187850753
H167	H	0.009205384875	0.327335424546	0.526288338213
H168	H	0.990794615125	0.827335424543	0.473711661787
H169	H	0.028823239962	0.424469738552	0.527935641126
H170	H	0.971176760038	0.924469738549	0.472064358874
H171	H	0.853319668200	0.262514464115	0.636234035306
H172	H	0.146680331800	0.762514464112	0.363765964694
H173	H	0.754235852973	0.155740124899	0.619257647801
H174	H	0.245764147027	0.655740124896	0.380742352199
H175	H	0.839303716742	0.125118638563	0.659751886373
H176	H	0.160696283258	0.625118638560	0.340248113626
H177	H	0.822938014508	0.207955358405	0.684675708807
H178	H	0.177061985492	0.707955358402	0.315324291193
H179	H	0.725598322178	0.214587222078	0.723340359041
H180	H	0.274401677822	0.714587222075	0.276659640959
H181	H	0.781756496304	0.007589499088	0.669634861993
H182	H	0.218243503696	0.507589499084	0.330365138007
H183	H	0.654008785195	0.138615020539	0.760952076937
H184	H	0.345991214805	0.638615020535	0.239047923063
H185	H	0.709360788195	0.930760755967	0.707453709023
H186	H	0.290639211805	0.430760755964	0.292546290977
H187	H	0.645042206972	0.997360922450	0.753354491205
H188	H	0.354957793028	0.497360922446	0.246645508795
H189	H	0.754111381818	0.324276541397	0.673413152833
H190	H	0.245888618182	0.824276541394	0.326586847166
H191	H	0.625079887691	0.306669145418	0.695520701385
H192	H	0.374920112309	0.806669145415	0.304479298614
H193	H	0.706421118992	0.452448653786	0.686999144362
H194	H	0.293578881008	0.952448653782	0.313000855637
H195	H	0.631985640108	0.447023581105	0.708984797948
H196	H	0.368014359892	0.947023581102	0.291015202052
H197	H	0.757242606889	0.492179351419	0.734789580988
H198	H	0.242757393111	0.992179351415	0.265210419011
H199	H	0.767655172401	0.440009533064	0.772502401551
H200	H	0.232344827599	0.940009533060	0.227497598448
H201	H	0.689966213570	0.402895835563	0.620988293766
H202	H	0.310033786430	0.902895835560	0.379011706234
H203	H	0.567529210028	0.460351574962	0.597161686743
H204	H	0.432470789972	0.960351574958	0.402838313256
H205	H	0.693568706287	0.422800039752	0.561637753045
H206	H	0.306431293713	0.922800039749	0.438362246955
H207	H	0.646832550591	0.486764366993	0.507652923770
H208	H	0.353167449409	0.986764366989	0.492347076230
H209	H	0.570487012537	0.477689239277	0.526585312900
H210	H	0.429512987463	0.977689239273	0.473414687100
H211	H	0.615677738562	0.393987930453	0.516993160375
H212	H	0.384322261439	0.893987930450	0.483006839624
H213	H	0.692546455193	0.569750271850	0.554122764690
H214	H	0.307453544807	0.069750271846	0.445877235310
H215	H	0.690442956252	0.541490531661	0.597992079903
H216	H	0.309557043748	0.041490531657	0.402007920097
H217	H	0.619387920795	0.571560547056	0.577060406387
H218	H	0.380612079205	0.071560547053	0.422939593612
H219	H	0.650706692105	0.282314858861	0.582269344542
H220	H	0.349293307895	0.782314858857	0.417730655458
H221	H	0.551048575817	0.215138540030	0.541202917003
H222	H	0.448951424183	0.715138540027	0.458797082997
H223	H	0.656335765881	0.158631870002	0.543802605932
H224	H	0.343664234119	0.658631869998	0.456197394068
H225	H	0.652657884974	0.150203088987	0.589772616683
H226	H	0.347342115026	0.650203088983	0.410227383317
H227	H	0.566690679527	0.214517579829	0.634777566117
H228	H	0.433309320473	0.714517579825	0.365222433883

H229	H	0.451029637069	0.125411308853	0.641979867974
H230	H	0.548970362931	0.625411308850	0.358020132026
H231	H	0.506494087572	0.242580576794	0.693236806439
H232	H	0.493505912428	0.742580576791	0.306763193561
H233	H	0.447579327725	0.173071090630	0.702835040372
H234	H	0.552420672275	0.673071090627	0.297164959628
H235	H	0.583970553920	0.132816652057	0.678990280944
H236	H	0.416029446080	0.632816652054	0.321009719056
H237	H	0.562773750728	0.146246375135	0.722373370999
H238	H	0.437226249272	0.646246375132	0.277626629001
H239	H	0.494510482811	0.022405940312	0.680072040544
H240	H	0.505489517189	0.522405940309	0.319927959456
H241	H	0.571319259494	0.008047852375	0.696308688571
H242	H	0.428680740506	0.508047852372	0.303691311428
H243	H	0.535663904166	0.000813633897	0.753327437867
H244	H	0.464336095835	0.500813633893	0.246672562133
H245	H	0.402829783518	0.019325511888	0.696442319154
H246	H	0.597170216482	0.519325511885	0.303557680846
H247	H	0.351512244426	0.041516643797	0.731427523717
H248	H	0.648487755574	0.541516643794	0.268572476283
H249	H	0.464878672375	0.012705545566	0.796597530100
H250	H	0.535121327625	0.512705545562	0.203402469900
H251	H	0.384653729660	0.994253583287	0.787342737086
H252	H	0.615346270340	0.494253583284	0.212657262914
H253	H	0.474508366571	0.327350559847	0.634018951030
H254	H	0.525491633429	0.827350559843	0.365981048970
H255	H	0.369619805396	0.387040343567	0.662675450879
H256	H	0.630380194604	0.887040343564	0.337324549121
H257	H	0.455230371642	0.434646790014	0.607698905629
H258	H	0.544769628358	0.934646790010	0.392301094371
H259	H	0.378984793966	0.455869615765	0.592861662842
H260	H	0.621015206034	0.955869615762	0.407138337157
H261	H	0.436694160365	0.512021692036	0.660685967799
H262	H	0.563305839635	0.012021692033	0.339314032201
H263	H	0.476861834945	0.571507728548	0.604084183725
H264	H	0.523138165056	0.071507728545	0.395915816275
H265	H	0.437915909607	0.639691617064	0.631226566104
H266	H	0.562084090393	0.139691617060	0.368773433896
H267	H	0.398103086433	0.600758927392	0.594778504610
H268	H	0.601896913567	0.100758927388	0.405221495390
H269	H	0.339265988767	0.592716619867	0.668517725214
H270	H	0.660734011233	0.092716619864	0.331482274786
H271	H	0.317591699946	0.494386897207	0.668350974035
H272	H	0.682408300054	0.994386897203	0.331649025965
H273	H	0.304790612688	0.550512046170	0.630887504918
H274	H	0.695209387312	0.050512046167	0.369112495082
H275	H	0.216982633862	0.357280646586	0.578314163426
H276	H	0.783017366138	0.857280646582	0.421685836573
H277	H	0.228619328549	0.267344661382	0.520138382835
H278	H	0.771380671451	0.767344661378	0.479861617165
H279	H	0.267707958702	0.357661587877	0.522127646730
H280	H	0.732292041298	0.857661587873	0.477872353269
H281	H	0.327937236504	0.201313813361	0.539192886785
H282	H	0.672062763496	0.701313813358	0.460807113214
H283	H	0.354844607179	0.270917848120	0.508668932493
H284	H	0.645155392821	0.770917848117	0.491331067507
H285	H	0.401788313999	0.271065990641	0.575201762653
H286	H	0.598211686001	0.771065990638	0.424798237346
H287	H	0.386172250095	0.359535145817	0.554191508634
H288	H	0.613827749905	0.859535145813	0.445808491366
H289	H	0.291546732708	0.205272453633	0.616083801839
H290	H	0.708453267292	0.705272453629	0.383916198161
H291	H	0.216719421965	0.121626067824	0.646592485724

H292	H	0.783280578035	0.621626067821	0.353407514276
H293	H	0.177749095842	0.104920925537	0.606535622996
H294	H	0.822250904158	0.604920925533	0.393464377004
H295	H	0.336333195248	0.103650422716	0.588028238448
H296	H	0.663666804752	0.603650422713	0.411971761552
H297	H	0.332651781228	0.942345742172	0.577220006402
H298	H	0.667348218772	0.442345742169	0.422779993598
H299	H	0.452959896653	0.035849669008	0.587448441270
H300	H	0.547040103347	0.535849669004	0.412551558730
H301	H	0.426340519817	0.105589538688	0.548289943764
H302	H	0.573659480183	0.605589538685	0.451710056236
H303	H	0.466311151328	0.897684660328	0.577453303353
H304	H	0.533688848672	0.397684660325	0.422546696646
H305	H	0.492738152669	0.948269361141	0.539858079402
H306	H	0.507261847331	0.448269361138	0.460141920598
H307	H	0.417773004075	0.900752669432	0.539688983338
H308	H	0.582226995925	0.400752669429	0.460311016662
H309	H	0.370569044263	0.884438334640	0.688616816098
H310	H	0.629430955738	0.384438334637	0.311383183902
H311	H	0.392507508647	0.723142381681	0.659664403036
H312	H	0.607492491353	0.223142381677	0.340335596964
H313	H	0.344838645785	0.749918207854	0.695892824843
H314	H	0.655161354215	0.249918207851	0.304107175157
H315	H	0.286961427393	0.715782048113	0.634969557259
H316	H	0.713038572607	0.215782048110	0.365030442741
H317	H	0.261141669870	0.799785304403	0.657864561948
H318	H	0.738858330130	0.299785304400	0.342135438052
H319	H	0.352870840809	0.792909948404	0.595766304371
H320	H	0.647129159191	0.292909948401	0.404233695629
H321	H	0.288240343389	0.858165041623	0.603603930992
H322	H	0.711759656611	0.358165041619	0.396396069008
H323	H	0.446936676864	0.847334896733	0.722961381641
H324	H	0.553063323137	0.347334896729	0.277038618359
H325	H	0.568250850817	0.883114517139	0.719626082982
H326	H	0.431749149183	0.383114517135	0.280373917018
H327	H	0.515625215454	0.732408904278	0.749607515616
H328	H	0.484374784546	0.232408904275	0.250392484384
H329	H	0.591546667402	0.768112657230	0.759485860689
H330	H	0.408453332598	0.268112657227	0.240514139311
H331	H	0.474313449549	0.868535718664	0.774502279857
H332	H	0.525686550451	0.368535718661	0.225497720143
H333	H	0.493756881282	0.790030153925	0.801645105697
H334	H	0.506243118718	0.290030153921	0.198354894303
H335	H	0.525665637060	0.690126435242	0.689804797377
H336	H	0.474334362940	0.190126435239	0.310195202623
H337	H	0.657555791096	0.655971712518	0.678808114843
H338	H	0.342444208904	0.155971712515	0.321191885157
H339	H	0.572864891833	0.552459453886	0.686934998444
H340	H	0.427135108167	0.052459453882	0.313065001556
H341	H	0.620766288282	0.538013402480	0.649388238060
H342	H	0.379233711718	0.038013402476	0.350611761940
H343	H	0.542705900821	0.575148802615	0.645141333201
H344	H	0.457294099179	0.075148802612	0.354858666799
H345	H	0.542642373571	0.728601668040	0.619244866901
H346	H	0.457357626429	0.228601668037	0.380755133099
H347	H	0.624748495275	0.699175375848	0.558367640391
H348	H	0.375251504725	0.199175375845	0.441632359609
H349	H	0.508661286699	0.800830654327	0.572945157893
H350	H	0.491338713302	0.300830654323	0.427054842107
H351	H	0.548054389731	0.802479402190	0.532521380159
H352	H	0.451945610269	0.302479402186	0.467478619841
H353	H	0.489657942082	0.657269987207	0.564618731054
H354	H	0.510342057919	0.157269987203	0.435381268946

H355	H	0.403912959467	0.752554913411	0.553495987965
H356	H	0.596087040534	0.252554913408	0.446504012035
H357	H	0.399386359923	0.676165954751	0.523150378403
H358	H	0.600613640077	0.176165954747	0.476849621597
H359	H	0.432058273096	0.766622069179	0.510544413641
H360	H	0.567941726904	0.266622069176	0.489455586358
H361	H	0.499039195318	0.603380933551	0.504406070925
H362	H	0.500960804682	0.103380933548	0.495593929075
H363	H	0.574401097157	0.628001074138	0.520820453507
H364	H	0.425598902843	0.128001074135	0.479179546493
H365	H	0.536760595589	0.689516859859	0.490517175637
H366	H	0.463239404411	0.189516859856	0.509482824363
H367	H	0.601485906625	0.850427376642	0.619394132198
H368	H	0.398514093375	0.350427376638	0.380605867802
H369	H	0.688252042503	0.948892899529	0.580508429915
H370	H	0.311747957497	0.448892899525	0.419491570085
H371	H	0.634930354506	0.963677599734	0.654454291533
H372	H	0.365069645494	0.463677599731	0.345545708467
H373	H	0.678254351218	0.036048005995	0.632008651158
H374	H	0.321745648782	0.536048005992	0.367991348842
H375	H	0.710846249446	0.804243471993	0.640292016279
H376	H	0.289153750554	0.304243471990	0.359707983721
H377	H	0.842163172011	0.839437654197	0.656275572625
H378	H	0.157836827989	0.339437654193	0.343724427375
H379	H	0.779339108041	0.747041257048	0.691858915730
H380	H	0.220660891959	0.247041257045	0.308141084270
H381	H	0.846599763012	0.700630554402	0.672664459426
H382	H	0.153400236988	0.200630554399	0.327335540574
H383	H	0.769148556224	0.685593843906	0.655081247891
H384	H	0.230851443776	0.185593843903	0.344918752108
H385	H	0.775761328229	0.712944587543	0.589471772729
H386	H	0.224238671771	0.212944587539	0.410528227271
H387	H	0.904028857344	0.694810132940	0.560334366699
H388	H	0.095971142656	0.194810132937	0.439665633300
H389	H	0.850018974823	0.648428207063	0.508024082590
H390	H	0.149981025177	0.148428207060	0.491975917410
H391	H	0.739766280451	0.640173021868	0.511158894275
H392	H	0.260233719549	0.140173021865	0.488841105725
H393	H	0.876019643694	0.552316654676	0.553319710579
H394	H	0.123980356306	0.052316654673	0.446680289421
H395	H	0.801930224909	0.528995861136	0.534469694265
H396	H	0.198069775091	0.028995861133	0.465530305735
H397	H	0.803968884943	0.572468111043	0.576197753843
H398	H	0.196031115057	0.072468111040	0.423802246157
H399	H	0.781398646902	0.833791908700	0.545879567127
H400	H	0.218601353098	0.333791908697	0.454120432873
H401	H	0.834175752263	0.904493598807	0.487554514534
H402	H	0.165824247737	0.404493598803	0.512445485466
H403	H	0.731691623562	0.933657938697	0.521765767534
H404	H	0.268308376438	0.433657938693	0.478234232466
H405	H	0.777450295445	0.984553907553	0.551959700475
H406	H	0.222549704556	0.484553907550	0.448040299525
H407	H	0.752256099154	0.982211418466	0.454142542537
H408	H	0.247743900846	0.482211418463	0.545857457463
H409	H	0.777011204366	0.119693217808	0.546002634135
H410	H	0.222988795634	0.619693217805	0.453997365865
H411	H	0.738872463698	0.100627748576	0.419708543514
H412	H	0.261127536302	0.600627748573	0.580291456486
H413	H	0.763993622193	0.238283026341	0.511631415691
H414	H	0.236006377807	0.738283026337	0.488368584309
H415	H	0.756857508358	0.233556959866	0.420620459252
H416	H	0.243142491642	0.733556959863	0.579379540747
H417	H	0.894213877786	0.914338331793	0.572121894610

H418	H	0.105786122214	0.414338331789	0.427878105389
H419	H	0.987229691591	0.033282174193	0.548418547657
H420	H	0.012770308409	0.533282174189	0.451581452343
H421	H	0.979436568506	0.080394295641	0.610406995221
H422	H	0.020563431494	0.580394295638	0.389593004779
H423	H	0.870808432487	0.984121865889	0.619242106078
H424	H	0.129191567513	0.484121865885	0.380757893922
H425	H	0.908322825874	0.135512310712	0.561552034570
H426	H	0.091677174126	0.635512310709	0.438447965430
H427	H	0.881016585546	0.149478051409	0.604861362012
H428	H	0.118983414454	0.649478051405	0.395138637988
H429	H	0.846554262849	0.076319037813	0.578455965771
H430	H	0.153445737151	0.576319037810	0.421544034229
H431	H	0.981614778581	0.850513928402	0.583247357622
H432	H	0.018385221419	0.350513928398	0.416752642378
H433	H	0.065571520647	0.777780921987	0.607296788423
H434	H	0.934428479353	0.277780921984	0.392703211577
H435	H	0.112739844708	0.862665547030	0.610624283032
H436	H	0.887260155292	0.362665547027	0.389375716968
H437	H	0.965267555627	0.902784367350	0.642020458822
H438	H	0.034732444373	0.402784367347	0.357979541178
H439	H	0.001048548262	0.882749002365	0.715227136771
H440	H	0.998951451738	0.382749002362	0.284772863229
H441	H	0.871507645049	0.921660950112	0.683085823943
H442	H	0.128492354951	0.421660950108	0.316914176057
H443	H	0.890377051626	0.923911591023	0.727730760129
H444	H	0.109622948374	0.423911591020	0.272269239870
H445	H	0.920487308933	0.815958459022	0.750369857799
H446	H	0.079512691067	0.315958459018	0.249630142201
H447	H	0.906054895620	0.656146770550	0.723516441315
H448	H	0.093945104380	0.156146770547	0.276483558685
H449	H	0.967561062594	0.606073508170	0.766348359182
H450	H	0.032438937406	0.106073508167	0.233651640818
H451	H	0.948654517554	0.752034186644	0.809890648140
H452	H	0.051345482446	0.252034186641	0.190109351860
H453	H	0.912473913364	0.662313148782	0.817709924287
H454	H	0.087526086636	0.162313148779	0.182290075713
H455	H	0.068497192214	0.681963804041	0.768533190157
H456	H	0.931502807786	0.181963804037	0.231466809843
H457	H	0.027780680420	0.766262908281	0.757472738889
H458	H	0.972219319580	0.266262908278	0.242527261111
H459	H	0.034708908648	0.690037878405	0.726935683176
H460	H	0.965291091352	0.190037878402	0.273064316824
H461	H	0.021429849088	0.604100209385	0.829566206002
H462	H	0.978570150912	0.104100209382	0.170433793998
H463	H	0.998721255384	0.677968772071	0.858811865653
H464	H	0.001278744616	0.177968772067	0.141188134347
H465	H	0.055138123675	0.697245098492	0.825471723048
H466	H	0.944861876325	0.197245098489	0.174528276951
H467	H	0.842316028925	0.577934155939	0.764834153591
H468	H	0.157683971075	0.077934155935	0.235165846409
H469	H	0.779259287034	0.673572461777	0.816266273751
H470	H	0.220740712966	0.173572461773	0.183733726248
H471	H	0.702811221599	0.534582980480	0.794310905322
H472	H	0.297188778401	0.034582980477	0.205689094678
H473	H	0.679603511663	0.693539197204	0.763275517739
H474	H	0.320396488337	0.193539197201	0.236724482261
H475	H	0.710637719191	0.614138401403	0.740721820407
H476	H	0.289362280809	0.114138401399	0.259278179593
H477	H	0.686861073602	0.601618772681	0.852237133683
H478	H	0.313138926398	0.101618772678	0.147762866317
H479	H	0.617695720343	0.596613272180	0.826718608443
H480	H	0.382304279657	0.096613272177	0.173281391556

H481	H	0.659876438179	0.685050027457	0.829155563684
H482	H	0.340123561822	0.185050027454	0.170844436316
H483	H	0.611370312703	0.536807984241	0.754100572951
H484	H	0.388629687297	0.036807984238	0.245899427049
H485	H	0.593578065003	0.622902394037	0.731096675343
H486	H	0.406421934997	0.122902394034	0.268903324657
H487	H	0.577919912748	0.617241534111	0.775882265369
H488	H	0.422080087252	0.117241534108	0.224117734631
H489	H	0.795954838722	0.623384683294	0.867215624516
H490	H	0.204045161278	0.123384683291	0.132784375484
H491	H	0.829354203947	0.457599190378	0.869514029184
H492	H	0.170645796053	0.957599190374	0.130485970816
H493	H	0.877879836653	0.487690578384	0.929920714060
H494	H	0.122120163347	0.987690578380	0.070079285940
H495	H	0.892884512977	0.648454799693	0.898211307995
H496	H	0.107115487024	0.148454799689	0.101788692005
H497	H	0.836039263046	0.625569470180	0.930277723286
H498	H	0.163960736954	0.125569470177	0.069722276714
H499	H	0.985161839693	0.515070878689	0.900469648299
H500	H	0.014838160307	0.015070878686	0.099530351701
H501	H	0.943456595480	0.533239826927	0.861404080088
H502	H	0.056543404520	0.033239826924	0.138595919912
H503	H	0.941199587877	0.440042337969	0.880470822889
H504	H	0.058800412123	0.940042337965	0.119529177110
H505	H	0.920890181109	0.592149867910	0.972389643392
H506	H	0.079109818891	0.092149867907	0.027610356608
H507	H	0.934774169766	0.686172383865	0.954073058505
H508	H	0.065225830234	0.186172383861	0.045926941495
H509	H	0.981171898945	0.607978154110	0.940693083198
H510	H	0.018828101055	0.107978154107	0.059306916802
H511	H	0.651610916249	0.441220930428	0.926304988833
H512	H	0.348389083751	0.941220930425	0.073695011167
H513	H	0.669456049419	0.322597995403	0.974848958621
H514	H	0.330543950581	0.822597995399	0.025151041379
H515	H	0.660787127667	0.308189506220	0.929844860891
H516	H	0.339212872333	0.808189506217	0.070155139109
H517	H	0.786430744675	0.317333844336	0.969869454274
H518	H	0.213569255325	0.817333844333	0.030130545725
H519	H	0.764092569105	0.248576930687	0.938222330697
H520	H	0.235907430896	0.748576930684	0.061777669303
H521	H	0.839195085164	0.372108668247	0.921505318643
H522	H	0.160804914836	0.872108668244	0.078494681357
H523	H	0.780691344984	0.340165818829	0.891517022019
H524	H	0.219308655017	0.840165818825	0.108482977981
H525	H	0.781968585758	0.482624703979	0.980966575655
H526	H	0.218031414242	0.982624703975	0.019033424345
H527	H	0.692633547332	0.562672593580	0.027487216636
H528	H	0.307366452668	0.062672593577	0.972512783364
H529	H	0.773816124476	0.538480347418	0.035864131961
H530	H	0.226183875524	0.038480347414	0.964135868039
H531	H	0.818349469660	0.651601991983	0.047146829793
H532	H	0.181650530340	0.151601991980	0.952853170207
H533	H	0.817857046706	0.774088520642	0.994227199273
H534	H	0.182142953295	0.274088520639	0.005772800727
H535	H	0.719438982848	0.806419967760	0.025799559377
H536	H	0.280561017152	0.306419967757	0.974200440623
H537	H	0.779896238236	0.872335542247	0.038573643762
H538	H	0.220103761764	0.372335542243	0.961426356238
H539	H	0.758101558165	0.795023553381	0.066585034178
H540	H	0.241898441835	0.295023553378	0.933414965822
H541	H	0.918608174493	0.695340465845	0.003660649748
H542	H	0.081391825507	0.195340465842	0.996339350252
H543	H	0.998624620510	0.789111196243	0.050622576968

H544	H	0.001375379490	0.289111196239	0.949377423032
H545	H	0.029266969721	0.627764404421	0.022969580621
H546	H	0.970733030279	0.127764404417	0.977030419379
H547	H	0.948007271617	0.641722822026	0.069981775297
H548	H	0.051992728383	0.141722822022	0.930018224703
H549	H	0.126460181974	0.704800024399	0.035688346779
H550	H	0.873539818026	0.204800024396	0.964311653221
H551	H	0.115811339233	0.630152970269	0.067025315517
H552	H	0.884188660767	0.130152970266	0.932974684483
H553	H	0.095494449433	0.726021901897	0.078437296804
H554	H	0.904505550567	0.226021901893	0.921562703196
H555	H	0.015198647156	0.884404288320	0.009127869354
H556	H	0.984801352844	0.384404288316	0.990872130646
H557	H	0.059112604169	0.834889008864	0.939931274309
H558	H	0.940887395831	0.334889008861	0.060068725691
H559	H	0.039097899920	0.999192086883	0.967185228331
H560	H	0.960902100080	0.499192086879	0.032814771669
H561	H	0.066582299264	0.973476642290	0.924963725620
H562	H	0.933417700736	0.473476642287	0.075036274380
H563	H	0.251876872340	0.863837962216	0.988329200197
H564	H	0.748123127660	0.363837962213	0.011670799803
H565	H	0.311161233459	0.752425951167	0.942396347247
H566	H	0.688838766541	0.252425951164	0.057603652753
H567	H	0.277712323874	0.731720313743	0.983939232178
H568	H	0.722287676126	0.231720313739	0.016060767822
H569	H	0.229571124333	0.680485522643	0.918189410242
H570	H	0.770428875667	0.180485522639	0.081810589758
H571	H	0.186820548124	0.676960818018	0.958804522053
H572	H	0.813179451876	0.176960818015	0.041195477947
H573	H	0.180394866151	0.800625730408	0.902587913692
H574	H	0.819605133849	0.300625730404	0.097412086308
H575	H	0.117802401523	0.759536167554	0.928591479150
H576	H	0.882197598477	0.259536167550	0.071408520850
H577	H	0.362387587630	0.894368529399	0.966528559506
H578	H	0.637612412370	0.394368529396	0.033471440494
H579	H	0.356936871541	0.022288383500	0.925105291661
H580	H	0.643063128459	0.522288383496	0.074894708339
H581	H	0.427085175119	0.969348915013	0.933593803213
H582	H	0.572914824881	0.469348915010	0.066406196787
H583	H	0.355677190841	0.836208238825	0.888831004957
H584	H	0.644322809159	0.336208238821	0.111168995043
H585	H	0.410209435992	0.885252276044	0.822330181444
H586	H	0.589790564008	0.385252276041	0.177669818556
H587	H	0.367208765183	0.755335237634	0.803834432848
H588	H	0.632791234817	0.255335237631	0.196165567152
H589	H	0.424334226609	0.750877523851	0.837126680490
H590	H	0.575665773392	0.250877523847	0.162873319510
H591	H	0.260573125613	0.828681857556	0.856058252036
H592	H	0.739426874387	0.328681857553	0.143941747963
H593	H	0.200091373597	0.929097378208	0.802241384408
H594	H	0.799908626403	0.429097378204	0.197758615592
H595	H	0.140307538579	0.796307255734	0.843261368543
H596	H	0.859692461421	0.296307255730	0.156738631457
H597	H	0.100594288821	0.854638568135	0.812776063693
H598	H	0.899405711179	0.354638568131	0.187223936307
H599	H	0.227804602382	0.705384807549	0.823061364925
H600	H	0.772195397618	0.205384807546	0.176938635075
H601	H	0.125309665070	0.844206291433	0.749774446313
H602	H	0.874690334931	0.344206291430	0.250225553687
H603	H	0.276896049650	0.632921369330	0.775178391819
H604	H	0.723103950351	0.132921369327	0.224821608181
H605	H	0.168290339039	0.767346798367	0.701773154810
H606	H	0.831709660961	0.267346798363	0.298226845190

H607	H	0.232199085351	0.663494224310	0.687705831582
H608	H	0.767800914649	0.163494224306	0.312294168418
H609	H	0.241054912938	0.024819106732	0.835992202714
H610	H	0.758945087062	0.524819106729	0.164007797286
H611	H	0.210967567796	0.038120132417	0.906941098178
H612	H	0.789032432204	0.538120132413	0.093058901822
H613	H	0.307744982838	0.094108069767	0.876837891558
H614	H	0.692255017162	0.594108069763	0.123162108442
H615	H	0.266988018608	0.163921939374	0.902822628003
H616	H	0.733011981392	0.663921939371	0.097177371997
H617	H	0.258276675413	0.164966266547	0.856850819416
H618	H	0.741723324587	0.664966266544	0.143149180584
H619	H	0.121880110452	0.110654317165	0.836978492654
H620	H	0.878119889548	0.610654317162	0.163021507346
H621	H	0.068405133398	0.228270687678	0.885803176482
H622	H	0.931594866602	0.728270687674	0.114196823518
H623	H	0.091467816309	0.241934361313	0.806899972513
H624	H	0.908532183691	0.741934361309	0.193100027487
H625	H	0.064327069911	0.315502560316	0.835464267078
H626	H	0.935672930089	0.815502560313	0.164535732922
H627	H	0.146529204762	0.321156566287	0.891376326779
H628	H	0.853470795238	0.821156566283	0.108623673221
H629	H	0.223774570705	0.329941195644	0.875244317314
H630	H	0.776225429295	0.829941195641	0.124755682685
C1	C	0.669048416985	0.773316205126	0.903978490027
C2	C	0.330951583015	0.273316205122	0.096021509973
C3	C	0.720386258740	0.778612711599	0.875940724412
C4	C	0.279613741260	0.278612711596	0.124059275588
C5	C	0.691306001128	0.817407063196	0.937210171067
C6	C	0.308693998872	0.317407063193	0.062789828933
C7	C	0.683702918126	0.904059695902	0.933369417756
C8	C	0.316297081874	0.404059695899	0.066630582244
C9	C	0.764564485202	0.858324297621	0.830111288910
C10	C	0.235435514798	0.358324297617	0.169888711090
C11	C	0.819272347691	0.900964061514	0.847771878587
C12	C	0.180727652309	0.400964061510	0.152228121413
C13	C	0.737021509070	0.906261210017	0.799698665382
C14	C	0.262978490930	0.406261210013	0.200301334617
C15	C	0.702554906941	0.852394468981	0.774672751705
C16	C	0.297445093059	0.352394468978	0.225327248295
C17	C	0.936516285810	0.919823117015	0.840962497825
C18	C	0.063483714190	0.419823117012	0.159037502175
C19	C	0.969940676551	0.933448264646	0.805911498204
C20	C	0.030059323449	0.433448264642	0.194088501796
C21	C	0.980312197862	0.870911083841	0.863997692831
C22	C	0.019687802138	0.370911083838	0.136002307169
C23	C	0.031881060352	0.020733125233	0.770374776987
C24	C	0.968118939648	0.520733125230	0.229625223013
C25	C	0.104077147627	0.030915272805	0.770308783300
C26	C	0.895922852373	0.530915272802	0.229691216699
C27	C	0.999575088459	0.096568086191	0.759792006318
C28	C	0.000424911541	0.596568086187	0.240207993681
C29	C	0.205020081229	0.011636737783	0.738515086904
C30	C	0.794979918771	0.511636737780	0.261484913096
C31	C	0.233372976833	0.092282995653	0.739837880086
C32	C	0.766627023167	0.592282995650	0.260162119914
C33	C	0.218960022245	0.971435110007	0.703486395359
C34	C	0.781039977755	0.471435110003	0.296513604641
C35	C	0.158987800900	0.986724335042	0.682305339972
C36	C	0.841012199100	0.486724335039	0.317694660028
C37	C	0.105751436117	0.979555517173	0.708886103154
C38	C	0.894248563883	0.479555517169	0.291113896846
C39	C	0.218167746827	0.230649349752	0.736471752801

C40	C	0.781832253173	0.730649349748	0.263528247199
C41	C	0.161395191361	0.283484119851	0.742307245710
C42	C	0.838604808639	0.783484119847	0.257692754289
C43	C	0.253702381493	0.255805597103	0.703520856935
C44	C	0.746297618507	0.755805597100	0.296479143065
C45	C	0.127979849768	0.414643841451	0.756614097167
C46	C	0.872020150232	0.914643841448	0.243385902833
C47	C	0.091610055069	0.433979236646	0.723265380776
C48	C	0.908389944931	0.933979236642	0.276734619223
C49	C	0.157492556087	0.485698278014	0.774735854485
C50	C	0.842507443914	0.985698278011	0.225264145515
C51	C	0.175667180407	0.464279808977	0.812291145632
C52	C	0.824332819593	0.964279808973	0.187708854368
C53	C	0.111892995502	0.553585910199	0.773003932555
C54	C	0.888107004498	0.053585910195	0.226996067445
C55	C	0.205461897146	0.527889395071	0.834239877685
C56	C	0.794538102854	0.027889395068	0.165760122315
C57	C	0.093658725509	0.455288220204	0.660579990526
C58	C	0.906341274491	0.955288220200	0.339420009474
C59	C	0.057565948429	0.383247485487	0.648883427738
C60	C	0.942434051571	0.883247485483	0.351116572262
C61	C	0.139108872202	0.488103524748	0.632461102965
C62	C	0.860891127798	0.988103524744	0.367538897035
C63	C	0.110013361458	0.483410449502	0.596055819568
C64	C	0.889986638542	0.983410449499	0.403944180432
C65	C	0.156967663515	0.571097030707	0.642259863931
C66	C	0.843032336485	0.071097030704	0.357740136068
C67	C	0.043227847487	0.245486587315	0.648842711955
C68	C	0.956772152513	0.745486587312	0.351157288045
C69	C	0.979570630562	0.252250857443	0.668191303736
C70	C	0.020429369438	0.752250857439	0.331808696264
C71	C	0.077303930920	0.170856528717	0.657992249316
C72	C	0.922696069080	0.670856528713	0.342007750684
C73	C	0.922550606168	0.291783066580	0.720676896107
C74	C	0.077449393832	0.791783066577	0.279323103893
C75	C	0.881159962104	0.354080395942	0.703320245441
C76	C	0.118840037896	0.854080395938	0.296679754559
C77	C	0.936039479509	0.308985643988	0.759107227539
C78	C	0.063960520491	0.808985643985	0.240892772461
C79	C	0.875730110829	0.322215413123	0.780972591849
C80	C	0.124269889171	0.822215413119	0.219027408151
C81	C	0.831439556722	0.252937114990	0.786250109234
C82	C	0.168560443278	0.752937114987	0.213749890766
C83	C	0.825600795076	0.138466745775	0.824689483362
C84	C	0.174399204924	0.638466745771	0.175310516637
C85	C	0.871042938059	0.476413344105	0.672640529977
C86	C	0.128957061941	0.976413344102	0.327359470023
C87	C	0.835284722374	0.439790544510	0.641917461819
C88	C	0.164715277626	0.939790544506	0.358082538181
C89	C	0.912809979855	0.541696569324	0.658205112704
C90	C	0.087190020145	0.041696569320	0.341794887296
C91	C	0.848341704289	0.363164253864	0.588887841256
C92	C	0.151658295711	0.863164253860	0.411112158744
C93	C	0.797848478754	0.300444560274	0.596305418399
C94	C	0.202151521247	0.800444560271	0.403694581601
C95	C	0.905006128587	0.333550660599	0.567273119476
C96	C	0.094993871413	0.833550660596	0.432726880523
C97	C	0.950694122431	0.399281655579	0.556804748062
C98	C	0.049305877569	0.899281655576	0.443195251938
C99	C	0.767196690947	0.199548322013	0.638600828348
C100	C	0.232803309054	0.699548322009	0.361399171652
C101	C	0.704634412469	0.237839425728	0.649618884513
C102	C	0.295365587531	0.737839425724	0.350381115487

C103	C	0.800802253886	0.161543421980	0.669817396645
C104	C	0.199197746114	0.661543421977	0.330182603355
C105	C	0.758151688060	0.115764757067	0.693451098725
C106	C	0.241848311941	0.615764757063	0.306548901275
C107	C	0.722369678286	0.152442136657	0.719535858251
C108	C	0.277630321715	0.652442136653	0.280464141749
C109	C	0.753595259308	0.035851368443	0.689760087326
C110	C	0.246404740692	0.535851368440	0.310239912674
C111	C	0.681909814891	0.109559511727	0.740820031417
C112	C	0.318090185109	0.609559511724	0.259179968583
C113	C	0.713115101699	0.992952757411	0.711089047374
C114	C	0.286884898301	0.492952757407	0.288910952626
C115	C	0.676897539902	0.029910525247	0.736583261664
C116	C	0.323102460098	0.529910525244	0.263416738336
C117	C	0.654611605988	0.345867549420	0.679834461874
C118	C	0.345388394012	0.845867549416	0.320165538126
C119	C	0.611659110938	0.374249481082	0.650362528401
C120	C	0.388340889062	0.874249481078	0.349637471599
C121	C	0.675072724892	0.414139430949	0.702145747311
C122	C	0.324927275108	0.914139430946	0.297854252689
C123	C	0.706155100720	0.391244802803	0.736403422362
C124	C	0.293844899281	0.891244802799	0.263596577638
C125	C	0.605615193983	0.419459449708	0.590389442052
C126	C	0.394384806017	0.919459449704	0.409610557948
C127	C	0.569989417612	0.348680236140	0.576877067838
C128	C	0.430010582388	0.848680236136	0.423122932161
C129	C	0.649117931079	0.456619187978	0.562560083168
C130	C	0.350882068921	0.956619187975	0.437439916832
C131	C	0.618869021309	0.453519957637	0.526583921443
C132	C	0.381130978691	0.953519957634	0.473416078557
C133	C	0.663985322510	0.539113766767	0.573573124437
C134	C	0.336014677490	0.039113766763	0.426426875563
C135	C	0.572819830231	0.210259491150	0.566864670315
C136	C	0.427180169769	0.710259491146	0.433135329685
C137	C	0.515684733230	0.192432954295	0.590914997880
C138	C	0.484315266770	0.692432954292	0.409085002120
C139	C	0.624377472452	0.147890547199	0.565981293483
C140	C	0.375622527548	0.647890547196	0.434018706516
C141	C	0.470103509773	0.182437148266	0.648722470540
C142	C	0.529896490227	0.682437148263	0.351277529460
C143	C	0.413783282914	0.237927883094	0.643976924917
C144	C	0.586216717086	0.737927883090	0.356023075083
C145	C	0.490630211764	0.183596831767	0.687073760271
C146	C	0.509369788236	0.683596831764	0.312926239728
C147	C	0.543888174175	0.127301102268	0.697273480280
C148	C	0.456111825825	0.627301102264	0.302726519720
C149	C	0.527456012329	0.041065002889	0.700388106962
C150	C	0.472543987671	0.541065002886	0.299611893038
C151	C	0.442516412685	0.016954672345	0.745110995336
C152	C	0.557483587315	0.516954672341	0.254889004664
C153	C	0.380978748689	0.373167157621	0.635465617822
C154	C	0.619021251311	0.873167157617	0.364534382178
C155	C	0.316213949430	0.351867454360	0.620263729994
C156	C	0.683786050570	0.851867454357	0.379736270006
C157	C	0.406514285750	0.446082502588	0.616712997811
C158	C	0.593485714250	0.946082502585	0.383287002189
C159	C	0.404665269644	0.520490502952	0.638331990057
C160	C	0.595334730356	0.020490502949	0.361668009943
C161	C	0.430898445837	0.586613954134	0.616061267223
C162	C	0.569101554163	0.086613954131	0.383938732777
C163	C	0.338221226928	0.540551816366	0.652348922995
C164	C	0.661778773072	0.040551816363	0.347651077005
C165	C	0.248989884212	0.308826310073	0.572757576497

C166	C	0.751010115788	0.808826310069	0.427242423503
C167	C	0.214859468763	0.236197988022	0.586098852249
C168	C	0.785140531237	0.736197988019	0.413901147751
C169	C	0.264639701068	0.300319271272	0.533989673920
C170	C	0.735360298933	0.800319271268	0.466010326080
C171	C	0.330672510494	0.263573900742	0.533726039802
C172	C	0.669327489507	0.763573900739	0.466273960198
C173	C	0.364494404088	0.305370705825	0.563229428891
C174	C	0.635505595912	0.805370705822	0.436770571109
C175	C	0.224273234959	0.115447620945	0.618449298602
C176	C	0.775726765041	0.615447620942	0.381550701398
C177	C	0.264319562004	0.043679307684	0.612926934845
C178	C	0.735680437996	0.543679307681	0.387073065155
C179	C	0.360510509220	0.985084666728	0.591630483995
C180	C	0.639489490780	0.485084666724	0.408369516005
C181	C	0.380905541646	0.952657199607	0.626899670828
C182	C	0.619094458354	0.452657199603	0.373100329172
C183	C	0.419946610758	0.006416345380	0.569977375319
C184	C	0.580053389242	0.506416345377	0.430022624681
C185	C	0.450887066438	0.933950775067	0.555750747223
C186	C	0.549112933562	0.433950775064	0.444249252777
C187	C	0.389106083150	0.847209410044	0.667707774206
C188	C	0.610893916850	0.347209410041	0.332292225794
C189	C	0.461593987607	0.841678461553	0.671003227916
C190	C	0.538406012393	0.341678461550	0.328996772084
C191	C	0.358532302293	0.766367905044	0.669414798501
C192	C	0.641467697707	0.266367905041	0.330585201499
C193	C	0.302005830969	0.772508606963	0.644717536184
C194	C	0.697994169032	0.272508606960	0.355282463816
C195	C	0.326531660225	0.825334165325	0.615754472084
C196	C	0.673468339775	0.325334165321	0.384245527916
C197	C	0.545228007399	0.826590698860	0.717182416486
C198	C	0.454771992601	0.326590698856	0.282817583514
C199	C	0.588221886223	0.781119562346	0.692050624318
C200	C	0.411778113777	0.281119562342	0.307949375682
C201	C	0.543078751191	0.785967477475	0.752841635087
C202	C	0.456921248809	0.285967477471	0.247158364913
C203	C	0.513730426001	0.831853716214	0.783315173511
C204	C	0.486269573999	0.331853716210	0.216684826489
C205	C	0.562175773074	0.880799110606	0.803449812011
C206	C	0.437824226926	0.380799110602	0.196550187989
C207	C	0.612825400625	0.657304491894	0.664215562470
C208	C	0.387174599375	0.157304491891	0.335784437530
C209	C	0.631799678964	0.686304756870	0.627976599850
C210	C	0.368200321036	0.186304756867	0.372023400150
C211	C	0.585293534335	0.576078140448	0.661358180888
C212	C	0.414706465665	0.076078140445	0.338641819111
C213	C	0.598436366261	0.743160006479	0.572998390268
C214	C	0.401563633739	0.243160006475	0.427001609732
C215	C	0.644274169245	0.811614100474	0.574481954171
C216	C	0.355725830755	0.311614100471	0.425518045829
C217	C	0.536373124325	0.765122618052	0.554794682741
C218	C	0.463626875675	0.265122618049	0.445205317259
C219	C	0.495706801039	0.696763987351	0.542476489417
C220	C	0.504293198961	0.196763987348	0.457523510582
C221	C	0.429266827645	0.724430264122	0.531890708147
C222	C	0.570733172355	0.224430264119	0.468109291852
C223	C	0.528275963126	0.652105427232	0.513157825617
C224	C	0.471724036874	0.152105427229	0.486842174383
C225	C	0.679345816281	0.924414563276	0.606361780808
C226	C	0.320654183720	0.424414563272	0.393638219192
C227	C	0.746096371170	0.902211568009	0.618958273157
C228	C	0.253903628830	0.402211568006	0.381041726843

C229	C	0.647179339648	0.985961770077	0.628534609346
C230	C	0.352820660353	0.485961770074	0.371465390654
C231	C	0.811739387328	0.797214833939	0.643423788557
C232	C	0.188260612672	0.297214833936	0.356576211443
C233	C	0.849179094535	0.777508999501	0.610070661546
C234	C	0.150820905465	0.277508999497	0.389929338454
C235	C	0.800810600273	0.727841354044	0.667214607406
C236	C	0.199189399727	0.227841354040	0.332785392594
C237	C	0.855802252468	0.714546725993	0.554076987376
C238	C	0.144197747532	0.214546725990	0.445923012624
C239	C	0.867555108416	0.787058145234	0.531802733857
C240	C	0.132444891585	0.287058145231	0.468197266143
C241	C	0.823770692374	0.650646246568	0.532941159799
C242	C	0.176229307626	0.150646246565	0.467058840201
C243	C	0.826754654280	0.572126909233	0.550430038422
C244	C	0.173245345720	0.072126909229	0.449569961578
C245	C	0.832195741174	0.916685504839	0.515569483661
C246	C	0.167804258826	0.416685504836	0.484430516339
C247	C	0.895103953206	0.958467114083	0.523185437539
C248	C	0.104896046794	0.458467114079	0.476814562461
C249	C	0.774688349393	0.969307776274	0.524347347492
C250	C	0.225311650607	0.469307776271	0.475652652508
C251	C	0.767360468365	0.041417294662	0.502819842037
C252	C	0.232639531635	0.541417294659	0.497180157963
C253	C	0.755636121030	0.037948363767	0.467129887952
C254	C	0.244363878970	0.537948363764	0.532870112047
C255	C	0.769327711244	0.114575350445	0.518168434799
C256	C	0.230672288756	0.614575350442	0.481831565201
C257	C	0.748083879195	0.104720798652	0.447497887562
C258	C	0.251916120805	0.604720798649	0.552502112438
C259	C	0.761938155534	0.182084039946	0.498991725031
C260	C	0.238061844466	0.682084039942	0.501008274969
C261	C	0.752468894662	0.177157408552	0.463252956126
C262	C	0.247531105339	0.677157408548	0.536747043874
C263	C	0.968819894284	0.997370481505	0.569674313491
C264	C	0.031180105716	0.497370481502	0.430325686509
C265	C	0.025931692598	0.956237066462	0.586074796405
C266	C	0.974068307402	0.456237066459	0.413925203595
C267	C	0.939858872201	0.049104050412	0.598455966964
C268	C	0.060141127799	0.549104050409	0.401544033036
C269	C	0.890986347772	0.106010362260	0.585042840348
C270	C	0.109013652228	0.606010362256	0.414957159651
C271	C	0.064399284641	0.839459704469	0.613716283962
C272	C	0.935600715359	0.339459704465	0.386283716038
C273	C	0.047398429663	0.844018366291	0.652487503578
C274	C	0.952601570337	0.344018366287	0.347512496422
C275	C	0.964818455285	0.874011409011	0.695241960071
C276	C	0.035181544715	0.374011409008	0.304758039928
C277	C	0.942884356491	0.788776446580	0.700475796128
C278	C	0.057115643509	0.288776446576	0.299524203872
C279	C	0.910097774353	0.931596143020	0.701571154309
C280	C	0.089902225647	0.431596143017	0.298428845691
C281	C	0.908556072169	0.696171592884	0.745406750364
C282	C	0.091443927831	0.196171592881	0.254593249636
C283	C	0.846786532347	0.695586664356	0.765864298495
C284	C	0.153213467653	0.195586664353	0.234135701505
C285	C	0.965586725742	0.669381315994	0.768960443484
C286	C	0.034413274258	0.169381315990	0.231039556516
C287	C	0.956231356917	0.689534590233	0.807710233166
C288	C	0.043768643083	0.189534590230	0.192289766834
C289	C	0.027381783385	0.703419898525	0.754517043893
C290	C	0.972618216615	0.203419898521	0.245482956107
C291	C	0.010920646808	0.666039876854	0.831502300767

C292	C	0.989079353192	0.166039876850	0.168497699233
C293	C	0.777095593447	0.618210060086	0.802659301661
C294	C	0.222904406553	0.118210060083	0.197340698339
C295	C	0.805728816555	0.554264125246	0.824822492051
C296	C	0.194271183445	0.054264125242	0.175177507949
C297	C	0.705789079168	0.597660765130	0.796375492205
C298	C	0.294210920832	0.097660765127	0.203624507795
C299	C	0.679023448102	0.630096332739	0.762476124796
C300	C	0.320976551898	0.130096332735	0.237523875204
C301	C	0.665552123079	0.621948634035	0.828010205856
C302	C	0.334447876921	0.121948634031	0.171989794144
C303	C	0.611941523307	0.600204665498	0.755489342837
C304	C	0.388058476694	0.100204665495	0.244510657163
C305	C	0.821907309745	0.510801876981	0.884410820818
C306	C	0.178092690255	0.010801876978	0.115589179182
C307	C	0.761184399708	0.502391581809	0.906088549445
C308	C	0.238815600292	0.002391581806	0.093911450555
C309	C	0.881758370968	0.525636425501	0.906975616780
C310	C	0.118241629032	0.025636425498	0.093024383220
C311	C	0.883439972291	0.609372810924	0.920222732119
C312	C	0.116560027709	0.109372810921	0.079777267881
C313	C	0.941247058361	0.502215249931	0.886280688475
C314	C	0.058752941639	0.002215249928	0.113719311525
C315	C	0.932548464659	0.624211076236	0.948411175317
C316	C	0.067451535341	0.124211076233	0.051588824683
C317	C	0.692124467337	0.423889618371	0.942384239669
C318	C	0.307875532663	0.923889618367	0.057615760331
C319	C	0.685256169595	0.476176644384	0.974563661949
C320	C	0.314743830405	0.976176644380	0.025436338051
C321	C	0.690651725094	0.336235911186	0.949472764649
C322	C	0.309348274906	0.836235911183	0.050527235351
C323	C	0.760029194653	0.309443291141	0.945639250588
C324	C	0.239970805347	0.809443291137	0.054360749412
C325	C	0.788058315858	0.362828706684	0.917936730816
C326	C	0.211941684142	0.862828706681	0.082063269184
C327	C	0.740737793829	0.559812100892	0.016172607306
C328	C	0.259262206171	0.059812100888	0.983827392694
C329	C	0.758072566442	0.641940281776	0.005035616329
C330	C	0.241927433558	0.141940281773	0.994964383671
C331	C	0.815242260196	0.760556551911	0.022170672230
C332	C	0.184757739804	0.260556551908	0.977829327770
C333	C	0.882335793825	0.775862723719	0.037144751231
C334	C	0.117664206175	0.275862723716	0.962855248769
C335	C	0.764987733867	0.811700571406	0.039335159279
C336	C	0.235012266133	0.311700571403	0.960664840721
C337	C	0.997026564024	0.744098110909	0.030595644990
C338	C	0.002973435976	0.244098110906	0.969404355010
C339	C	0.030982326716	0.770386527188	0.997122371348
C340	C	0.969017673284	0.270386527185	0.002877628652
C341	C	0.027968873954	0.669518699624	0.044717117574
C342	C	0.972031126046	0.169518699621	0.955282882426
C343	C	0.095779025926	0.684126751665	0.056935764624
C344	C	0.904220974074	0.184126751661	0.943064235376
C345	C	0.065048177221	0.877728421340	0.960407425148
C346	C	0.934951822779	0.377728421337	0.039592574852
C347	C	0.135643738686	0.886294987901	0.969894633729
C348	C	0.864356261314	0.386294987897	0.030105366271
C349	C	0.037971240295	0.953820456402	0.947227122531
C350	C	0.962028759705	0.453820456399	0.052772877469
C351	C	0.245986821349	0.841560215246	0.962063449837
C352	C	0.754013178651	0.341560215243	0.037936550163
C353	C	0.280956271170	0.896493956460	0.937208208751
C354	C	0.719043728830	0.396493956456	0.062791791249

C355	C	0.267845702724	0.756423019889	0.958266583070
C356	C	0.732154297276	0.256423019885	0.041733416930
C357	C	0.212213656559	0.714389929115	0.940306656950
C358	C	0.787786343441	0.214389929111	0.059693343050
C359	C	0.167323871935	0.778312041171	0.928316910188
C360	C	0.832676128065	0.278312041168	0.071683089812
C361	C	0.379009724018	0.964994646293	0.922805775503
C362	C	0.620990275982	0.464994646290	0.077194224497
C363	C	0.384971078241	0.949516387281	0.883707148754
C364	C	0.615028921759	0.449516387278	0.116292851245
C365	C	0.371310618042	0.857166027014	0.836457886262
C366	C	0.628689381958	0.357166027011	0.163542113738
C367	C	0.309935281844	0.892676880125	0.821387517925
C368	C	0.690064718156	0.392676880122	0.178612482074
C369	C	0.375403496207	0.769367942882	0.831287868153
C370	C	0.624596503793	0.269367942879	0.168712131847
C371	C	0.330502496709	0.717531625489	0.853613575112
C372	C	0.669497503291	0.217531625486	0.146386424888
C373	C	0.193199645854	0.896915329604	0.826425342861
C374	C	0.806800354146	0.396915329601	0.173574657139
C375	C	0.170185240053	0.954986707519	0.853851037994
C376	C	0.829814759947	0.454986707516	0.146148962006
C377	C	0.147230870853	0.830187207568	0.819394580286
C378	C	0.852769129147	0.330187207565	0.180605419714
C379	C	0.172736163075	0.780817871028	0.790160640985
C380	C	0.827263836925	0.280817871025	0.209839359015
C381	C	0.215517659371	0.720683318204	0.796567187532
C382	C	0.784482340629	0.220683318201	0.203432812468
C383	C	0.158390101016	0.797750137795	0.755623190258
C384	C	0.841609898985	0.297750137791	0.244376809742
C385	C	0.243133001270	0.678836370533	0.769534035249
C386	C	0.756866998730	0.178836370529	0.230465964751
C387	C	0.183773326415	0.755018235973	0.728203035633
C388	C	0.816226673586	0.255018235970	0.271796964367
C389	C	0.226335503645	0.695308937795	0.735340432748
C390	C	0.773664496355	0.195308937791	0.264659567252
C391	C	0.206543698096	0.072310791897	0.883160163523
C392	C	0.793456301904	0.572310791893	0.116839836477
C393	C	0.146054123026	0.119470213001	0.887708593298
C394	C	0.853945876974	0.619470212997	0.112291406702
C395	C	0.263434883135	0.127323042392	0.879653735219
C396	C	0.736565116865	0.627323042389	0.120346264781
C397	C	0.068705520345	0.203886789487	0.859569219591
C398	C	0.931294479655	0.703886789483	0.140430780409
C399	C	0.998505202330	0.185157064995	0.851928186983
C400	C	0.001494797670	0.685157064992	0.148071813017
C401	C	0.094458521620	0.263841649602	0.833566845443
C402	C	0.905541478380	0.763841649599	0.166433154557
C403	C	0.163735219819	0.284536736467	0.839814362724
C404	C	0.836264780181	0.784536736463	0.160185637276
N1	N	0.660190300720	0.690765159752	0.913461270651
N2	N	0.339809699280	0.190765159748	0.086538729349
N3	N	0.714462147171	0.838142806474	0.854370408162
N4	N	0.285537852829	0.338142806471	0.145629591838
N5	N	0.876270312148	0.882617678980	0.833569609201
N6	N	0.123729687852	0.382617678976	0.166430390799
N7	N	0.008003342664	0.995258081245	0.803840669542
N8	N	0.991996657336	0.495258081242	0.196159330458
N9	N	0.135028380154	0.009625241029	0.740990425784
N10	N	0.864971619846	0.509625241026	0.259009574216
N11	N	0.194982277522	0.152487431653	0.733999655429
N12	N	0.805017722478	0.652487431649	0.266000344571
N13	N	0.176711421962	0.357199969806	0.749750051434

N14	N	0.823288578038	0.857199969803	0.250249948566
N15	N	0.125874002526	0.442726419478	0.693613686624
N16	N	0.874125997474	0.942726419475	0.306386313376
N17	N	0.082157601179	0.313233541608	0.655786573741
N18	N	0.917842398821	0.813233541604	0.344213426259
N19	N	0.981046157966	0.277533096341	0.701315727318
N20	N	0.018953842034	0.777533096337	0.298684272682
N21	N	0.856766173099	0.200034440774	0.812274568898
N22	N	0.143233826901	0.700034440770	0.187725431102
N23	N	0.770341663816	0.115491907173	0.810738335542
N24	N	0.229658336184	0.615491907169	0.189261664458
N25	N	0.849412019420	0.102119616847	0.852428532500
N26	N	0.150587980580	0.602119616843	0.147571467500
N27	N	0.908240503795	0.419745720258	0.691959582872
N28	N	0.091759496205	0.919745720254	0.308040417128
N29	N	0.871370628791	0.395047843053	0.621396697377
N30	N	0.128629371209	0.895047843050	0.378603302623
N31	N	0.000038299285	0.380511616303	0.536077766951
N32	N	0.999961700715	0.880511616299	0.463922233049
N33	N	0.810912703344	0.255383566008	0.623674430262
N34	N	0.189087296656	0.755383566004	0.376325569738
N35	N	0.709825867179	0.304972889016	0.666586916237
N36	N	0.290174132821	0.804972889012	0.333413083763
N37	N	0.743751565377	0.447172274860	0.749807133183
N38	N	0.256248434623	0.947172274857	0.250192866817
N39	N	0.641318139884	0.400893814051	0.621633379729
N40	N	0.358681860116	0.900893814047	0.378366620271
N41	N	0.603581228897	0.282344042679	0.575904713648
N42	N	0.396418771103	0.782344042676	0.424095286352
N43	N	0.522548479414	0.201641235244	0.625247802055
N44	N	0.477451520586	0.701641235240	0.374752197945
N45	N	0.503539554433	0.017095308703	0.734598410960
N46	N	0.496460445567	0.517095308699	0.265401589040
N47	N	0.394373503707	0.019250649561	0.722585014892
N48	N	0.605626496294	0.519250649558	0.277414985108
N49	N	0.429093785050	0.012243569442	0.779058058675
N50	N	0.570906214950	0.512243569438	0.220941941325
N51	N	0.427785484233	0.311119081311	0.635232766129
N52	N	0.572214515767	0.811119081308	0.364767233871
N53	N	0.311978722420	0.322526742241	0.588124170642
N54	N	0.688021277580	0.822526742238	0.411875829358
N55	N	0.251186382843	0.185520792262	0.604313927543
N56	N	0.748813617157	0.685520792259	0.395686072457
N57	N	0.321501001932	0.052202861181	0.597515687336
N58	N	0.678498998068	0.552202861177	0.402484312664
N59	N	0.369536817660	0.877787410764	0.634227141478
N60	N	0.630463182340	0.377787410760	0.365772858522
N61	N	0.480315467387	0.835505554971	0.704525640285
N62	N	0.519684532613	0.335505554968	0.295474359715
N63	N	0.569010232049	0.709627831784	0.682108967352
N64	N	0.430989767951	0.209627831780	0.317891032648
N65	N	0.584428249470	0.713056317790	0.607663957323
N66	N	0.415571750530	0.213056317786	0.392336042677
N67	N	0.638011596555	0.858506661221	0.602299803916
N68	N	0.361988403446	0.358506661218	0.397700196084
N69	N	0.751384297432	0.834161658061	0.635129931170
N70	N	0.248615702568	0.334161658057	0.364870068830
N71	N	0.821827399812	0.730621619792	0.586471087292
N72	N	0.178172600188	0.230621619789	0.413528912708
N73	N	0.824923617151	0.845454823903	0.535163594748
N74	N	0.175076382849	0.345454823900	0.464836405251
N75	N	0.919314830094	0.948453523388	0.555434174036
N76	N	0.080685169906	0.448453523384	0.444565825964

N77	N	0.021957398157	0.878695897570	0.590317909166
N78	N	0.978042601843	0.378695897566	0.409682090834
N79	N	0.993759906992	0.882956298030	0.661390123101
N80	N	0.006240093008	0.382956298026	0.338609876899
N81	N	0.917624312280	0.773698964083	0.731992823559
N82	N	0.082375687720	0.273698964080	0.268007176441
N83	N	0.818185154436	0.626366364309	0.771998374920
N84	N	0.181814845564	0.126366364305	0.228001625080
N85	N	0.811350145026	0.570633917091	0.858795875745
N86	N	0.188649854974	0.070633917088	0.141204124255
N87	N	0.751751689268	0.434935210892	0.922844892830
N88	N	0.248248310732	0.934935210889	0.077155107170
N89	N	0.739307946868	0.507104982694	0.987252594439
N90	N	0.260692053132	0.007104982690	0.012747405561
N91	N	0.799907405299	0.678433053309	0.025332566023
N92	N	0.200092594701	0.178433053306	0.974667433977
N93	N	0.929771085390	0.733858282322	0.022498507294
N94	N	0.070228914610	0.233858282318	0.977501492706
N95	N	0.031750308376	0.847328095368	0.990818696043
N96	N	0.968249691624	0.347328095365	0.009181303957
N97	N	0.178138272165	0.839948893113	0.954023358879
N98	N	0.821861727835	0.339948893109	0.045976641121
N99	N	0.344303550166	0.907513294306	0.942894994568
N100	N	0.655696449834	0.407513294303	0.057105005432
N101	N	0.377932731870	0.875827714499	0.873167711167
N102	N	0.622067268130	0.375827714496	0.126832288833
N103	N	0.255694421101	0.868976576733	0.836364213300
N104	N	0.744305578899	0.368976576730	0.163635786700
N105	N	0.206083949295	0.019745043740	0.854176199788
N106	N	0.793916050705	0.519745043736	0.145823800212
N107	N	0.111848822125	0.138044908418	0.860046091633
N108	N	0.888151177876	0.638044908415	0.139953908367
N109	N	0.177901721677	0.314460114502	0.871277055422
N110	N	0.822098278323	0.814460114498	0.128722944578
O1	O	0.765733995698	0.732621233346	0.876379380981
O2	O	0.234266004302	0.232621233343	0.123620619019
O3	O	0.655382974319	0.789833904918	0.965990636088
O4	O	0.344617025681	0.289833904914	0.034009363912
O5	O	0.811215073289	0.948610647706	0.871192558776
O6	O	0.188784926711	0.448610647703	0.128807441224
O7	O	0.699947219629	0.967848771697	0.813347985517
O8	O	0.300052780371	0.467848771693	0.186652014483
O9	O	0.962206960769	0.886879120325	0.781760047896
O10	O	0.037793039231	0.386879120322	0.218239952104
O11	O	0.132384688733	0.060020200372	0.795114481883
O12	O	0.867615311267	0.560020200368	0.204885518117
O13	O	0.291121499845	0.100913198686	0.744962293451
O14	O	0.708878500155	0.600913198682	0.255037706549
O15	O	0.105962123926	0.261749468265	0.737928814462
O16	O	0.894037876074	0.761749468262	0.262071185538
O17	O	0.209137102083	0.264476647548	0.676296214110
O18	O	0.790862897918	0.764476647544	0.323703785890
O19	O	0.033249070938	0.442014888039	0.724357772084
O20	O	0.966750929062	0.942014888035	0.275642227916
O21	O	0.005837945982	0.390909748430	0.634157818565
O22	O	0.994162054018	0.890909748427	0.365842181435
O23	O	0.929667572570	0.234001643174	0.653501832816
O24	O	0.070332427430	0.734001643170	0.346498167184
O25	O	0.823384203393	0.341360396775	0.699971379141
O26	O	0.176615796607	0.841360396772	0.300028620859
O27	O	0.778343989015	0.454325762995	0.636520919614
O28	O	0.221656010985	0.954325762992	0.363479080386
O29	O	0.943699001255	0.579962674007	0.685834788725

O30	O	0.056300998745	0.079962674004	0.314165211275
O31	O	0.748758854015	0.294543199444	0.578756140936
O32	O	0.251241145985	0.794543199440	0.421243859064
O33	O	0.942057404359	0.465657545565	0.567265955582
O34	O	0.057942595641	0.965657545562	0.432734044418
O35	O	0.652995064882	0.206236878636	0.643412758967
O36	O	0.347004935118	0.706236878632	0.356587241033
O37	O	0.553064514635	0.373308688660	0.653891699493
O38	O	0.446935485365	0.873308688656	0.346108300507
O39	O	0.694695420746	0.330165367446	0.751267167316
O40	O	0.305304579254	0.830165367443	0.248732832684
O41	O	0.514097390132	0.352962107063	0.567611602720
O42	O	0.485902609868	0.852962107059	0.432388397280
O43	O	0.464582223134	0.170918374002	0.578164564732
O44	O	0.535417776866	0.670918373998	0.421835435267
O45	O	0.358569856259	0.214809346434	0.648826118294
O46	O	0.641430143741	0.714809346430	0.351173881706
O47	O	0.267399546157	0.367868670837	0.637578603711
O48	O	0.732600453843	0.867868670834	0.362421396289
O49	O	0.159216883497	0.224895374390	0.578107368213
O50	O	0.840783116503	0.724895374387	0.421892631787
O51	O	0.245496732322	0.979813504438	0.622884804666
O52	O	0.754503267678	0.479813504434	0.377115195334
O53	O	0.408918234805	0.994873674012	0.647846321463
O54	O	0.591081765195	0.494873674009	0.352153678537
O55	O	0.402425775337	0.058467844831	0.543310802390
O56	O	0.597574224663	0.558467844827	0.456689197610
O57	O	0.497980337663	0.836351764136	0.645874039877
O58	O	0.502019662337	0.336351764132	0.354125960123
O59	O	0.640505651003	0.807071759280	0.683332855400
O60	O	0.359494348997	0.307071759276	0.316667144600
O61	O	0.586425279726	0.850599441219	0.829734270832
O62	O	0.413574720274	0.350599441216	0.170265729168
O63	O	0.576233957575	0.947313992034	0.791179280027
O64	O	0.423766042425	0.447313992030	0.208820719973
O65	O	0.687374406198	0.681250568114	0.617821108129
O66	O	0.312625593802	0.181250568111	0.382178891871
O67	O	0.684165111260	0.822558821943	0.551407576437
O68	O	0.315834888740	0.322558821940	0.448592423563
O69	O	0.791370169859	0.946170700615	0.612720209897
O70	O	0.208629830141	0.446170700612	0.387279790103
O71	O	0.902615244018	0.805508200662	0.605410915244
O72	O	0.097384755982	0.305508200658	0.394589084756
O73	O	0.914792680107	0.790645500095	0.512910850460
O74	O	0.085207319893	0.290645500092	0.487089149540
O75	O	0.759755559333	0.675963187990	0.527047410241
O76	O	0.240244440667	0.175963187987	0.472952589759
O77	O	0.919149369362	0.000378412960	0.500885437671
O78	O	0.080850630638	0.500378412956	0.499114562329
O79	O	0.747269678461	0.244415994050	0.444764922598
O80	O	0.252730321539	0.744415994046	0.555235077402
O81	O	0.070687504079	0.994746857116	0.597000788163
O82	O	0.929312495921	0.494746857113	0.402999211837
O83	O	0.914080863845	0.000541833145	0.625116033408
O84	O	0.085919136155	0.500541833142	0.374883966592
O85	O	0.082706511425	0.813099043413	0.673830818025
O86	O	0.917293488575	0.313099043409	0.326169181975
O87	O	0.950122740299	0.740248721392	0.677325229445
O88	O	0.049877259701	0.240248721388	0.322674770555
O89	O	0.825748474351	0.756135817491	0.778090401245
O90	O	0.174251525649	0.256135817487	0.221909598755
O91	O	0.820708324245	0.491584686521	0.811671519115
O92	O	0.179291675755	0.991584686518	0.188328480885

```
O93   O     0.721702142265   0.555316637404   0.908606922335
O94   O     0.278297857735   0.055316637400   0.091393077665
O95   O     0.631782948310   0.487062139301   0.986914674657
O96   O     0.368217051690   0.987062139297   0.013085325343
O97   O     0.732603974411   0.671858846233   0.979217535550
O98   O     0.267396025590   0.171858846230   0.020782464450
O99   O     0.891552331192   0.824629511176   0.059804772798
O100   O     0.108447668808   0.324629511173   0.940195227202
O101   O     0.054076385882   0.723131500967   0.976786471569
O102   O     0.945923614118   0.223131500963   0.023213528431
O103   O     0.994238915289   0.639280157089   0.073261930048
O104   O     0.005761084711   0.139280157085   0.926738069952
O105   O     0.149797191932   0.933059658274   0.993177871045
O106   O     0.850202808068   0.433059658271   0.006822128955
O107   O     0.253945902861   0.927627989068   0.912888669678
O108   O     0.746054097139   0.427627989065   0.087111330322
O109   O     0.397619990619   0.003491086423   0.863994693040
O110   O     0.602380009381   0.503491086420   0.136005306960
O111   O     0.311978728206   0.940527491007   0.797249244171
O112   O     0.688021271794   0.440527491003   0.202750755829
O113   O     0.299480194021   0.751483372055   0.878146813854
O114   O     0.700519805979   0.251483372051   0.121853186146
O115   O     0.328799992879   0.648428877068   0.845817131644
O116   O     0.671200007121   0.148428877065   0.154182868356
O117   O     0.125347604979   0.943337528477   0.873937958131
O118   O     0.874652395021   0.443337528474   0.126062041869
O119   O     0.252814239245   0.650748012151   0.709408436363
O120   O     0.747185760755   0.150748012148   0.290591563637
O121   O     0.134000229011   0.144243668605   0.917675941054
O122   O     0.865999770989   0.644243668601   0.082324058946
O123   O     0.982442586012   0.115794032366   0.847069624301
O124   O     0.017557413988   0.615794032363   0.152930375699
O125   O     0.205037679680   0.274875805766   0.817264890330
O126   O     0.794962320321   0.774875805762   0.182735109670
O127   O     0.962003215603   0.242655606329   0.850696359830
O128   O     0.037996784397   0.742655606326   0.149303640170
S1   S     0.946910271287   0.846260560325   0.906714145022
S2   S     0.053089728713   0.346260560321   0.093285854978
S3   S     0.916790539340   0.083136663073   0.746460406696
S4   S     0.083209460660   0.583136663070   0.253539593304
S5   S     0.596106809095   0.049344066820   0.559963163275
S6   S     0.403893190905   0.549344066816   0.440036836725
S7   S     0.570730238440   0.016176948940   0.610199487293
S8   S     0.429269761560   0.516176948936   0.389800512707
S9   S     0.928531637109   0.035703181074   0.696911592177
S10   S     0.071468362891   0.535703181071   0.303088407823
S11   S     0.953286066695   0.948920430483   0.934499320238
S12   S     0.046713933305   0.448920430479   0.065500679762
#END
```

2) CRAMBIN CRYSTAL – optimized 84W

```
data_CSD_CIF_MOLDRAW
_audit_creation_date 09-04-2015
_audit_creation_method MOLDRAW
_chemical_formula_sum 'H798 C404 N110 O212 S12'
_chemical_formula_moiety
```

```
;
H798 C404 N110 O212 S12
;
loop_
_publ_author_name
Delle Piane, Corno, Orlando, Dovesi, Ugliengo
_cell_volume 15471.6953
_exptl_crystal_density_diffrn ?
_diffrn_ambient_temperature ?
_refine_ls_R_factor_gt ?
_refine_ls_wR_factor_gt ?
_symmetry_space_group_name_H-M 'P 1'
_symmetry_cell_setting monoclinic
_symmetry_Int_Tables_number 1
loop_
_symmetry_equiv_pos_site_id
_symmetry_equiv_pos_as_xyz
1 x,y,z
_cell_length_a  21.7410932
_cell_length_b  17.8523195
_cell_length_c  39.8623203
_cell_angle_alpha  90
_cell_angle_beta  89.897202
_cell_angle_gamma  90
_cell_formula_units_Z 1
loop_
_atom_type_symbol
_atom_type_radius_bond
H  .38
C  .77
N  .75
O  .73
S  1.04
loop_
_atom_site_label
_atom_site_type_symbol
_atom_site_fract_x
_atom_site_fract_y
_atom_site_fract_z
H1   H     0.687337736790   0.692560758752   0.910104710761
H2   H     0.312662263210   0.192560758752   0.089895289239
H3   H     0.621590265058   0.733773375377   0.894589781017
H4   H     0.378409734942   0.233773375377   0.105410218983
H5   H     0.637452395337   0.729160278041   0.937301069221
H6   H     0.362547604664   0.229160278041   0.062698930779
H7   H     0.649618786138   0.853176197160   0.913659252554
H8   H     0.350381213862   0.353176197160   0.086340747446
H9   H     0.766179670228   0.778429817851   0.941066166204
H10   H     0.233820329772   0.278429817851   0.058933833796
H11   H     0.714364452187   0.753218386005   0.982406902501
H12   H     0.285635547813   0.253218386005   0.017593097499
H13   H     0.778894828306   0.899897664884   0.972328848105
H14   H     0.221105171694   0.399897664884   0.027671151895
H15   H     0.715717950024   0.938796112655   0.949880478575
H16   H     0.284282049976   0.438796112655   0.050119521424
H17   H     0.784241506774   0.912645729534   0.928221702936
H18   H     0.215758493226   0.412645729534   0.071778297064
H19   H     0.709650292388   0.929642874694   0.878324467184
H20   H     0.290349707612   0.429642874694   0.121675532816
H21   H     0.785479536145   0.840295398837   0.830484455850
H22   H     0.214520463855   0.340295398837   0.169515544150
H23   H     0.772620896133   0.956044787253   0.792069384656
H24   H     0.227379103867   0.456044787253   0.207930615344
```

H25	H	0.655114577091	0.982106008500	0.815265134191
H26	H	0.344885422910	0.482106008500	0.184734865809
H27	H	0.734587571563	0.829839069963	0.780376401008
H28	H	0.265412428437	0.329839069963	0.219623598992
H29	H	0.678432692979	0.896070178292	0.767796819783
H30	H	0.321567307021	0.396070178292	0.232203180217
H31	H	0.667748469231	0.844194812788	0.805554052121
H32	H	0.332251530769	0.344194812788	0.194445947879
H33	H	0.873298226623	0.871253118343	0.816846216817
H34	H	0.126701773377	0.371253118343	0.183153783182
H35	H	0.937732374648	0.993808594592	0.853972954947
H36	H	0.062267625352	0.493808594592	0.146027045053
H37	H	0.988796008946	0.837951722602	0.836411016595
H38	H	0.011203991054	0.337951722602	0.163588983405
H39	H	0.026956676040	0.909584177783	0.859447647998
H40	H	0.973043323960	0.409584177783	0.140552352002
H41	H	0.008116989142	0.059667254010	0.818650911465
H42	H	0.991883010858	0.559667254010	0.181349088535
H43	H	0.012946671377	0.995155961355	0.750094813384
H44	H	0.987053328623	0.495155961355	0.249905186616
H45	H	0.998751795980	0.158643565745	0.771959251007
H46	H	0.001248204020	0.658643565745	0.228040748993
H47	H	0.019685318886	0.134151461988	0.730648282770
H48	H	0.980314681114	0.634151461988	0.269351717230
H49	H	0.213346450980	0.006588507647	0.762783271113
H50	H	0.786653549020	0.506588507647	0.237216728887
H51	H	0.253087189589	0.979854043828	0.698840592080
H52	H	0.746912810411	0.479854043828	0.301159407920
H53	H	0.208262610404	0.912763361805	0.720849697223
H54	H	0.791737389596	0.412763361805	0.279150302776
H55	H	0.159253115924	0.028864261163	0.670786009304
H56	H	0.840746884076	0.528864261163	0.329213990696
H57	H	0.148495706008	0.931364708485	0.669277926841
H58	H	0.851504293992	0.431364708485	0.330722073159
H59	H	0.062235059441	0.022109410510	0.699069814209
H60	H	0.937764940559	0.522109410510	0.300930185791
H61	H	0.079935303522	0.933017644588	0.716842594394
H62	H	0.920064696478	0.433017644588	0.283157405606
H63	H	0.136581156947	0.144539577330	0.719787798017
H64	H	0.863418843053	0.644539577330	0.280212201983
H65	H	0.235170151480	0.244447454359	0.736468506007
H66	H	0.764829848520	0.744447454359	0.263531493993
H67	H	0.252955331636	0.302851429782	0.684173831637
H68	H	0.747044668364	0.802851429782	0.315826168363
H69	H	0.265414406165	0.206409413371	0.678463207790
H70	H	0.734585593835	0.706409413371	0.321536792210
H71	H	0.202913923580	0.276309795029	0.638770882473
H72	H	0.797086076420	0.776309795029	0.361229117527
H73	H	0.205648704469	0.367769034000	0.730498138572
H74	H	0.794351295531	0.867769034000	0.269501861428
H75	H	0.080836287200	0.395110961202	0.752785140627
H76	H	0.919163712800	0.895110961202	0.247214859373
H77	H	0.182153602847	0.496748919605	0.724871661763
H78	H	0.817846397154	0.996748919605	0.275128338236
H79	H	0.143217967214	0.464968220302	0.797536411149
H80	H	0.856782032786	0.964968220302	0.202463588851
H81	H	0.208529095928	0.427463337125	0.777488422817
H82	H	0.791470904072	0.927463337125	0.222511577183
H83	H	0.082370477145	0.559166401424	0.717673184638
H84	H	0.917629522855	0.059166401423	0.282326815362
H85	H	0.123916313334	0.602307364480	0.750246421690
H86	H	0.876083686666	0.102307364480	0.249753578309
H87	H	0.061684650521	0.540838144560	0.760007805675

H88	H	0.938315349479	0.040838144560	0.239992194324
H89	H	0.246265575164	0.561332304071	0.766506439582
H90	H	0.753734424836	0.061332304071	0.233493560418
H91	H	0.245398599611	0.535050502800	0.809311933468
H92	H	0.754601400389	0.035050502799	0.190688066532
H93	H	0.187302696923	0.593950923058	0.793265772162
H94	H	0.812697303077	0.093950923058	0.206734227838
H95	H	0.150595252716	0.425275991896	0.670957203225
H96	H	0.849404747284	0.925275991896	0.329042796775
H97	H	0.028415279311	0.469289033187	0.647282870063
H98	H	0.971584720689	0.969289033187	0.352717129937
H99	H	0.151570919352	0.446593121374	0.614023978610
H100	H	0.848429080648	0.946593121374	0.385976021389
H101	H	0.081567564159	0.395016898716	0.571388486581
H102	H	0.918432435841	0.895016898716	0.428611513419
H103	H	0.107372964135	0.484934215821	0.559140666193
H104	H	0.892627035865	0.984934215821	0.440859333807
H105	H	0.031477921726	0.472707893878	0.574900173731
H106	H	0.968522078274	0.972707893878	0.425099826269
H107	H	0.132591210825	0.584546385841	0.601341597398
H108	H	0.867408789175	0.084546385841	0.398658402602
H109	H	0.130305417974	0.565329810557	0.645016976663
H110	H	0.869694582026	0.065329810557	0.354983023337
H111	H	0.060864539396	0.578302694051	0.622289710016
H112	H	0.939135460604	0.078302694051	0.377710289984
H113	H	0.100283154777	0.289911638011	0.653117826384
H114	H	0.899716845223	0.789911638011	0.346882173616
H115	H	0.004644929986	0.220486717073	0.612538895519
H116	H	0.995355070014	0.720486717073	0.387461104481
H117	H	0.094917545907	0.146555585983	0.629427247891
H118	H	0.905082454093	0.646555585983	0.370572752109
H119	H	0.026511660688	0.105816365007	0.645458354871
H120	H	0.973488339312	0.605816365007	0.354541645129
H121	H	0.076361103858	0.157763370020	0.672585869410
H122	H	0.923638896142	0.657763370020	0.327414130589
H123	H	0.009744450705	0.279182301677	0.699053684009
H124	H	0.990255549295	0.779182301677	0.300946315991
H125	H	0.883955571647	0.237739045413	0.712911173133
H126	H	0.116044428353	0.737739045413	0.287088826867
H127	H	0.959979298752	0.272448845859	0.757098459583
H128	H	0.040020701248	0.772448845859	0.242901540417
H129	H	0.963673255653	0.364173692591	0.741193453520
H130	H	0.036326744347	0.864173692591	0.258806546480
H131	H	0.898271484113	0.369498874612	0.789319420786
H132	H	0.101728515887	0.869498874612	0.210680579214
H133	H	0.852199802743	0.384246497381	0.754107426280
H134	H	0.147800197257	0.884246497381	0.245892573720
H135	H	0.794075461485	0.305825778918	0.789900977779
H136	H	0.205924538515	0.805825778918	0.210099022221
H137	H	0.818182124519	0.246339229883	0.757210111578
H138	H	0.181817875481	0.746339229883	0.242789888422
H139	H	0.909347834386	0.240573913044	0.809210315045
H140	H	0.090652165614	0.740573913044	0.190789684955
H141	H	0.748012816383	0.194952858051	0.807774968551
H142	H	0.251987183617	0.694952858051	0.192225031449
H143	H	0.762343814424	0.101469349324	0.817724390639
H144	H	0.237656185576	0.601469349324	0.182275609361
H145	H	0.920091489136	0.134713673544	0.836800367591
H146	H	0.079908510864	0.634713673544	0.163199632408
H147	H	0.856023523051	0.085433232735	0.849389178213
H148	H	0.143976476949	0.585433232735	0.150610821787
H149	H	0.946018141272	0.410360313763	0.679562399515
H150	H	0.053981858728	0.910360313763	0.320437600485

H151	H	0.830013032238	0.480227929668	0.675226662508
H152	H	0.169986967763	0.980227929668	0.324773337491
H153	H	0.933111681167	0.494921120791	0.623077557714
H154	H	0.066888318833	0.994921120791	0.376922442286
H155	H	0.869864617624	0.557054036406	0.629569070797
H156	H	0.130135382376	0.057054036406	0.370430929203
H157	H	0.955107588646	0.600347062272	0.656900591444
H158	H	0.044892411354	0.100347062272	0.343099408556
H159	H	0.903585666711	0.365844960840	0.613599549869
H160	H	0.096414333289	0.865844960840	0.386400450131
H161	H	0.804712911967	0.373160906070	0.565959867243
H162	H	0.195287088033	0.873160906070	0.434040132757
H163	H	0.903598832656	0.251904197435	0.577024929861
H164	H	0.096401167344	0.751904197435	0.422975070139
H165	H	0.854605140311	0.258718959095	0.541790057435
H166	H	0.145394859689	0.758718959095	0.458209942565
H167	H	0.982014855395	0.260668490211	0.533942010256
H168	H	0.017985144605	0.760668490211	0.466057989744
H169	H	0.004637554999	0.350016448012	0.519858888589
H170	H	0.995362445001	0.850016448012	0.480141111410
H171	H	0.835256173138	0.246900746486	0.633618447592
H172	H	0.164743826862	0.746900746486	0.366381552408
H173	H	0.732629636465	0.149799077295	0.623567735844
H174	H	0.267370363535	0.649799077295	0.376432264156
H175	H	0.819284158184	0.121540937621	0.660694845483
H176	H	0.180715841816	0.621540937621	0.339305154517
H177	H	0.805161399604	0.202237761696	0.684864801723
H178	H	0.194838600396	0.702237761696	0.315135198277
H179	H	0.719643883371	0.208061699208	0.725608879566
H180	H	0.280356116630	0.708061699208	0.274391120434
H181	H	0.755824765758	0.008286164201	0.668371760080
H182	H	0.244175234242	0.508286164201	0.331628239920
H183	H	0.653128844756	0.135738201328	0.764149169617
H184	H	0.346871155244	0.635738201328	0.235850830383
H185	H	0.687631884571	0.935396592841	0.706516536742
H186	H	0.312368115430	0.435396592842	0.293483463258
H187	H	0.636234111315	0.000483381306	0.754878935871
H188	H	0.363765888685	0.500483381306	0.245121064129
H189	H	0.741729722189	0.319066234576	0.673797221584
H190	H	0.258270277811	0.819066234576	0.326202778416
H191	H	0.615154898878	0.301986994991	0.694401026655
H192	H	0.384845101123	0.801986994991	0.305598973345
H193	H	0.692580676532	0.446236828187	0.690391110687
H194	H	0.307419323468	0.946236828187	0.309608889313
H195	H	0.618713638533	0.436923642536	0.708555813985
H196	H	0.381286361467	0.936923642536	0.291444186015
H197	H	0.748068585506	0.472741332650	0.739372014685
H198	H	0.251931414494	0.972741332650	0.260627985315
H199	H	0.740907763013	0.416221662719	0.775291795026
H200	H	0.259092236987	0.916221662719	0.224708204974
H201	H	0.679084163474	0.383953472982	0.620688379838
H202	H	0.320915836526	0.883953472982	0.379311620162
H203	H	0.559333118600	0.440830292852	0.598510440848
H204	H	0.440666881400	0.940830292852	0.401489559152
H205	H	0.681713400684	0.408802013343	0.564238941640
H206	H	0.318286599316	0.908802013343	0.435761058360
H207	H	0.638501367326	0.465377004816	0.511967757219
H208	H	0.361498632675	0.965377004816	0.488032242781
H209	H	0.563256161028	0.462277788497	0.529431373464
H210	H	0.436743838972	0.962277788497	0.470568626535
H211	H	0.604056172470	0.377994992337	0.521446922094
H212	H	0.395943827530	0.877994992337	0.478553077906
H213	H	0.679192438706	0.550744640754	0.557852342032

H214	H	0.320807561294	0.050744640754	0.442147657968
H215	H	0.674270357846	0.522603788492	0.600063478508
H216	H	0.325729642154	0.022603788492	0.399936521492
H217	H	0.606788146482	0.551369726858	0.578637153938
H218	H	0.393211853518	0.051369726858	0.421362846062
H219	H	0.643178021107	0.270477267169	0.581231000084
H220	H	0.356821978893	0.770477267169	0.418768999916
H221	H	0.550282772744	0.202183087595	0.540665516594
H222	H	0.449717227256	0.702183087595	0.459334483406
H223	H	0.655259497245	0.152478889524	0.548563880485
H224	H	0.344740502755	0.652478889524	0.451436119515
H225	H	0.642402833130	0.140799591736	0.592263648479
H226	H	0.357597166870	0.640799591736	0.407736351520
H227	H	0.559294631779	0.215411161657	0.630002624275
H228	H	0.440705368221	0.715411161657	0.369997375725
H229	H	0.447690512102	0.129489593687	0.639744618548
H230	H	0.552309487898	0.629489593687	0.360255381452
H231	H	0.521049740886	0.238494670085	0.683580301952
H232	H	0.478950259114	0.738494670085	0.316419698048
H233	H	0.456323780954	0.190492896246	0.698713645596
H234	H	0.543676219046	0.690492896246	0.301286354404
H235	H	0.569579584952	0.109820394181	0.669469373305
H236	H	0.430420415048	0.609820394181	0.330530626695
H237	H	0.561640997955	0.132720384093	0.711935180717
H238	H	0.438359002045	0.632720384093	0.288064819283
H239	H	0.469995615697	0.035148086859	0.672753108000
H240	H	0.530004384303	0.535148086859	0.327246892000
H241	H	0.531774774758	0.999552081210	0.696267539592
H242	H	0.468225225242	0.499552081210	0.303732460408
H243	H	0.482073803181	0.077825454706	0.744955669440
H244	H	0.517926196819	0.577825454706	0.255044330560
H245	H	0.401755389315	0.962679752294	0.684682953960
H246	H	0.598244610685	0.462679752294	0.315317046040
H247	H	0.356096539019	0.923245814971	0.716352226422
H248	H	0.643903460981	0.423245814971	0.283647773578
H249	H	0.418469876059	0.024606082941	0.783701539497
H250	H	0.581530123941	0.524606082941	0.216298460503
H251	H	0.356943409406	0.969650504037	0.769936801688
H252	H	0.643056590594	0.469650504037	0.230063198312
H253	H	0.460619925910	0.315080430589	0.612428824817
H254	H	0.539380074091	0.815080430589	0.387571175183
H255	H	0.356470160870	0.369946099827	0.649637688410
H256	H	0.643529839131	0.869946099827	0.350362311590
H257	H	0.411602861996	0.415046345963	0.581884012902
H258	H	0.588397138004	0.915046345963	0.418115987097
H259	H	0.347808320941	0.460884589700	0.600021686882
H260	H	0.652191679059	0.960884589700	0.399978313118
H261	H	0.476128826413	0.449318385142	0.630965309696
H262	H	0.523871173587	0.949318385142	0.369034690304
H263	H	0.470677535033	0.526018228276	0.579292627073
H264	H	0.529322464967	0.026018228276	0.420707372927
H265	H	0.481727604534	0.585702857553	0.614307789476
H266	H	0.518272395466	0.085702857553	0.385692210523
H267	H	0.408026423770	0.578174968158	0.595668208594
H268	H	0.591973576230	0.078174968158	0.404331791406
H269	H	0.437020953394	0.546223664017	0.672063838098
H270	H	0.562979046606	0.046223664017	0.327936161902
H271	H	0.400445404887	0.458799970200	0.676878681411
H272	H	0.599554595113	0.958799970200	0.323121318589
H273	H	0.361624624322	0.533425490484	0.656104438484
H274	H	0.638375375678	0.033425490484	0.343895561516
H275	H	0.217236247060	0.279643412531	0.574536815572
H276	H	0.782763752940	0.779643412531	0.425463184428

H277	H	0.241171373079	0.195953673788	0.519035598619
H278	H	0.758828626921	0.695953673788	0.480964401381
H279	H	0.256067669008	0.292798454802	0.519275152980
H280	H	0.743932330992	0.792798454802	0.480724847020
H281	H	0.349269577070	0.168567081586	0.533216993548
H282	H	0.650730422930	0.668567081586	0.466783006452
H283	H	0.355121364819	0.241314797434	0.503059238479
H284	H	0.644878635181	0.741314797434	0.496940761521
H285	H	0.407188314630	0.256528314927	0.565987147810
H286	H	0.592811685370	0.756528314927	0.434012852190
H287	H	0.372758997129	0.334929843090	0.545790204928
H288	H	0.627241002871	0.834929843090	0.454209795072
H289	H	0.306168127555	0.191135890639	0.623884845136
H290	H	0.693831872445	0.691135890639	0.376115154864
H291	H	0.275421211897	0.101527533411	0.660340572765
H292	H	0.724578788103	0.601527533411	0.339659427234
H293	H	0.210560921405	0.079687332157	0.634562094216
H294	H	0.789439078595	0.579687332157	0.365437905784
H295	H	0.345323221114	0.072258395885	0.590022083127
H296	H	0.654676778886	0.572258395885	0.409977916873
H297	H	0.338302795009	0.920671973104	0.573398662093
H298	H	0.661697204991	0.420671973104	0.426601337907
H299	H	0.455593950893	0.009954032995	0.585683643783
H300	H	0.544406049107	0.509954032995	0.414316356217
H301	H	0.429063079573	0.083609692107	0.549456612927
H302	H	0.570936920427	0.583609692107	0.450543387073
H303	H	0.476097055265	0.880700388383	0.569889348589
H304	H	0.523902944735	0.380700388383	0.430110651411
H305	H	0.493497125596	0.938673334635	0.534855319089
H306	H	0.506502874404	0.438673334635	0.465144680911
H307	H	0.424253315580	0.885463459291	0.535126303763
H308	H	0.575746684420	0.385463459291	0.464873696237
H309	H	0.383507360748	0.813332206160	0.673709834558
H310	H	0.616492639252	0.313332206160	0.326290165441
H311	H	0.443860347304	0.703537428152	0.625050750963
H312	H	0.556139652696	0.203537428152	0.374949249037
H313	H	0.402873637091	0.682661986915	0.663101112137
H314	H	0.597126362909	0.182661986915	0.336898887863
H315	H	0.340362232041	0.671150097943	0.604282370004
H316	H	0.659637767959	0.171150097943	0.395717629996
H317	H	0.305925015368	0.718673233602	0.638553484022
H318	H	0.694074984632	0.218673233602	0.361446515978
H319	H	0.363473756055	0.788457696679	0.576522302111
H320	H	0.636526243945	0.288457696679	0.423477697889
H321	H	0.298497787703	0.817976942267	0.600529925142
H322	H	0.701502212297	0.317976942267	0.399470074858
H323	H	0.460583199654	0.813366021460	0.708385040291
H324	H	0.539416800347	0.313366021460	0.291614959709
H325	H	0.575235796062	0.888269789338	0.690638345669
H326	H	0.424764203938	0.388269789338	0.309361654331
H327	H	0.546396755501	0.779053624712	0.747921307848
H328	H	0.453603244499	0.279053624712	0.252078692152
H329	H	0.614500666548	0.831036748089	0.743103526103
H330	H	0.385499333453	0.331036748089	0.256896473897
H331	H	0.544915686295	0.950907853642	0.743882476610
H332	H	0.455084313705	0.450907853642	0.256117523390
H333	H	0.484603501887	0.890780363028	0.756916260083
H334	H	0.515396498113	0.390780363028	0.243083739917
H335	H	0.537328645985	0.690214338687	0.693302926568
H336	H	0.462671354015	0.190214338687	0.306697073432
H337	H	0.657243693068	0.644284085512	0.678679072637
H338	H	0.342756306932	0.144284085512	0.321320927363
H339	H	0.570841095845	0.555390824958	0.691127507659

H340	H	0.429158904155	0.055390824958	0.308872492341
H341	H	0.612166022549	0.529486227072	0.654293222424
H342	H	0.387833977451	0.029486227072	0.345706777576
H343	H	0.538824808823	0.572992495145	0.651002048579
H344	H	0.461175191177	0.072992495145	0.348997951421
H345	H	0.550199285351	0.717374665136	0.619758687068
H346	H	0.449800714649	0.217374665136	0.380241312932
H347	H	0.629490958111	0.679883690137	0.561551659165
H348	H	0.370509041890	0.179883690137	0.438448340835
H349	H	0.512269595515	0.772228162838	0.572395208511
H350	H	0.487730404485	0.272228162838	0.427604791488
H351	H	0.552651591483	0.776186227794	0.534098924346
H352	H	0.447348408517	0.276186227794	0.465901075654
H353	H	0.506639369512	0.629393359038	0.564101079515
H354	H	0.493360630488	0.129393359038	0.435898920485
H355	H	0.415070934071	0.707104392844	0.556499414153
H356	H	0.584929065929	0.207104392844	0.443500585847
H357	H	0.416564114736	0.639410935605	0.524054126218
H358	H	0.583435885264	0.139410935605	0.475945873782
H359	H	0.438496383230	0.733641875856	0.515495645789
H360	H	0.561503616770	0.233641875856	0.484504354211
H361	H	0.516224129793	0.579419689698	0.506545705961
H362	H	0.483775870207	0.079419689698	0.493454294039
H363	H	0.587881008314	0.616052713146	0.519323854633
H364	H	0.412118991686	0.116052713146	0.480676145367
H365	H	0.540485275084	0.667955237011	0.491456333287
H366	H	0.459514724916	0.167955237011	0.508543666713
H367	H	0.595750155285	0.838333676624	0.614427246726
H368	H	0.404249844715	0.338333676624	0.385572753274
H369	H	0.679855185907	0.930791707783	0.575628565240
H370	H	0.320144814093	0.430791707783	0.424371434760
H371	H	0.633438122020	0.948238176913	0.648099215603
H372	H	0.366561877980	0.448238176913	0.351900784397
H373	H	0.676719830749	0.015891762270	0.625548774268
H374	H	0.323280169251	0.515891762270	0.374451225731
H375	H	0.706573940345	0.799662541700	0.639889300456
H376	H	0.293426059655	0.299662541700	0.360110699544
H377	H	0.835154553765	0.836685484830	0.648263982694
H378	H	0.164845446235	0.336685484830	0.351736017306
H379	H	0.782621032198	0.759521292063	0.690341092138
H380	H	0.217378967802	0.259521292063	0.309658907862
H381	H	0.837736247348	0.701051053252	0.670098976696
H382	H	0.162263752652	0.201051053252	0.329901023304
H383	H	0.758369487690	0.694388910603	0.659310547223
H384	H	0.241630512310	0.194388910603	0.340689452777
H385	H	0.763424343524	0.700165428324	0.591700421172
H386	H	0.236575656476	0.200165428324	0.408299578828
H387	H	0.887028264096	0.668664492555	0.564903366655
H388	H	0.112971735904	0.168664492555	0.435096633345
H389	H	0.834912343053	0.607625338668	0.518356825004
H390	H	0.165087656947	0.107625338668	0.481643174996
H391	H	0.732414042733	0.605841782331	0.516055810059
H392	H	0.267585957267	0.105841782331	0.483944189941
H393	H	0.858141842719	0.528541146934	0.567013969404
H394	H	0.141858157281	0.028541146934	0.432986030595
H395	H	0.786318257034	0.502584843524	0.550365561889
H396	H	0.213681742966	0.002584843524	0.449634438111
H397	H	0.788193886064	0.556929934765	0.587644782296
H398	H	0.211806113936	0.056929934765	0.412355217704
H399	H	0.772959212941	0.805537388227	0.545535564791
H400	H	0.227040787059	0.305537388227	0.454464435209
H401	H	0.834602392138	0.853561764481	0.485453391229
H402	H	0.165397607862	0.353561764481	0.514546608771

H403	H	0.726455264663	0.887762005884	0.507553674132
H404	H	0.273544735337	0.387762005884	0.492446325868
H405	H	0.761308466018	0.950061835018	0.535560150879
H406	H	0.238691533982	0.450061835018	0.464439849121
H407	H	0.746815127640	0.906747643272	0.443460453562
H408	H	0.253184872361	0.406747643272	0.556539546438
H409	H	0.788526030689	0.075572870036	0.516809811695
H410	H	0.211473969311	0.575572870036	0.483190188305
H411	H	0.747963017271	0.002418384071	0.398990555841
H412	H	0.252036982729	0.502418384071	0.601009444159
H413	H	0.789977148695	0.171994859500	0.471878183556
H414	H	0.210022851306	0.671994859500	0.528121816444
H415	H	0.761246643335	0.126115260454	0.386217723300
H416	H	0.238753356665	0.626115260454	0.613782276700
H417	H	0.846951011004	0.923477173540	0.569439803133
H418	H	0.153048988996	0.423477173540	0.430560196867
H419	H	0.939502068581	0.030510135101	0.545535046141
H420	H	0.060497931419	0.530510135101	0.454464953858
H421	H	0.930147948638	0.086756589863	0.601761529639
H422	H	0.069852051362	0.586756589863	0.398238470361
H423	H	0.844078610569	0.973848897610	0.619391689681
H424	H	0.155921389431	0.473848897610	0.380608310319
H425	H	0.852454055827	0.119395703959	0.558024400164
H426	H	0.147545944173	0.619395703959	0.441975599836
H427	H	0.826039245534	0.130666946753	0.599652741404
H428	H	0.173960754466	0.630666946753	0.400347258596
H429	H	0.800497913450	0.052312958195	0.575685324874
H430	H	0.199502086551	0.552312958195	0.424314675126
H431	H	0.946233517477	0.862223799675	0.589197153692
H432	H	0.053766482523	0.362223799675	0.410802846308
H433	H	0.038017849827	0.803898485987	0.608229179661
H434	H	0.961982150173	0.303898485987	0.391770820339
H435	H	0.075675580947	0.892311455507	0.609949554130
H436	H	0.924324419054	0.392311455507	0.390050445870
H437	H	0.942044772216	0.930959241731	0.645519785340
H438	H	0.057955227784	0.430959241731	0.354480214660
H439	H	0.984658118857	0.911591721264	0.714686611028
H440	H	0.015341881143	0.411591721264	0.285313388971
H441	H	0.855648610496	0.944252297465	0.686644029994
H442	H	0.144351389504	0.444252297465	0.313355970006
H443	H	0.877993395507	0.951225975739	0.729264578727
H444	H	0.122006604493	0.451225975739	0.270735421273
H445	H	0.918042376907	0.844000868000	0.754959855418
H446	H	0.081957623093	0.344000868000	0.245040144582
H447	H	0.880873958717	0.696055225439	0.726904089258
H448	H	0.119126041283	0.196055225439	0.273095910742
H449	H	0.941935085709	0.630854637928	0.767351235334
H450	H	0.058064914291	0.130854637928	0.232648764666
H451	H	0.995833495626	0.780987130296	0.790111488543
H452	H	0.004166504374	0.280987130296	0.209888511457
H453	H	0.943223982139	0.726620280280	0.813305671018
H454	H	0.056776017861	0.226620280280	0.186694328982
H455	H	0.047676015613	0.664473644298	0.743477947010
H456	H	0.952323984387	0.164473644298	0.256522052990
H457	H	0.014769446281	0.743233344660	0.724634228446
H458	H	0.985230553719	0.243233344660	0.275365771553
H459	H	0.989093614880	0.652430030709	0.713057444701
H460	H	0.010906385120	0.152430030709	0.286942555299
H461	H	0.021027394232	0.619924432864	0.814732096248
H462	H	0.978972605768	0.119924432864	0.185267903752
H463	H	0.045810470588	0.702892729558	0.835570396408
H464	H	0.954189529412	0.202892729558	0.164429603592
H465	H	0.076031136532	0.680935233310	0.795571291243

H466	H	0.923968863468	0.180935233310	0.204428708757
H467	H	0.813017737131	0.629261408293	0.758476251709
H468	H	0.186982262869	0.129261408293	0.241523748291
H469	H	0.763780445609	0.706374108838	0.818921324833
H470	H	0.236219554391	0.206374108838	0.181078675167
H471	H	0.721816698897	0.562380800263	0.784430726881
H472	H	0.278183301103	0.062380800263	0.215569273118
H473	H	0.648570972577	0.707128916782	0.781599177401
H474	H	0.351429027423	0.207128916782	0.218400822599
H475	H	0.707444743639	0.689641352236	0.751950789754
H476	H	0.292555256361	0.189641352236	0.248049210246
H477	H	0.689143188863	0.563483956379	0.844745874876
H478	H	0.310856811137	0.063483956379	0.155254125124
H479	H	0.624140519936	0.580904445823	0.818901170102
H480	H	0.375859480064	0.080904445823	0.181098829898
H481	H	0.660559770999	0.655651345719	0.840214401983
H482	H	0.339440229001	0.155651345719	0.159785598017
H483	H	0.658340025020	0.573203601800	0.733151902033
H484	H	0.341659974980	0.073203601800	0.266848097967
H485	H	0.601919374727	0.645583432801	0.732971266144
H486	H	0.398080625273	0.145583432801	0.267028733855
H487	H	0.601161163814	0.578656073738	0.765049612189
H488	H	0.398838836186	0.078656073738	0.234950387810
H489	H	0.780440135103	0.664252501593	0.869052503632
H490	H	0.219559864897	0.164252501593	0.130947496368
H491	H	0.827078360540	0.508985048901	0.875789579717
H492	H	0.172921639460	0.008985048901	0.124210420283
H493	H	0.882387098668	0.542609191743	0.927359268794
H494	H	0.117612901332	0.042609191743	0.072640731206
H495	H	0.866952022139	0.706742379478	0.906246968040
H496	H	0.133047977861	0.206742379478	0.093753031960
H497	H	0.825452794571	0.663123988605	0.938933019360
H498	H	0.174547205429	0.163123988605	0.061066980640
H499	H	0.975169067703	0.594567669759	0.897794095979
H500	H	0.024830932297	0.094567669758	0.102205904021
H501	H	0.928600246823	0.621202260774	0.863318411904
H502	H	0.071399753177	0.121202260774	0.136681588096
H503	H	0.937993265846	0.525039823637	0.873558204783
H504	H	0.062006734154	0.025039823637	0.126441795216
H505	H	0.924099940631	0.637352687835	0.970197054105
H506	H	0.075900059369	0.137352687835	0.029802945895
H507	H	0.912585483425	0.734442369292	0.962594142496
H508	H	0.087414516575	0.234442369292	0.037405857504
H509	H	0.966330911381	0.683597718266	0.938319160330
H510	H	0.033669088619	0.183597718266	0.061680839670
H511	H	0.663744990369	0.506291546043	0.944845330330
H512	H	0.336255009631	0.006291546043	0.055154669670
H513	H	0.683602606968	0.375986139972	0.980890446080
H514	H	0.316397393032	0.875986139972	0.019109553920
H515	H	0.661080659336	0.374996789879	0.938011513714
H516	H	0.338919340664	0.874996789879	0.061988486286
H517	H	0.791475398778	0.359456446006	0.966425000972
H518	H	0.208524601222	0.859456446006	0.033574999028
H519	H	0.756999070453	0.303159746241	0.934970138446
H520	H	0.243000929547	0.803159746241	0.065029861554
H521	H	0.833974067299	0.420297085659	0.919008649458
H522	H	0.166025932701	0.920297085659	0.080991350542
H523	H	0.769484712939	0.398955825891	0.892605701229
H524	H	0.230515287061	0.898955825891	0.107394298771
H525	H	0.815641415711	0.514191159121	0.976546523133
H526	H	0.184358584290	0.014191159121	0.023453476867
H527	H	0.771305880289	0.562733269800	0.042575490163
H528	H	0.228694119711	0.062733269800	0.957424509837

H529	H	0.848239094495	0.564416243001	0.026773667230
H530	H	0.151760905505	0.064416243001	0.973226332770
H531	H	0.836651919940	0.688682731406	0.057769013014
H532	H	0.163348080060	0.188682731406	0.942230986986
H533	H	0.810065579497	0.802435872328	0.006048990577
H534	H	0.189934420503	0.302435872328	0.993951009423
H535	H	0.721538746321	0.803929070608	0.045145627622
H536	H	0.278461253679	0.303929070608	0.954854372378
H537	H	0.767451317686	0.885708023929	0.050894134201
H538	H	0.232548682314	0.385708023929	0.949105865799
H539	H	0.772505875701	0.811065675809	0.080391264398
H540	H	0.227494124299	0.311065675809	0.919608735602
H541	H	0.921444081580	0.722136396124	0.029516072908
H542	H	0.078555918420	0.222136396124	0.970483927092
H543	H	0.992209391110	0.859096515546	0.046635837006
H544	H	0.007790608890	0.359096515546	0.953364162994
H545	H	0.019566446640	0.700103412139	0.070952169395
H546	H	0.980433553360	0.200103412139	0.929047830605
H547	H	0.941686602166	0.758351645378	0.098973007193
H548	H	0.058313397834	0.258351645378	0.901026992807
H549	H	0.116368990259	0.768394839153	0.060590517154
H550	H	0.883631009741	0.268394839153	0.939409482846
H551	H	0.101314568532	0.759374748632	0.104226600078
H552	H	0.898685431468	0.259374748632	0.895773399922
H553	H	0.088096207239	0.847289003046	0.083256386958
H554	H	0.911903792761	0.347289003046	0.916743613042
H555	H	0.014162731797	0.890611062414	0.996802624902
H556	H	0.985837268203	0.390611062414	0.003197375098
H557	H	0.045289561741	0.787849778469	0.943318481839
H558	H	0.954710438259	0.287849778469	0.056681518161
H559	H	0.060649144837	0.958051856503	0.952420523484
H560	H	0.939350855163	0.458051856503	0.047579476516
H561	H	0.074823759029	0.911599019396	0.914119931326
H562	H	0.925176240971	0.411599019396	0.085880068674
H563	H	0.244160343362	0.770829816291	0.967946238791
H564	H	0.755839656638	0.270829816291	0.032053761209
H565	H	0.291478146859	0.717154760051	0.907843222391
H566	H	0.708521853141	0.217154760051	0.092156777609
H567	H	0.239047047909	0.662770748588	0.931839101522
H568	H	0.760952952091	0.162770748588	0.068160898478
H569	H	0.209039116198	0.768657918067	0.873554591984
H570	H	0.790960883802	0.268657918067	0.126445408016
H571	H	0.187786596978	0.672602829130	0.876920692582
H572	H	0.812213403022	0.172602829130	0.123079307418
H573	H	0.106880416108	0.782679883082	0.896079752591
H574	H	0.893119583893	0.282679883082	0.103920247409
H575	H	0.112174536253	0.697849328826	0.919518550386
H576	H	0.887825463747	0.197849328826	0.080481449614
H577	H	0.340316617457	0.820834014719	0.953089864084
H578	H	0.659683382544	0.320834014719	0.046910135916
H579	H	0.335807932551	0.978411769749	0.940121442235
H580	H	0.664192067450	0.478411769749	0.059878557765
H581	H	0.402257162975	0.922378083544	0.941328505004
H582	H	0.597742837025	0.422378083544	0.058671494996
H583	H	0.343812289184	0.831493898585	0.879999325095
H584	H	0.656187710816	0.331493898585	0.120000674905
H585	H	0.384918417955	0.940163685433	0.829195825098
H586	H	0.615081582045	0.440163685433	0.170804174902
H587	H	0.392910136770	0.829037828470	0.791246939191
H588	H	0.607089863230	0.329037828470	0.208753060809
H589	H	0.441574592719	0.826650784672	0.826099904846
H590	H	0.558425407281	0.326650784672	0.173900095154
H591	H	0.253139035471	0.899729038322	0.865047407538

H592	H	0.746860964529	0.399729038322	0.134952592462
H593	H	0.191231471071	0.961400916959	0.805657608114
H594	H	0.808768528929	0.461400916959	0.194342391886
H595	H	0.134838936527	0.837215216432	0.848577236145
H596	H	0.865161063473	0.337215216432	0.151422763855
H597	H	0.094328300829	0.894973302797	0.820332724121
H598	H	0.905671699171	0.394973302797	0.179667275879
H599	H	0.231700539233	0.761814490013	0.820174819051
H600	H	0.768299460767	0.261814490013	0.179825180949
H601	H	0.084829982875	0.864168243291	0.763894146023
H602	H	0.915170017125	0.364168243291	0.236105853977
H603	H	0.261950619422	0.690089073860	0.770591107195
H604	H	0.738049380579	0.190089073860	0.229408892805
H605	H	0.113893818925	0.789818125893	0.714326510041
H606	H	0.886106181075	0.289818125893	0.285673489959
H607	H	0.183022320536	0.699952798601	0.692705156918
H608	H	0.816977679464	0.199952798601	0.307294843082
H609	H	0.236543727398	0.056927660422	0.840824944707
H610	H	0.763456272602	0.556927660422	0.159175055293
H611	H	0.179871891568	0.073925400021	0.907600518422
H612	H	0.820128108432	0.573925400021	0.092399481578
H613	H	0.294145011253	0.092005493390	0.897384576973
H614	H	0.705854988747	0.592005493390	0.102615423027
H615	H	0.259570114062	0.175227203402	0.913807553083
H616	H	0.740429885938	0.675227203402	0.086192446917
H617	H	0.277716483126	0.168302634092	0.870313503802
H618	H	0.722283516874	0.668302634092	0.129686496198
H619	H	0.139421347784	0.130766928382	0.829634123597
H620	H	0.860578652216	0.630766928382	0.170365876403
H621	H	0.093402867125	0.275795891903	0.855726088187
H622	H	0.906597132875	0.775795891903	0.144273911813
H623	H	0.117138758885	0.231778246960	0.782695604116
H624	H	0.882861241115	0.731778246960	0.217304395884
H625	H	0.100300771735	0.322362235716	0.798644935148
H626	H	0.899699228265	0.822362235716	0.201355064851
H627	H	0.177985588113	0.360519554988	0.847327712300
H628	H	0.822014411887	0.860519554988	0.152672287700
H629	H	0.254306911562	0.337310676816	0.837020220869
H630	H	0.745693088438	0.837310676816	0.162979779131
H631	H	0.692217347540	0.034641708766	0.866572092650
H632	H	0.307782652460	0.534641708766	0.133427907350
H633	H	0.748286988652	0.026311274434	0.888666312534
H634	H	0.251713011348	0.526311274434	0.111333687466
H635	H	0.266283114802	0.149660292794	0.803484128728
H636	H	0.733716885198	0.649660292794	0.196515871272
H637	H	0.298055901929	0.083903726946	0.786453582932
H638	H	0.701944098071	0.583903726946	0.213546417068
H639	H	0.430958954321	0.034431089016	0.847348922418
H640	H	0.569041045679	0.534431089016	0.152651077582
H641	H	0.492444668717	0.007906142569	0.830186451821
H642	H	0.507555331283	0.507906142569	0.169813548179
H643	H	0.316675786093	0.345617682874	0.728774710097
H644	H	0.683324213907	0.845617682874	0.271225289903
H645	H	0.313407201490	0.434307039983	0.735672326944
H646	H	0.686592798510	0.934307039983	0.264327673056
H647	H	0.248911773417	0.409004261949	0.651338521626
H648	H	0.751088226584	0.909004261949	0.348661478374
H649	H	0.260639843844	0.426967784239	0.689140541910
H650	H	0.739360156156	0.926967784239	0.310859458090
H651	H	0.245366691711	0.540246891154	0.658792131545
H652	H	0.754633308289	0.040246891154	0.341207868455
H653	H	0.283884440375	0.610553940104	0.668674412875
H654	H	0.716115559625	0.110553940104	0.331325587125

H655	H	0.335039783154	0.602532491248	0.719040771234
H656	H	0.664960216846	0.102532491248	0.280959228766
H657	H	0.291139197010	0.668591107565	0.710335498684
H658	H	0.708860802990	0.168591107565	0.289664501316
H659	H	0.392620610655	0.510694770318	0.739682193354
H660	H	0.607379389345	0.010694770318	0.260317806646
H661	H	0.350460446097	0.528475124777	0.769735527461
H662	H	0.649539553903	0.028475124777	0.230264472539
H663	H	0.683946331011	0.290752058229	0.790891503926
H664	H	0.316053668989	0.790752058229	0.209108496074
H665	H	0.716174821269	0.312751585122	0.823615507189
H666	H	0.283825178732	0.812751585122	0.176384492811
H667	H	0.930829952479	0.502917266845	0.755414687305
H668	H	0.069170047521	0.002917266845	0.244585312695
H669	H	0.937189564756	0.520524994074	0.716594959758
H670	H	0.062810435244	0.020524994074	0.283405040242
H671	H	0.778832014088	0.563787050505	0.702434494842
H672	H	0.221167985912	0.063787050505	0.297565505158
H673	H	0.834746008061	0.539551602673	0.726424566805
H674	H	0.165253991939	0.039551602673	0.273575433195
H675	H	0.747799211735	0.507175062975	0.648661819111
H676	H	0.252200788266	0.007175062975	0.351338180889
H677	H	0.725981935652	0.590373320927	0.645772709663
H678	H	0.274018064348	0.090373320927	0.354227290337
H679	H	0.041509653345	0.190398702212	0.562328854586
H680	H	0.958490346655	0.690398702212	0.437671145414
H681	H	0.012895483381	0.112614956815	0.568473067223
H682	H	0.987104516619	0.612614956815	0.431526932777
H683	H	0.545535581964	0.212519126210	0.759985090916
H684	H	0.454464418036	0.712519126210	0.240014909084
H685	H	0.590491203992	0.277817795988	0.750345021125
H686	H	0.409508796008	0.777817795988	0.249654978875
H687	H	0.497990539845	0.326910438948	0.727898274303
H688	H	0.502009460155	0.826910438948	0.272101725697
H689	H	0.504502216024	0.367960903945	0.693478917206
H690	H	0.495497783976	0.867960903945	0.306521082794
H691	H	0.472395019598	0.491309452167	0.767564860329
H692	H	0.527604980402	0.991309452167	0.232435139671
H693	H	0.480558134686	0.449060970027	0.732865612511
H694	H	0.519441865314	0.949060970027	0.267134387489
H695	H	0.329747018135	0.206420074737	0.733736050445
H696	H	0.670252981865	0.706420074737	0.266263949555
H697	H	0.359567078633	0.242543637466	0.702875061463
H698	H	0.640432921367	0.742543637466	0.297124938537
H699	H	0.140874898298	0.190035934762	0.557389085319
H700	H	0.859125101702	0.690035934762	0.442610914681
H701	H	0.110098612758	0.243653090041	0.532016244570
H702	H	0.889901387242	0.743653090041	0.467983755430
H703	H	0.101675185689	0.009202058245	0.602631576275
H704	H	0.898324814311	0.509202058245	0.397368423725
H705	H	0.165466635804	0.035247603338	0.591165882807
H706	H	0.834533364196	0.535247603338	0.408834117193
H707	H	0.255874222131	0.869814933274	0.655764238760
H708	H	0.744125777869	0.369814933274	0.344235761240
H709	H	0.214776870786	0.797840150583	0.660902007322
H710	H	0.785223129214	0.297840150583	0.339097992678
H711	H	0.256791380171	0.059929299635	0.537358127283
H712	H	0.743208619829	0.559929299635	0.462641872717
H713	H	0.325042295543	0.044208088389	0.527740684484
H714	H	0.674957704458	0.544208088389	0.472259315516
H715	H	0.372924840663	0.787966774762	0.734532559744
H716	H	0.627075159337	0.287966774762	0.265467440256
H717	H	0.423901698920	0.724490310605	0.736887877057

H718	H	0.576098301080	0.224490310605	0.263112122943
H719	H	0.284795551710	0.852560509274	0.746557138877
H720	H	0.715204448290	0.352560509274	0.253442861123
H721	H	0.280553875634	0.830166530908	0.707401991561
H722	H	0.719446124366	0.330166530908	0.292598008439
H723	H	0.482552195123	0.598220267578	0.733349584459
H724	H	0.517447804878	0.098220267578	0.266650415541
H725	H	0.502887540506	0.666721143554	0.754321388700
H726	H	0.497112459494	0.166721143554	0.245678611300
H727	H	0.502991209764	0.112292644686	0.799818264227
H728	H	0.497008790236	0.612292644686	0.200181735773
H729	H	0.554305564578	0.064758242900	0.785220230266
H730	H	0.445694435422	0.564758242900	0.214779769734
H731	H	0.528673782179	0.751778432606	0.798139768956
H732	H	0.471326217821	0.251778432606	0.201860231044
H733	H	0.473990390163	0.696786499484	0.803697519798
H734	H	0.526009609837	0.196786499484	0.196302480202
H735	H	0.559672696836	0.714031917594	0.845424207642
H736	H	0.440327303165	0.214031917594	0.154575792358
H737	H	0.574408590176	0.797117664298	0.847939379153
H738	H	0.425591409824	0.297117664298	0.152060620847
H739	H	0.144916476312	0.704884562876	0.636588881870
H740	H	0.855083523688	0.204884562876	0.363411118130
H741	H	0.109147253348	0.765335808993	0.658449536198
H742	H	0.890852746652	0.265335808993	0.341550463802
H743	H	0.955519590103	0.702205375274	0.619687713634
H744	H	0.044480409897	0.202205375274	0.380312286365
H745	H	0.971030264823	0.717068247407	0.655865590060
H746	H	0.028969735177	0.217068247407	0.344134409940
H747	H	0.910510980641	0.516071369235	0.809149489383
H748	H	0.089489019359	0.016071369235	0.190850510617
H749	H	0.968034141978	0.468358200427	0.817714305959
H750	H	0.031965858022	0.968358200427	0.182285694041
H751	H	0.713280347888	0.450404872845	0.830716509390
H752	H	0.286719652112	0.950404872845	0.169283490610
H753	H	0.782829906750	0.456850100205	0.824258452885
H754	H	0.217170093250	0.956850100205	0.175741547115
H755	H	0.878366118365	0.422020722065	0.984045208411
H756	H	0.121633881635	0.922020722065	0.015954791589
H757	H	0.931960547485	0.472839456085	0.971101445866
H758	H	0.068039452515	0.972839456084	0.028898554134
H759	H	0.049552672516	0.485649539751	0.979649595522
H760	H	0.950447327484	0.985649539751	0.020350404478
H761	H	0.002911549320	0.528916506276	0.004106760705
H762	H	0.997088450680	0.028916506276	0.995893239295
H763	H	0.973887634241	0.598313999859	0.052638731766
H764	H	0.026112365759	0.098313999859	0.947361268234
H765	H	0.006392561036	0.640779947123	0.022387769486
H766	H	0.993607438964	0.140779947123	0.977612230514
H767	H	0.934496626085	0.546120445517	0.106978588412
H768	H	0.065503373915	0.046120445517	0.893021411588
H769	H	0.972882979868	0.614825566463	0.117751279893
H770	H	0.027117020132	0.114825566463	0.882248720107
H771	H	0.118479188112	0.550550201191	0.098322713435
H772	H	0.881520811889	0.050550201191	0.901677286565
H773	H	0.051354973059	0.575302593157	0.093794910429
H774	H	0.948645026941	0.075302593157	0.906205089571
H775	H	0.354388464314	0.686569211038	0.966555700938
H776	H	0.645611535686	0.186569211038	0.033444299062
H777	H	0.353277540915	0.724326010870	0.001150968688
H778	H	0.646722459085	0.224326010870	0.998849031312
H779	H	0.300663774069	0.636656101945	0.855862585639
H780	H	0.699336225932	0.136656101945	0.144137414361

H781	H	0.249167631361	0.576990959321	0.862042955119
H782	H	0.750832368639	0.076990959321	0.137957044881
H783	H	0.382809606879	0.598652420165	0.809988575473
H784	H	0.617190393121	0.098652420165	0.190011424527
H785	H	0.345163292690	0.540080548736	0.828409626758
H786	H	0.654836707310	0.040080548736	0.171590373242
H787	H	0.994093483457	0.366051628948	0.868521983114
H788	H	0.005906516543	0.866051628948	0.131478016886
H789	H	0.000921091875	0.349745812853	0.831440850918
H790	H	0.999078908125	0.849745812853	0.168559149082
H791	H	0.078223819813	0.409249621607	0.864468159414
H792	H	0.921776180187	0.909249621607	0.135531840586
H793	H	0.114887116814	0.403230446400	0.898387207972
H794	H	0.885112883186	0.903230446400	0.101612792028
H795	H	0.137602083213	0.572299305165	0.841267368901
H796	H	0.862397916787	0.072299305165	0.158732631099
H797	H	0.144872388171	0.509786933446	0.867691740591
H798	H	0.855127611829	0.009786933446	0.132308259409
C1	C	0.686001285101	0.810401429759	0.912889440538
C2	C	0.313998714899	0.310401429759	0.087110559462
C3	C	0.723229848918	0.815095288748	0.880638503843
C4	C	0.276770151082	0.315095288748	0.119361496157
C5	C	0.727606244869	0.817980302706	0.944324252072
C6	C	0.272393755131	0.317980302706	0.055675747928
C7	C	0.752899677476	0.897029341292	0.948733858068
C8	C	0.247100322524	0.397029341292	0.051266141932
C9	C	0.769903255926	0.895457944756	0.838905133836
C10	C	0.230096744074	0.395457944756	0.161094866164
C11	C	0.827424540276	0.938207648291	0.849648330972
C12	C	0.172575459724	0.438207648291	0.150351669028
C13	C	0.736734568752	0.932444845017	0.808602097307
C14	C	0.263265431248	0.432444845017	0.191397902693
C15	C	0.701934108444	0.872104647972	0.789517790255
C16	C	0.298065891556	0.372104647972	0.210482209745
C17	C	0.939635129854	0.943987583183	0.837965506548
C18	C	0.060364870146	0.443987583183	0.162034493452
C19	C	0.966072883651	0.961300512655	0.803053495957
C20	C	0.033927116349	0.461300512655	0.196946504043
C21	C	0.981915328352	0.884583336815	0.854009528190
C22	C	0.018084671648	0.384583336815	0.145990471810
C23	C	0.025831050398	0.041312959449	0.766645964287
C24	C	0.974168949602	0.541312959449	0.233354035712
C25	C	0.095887390839	0.047793124476	0.767507791008
C26	C	0.904112609161	0.547793124476	0.232492208992
C27	C	0.996334958230	0.113521867686	0.753280994472
C28	C	0.003665041770	0.613521867686	0.246719005528
C29	C	0.193923083150	0.024764904250	0.738804497461
C30	C	0.806076916851	0.524764904250	0.261195502539
C31	C	0.221139337905	0.102097209243	0.732190833572
C32	C	0.778860662095	0.602097209243	0.267809166428
C33	C	0.208223846377	0.969476546767	0.710396990919
C34	C	0.791776153623	0.469476546767	0.289603009081
C35	C	0.154065253511	0.978767578953	0.686400227698
C36	C	0.845934746489	0.478767578953	0.313599772302
C37	C	0.099112667163	0.987785304852	0.709905035575
C38	C	0.900887332837	0.487785304852	0.290094964425
C39	C	0.200909203519	0.232120735658	0.717101987437
C40	C	0.799090796481	0.732120735658	0.282898012563
C41	C	0.145251302387	0.282016993809	0.722933557112
C42	C	0.854748697613	0.782016993809	0.277066442887
C43	C	0.229800906834	0.248553805858	0.683053238035
C44	C	0.770199093166	0.748553805858	0.316946761965
C45	C	0.114053247212	0.411887187487	0.733294813646

C46	C	0.885946752788	0.911887187487	0.266705186354
C47	C	0.074539046725	0.421453871169	0.701717126225
C48	C	0.925460953275	0.921453871169	0.298282873775
C49	C	0.146143729633	0.485195504369	0.743748387542
C50	C	0.853856270367	0.985195504369	0.256251612458
C51	C	0.178128974250	0.476633290155	0.777990086333
C52	C	0.821871025750	0.976633290155	0.222009913667
C53	C	0.100898679140	0.550260587477	0.742964992663
C54	C	0.899101320860	0.050260587477	0.257035007337
C55	C	0.216167251099	0.545129083418	0.787448728014
C56	C	0.783832748901	0.045129083418	0.212551271986
C57	C	0.067083101925	0.432060113264	0.641290231217
C58	C	0.932916898075	0.932060113264	0.358709768783
C59	C	0.034599611132	0.359050977267	0.631692736328
C60	C	0.965400388868	0.859050977267	0.368307263672
C61	C	0.104657631131	0.470100404706	0.613061547328
C62	C	0.895342368869	0.970100404706	0.386938452672
C63	C	0.079630166470	0.454865928644	0.577823148312
C64	C	0.920369833530	0.954865928644	0.422176851688
C65	C	0.107440114947	0.554231416273	0.620908935408
C66	C	0.892559885053	0.054231416273	0.379091064592
C67	C	0.020964268804	0.225610142205	0.638450112702
C68	C	0.979035731196	0.725610142205	0.361549887298
C69	C	0.961776778276	0.235027174048	0.659347541807
C70	C	0.038223221724	0.735027174048	0.340652458192
C71	C	0.056905944930	0.154799219445	0.647210497594
C72	C	0.943094055070	0.654799219445	0.352789502406
C73	C	0.913329043030	0.287206744852	0.709970577815
C74	C	0.086670956970	0.787206744852	0.290029422185
C75	C	0.872537928012	0.344287891009	0.691757068116
C76	C	0.127462071988	0.844287891009	0.308242931884
C77	C	0.932759179639	0.316240804113	0.744729207951
C78	C	0.067240820361	0.816240804113	0.255270792049
C79	C	0.879138148932	0.341136669156	0.767136921760
C80	C	0.120861851068	0.841136669156	0.232863078240
C81	C	0.834758168197	0.279957760413	0.778544648271
C82	C	0.165241831803	0.779957760413	0.221455351729
C83	C	0.839132463785	0.167517113706	0.815298461984
C84	C	0.160867536215	0.667517113706	0.184701538016
C85	C	0.863238351379	0.455904149905	0.657698816614
C86	C	0.136761648621	0.955904149905	0.342301183386
C87	C	0.825526206716	0.417761530638	0.629895894543
C88	C	0.174473793284	0.917761530638	0.370104105457
C89	C	0.902062359413	0.518664135749	0.642267889423
C90	C	0.097937640587	0.018664135749	0.357732110577
C91	C	0.828353676217	0.332486950925	0.582068639631
C92	C	0.171646323783	0.832486950925	0.417931360369
C93	C	0.778113002215	0.279402179297	0.595272534743
C94	C	0.221886997785	0.779402179297	0.404727465257
C95	C	0.877623111753	0.291765112364	0.561459062937
C96	C	0.122376888247	0.791765112364	0.438540937063
C97	C	0.921961529740	0.347412932326	0.544313007043
C98	C	0.078038470260	0.847412932326	0.455686992957
C99	C	0.748910876201	0.193414179828	0.640924948664
C100	C	0.251089123800	0.693414179828	0.359075051336
C101	C	0.690698364097	0.234956286871	0.651803901507
C102	C	0.309301635903	0.734956286871	0.348196098493
C103	C	0.782630212756	0.157183786758	0.670827214762
C104	C	0.217369787244	0.657183786758	0.329172785238
C105	C	0.741973549580	0.112988739186	0.694136817334
C106	C	0.258026450420	0.612988739186	0.305863182666
C107	C	0.712950574196	0.148265768377	0.721337794494
C108	C	0.287049425804	0.648265768377	0.278662205506

C109	C	0.732582623377	0.036140890611	0.689256289129
C110	C	0.267417376623	0.536140890611	0.310743710871
C111	C	0.675271882332	0.107704000719	0.743019271243
C112	C	0.324728117668	0.607704000719	0.256980728757
C113	C	0.694369819902	0.995364464838	0.710743588347
C114	C	0.305630180098	0.495364464838	0.289256411652
C115	C	0.665572974720	0.031359251275	0.737646841952
C116	C	0.334427025280	0.531359251275	0.262353158048
C117	C	0.644584123521	0.340635424321	0.680160606449
C118	C	0.355415876479	0.840635424321	0.319839393550
C119	C	0.604070888660	0.366993728350	0.650941026209
C120	C	0.395929111340	0.866993728350	0.349058973791
C121	C	0.661632506043	0.406464643800	0.702922470406
C122	C	0.338367493957	0.906464643800	0.297077529594
C123	C	0.686734913573	0.382461539315	0.736853777446
C124	C	0.313265086427	0.882461539315	0.263146222554
C125	C	0.596819454253	0.402423485640	0.591841172814
C126	C	0.403180545748	0.902423485640	0.408158827186
C127	C	0.563508440292	0.332698039138	0.578733713148
C128	C	0.436491559708	0.832698039138	0.421266286851
C129	C	0.637892318580	0.440011479240	0.565048962067
C130	C	0.362107681420	0.940011479240	0.434951037933
C131	C	0.609104599645	0.436179945177	0.530098162765
C132	C	0.390895400355	0.936179945177	0.469901837235
C133	C	0.650424684964	0.520556076801	0.575906403483
C134	C	0.349575315036	0.020556076801	0.424093596517
C135	C	0.569005434074	0.198712694148	0.566208322588
C136	C	0.430994565927	0.698712694148	0.433791677412
C137	C	0.512734753366	0.180248443482	0.588440669113
C138	C	0.487265246634	0.680248443482	0.411559330887
C139	C	0.620106440754	0.139298392207	0.567585990735
C140	C	0.379893559246	0.639298392207	0.432414009265
C141	C	0.468492673033	0.184531882703	0.644960314912
C142	C	0.531507326967	0.684531882703	0.355039685087
C143	C	0.414694652441	0.239123194578	0.642044850561
C144	C	0.585305347559	0.739123194578	0.357955149439
C145	C	0.494622598096	0.186669010726	0.680782029896
C146	C	0.505377401904	0.686669010726	0.319217970104
C147	C	0.535227698633	0.119339814436	0.689266067374
C148	C	0.464772301367	0.619339814436	0.310733932626
C149	C	0.499309256590	0.046929147061	0.694470167905
C150	C	0.500690743410	0.546929147061	0.305529832095
C151	C	0.414488296547	0.005606493239	0.732388608714
C152	C	0.585511703453	0.505606493239	0.267611391285
C153	C	0.370613494188	0.357285074793	0.623785101737
C154	C	0.629386505812	0.857285074793	0.376214898263
C155	C	0.311869773624	0.325576001606	0.608164740300
C156	C	0.688130226376	0.825576001606	0.391835259700
C157	C	0.389884913077	0.429417032142	0.605967484709
C158	C	0.610115086923	0.929417032142	0.394032515291
C159	C	0.433568350939	0.480615347397	0.625757804312
C160	C	0.566431649061	0.980615347397	0.374242195688
C161	C	0.449425436799	0.546298083105	0.602679326407
C162	C	0.550574563201	0.046298083105	0.397320673593
C163	C	0.406468309534	0.506039661962	0.659434605116
C164	C	0.593531690466	0.006039661962	0.340565394884
C165	C	0.257850592556	0.245516407494	0.568575422497
C166	C	0.742149407444	0.745516407494	0.431424577503
C167	C	0.246010046708	0.169327861276	0.585134127930
C168	C	0.753989953292	0.669327861276	0.414865872070
C169	C	0.268571595618	0.239600302779	0.530908084704
C170	C	0.731428404382	0.739600302779	0.469091915296
C171	C	0.337808530981	0.227348976721	0.528103589226

C172	C	0.662191469019	0.727348976721	0.471896410774
C173	C	0.364514345584	0.278023473477	0.555364929139
C174	C	0.635485654416	0.778023473477	0.444635070861
C175	C	0.260312856066	0.089871671215	0.634612204672
C176	C	0.739687143934	0.589871671215	0.365387795328
C177	C	0.290732929433	0.017264662862	0.623987200863
C178	C	0.709267070567	0.517264662862	0.376012799137
C179	C	0.367945777856	0.956839187814	0.588760425241
C180	C	0.632054222144	0.456839187814	0.411239574759
C181	C	0.392221913305	0.916247243766	0.619938236195
C182	C	0.607778086695	0.416247243766	0.380061763805
C183	C	0.423897623527	0.983086927420	0.567986604108
C184	C	0.576102376473	0.483086927420	0.432013395892
C185	C	0.456067800030	0.917860990003	0.550868045739
C186	C	0.543932199970	0.417860990003	0.449131954261
C187	C	0.411236785620	0.801900771528	0.651231792366
C188	C	0.588763214380	0.301900771528	0.348768207634
C189	C	0.478079487973	0.822371291837	0.657574976298
C190	C	0.521920512027	0.322371291837	0.342425023702
C191	C	0.404736413786	0.719417296929	0.641110045221
C192	C	0.595263586214	0.219417296929	0.358889954779
C193	C	0.344850493743	0.719218588246	0.621128281397
C194	C	0.655149506257	0.219218588246	0.378871718602
C195	C	0.344796783767	0.793756316260	0.602060931577
C196	C	0.655203216233	0.293756316260	0.397939068423
C197	C	0.556976885921	0.834716020389	0.700085274928
C198	C	0.443023114080	0.334716020389	0.299914725072
C199	C	0.598168485250	0.775815839048	0.683571349939
C200	C	0.401831514750	0.275815839048	0.316428650061
C201	C	0.564755600256	0.832093615663	0.738349622135
C202	C	0.435244399744	0.332093615663	0.261650377865
C203	C	0.534809978452	0.898253147426	0.756824504064
C204	C	0.465190021548	0.398253147426	0.243175495936
C205	C	0.555225213840	0.905185072980	0.793388110251
C206	C	0.444774786160	0.405185072980	0.206611889749
C207	C	0.613968724432	0.648027564078	0.664666257582
C208	C	0.386031275568	0.148027564078	0.335333742418
C209	C	0.632965701182	0.669125500291	0.628656412024
C210	C	0.367034298818	0.169125500291	0.371343587976
C211	C	0.581815049911	0.571927144929	0.665286874400
C212	C	0.418184950089	0.071927144929	0.334713125600
C213	C	0.602152724104	0.722822223105	0.574402488421
C214	C	0.397847275896	0.222822223105	0.425597511579
C215	C	0.643625180591	0.791933033476	0.574545241148
C216	C	0.356374819409	0.291933033476	0.425454758852
C217	C	0.542006233876	0.739347835231	0.555546946415
C218	C	0.457993766125	0.239347835231	0.444453053585
C219	C	0.507667594605	0.669354914916	0.543164078439
C220	C	0.492332405395	0.169354914916	0.456835921560
C221	C	0.440880433133	0.688399274304	0.534333558327
C222	C	0.559119566867	0.188399274304	0.465666441673
C223	C	0.540043207250	0.631334300102	0.513684467481
C224	C	0.459956792751	0.131334300102	0.486315532519
C225	C	0.672691626116	0.907805692702	0.600948414541
C226	C	0.327308373884	0.407805692702	0.399051585459
C227	C	0.738109074171	0.886434328448	0.611970984275
C228	C	0.261890925829	0.386434328448	0.388029015725
C229	C	0.644506268775	0.969023291314	0.622806884074
C230	C	0.355493731225	0.469023291314	0.377193115926
C231	C	0.804543358055	0.792290232525	0.639239898897
C232	C	0.195456641946	0.292290232525	0.360760101103
C233	C	0.838858776923	0.759996347591	0.608677116042
C234	C	0.161141223077	0.259996347591	0.391322883958

C235	C	0.795204744302	0.732769261833	0.666455297757
C236	C	0.204795255698	0.232769261833	0.333544702243
C237	C	0.840350613743	0.686152311814	0.557920353677
C238	C	0.159649386258	0.186152311814	0.442079646323
C239	C	0.851684087173	0.749314749384	0.532380976712
C240	C	0.148315912827	0.249314749384	0.467619023288
C241	C	0.808573139764	0.618431013519	0.541609226137
C242	C	0.191426860236	0.118431013519	0.458390773863
C243	C	0.810725376382	0.548062416282	0.563284662328
C244	C	0.189274623618	0.048062416282	0.436715337672
C245	C	0.824581050996	0.873424272040	0.510970417854
C246	C	0.175418949004	0.373424272040	0.489029582146
C247	C	0.882440110287	0.917464730733	0.521639399295
C248	C	0.117559889713	0.417464730733	0.478360600705
C249	C	0.766814997975	0.924211315325	0.510714251883
C250	C	0.233185002025	0.424211315325	0.489285748117
C251	C	0.767418154060	0.983622432164	0.483730846231
C252	C	0.232581845940	0.483622432164	0.516269153769
C253	C	0.755939323268	0.964843851775	0.450216374659
C254	C	0.244060676732	0.464843851775	0.549783625341
C255	C	0.779412117207	0.059001985164	0.490974683553
C256	C	0.220587882793	0.559001985164	0.509025316447
C257	C	0.756341764243	0.018525863313	0.424906007640
C258	C	0.243658235757	0.518525863313	0.575093992360
C259	C	0.780164894028	0.113528221399	0.465947481793
C260	C	0.219835105972	0.613528221399	0.534052518207
C261	C	0.768851468616	0.093774497738	0.432511377550
C262	C	0.231148531384	0.593774497739	0.567488622450
C263	C	0.925112252409	0.995440524677	0.566817170743
C264	C	0.074887747591	0.495440524677	0.433182829257
C265	C	0.984120200335	0.965860042886	0.582422660387
C266	C	0.015879799665	0.465860042886	0.417577339612
C267	C	0.895155029192	0.046110467353	0.594021774492
C268	C	0.104844970808	0.546110467353	0.405978225508
C269	C	0.839765240727	0.089094708294	0.581017115457
C270	C	0.160234759273	0.589094708294	0.418982884543
C271	C	0.031710167750	0.863387126362	0.614359790381
C272	C	0.968289832250	0.363387126362	0.385640209619
C273	C	0.018299849586	0.865505406901	0.652041963274
C274	C	0.981700150414	0.365505406901	0.347958036726
C275	C	0.946882931163	0.900040510747	0.697216172225
C276	C	0.053117068837	0.400040510747	0.302783827774
C277	C	0.924862770893	0.819004869368	0.704884583727
C278	C	0.075137229107	0.319004869368	0.295115416273
C279	C	0.894626750229	0.956076855919	0.703388716375
C280	C	0.105373249771	0.456076855919	0.296611283625
C281	C	0.896997831678	0.728554669065	0.748517702029
C282	C	0.103002168322	0.228554669065	0.251482297971
C283	C	0.847365732790	0.730076031834	0.775494834533
C284	C	0.152634267211	0.230076031834	0.224505165467
C285	C	0.956121582312	0.688784853398	0.762056556732
C286	C	0.043878417688	0.188784853398	0.237943443268
C287	C	0.980999226449	0.722894546929	0.794908216757
C288	C	0.019000773551	0.222894546929	0.205091783243
C289	C	0.004449461709	0.687055926412	0.734194409629
C290	C	0.995550538291	0.187055926412	0.265805590371
C291	C	0.033995080068	0.678972749489	0.810910893825
C292	C	0.966004919932	0.178972749489	0.189089106175
C293	C	0.772126350789	0.653332242257	0.805907606296
C294	C	0.227873649211	0.153332242257	0.194092393703
C295	C	0.804354674215	0.598536066809	0.829573804392
C296	C	0.195645325785	0.098536066809	0.170426195608
C297	C	0.710115373663	0.616840762713	0.795756210169

C298	C	0.289884626337	0.116840762713	0.204243789831
C299	C	0.674925234206	0.662149630834	0.769207217869
C300	C	0.325074765794	0.162149630834	0.230792782131
C301	C	0.669110367770	0.603071063784	0.826610541322
C302	C	0.330889632230	0.103071063784	0.173389458678
C303	C	0.631871537263	0.612120519023	0.748989116440
C304	C	0.368128462737	0.112120519023	0.251010883560
C305	C	0.817479121501	0.562274294268	0.888359970444
C306	C	0.182520878499	0.062274294268	0.111640029555
C307	C	0.761514311710	0.553400116941	0.911579263106
C308	C	0.238485688290	0.053400116941	0.088420736894
C309	C	0.876522483658	0.585479632716	0.907832202053
C310	C	0.123477516342	0.085479632716	0.092167797947
C311	C	0.869470301955	0.662055014903	0.925346194521
C312	C	0.130529698045	0.162055014903	0.074653805479
C313	C	0.932416279344	0.581382223619	0.884323944935
C314	C	0.067583720656	0.081382223619	0.115676055065
C315	C	0.920798462288	0.680036716155	0.950330813826
C316	C	0.079201537712	0.180036716155	0.049669186174
C317	C	0.706063458037	0.478135138608	0.952914815061
C318	C	0.293936541963	0.978135138608	0.047085184939
C319	C	0.721753742217	0.515559451276	0.986664841239
C320	C	0.278246257783	0.015559451276	0.013335158761
C321	C	0.697589876135	0.392611677596	0.955450459064
C322	C	0.302410123865	0.892611677596	0.044549540936
C323	C	0.760224246299	0.360518491319	0.944854714434
C324	C	0.239775753701	0.860518491319	0.055145285566
C325	C	0.783895603140	0.415082555393	0.918102717583
C326	C	0.216104396860	0.915082555393	0.081897282417
C327	C	0.799969192222	0.576469154867	0.020622427251
C328	C	0.200030807778	0.076469154867	0.979377572749
C329	C	0.792712738599	0.660594179132	0.013174154160
C330	C	0.207287261401	0.160594179132	0.986825845840
C331	C	0.817657503657	0.789040056325	0.032690618590
C332	C	0.182342496343	0.289040056325	0.967309381410
C333	C	0.881070197794	0.822310720299	0.041389718404
C334	C	0.118929802206	0.322310720299	0.958610281596
C335	C	0.766467253787	0.824532095464	0.053684808626
C336	C	0.233532746213	0.324532095464	0.946315191374
C337	C	0.992952492915	0.798487568229	0.042241623012
C338	C	0.007047507085	0.298487568229	0.957758376988
C339	C	0.028098561413	0.781310011453	0.009973199787
C340	C	0.971901438587	0.281310011453	0.990026800213
C341	C	0.019982684353	0.761242734865	0.073802191196
C342	C	0.980017315647	0.261242734865	0.926197808804
C343	C	0.085427571714	0.785942992952	0.080948532338
C344	C	0.914572428286	0.285942992952	0.919051467662
C345	C	0.062407940036	0.836912714542	0.957039170203
C346	C	0.937592059964	0.336912714542	0.042960829797
C347	C	0.132334212003	0.830702270093	0.961748637746
C348	C	0.867665787997	0.330702270093	0.038251362254
C349	C	0.048075277724	0.908549720443	0.937469312094
C350	C	0.951924722276	0.408549720443	0.062530687906
C351	C	0.230325395432	0.782026617913	0.941965889408
C352	C	0.769674604568	0.282026617913	0.058034110592
C353	C	0.261002161645	0.855214023207	0.930241618262
C354	C	0.738997838355	0.355214023207	0.069758381738
C355	C	0.244747979398	0.716004827930	0.918188216172
C356	C	0.755252020602	0.216004827930	0.081811783828
C357	C	0.195482484079	0.724170565351	0.891051485501
C358	C	0.804517515921	0.224170565351	0.108948514498
C359	C	0.138144314534	0.747162548800	0.910734552194
C360	C	0.861855685466	0.247162548800	0.089265447806

C361	C	0.356744248901	0.927492316028	0.929598337055
C362	C	0.643255751099	0.427492316028	0.070401662945
C363	C	0.366469193059	0.940958531243	0.892098952724
C364	C	0.633530806941	0.440958531243	0.107901047276
C365	C	0.358603041261	0.889311371696	0.834835688376
C366	C	0.641396958739	0.389311371696	0.165164311624
C367	C	0.295162547165	0.904589462925	0.819172447863
C368	C	0.704837452835	0.404589462925	0.180827552137
C369	C	0.392877153085	0.823529888702	0.818683152512
C370	C	0.607122846915	0.323529888702	0.181316847488
C371	C	0.369258915492	0.744651280940	0.826879893133
C372	C	0.630741084508	0.244651280940	0.173120106867
C373	C	0.185533637653	0.932454073514	0.829714507479
C374	C	0.814466362347	0.432454073514	0.170285492521
C375	C	0.163804284886	0.990795244846	0.855348580076
C376	C	0.836195715114	0.490795244847	0.144651419924
C377	C	0.139095282753	0.868704508647	0.824996566280
C378	C	0.860904717247	0.368704508647	0.175003433720
C379	C	0.155466932977	0.818910174688	0.795872421252
C380	C	0.844533067023	0.318910174688	0.204127578748
C381	C	0.204907473667	0.768520623331	0.797326840276
C382	C	0.795092526333	0.268520623331	0.202673159724
C383	C	0.123739124146	0.825619069762	0.765582751794
C384	C	0.876260875854	0.325619069762	0.234417248206
C385	C	0.222236498304	0.727180307548	0.769361054805
C386	C	0.777763501696	0.227180307548	0.230638945195
C387	C	0.139843394877	0.783555545332	0.737418900907
C388	C	0.860156605123	0.283555545332	0.262581099093
C389	C	0.189141828705	0.733492016209	0.739370036353
C390	C	0.810858171295	0.233492016209	0.260629963647
C391	C	0.197236943956	0.103551054450	0.885313901892
C392	C	0.802763056044	0.603551054450	0.114686098108
C393	C	0.150740711852	0.165839261192	0.878124557013
C394	C	0.849259288148	0.665839261192	0.121875442987
C395	C	0.261017118520	0.137051878869	0.892298119470
C396	C	0.738982881480	0.637051878869	0.107701880530
C397	C	0.092827455112	0.233957859579	0.835642960395
C398	C	0.907172544888	0.733957859579	0.164357039604
C399	C	0.024691853496	0.212748750420	0.830670665550
C400	C	0.975308146504	0.712748750420	0.169329334450
C401	C	0.122810933772	0.268811140786	0.804322889002
C402	C	0.877189066228	0.768811140786	0.195677110997
C403	C	0.191699966870	0.279579083287	0.809815726470
C404	C	0.808300033130	0.779579083287	0.190184273530
N1	N	0.655528592047	0.735920891943	0.913616118429
N2	N	0.344471407954	0.235920891943	0.086383881571
N3	N	0.728583467397	0.882997056067	0.866841802912
N4	N	0.271416532603	0.382997056067	0.133158197087
N5	N	0.877813944435	0.915557136454	0.833179513374
N6	N	0.122186055565	0.415557136454	0.166820486626
N7	N	0.002248365501	0.021594412760	0.799584777492
N8	N	0.997751634499	0.521594412760	0.200415222508
N9	N	0.126189885208	0.024201017104	0.739948156215
N10	N	0.873810114792	0.524201017104	0.260051843785
N11	N	0.182397189073	0.154968103685	0.721050451058
N12	N	0.817602810928	0.654968103685	0.278949548942
N13	N	0.159812706914	0.353405683486	0.729249725470
N14	N	0.840187293086	0.853405683486	0.270750274529
N15	N	0.103260831005	0.424174977746	0.671896161995
N16	N	0.896739168995	0.924174977746	0.328103838005
N17	N	0.058630137097	0.293043013356	0.641376489430
N18	N	0.941369862903	0.793043013356	0.358623510570
N19	N	0.967087233686	0.265804352289	0.690200656741

N20	N	0.032912766314	0.765804352289	0.309799343258
N21	N	0.863973431339	0.230384579330	0.803127699337
N22	N	0.136026568661	0.730384579330	0.196872300663
N23	N	0.779595908864	0.151714644602	0.810209675189
N24	N	0.220404091136	0.651714644602	0.189790324811
N25	N	0.875637547278	0.118719852079	0.831566888636
N26	N	0.124362452722	0.618719852079	0.168433111364
N27	N	0.900149742767	0.400684277218	0.675400492922
N28	N	0.099850257233	0.900684277218	0.324599507078
N29	N	0.856984980832	0.371939357721	0.609911491191
N30	N	0.143015019168	0.871939357721	0.390088508809
N31	N	0.972421662924	0.315911962980	0.530484867401
N32	N	0.027578337076	0.815911962980	0.469515132599
N33	N	0.791734947030	0.242926714388	0.623812884578
N34	N	0.208265052971	0.742926714388	0.376187115422
N35	N	0.698230124094	0.300106904891	0.668288929853
N36	N	0.301769875906	0.800106904891	0.331711070147
N37	N	0.727923386192	0.427380901862	0.751195931861
N38	N	0.272076613809	0.927380901862	0.248804068139
N39	N	0.632129265336	0.386072961952	0.622124493481
N40	N	0.367870734664	0.886072961952	0.377875506519
N41	N	0.596989025532	0.269676241845	0.575819886357
N42	N	0.403010974468	0.769676241845	0.424180113643
N43	N	0.517491162854	0.198156599079	0.620961351580
N44	N	0.482508837146	0.698156599079	0.379038648419
N45	N	0.461732151455	0.051265923514	0.724906282175
N46	N	0.538267848545	0.551265923514	0.275093717824
N47	N	0.387279774285	0.963580014093	0.709164489146
N48	N	0.612720225715	0.463580014093	0.290835510854
N49	N	0.393891737418	0.002921886090	0.764097084205
N50	N	0.606108262583	0.502921886090	0.235902915795
N51	N	0.420937640423	0.303338422265	0.625226157217
N52	N	0.579062359577	0.803338422265	0.374773842783
N53	N	0.314320324024	0.281488178982	0.580902291397
N54	N	0.685679675976	0.781488178982	0.419097708603
N55	N	0.275351681189	0.154087080217	0.613880974187
N56	N	0.724648318811	0.654087080217	0.386119025813
N57	N	0.333209699717	0.021681300940	0.599542182959
N58	N	0.666790300283	0.521681300940	0.400457817040
N59	N	0.385326212530	0.841970035085	0.622441506251
N60	N	0.614673787470	0.341970035085	0.377558493749
N61	N	0.493342453349	0.826957752231	0.690029625175
N62	N	0.506657546652	0.326957752231	0.309970374825
N63	N	0.575232308047	0.706067614724	0.679464200450
N64	N	0.424767691954	0.206067614724	0.320535799550
N65	N	0.588747859938	0.696614282888	0.608450566003
N66	N	0.411252140062	0.196614282888	0.391549433997
N67	N	0.632858304425	0.843263537594	0.598657478118
N68	N	0.367141695575	0.343263537594	0.401342521882
N69	N	0.745108064053	0.824222101365	0.629992192832
N70	N	0.254891935948	0.324222101365	0.370007807168
N71	N	0.808886337642	0.712677693829	0.588073412062
N72	N	0.191113662359	0.212677693829	0.411926587937
N73	N	0.814045991815	0.809370172822	0.533096482621
N74	N	0.185954008185	0.309370172822	0.466903517379
N75	N	0.882878519508	0.939083037877	0.554508885505
N76	N	0.117121480492	0.439083037877	0.445491114495
N77	N	0.984329159071	0.894620615764	0.593220882368
N78	N	0.015670840929	0.394620615764	0.406779117632
N79	N	0.969447675726	0.904964504757	0.662903961424
N80	N	0.030552324274	0.404964504757	0.337096038576
N81	N	0.909229938955	0.804595955981	0.736938431092
N82	N	0.090770061045	0.304595955981	0.263061568907

N83	N	0.811768086412	0.668620378355	0.777317785577
N84	N	0.188231913588	0.168620378355	0.222682214423
N85	N	0.801357583170	0.615142160778	0.862129338035
N86	N	0.198642416830	0.115142160778	0.137870661965
N87	N	0.754251027016	0.486942745901	0.927291931428
N88	N	0.245748972985	0.986942745901	0.072708068572
N89	N	0.781586811807	0.531458112630	0.992525232086
N90	N	0.218413188193	0.031458112630	0.007474767914
N91	N	0.819562372994	0.707522965579	0.035207243753
N92	N	0.180437627006	0.207522965579	0.964792756247
N93	N	0.929443008830	0.776209678817	0.036463094456
N94	N	0.070556991170	0.276209678817	0.963536905544
N95	N	0.032544357228	0.840249239309	0.989361791568
N96	N	0.967455642772	0.340249239309	0.010638208432
N97	N	0.163466603854	0.788302967861	0.939769093117
N98	N	0.836533396146	0.288302967861	0.060230906883
N99	N	0.321082854883	0.861592011329	0.938410265102
N100	N	0.678917145117	0.361592011329	0.061589734898
N101	N	0.356608711443	0.882749293929	0.871150900674
N102	N	0.643391288557	0.382749293929	0.128849099326
N103	N	0.246603205770	0.907526811050	0.839894512201
N104	N	0.753396794230	0.407526811050	0.160105487799
N105	N	0.202137060563	0.049203027521	0.858510012451
N106	N	0.797862939437	0.549203027521	0.141489987549
N107	N	0.129774659126	0.171448858718	0.846961924666
N108	N	0.870225340874	0.671448858718	0.153038075334
N109	N	0.208544045251	0.329360849467	0.833487264397
N110	N	0.791455954749	0.829360849467	0.166512735603
O1	O	0.747949308550	0.757735045431	0.869506857597
O2	O	0.252050691450	0.257735045431	0.130493142403
O3	O	0.691615746532	0.795629061569	0.972300495803
O4	O	0.308384253468	0.295629061569	0.027699504197
O5	O	0.826390069803	0.989607342012	0.870950945112
O6	O	0.173609930198	0.489607342012	0.129049054888
O7	O	0.699039299891	0.993749691967	0.819199957305
O8	O	0.300960700109	0.493749691967	0.180800042695
O9	O	0.955607982911	0.917162376759	0.779720105512
O10	O	0.044392017089	0.417162376759	0.220279894488
O11	O	0.122920510577	0.075029140035	0.791977674852
O12	O	0.877079489423	0.575029140035	0.208022325148
O13	O	0.277197174639	0.112437986904	0.736091906720
O14	O	0.722802825361	0.612437986904	0.263908093280
O15	O	0.091761559832	0.257650599089	0.721232082033
O16	O	0.908238440168	0.757650599089	0.278767917966
O17	O	0.184547555882	0.248066292969	0.657319899247
O18	O	0.815452444118	0.748066292969	0.342680100753
O19	O	0.017864556332	0.426907074735	0.704652970979
O20	O	0.982135443668	0.926907074735	0.295347029021
O21	O	0.984747027214	0.362886907620	0.616748798828
O22	O	0.015252972786	0.862886907620	0.383251201172
O23	O	0.911553394608	0.215718483540	0.647496453324
O24	O	0.088446605392	0.715718483540	0.352503546675
O25	O	0.815604855777	0.336972763467	0.692825096002
O26	O	0.184395144223	0.836972763467	0.307174903998
O27	O	0.769188323997	0.430328373041	0.626411894524
O28	O	0.230811676003	0.930328373041	0.373588105476
O29	O	0.935345532026	0.556441944035	0.667630014501
O30	O	0.064654467974	0.056441944035	0.332369985499
O31	O	0.728416822200	0.272344302443	0.580318955742
O32	O	0.271583177800	0.772344302443	0.419681044258
O33	O	0.910259697814	0.414780488802	0.543027734231
O34	O	0.089740302186	0.914780488802	0.456972265769
O35	O	0.639381233874	0.207948115298	0.645725104708

O36	O	0.360618766126	0.707948115298	0.354274895292
O37	O	0.547295528908	0.369744811387	0.654421663951
O38	O	0.452704471092	0.869744811387	0.345578336049
O39	O	0.666357138597	0.324883767956	0.750979617150
O40	O	0.333642861403	0.824883767956	0.249020382850
O41	O	0.508090313730	0.335321359850	0.571481323594
O42	O	0.491909686270	0.835321359850	0.428518676406
O43	O	0.465505989384	0.151859700503	0.576341321172
O44	O	0.534494010617	0.651859700503	0.423658678828
O45	O	0.364972581277	0.222124989399	0.656022367844
O46	O	0.635027418723	0.722124989399	0.343977632156
O47	O	0.261273898606	0.341129822746	0.621546290259
O48	O	0.738726101394	0.841129822746	0.378453709741
O49	O	0.208829654530	0.124954033746	0.571895832921
O50	O	0.791170345470	0.624954033746	0.428104167078
O51	O	0.277328271635	0.957654093758	0.637882630482
O52	O	0.722671728365	0.457654093758	0.362117369518
O53	O	0.420805297837	0.953470534007	0.641159122064
O54	O	0.579194702163	0.453470534007	0.358840877936
O55	O	0.404303281533	0.038841714795	0.544564555828
O56	O	0.595696718467	0.538841714795	0.455435444172
O57	O	0.515829663403	0.827381838304	0.634073805464
O58	O	0.484170336597	0.327381838304	0.365926194536
O59	O	0.650690670858	0.793546560370	0.674437606295
O60	O	0.349309329142	0.293546560370	0.325562393705
O61	O	0.553443979761	0.846612729828	0.811052897974
O62	O	0.446556020239	0.346612729828	0.188947102026
O63	O	0.572997286946	0.969121880993	0.803981373028
O64	O	0.427002713054	0.469121880993	0.196018626972
O65	O	0.686415684346	0.657996732547	0.618725293800
O66	O	0.313584315654	0.157996732547	0.381274706200
O67	O	0.684702700416	0.799598890296	0.553318240386
O68	O	0.315297299584	0.299598890296	0.446681759613
O69	O	0.781664884242	0.927009493450	0.602663054236
O70	O	0.218335115758	0.427009493451	0.397336945764
O71	O	0.893760991702	0.776593519864	0.603918428794
O72	O	0.106239008298	0.276593519864	0.396081571206
O73	O	0.895395871145	0.743315411015	0.512618491902
O74	O	0.104604128855	0.243315411015	0.487381508098
O75	O	0.747363950347	0.640275276405	0.533623515596
O76	O	0.252636049653	0.140275276405	0.466376484404
O77	O	0.923320560735	0.933904019409	0.501766905380
O78	O	0.076679439265	0.433904019409	0.498233094620
O79	O	0.771375806498	0.147849139019	0.408644057027
O80	O	0.228624193502	0.647849139019	0.591355942973
O81	O	0.026833239518	0.011028993259	0.586950812045
O82	O	0.973166760482	0.511028993259	0.413049187955
O83	O	0.881592409012	0.003193186005	0.623636903828
O84	O	0.118407590988	0.503193186005	0.376363096172
O85	O	0.052941466837	0.830648125756	0.671358430378
O86	O	0.947058533163	0.330648125756	0.328641569622
O87	O	0.922009679025	0.771917060148	0.682311781172
O88	O	0.077990320975	0.271917060148	0.317688218827
O89	O	0.843486491322	0.783216671829	0.795601960804
O90	O	0.156513508678	0.283216671829	0.204398039196
O91	O	0.827506097798	0.539091614714	0.818438437103
O92	O	0.172493902203	0.039091614714	0.181561562897
O93	O	0.725455867229	0.606177482503	0.916648835468
O94	O	0.274544132771	0.106177482503	0.083351164532
O95	O	0.679924505474	0.528665329943	0.006684938657
O96	O	0.320075494527	0.028665329943	0.993315061343
O97	O	0.764311782990	0.682074358857	0.988075007040
O98	O	0.235688217010	0.182074358857	0.011924992960

O99	O	0.886267904321	0.887921698012	0.051218271804
O100	O	0.113732095679	0.387921698012	0.948781728196
O101	O	0.047922249915	0.717849234874	0.003153529726
O102	O	0.952077750085	0.217849234874	0.996846470274
O103	O	0.982337527320	0.781968751912	0.101575314672
O104	O	0.017662472680	0.281968751912	0.898424685328
O105	O	0.158367745065	0.865382711707	0.984724229790
O106	O	0.841632254935	0.365382711707	0.015275770210
O107	O	0.233491587352	0.902172252342	0.913062888100
O108	O	0.766508412648	0.402172252342	0.086937111900
O109	O	0.383317506449	0.003839101315	0.882695207192
O110	O	0.616682493551	0.503839101315	0.117304792808
O111	O	0.291168802909	0.916860328788	0.788460170351
O112	O	0.708831197091	0.416860328788	0.211539829649
O113	O	0.324158892286	0.737268749047	0.846703135437
O114	O	0.675841107714	0.237268749047	0.153296864563
O115	O	0.398072692041	0.691812493113	0.812591923216
O116	O	0.601927307959	0.191812493113	0.187408076784
O117	O	0.115790690340	0.982724613400	0.871991185552
O118	O	0.884209309661	0.482724613400	0.128008814448
O119	O	0.206858471162	0.689113538025	0.713308209895
O120	O	0.793141528838	0.189113538025	0.286691790104
O121	O	0.135670636194	0.210315306232	0.901010298590
O122	O	0.864329363806	0.710315306232	0.098989701410
O123	O	0.005791804658	0.150562602653	0.841960086277
O124	O	0.994208195342	0.650562602653	0.158039913723
O125	O	0.229849273812	0.242284810464	0.793830001769
O126	O	0.770150726188	0.742284810464	0.206169998231
O127	O	0.991554330988	0.259972708917	0.815613472096
O128	O	0.008445669012	0.759972708917	0.184386527904
O129	O	0.703260959321	0.027536933647	0.890128618373
O130	O	0.296739040679	0.527536933647	0.109871381627
O131	O	0.283816578692	0.100194092812	0.808496851019
O132	O	0.716183421308	0.600194092812	0.191503148981
O133	O	0.456590929383	0.040218166605	0.827057976451
O134	O	0.543409070618	0.540218166605	0.172942023549
O135	O	0.290477680124	0.390675059370	0.726363649190
O136	O	0.709522319876	0.890675059370	0.273636350810
O137	O	0.236676525789	0.444035046420	0.669369284041
O138	O	0.763323474211	0.944035046420	0.330630715958
O139	O	0.253349424860	0.592611877085	0.652201866166
O140	O	0.746650575140	0.092611877085	0.347798133834
O141	O	0.330010477372	0.645322765857	0.703582827930
O142	O	0.669989522628	0.145322765857	0.296417172070
O143	O	0.348686340509	0.518534641481	0.744853018091
O144	O	0.651313659491	0.018534641480	0.255146981909
O145	O	0.695338299289	0.270462564532	0.813116193625
O146	O	0.304661700711	0.770462564532	0.186883806375
O147	O	0.907865044350	0.515399496610	0.734995895897
O148	O	0.092134955650	0.015399496610	0.265004104103
O149	O	0.791095040513	0.556462456814	0.726013457513
O150	O	0.208904959487	0.056462456814	0.273986542487
O151	O	0.738858537928	0.552578597911	0.662215196419
O152	O	0.261141462072	0.052578597911	0.337784803581
O153	O	0.001579392275	0.164067616435	0.562696208554
O154	O	0.998420607725	0.664067616435	0.437303791446
O155	O	0.548461306581	0.259286291140	0.747334645991
O156	O	0.451538693419	0.759286291140	0.252665354008
O157	O	0.480773180922	0.368448298047	0.714524060959
O158	O	0.519226819078	0.868448298047	0.285475939041
O159	O	0.476093098526	0.500178275708	0.743557536504
O160	O	0.523906901474	0.000178275708	0.256442463496
O161	O	0.351625832426	0.251819332517	0.726697098051

O162	O	0.648374167574	0.751819332517	0.273302901949
O163	O	0.114771502402	0.234669312571	0.556316137878
O164	O	0.885228497598	0.734669312571	0.443683862121
O165	O	0.145339446240	0.006058964927	0.608676406954
O166	O	0.854660553760	0.506058964927	0.391323593046
O167	O	0.252495522202	0.822931900880	0.668408369457
O168	O	0.747504477798	0.322931900880	0.331591630543
O169	O	0.283124016619	0.045526530146	0.518584735969
O170	O	0.716875983382	0.545526530146	0.481415264031
O171	O	0.416878476340	0.778598297389	0.739001449039
O172	O	0.583121523660	0.278598297389	0.260998550961
O173	O	0.308742969923	0.840828110699	0.726532570091
O174	O	0.691257030077	0.340828110699	0.273467429909
O175	O	0.487653693494	0.652916406510	0.731745383609
O176	O	0.512346306506	0.152916406510	0.268254616391
O177	O	0.531638493109	0.111675529848	0.780790600493
O178	O	0.468361506892	0.611675529848	0.219209399507
O179	O	0.518977268636	0.697816626045	0.798358636854
O180	O	0.481022731364	0.197816626045	0.201641363146
O181	O	0.577950125303	0.750217244394	0.861032461759
O182	O	0.422049874697	0.250217244394	0.138967538240
O183	O	0.148264785001	0.737017726123	0.656187523249
O184	O	0.851735214999	0.237017726123	0.343812476751
O185	O	0.982691194311	0.683476624491	0.637352721279
O186	O	0.017308805689	0.183476624491	0.362647278721
O187	O	0.951641615857	0.502950286173	0.800923510734
O188	O	0.048358384143	0.002950286172	0.199076489266
O189	O	0.749431199643	0.419563740046	0.826860369419
O190	O	0.250568800357	0.919563740046	0.173139630581
O191	O	0.889483030158	0.458116299867	0.966302045034
O192	O	0.110516969842	0.958116299867	0.033697954966
O193	O	0.008539828740	0.484336570351	0.989395943013
O194	O	0.991460171260	0.984336570351	0.010604056987
O195	O	0.974679750373	0.603014618757	0.027982264702
O196	O	0.025320249627	0.103014618757	0.972017735298
O197	O	0.963733384301	0.583315287214	0.097999546455
O198	O	0.036266615699	0.083315287214	0.902000453545
O199	O	0.093411308969	0.594636021531	0.094017152891
O200	O	0.906588691031	0.094636021531	0.905982847109
O201	O	0.362318476067	0.734006251411	0.977689994587
O202	O	0.637681523933	0.234006251411	0.022310005413
O203	O	0.293681177734	0.584559129493	0.863272290993
O204	O	0.706318822267	0.084559129493	0.136727709007
O205	O	0.373215459773	0.544154432226	0.809107333008
O206	O	0.626784540227	0.044154432226	0.190892666992
O207	O	0.003178187284	0.392372712927	0.847486306728
O208	O	0.996821812716	0.892372712927	0.152513693272
O209	O	0.119881498450	0.413494941220	0.874343768312
O210	O	0.880118501550	0.913494941220	0.125656231687
O211	O	0.157695865316	0.561424818573	0.862435827686
O212	O	0.842304134684	0.061424818573	0.137564172314
S1	S	0.952181850748	0.838985874846	0.891813406985
S2	S	0.047818149252	0.338985874846	0.108186593015
S3	S	0.914169786136	0.103229285537	0.743860004386
S4	S	0.085830213864	0.603229285537	0.256139995614
S5	S	0.595538235707	0.043159501991	0.558967282485
S6	S	0.404461764293	0.543159501991	0.441032717515
S7	S	0.570805918082	0.003714643744	0.606340271636
S8	S	0.429194081918	0.503714643744	0.393659728364
S9	S	0.914270186479	0.055407536839	0.696177312427
S10	S	0.085729813521	0.555407536839	0.303822687572
S11	S	0.965643948017	0.920243219291	0.928417832773
S12	S	0.034356051983	0.420243219291	0.071582167227

#END

3) CRAMBIN CRYSTAL – optimized 172W

```
data_CSD_CIF_MOLDRAW
_audit_creation_date 09-04-2015
_audit_creation_method MOLDRAW
_chemical_formula_sum 'H974 C404 N110 O300 S12'
_chemical_formula_moiety
;
H974 C404 N110 O300 S12
;
loop_
_publ_author_name
Delle Piane, Corno, Orlando, Dovesi, Ugliengo
_cell_volume 15908.3864
_exptl_crystal_density_diffrn ?
_diffrn_ambient_temperature ?
_refine_ls_R_factor_gt ?
_refine_ls_wR_factor_gt ?
_symmetry_space_group_name_H-M 'P 1'
_symmetry_cell_setting triclinic
_symmetry_Int_Tables_number 1
loop_
_symmetry_equiv_pos_site_id
_symmetry_equiv_pos_as_xyz
1 x,y,z
_cell_length_a  22.1157478
_cell_length_b  18.1060023
_cell_length_c  39.7321474
_cell_angle_alpha  89.996365
_cell_angle_beta  90.778119
_cell_angle_gamma  90.028828
_cell_formula_units_Z 1
loop_
_atom_type_symbol
_atom_type_radius_bond
H  .38
C  .77
N  .75
O  .73
S  1.04
loop_
_atom_site_label
_atom_site_type_symbol
_atom_site_fract_x
_atom_site_fract_y
_atom_site_fract_z
H1   H     0.716064093014   0.718153111402   0.951247909248
H2   H     0.296776637555   0.199642733966   0.121218279019
H3   H     0.659033013041   0.755215927606   0.929041718822
H4   H     0.360292472921   0.253259068248   0.129753571479
H5   H     0.659391833414   0.766290797433   0.971352084626
H6   H     0.342779470756   0.222752724666   0.090983481752
H7   H     0.686781700934   0.878767777848   0.949211575791
H8   H     0.321015907196   0.357197143818   0.106394779055
H9   H     0.798109294844   0.791267841393   0.970897935933
```

H10	H	0.214024321575	0.261944573275	0.083609886588
H11	H	0.742079897837	0.789638210160	0.012696594763
H12	H	0.263311200533	0.248924821088	0.040443710400
H13	H	0.844681375765	0.907600053221	0.988854999726
H14	H	0.177848029711	0.375197431686	0.053956142709
H15	H	0.783493083393	0.959709549217	0.971914615376
H16	H	0.239635046979	0.426618961129	0.070903999571
H17	H	0.831500614944	0.909897170038	0.944638171158
H18	H	0.182147333775	0.392671693883	0.097914863851
H19	H	0.720994045434	0.944765154041	0.905689413899
H20	H	0.285188237789	0.428243828475	0.149367260257
H21	H	0.776008115361	0.858998827967	0.849980573661
H22	H	0.209819747741	0.353608432460	0.199848497174
H23	H	0.752553418069	0.962929528078	0.813259924653
H24	H	0.199883633677	0.502529585851	0.214474755632
H25	H	0.682217201828	0.039381819109	0.860941296198
H26	H	0.324108929728	0.500696047544	0.202321636472
H27	H	0.673880464127	0.869174500859	0.816596678579
H28	H	0.220944812925	0.404071102662	0.254505788366
H29	H	0.636510230630	0.956415169313	0.817086614316
H30	H	0.268916760705	0.482011690371	0.261511830431
H31	H	0.646171966315	0.905391532684	0.854830425561
H32	H	0.298040346782	0.400522364670	0.242801161377
H33	H	0.867524445754	0.878220295456	0.836045414794
H34	H	0.119210709415	0.362331054203	0.206167165439
H35	H	0.937082678304	0.995459799275	0.873444463463
H36	H	0.046443023476	0.474291445355	0.167945071872
H37	H	0.975157925284	0.835129183161	0.859087643210
H38	H	0.004342463434	0.318264571706	0.187604115964
H39	H	0.019847440687	0.903272145843	0.880148898051
H40	H	0.965291457250	0.384349421916	0.162260428832
H41	H	0.995517692432	0.065668556491	0.837273699409
H42	H	0.001589710149	0.551681618409	0.206639547248
H43	H	0.000866708217	0.999592179073	0.769241422453
H44	H	0.999056841988	0.488190471288	0.275290222919
H45	H	0.987266326789	0.160563075781	0.791249578317
H46	H	0.011180295239	0.648558004356	0.251959969426
H47	H	0.005440689246	0.136501316383	0.749556399669
H48	H	0.997284142109	0.625305978603	0.294166262991
H49	H	0.197843494279	0.005805788381	0.780693644290
H50	H	0.801658790431	0.496445520429	0.269756407269
H51	H	0.234565208797	0.983487351265	0.716007204795
H52	H	0.771013742633	0.476302499782	0.335707071060
H53	H	0.191894231073	0.916018639732	0.737301692676
H54	H	0.811009986605	0.408244864710	0.313258402060
H55	H	0.139929195226	0.034008753081	0.689517017530
H56	H	0.867882867352	0.524700027166	0.360134764294
H57	H	0.130930884900	0.937754825985	0.685875975631
H58	H	0.877095542092	0.428528614038	0.362125010906
H59	H	0.045719041872	0.020632478688	0.718069482457
H60	H	0.958777040875	0.513886739929	0.328064000478
H61	H	0.067142235963	0.933052160708	0.735056066134
H62	H	0.936727058939	0.425861937192	0.311858439169
H63	H	0.120679204923	0.142334529034	0.736887908919
H64	H	0.882195207249	0.633942673079	0.311344712448
H65	H	0.217704056322	0.243389176056	0.753480898872
H66	H	0.785954489604	0.733612578253	0.293926972593
H67	H	0.232547331951	0.296972338092	0.700215790064
H68	H	0.771428162518	0.794631050305	0.345265679978
H69	H	0.243044996338	0.201247797352	0.695122848564
H70	H	0.758698267752	0.700280161962	0.352619112582
H71	H	0.180978965816	0.267381264549	0.655246537956
H72	H	0.821185193133	0.768635433140	0.391599119710

H73	H	0.187109746272	0.366218731155	0.747252556614
H74	H	0.817036216942	0.855276294581	0.297880577662
H75	H	0.062576296472	0.386224598885	0.768235011218
H76	H	0.938684940036	0.879405058760	0.274569671098
H77	H	0.166728049983	0.488750685799	0.750897911720
H78	H	0.839765876817	0.980005035362	0.301752028891
H79	H	0.096686100973	0.451586652653	0.814786357097
H80	H	0.881947721241	0.946581397659	0.230158250293
H81	H	0.168932150082	0.417798260231	0.804431842542
H82	H	0.815819002300	0.912156082690	0.248920505166
H83	H	0.073951909819	0.555185936254	0.731976128930
H84	H	0.938173276025	0.042338234717	0.310744832617
H85	H	0.105115399470	0.594695006770	0.768777454517
H86	H	0.895109591906	0.084914614299	0.278479675342
H87	H	0.039405483929	0.537812143648	0.771365430000
H88	H	0.957624898857	0.027813845236	0.268001947409
H89	H	0.212719084269	0.544937408293	0.809145128572
H90	H	0.780583567295	0.047217610078	0.255809546894
H91	H	0.178425771932	0.515818431892	0.847011031218
H92	H	0.782815889280	0.013086660908	0.214185659638
H93	H	0.141216119020	0.580466433348	0.820639016648
H94	H	0.840827838748	0.072619265646	0.228666427311
H95	H	0.130902736964	0.420416406572	0.686802540599
H96	H	0.875892470176	0.913599038201	0.357421935059
H97	H	0.011734751532	0.468037336969	0.662359135822
H98	H	0.995813525321	0.959327692151	0.380432140780
H99	H	0.127392444277	0.422184841877	0.628255014866
H100	H	0.878431488770	0.922455084385	0.414732411259
H101	H	0.047625463566	0.364970278576	0.591784433808
H102	H	0.952663305742	0.869531567858	0.454673430574
H103	H	0.080335769099	0.442618769712	0.572068518467
H104	H	0.923293892412	0.953246895703	0.470351146040
H105	H	0.007081521583	0.448891240849	0.590183608520
H106	H	0.997592494122	0.951202886359	0.453317661753
H107	H	0.116116100022	0.553098587360	0.605625799416
H108	H	0.890822808542	0.056708793152	0.432435082022
H109	H	0.118067426553	0.550702516544	0.650324784369
H110	H	0.892534920232	0.046736031789	0.387964106135
H111	H	0.048018379846	0.562215822468	0.628466019404
H112	H	0.960842543588	0.059705670519	0.411235734367
H113	H	0.076552985998	0.286391590287	0.671106830481
H114	H	0.929286121247	0.778904858627	0.374908413953
H115	H	0.977496449189	0.217400393230	0.634660477191
H116	H	0.028588726682	0.711520883966	0.411843708756
H117	H	0.068119725613	0.146072242480	0.650135554286
H118	H	0.936414437829	0.638930719804	0.395417389926
H119	H	0.003007745089	0.105635772579	0.668092797848
H120	H	0.002433087337	0.598114146665	0.378778039695
H121	H	0.054184967276	0.158834658162	0.693761097000
H122	H	0.953287146347	0.651323261430	0.352136941004
H123	H	0.990733418512	0.281182880200	0.720186663246
H124	H	0.016057814407	0.770243953996	0.325140776401
H125	H	0.870191436573	0.238778741324	0.739324192092
H126	H	0.138735112473	0.726666757718	0.308940312368
H127	H	0.947596844138	0.278727239215	0.780970634396
H128	H	0.065380409063	0.759706894010	0.264046378937
H129	H	0.954025459210	0.365277724760	0.761133239339
H130	H	0.055556205091	0.848372453574	0.281434886405
H131	H	0.896602943411	0.383126445668	0.810996952023
H132	H	0.117856784271	0.864355518911	0.233849944877
H133	H	0.848868369190	0.399044842457	0.776468058831
H134	H	0.163357597699	0.881218812565	0.269203181879
H135	H	0.793461091096	0.334327470159	0.818416383925

H136	H	0.223684794381	0.815826625589	0.230745519996
H137	H	0.801132635813	0.273659843229	0.783907981658
H138	H	0.208651531038	0.751541032541	0.262910303103
H139	H	0.900206476507	0.255666027539	0.832613333555
H140	H	0.115134760534	0.738952703929	0.211898733518
H141	H	0.742006604689	0.212791475944	0.829770364305
H142	H	0.276413862680	0.710030879130	0.209690773428
H143	H	0.755072330454	0.118248171908	0.835098342551
H144	H	0.267092777462	0.616025117088	0.200071268877
H145	H	0.910315411223	0.144811892571	0.855100571738
H146	H	0.107309681896	0.629195404620	0.187358692859
H147	H	0.847208602309	0.092745534565	0.864431562333
H148	H	0.172185054148	0.587314301821	0.172090318944
H149	H	0.923751073765	0.410237649518	0.702640609792
H150	H	0.079178664072	0.898376600447	0.341966773424
H151	H	0.806173626750	0.474930267195	0.701090043643
H152	H	0.192933074555	0.969316492475	0.343284281513
H153	H	0.903964335016	0.492749079059	0.647687111766
H154	H	0.097523066853	0.982916128183	0.398338757829
H155	H	0.840904060360	0.552232516384	0.655179982957
H156	H	0.155529095525	0.047532060143	0.387806706527
H157	H	0.927979922568	0.596325285803	0.680976513214
H158	H	0.067534815712	0.083645693681	0.364367315886
H159	H	0.876729335010	0.357232591298	0.640368271685
H160	H	0.130874233716	0.851705357289	0.408012024205
H161	H	0.774866250242	0.356407422261	0.594615516049
H162	H	0.245855115732	0.864727358945	0.445673034966
H163	H	0.873805317350	0.240626358492	0.607668258318
H164	H	0.135774880955	0.766444173320	0.456831480086
H165	H	0.819414232697	0.237284316201	0.574634826385
H166	H	0.205137363335	0.760195782695	0.480191003966
H167	H	0.944630772786	0.240576711945	0.564592994439
H168	H	0.071101905827	0.831149945649	0.486895318681
H169	H	0.956224550418	0.316435653447	0.537469829818
H170	H	0.086012331583	0.912798124512	0.509017513924
H171	H	0.811658481900	0.238137777927	0.664985292092
H172	H	0.195123463547	0.740647509949	0.381564819375
H173	H	0.712583692696	0.139923916176	0.656841326675
H174	H	0.293039284367	0.639359394648	0.391624744701
H175	H	0.802049605268	0.130321833842	0.697285945902
H176	H	0.203800820137	0.624012566391	0.351466180756
H177	H	0.768988629651	0.198962269120	0.722600335868
H178	H	0.235602376933	0.691238475515	0.324943522491
H179	H	0.672925758034	0.168886323802	0.754595809227
H180	H	0.331859923655	0.662375587558	0.293630268937
H181	H	0.760956391763	0.007362050797	0.692569319510
H182	H	0.247435367814	0.501332397528	0.357505958128
H183	H	0.610498439414	0.070551878362	0.779817517646
H184	H	0.395712742101	0.564210961692	0.269449146252
H185	H	0.697587899979	0.907677846018	0.717962634814
H186	H	0.314272442808	0.403192411879	0.333930713873
H187	H	0.622588308866	0.939812436174	0.761259042883
H188	H	0.388718893379	0.435655224328	0.290051964342
H189	H	0.718171013611	0.312060496721	0.702954798217
H190	H	0.286382940556	0.807023929044	0.342974785853
H191	H	0.593353291904	0.294434899178	0.722454970107
H192	H	0.411757028960	0.797055473295	0.323782546160
H193	H	0.666414974658	0.438854313489	0.716820965385
H194	H	0.327532682388	0.931387066620	0.329452605318
H195	H	0.594801601436	0.427254939376	0.735641575761
H196	H	0.401956268856	0.934038348995	0.313772393102
H197	H	0.718123830554	0.475822339217	0.764721920235
H198	H	0.293853761767	0.974157359096	0.277475808172

H199	H	0.717457432925	0.422647860237	0.802115484290
H200	H	0.303939597045	0.923946385466	0.239583918249
H201	H	0.657407167391	0.375613941337	0.648956643850
H202	H	0.342991019402	0.878294917283	0.397117444917
H203	H	0.540010261644	0.429272262515	0.624574748521
H204	H	0.458455902265	0.932250893796	0.422972000161
H205	H	0.660172018080	0.394415915034	0.591442321770
H206	H	0.339743295591	0.890111552001	0.456247033749
H207	H	0.617562639046	0.454305002725	0.539010900019
H208	H	0.381014530527	0.951584015995	0.508577294163
H209	H	0.544141679343	0.451149000679	0.556509281281
H210	H	0.454242012869	0.951570959737	0.490897671882
H211	H	0.583211142343	0.367958847968	0.547835013114
H212	H	0.417447006203	0.866699092821	0.499432811025
H213	H	0.664169013252	0.532746165313	0.585157078046
H214	H	0.331018567764	0.027570859817	0.463852436457
H215	H	0.655352420308	0.507779222619	0.627624413839
H216	H	0.338928896617	0.005554226039	0.420993074778
H217	H	0.591937091267	0.539765422947	0.604034911918
H218	H	0.402083692654	0.039861619525	0.444250237995
H219	H	0.622022243134	0.260209183408	0.611017985991
H220	H	0.380871470533	0.759357257969	0.436259424885
H221	H	0.534233639294	0.193579122532	0.567175337314
H222	H	0.472803422731	0.697999228117	0.479501557439
H223	H	0.637489632457	0.146051414384	0.577432338081
H224	H	0.370025466706	0.646935906913	0.472623560265
H225	H	0.622471203809	0.131887182144	0.620699543650
H226	H	0.381193792629	0.631311039119	0.429111082457
H227	H	0.536165905695	0.205752686787	0.656672903498
H228	H	0.464836072669	0.708637110250	0.390108323511
H229	H	0.427788302201	0.115625283134	0.662066749078
H230	H	0.575525361779	0.623479902612	0.383621155949
H231	H	0.490779220971	0.223437360545	0.710813465606
H232	H	0.501977025523	0.720952230559	0.335230489897
H233	H	0.429537350466	0.167558753279	0.721998299429
H234	H	0.567912812235	0.674164042909	0.323032743837
H235	H	0.548236062837	0.102960605071	0.694868091075
H236	H	0.458158698571	0.595173042861	0.353884484468
H237	H	0.536773931306	0.122089520862	0.737441687678
H238	H	0.465056984181	0.610830150284	0.310827430410
H239	H	0.453589767140	0.019224240878	0.695879566463
H240	H	0.558113136773	0.524041845195	0.353460124267
H241	H	0.517605664901	0.988537400682	0.718717612501
H242	H	0.497561006933	0.483691751400	0.330825296636
H243	H	0.468094669836	0.050484816053	0.769965027622
H244	H	0.544576999995	0.552856096337	0.279469386564
H245	H	0.382315353942	0.949798697969	0.706897338250
H246	H	0.623685412873	0.445937517796	0.342823355170
H247	H	0.331993690529	0.920497610321	0.737823300914
H248	H	0.674280892775	0.414532222901	0.312383146028
H249	H	0.402104490108	0.020486031416	0.803958202836
H250	H	0.611655168690	0.515195258682	0.245273407147
H251	H	0.338615610338	0.964311109612	0.792959699311
H252	H	0.669781481683	0.454617138362	0.256933230088
H253	H	0.440885450419	0.299613528174	0.636859434152
H254	H	0.557896330513	0.806700795780	0.407234629245
H255	H	0.334991102685	0.352451695090	0.670143337259
H256	H	0.662574586382	0.863172866284	0.374972992227
H257	H	0.393590524073	0.397729627218	0.603818662758
H258	H	0.598209299518	0.899782187279	0.440416297864
H259	H	0.330219901558	0.442678068482	0.620937229067
H260	H	0.666451300273	0.944651153401	0.430173411924
H261	H	0.454846898015	0.431614675098	0.654822119771

H262	H	0.553185016129	0.945659372093	0.385231496389
H263	H	0.451888970942	0.509799459137	0.603200698283
H264	H	0.535896092688	0.007331861180	0.440207836555
H265	H	0.460971242237	0.566190304741	0.639495661702
H266	H	0.539875189606	0.074653994564	0.407737174952
H267	H	0.389463120155	0.560143578638	0.618887229450
H268	H	0.602368828472	0.061044752105	0.435492969698
H269	H	0.413455275954	0.526450056161	0.695132037118
H270	H	0.607267500804	0.050966051325	0.356569360676
H271	H	0.377856695202	0.439827758351	0.699063049695
H272	H	0.647266750267	0.967573308490	0.352508695364
H273	H	0.340205280244	0.512932143732	0.677401193941
H274	H	0.671823049745	0.034272613928	0.382412908523
H275	H	0.204790195943	0.267911537552	0.587081028601
H276	H	0.793036767901	0.768877482199	0.455657132609
H277	H	0.236649897652	0.156834697373	0.542591460047
H278	H	0.761209366477	0.675776217414	0.506052868943
H279	H	0.246754241259	0.251273839844	0.533065714306
H280	H	0.748069447578	0.771248909587	0.509511543030
H281	H	0.341793713084	0.144500813112	0.560699589062
H282	H	0.655406528389	0.656276567821	0.486481850459
H283	H	0.348156442290	0.207692951211	0.526698100832
H284	H	0.647891411713	0.722979033512	0.518955911900
H285	H	0.391653872943	0.244279466020	0.589525486601
H286	H	0.605077709093	0.752996403746	0.455267859466
H287	H	0.355292345248	0.312383884205	0.564167278303
H288	H	0.641798709352	0.823530061019	0.478903612957
H289	H	0.292061178946	0.168734975489	0.640528207486
H290	H	0.709011344764	0.678725136921	0.401570746125
H291	H	0.251473698117	0.086843993290	0.678745981460
H292	H	0.737390268636	0.585857949561	0.367377468589
H293	H	0.192528304966	0.062226758893	0.649496935176
H294	H	0.804550541597	0.571798891204	0.392229084049
H295	H	0.322340337355	0.051444404868	0.606847254391
H296	H	0.667809346992	0.561895706653	0.434582648580
H297	H	0.324316577542	0.903753654298	0.594864425456
H298	H	0.680012018104	0.414606389699	0.457122113975
H299	H	0.436595145937	0.996557331311	0.610093921869
H300	H	0.563710357641	0.494455421694	0.437053910531
H301	H	0.413135287896	0.064150606924	0.570708268127
H302	H	0.581898732756	0.570933874898	0.473808465795
H303	H	0.462115758738	0.869082386259	0.597027821908
H304	H	0.542826409021	0.368258977715	0.452454476532
H305	H	0.479939476981	0.924056312087	0.561420874370
H306	H	0.521659558948	0.424055206824	0.487210153082
H307	H	0.413560473424	0.868408789905	0.560583396039
H308	H	0.590292748172	0.373114646646	0.489244010711
H309	H	0.365944284307	0.800514985405	0.696957939170
H310	H	0.644672919211	0.294836210776	0.357591291479
H311	H	0.427370458589	0.691171115078	0.649879214652
H312	H	0.583042424257	0.190063046574	0.406432379838
H313	H	0.385833491516	0.671807196708	0.687454295474
H314	H	0.626370897281	0.167138556497	0.370304366483
H315	H	0.326057742578	0.657756728623	0.627613299196
H316	H	0.682659664600	0.162002058981	0.431824576183
H317	H	0.291257302173	0.707454912579	0.660624918031
H318	H	0.719823763168	0.206948717053	0.398249184746
H319	H	0.353835723749	0.771316179984	0.599264393516
H320	H	0.652810805753	0.278767478260	0.456153874871
H321	H	0.286640625942	0.801390769218	0.619683179428
H322	H	0.720733012555	0.307479310269	0.436070055459
H323	H	0.441723718127	0.797001805341	0.733024527021
H324	H	0.570814250174	0.286985361237	0.321480385946

H325	H	0.553071594068	0.873989813506	0.718952274853
H326	H	0.462009022713	0.373046142568	0.334282208658
H327	H	0.528393987430	0.755142783245	0.772025089028
H328	H	0.484921100209	0.254981734952	0.280885480072
H329	H	0.593568423049	0.810157627753	0.770050689768
H330	H	0.424184914778	0.317438749260	0.280925771801
H331	H	0.526501805350	0.924975316132	0.778091012502
H332	H	0.495720034878	0.424087896708	0.273450852065
H333	H	0.464195391833	0.865459203215	0.782539981100
H334	H	0.557781175373	0.363540676473	0.276418355656
H335	H	0.520743288527	0.677161329696	0.716280520392
H336	H	0.486562955909	0.173716865703	0.336173969828
H337	H	0.640626376640	0.638973745360	0.702906258341
H338	H	0.361895230258	0.144844889362	0.345987634628
H339	H	0.560272774164	0.548283633230	0.716058940156
H340	H	0.438076990755	0.048755309238	0.332573175099
H341	H	0.598015139265	0.523060885263	0.678447029947
H342	H	0.395365085387	0.024484600126	0.368623917139
H343	H	0.524578964118	0.563423145074	0.676348541740
H344	H	0.471113940545	0.056954290738	0.373473008022
H345	H	0.532750261757	0.707456174454	0.643337224002
H346	H	0.469066910007	0.207222316747	0.406948443709
H347	H	0.613766484475	0.673573958083	0.586431771978
H348	H	0.384958377977	0.175275245773	0.462637178932
H349	H	0.493972801074	0.756647377171	0.593850633913
H350	H	0.505782696471	0.257825089596	0.457208199101
H351	H	0.535915661339	0.763081938006	0.556540980160
H352	H	0.462856966319	0.260319226310	0.494281477714
H353	H	0.495139951788	0.615540011853	0.586042587071
H354	H	0.504222720991	0.115672433314	0.462367407084
H355	H	0.403164304905	0.687297903810	0.573457484944
H356	H	0.596204779480	0.186830367526	0.475056890076
H357	H	0.410873304722	0.617518114971	0.542605354270
H358	H	0.589645479773	0.115432076214	0.505294386987
H359	H	0.429705459187	0.710662621253	0.532813802553
H360	H	0.570911335895	0.208003605877	0.516133197424
H361	H	0.514312767201	0.567213254670	0.528066211005
H362	H	0.486921018181	0.063519202152	0.519949797982
H363	H	0.580997524561	0.605119946434	0.545820860356
H364	H	0.419628736499	0.101137389391	0.502705560163
H365	H	0.539230981769	0.655760063712	0.515281676279
H366	H	0.461128167212	0.150797281360	0.533862622752
H367	H	0.577288348530	0.827335185885	0.639599890244
H368	H	0.428683268364	0.329465913974	0.411176402382
H369	H	0.658921010665	0.921283428206	0.601819240817
H370	H	0.347331740178	0.426567880317	0.447494238724
H371	H	0.610923965903	0.935108536540	0.673731377748
H372	H	0.397522253836	0.437382470340	0.375892578784
H373	H	0.655824924140	0.002451419502	0.653430967060
H374	H	0.353059897498	0.506031017842	0.395398168972
H375	H	0.686304650262	0.793400949424	0.668081223693
H376	H	0.322329721426	0.300701054770	0.380371169119
H377	H	0.811753726995	0.833160716350	0.677295473569
H378	H	0.199353400880	0.347121005678	0.367149896069
H379	H	0.760537990045	0.752441300362	0.718146853933
H380	H	0.256564354427	0.268050973396	0.327531274684
H381	H	0.820194335863	0.701814487352	0.699116800320
H382	H	0.189402987985	0.222438230415	0.341034032134
H383	H	0.743807829146	0.686043135541	0.686317623520
H384	H	0.260974552880	0.195863970048	0.357911509744
H385	H	0.745292473654	0.695367248893	0.619586888540
H386	H	0.256140672214	0.199788677162	0.423359522571
H387	H	0.867525572026	0.671805606834	0.593290713022

H388	H	0.132477900911	0.178834251632	0.445532147958
H389	H	0.817274995804	0.614585080757	0.544746250300
H390	H	0.176968936870	0.119199934292	0.495879164116
H391	H	0.718060487309	0.601585610117	0.544086496653
H392	H	0.277044843457	0.100424839897	0.496972914551
H393	H	0.847364958016	0.534537632761	0.592693510402
H394	H	0.146775989593	0.044657978270	0.448114353869
H395	H	0.780599143515	0.502504877601	0.573085473794
H396	H	0.214115515164	0.008014770689	0.465498369060
H397	H	0.775536398388	0.550308539955	0.612143296192
H398	H	0.217828032860	0.060099352570	0.427566610103
H399	H	0.749647420165	0.802193163501	0.572532626646
H400	H	0.251022122121	0.301244245531	0.474907552944
H401	H	0.812341763128	0.847404994321	0.513541362782
H402	H	0.184878461698	0.347194174815	0.532506552463
H403	H	0.703802961591	0.879259874763	0.527385825392
H404	H	0.294056650300	0.377599764352	0.520121641865
H405	H	0.729988996558	0.941871670157	0.558172015358
H406	H	0.270297576869	0.438087855485	0.487975867745
H407	H	0.757039243643	0.896327910992	0.466825709568
H408	H	0.248389820774	0.401144269477	0.580050350375
H409	H	0.746979180504	0.068771156131	0.540846306155
H410	H	0.247034028577	0.566306025786	0.502360653810
H411	H	0.780876415120	0.987533157085	0.423349136610
H412	H	0.230457958392	0.496894507273	0.622685126212
H413	H	0.772747393342	0.161248195780	0.496935273538
H414	H	0.228313695704	0.663144974389	0.545461727945
H415	H	0.789841702876	0.112264252279	0.411309532478
H416	H	0.219325300426	0.619626016634	0.632810879832
H417	H	0.828044597108	0.913264345718	0.596802854390
H418	H	0.182966736217	0.424377573757	0.449733506012
H419	H	0.910990027340	0.029764699779	0.572681520829
H420	H	0.097682632081	0.537994251369	0.471611274729
H421	H	0.900641241878	0.080480214453	0.630248017615
H422	H	0.097715587043	0.577447153869	0.412237836393
H423	H	0.821278340983	0.961994028273	0.645295758834
H424	H	0.181614663076	0.462131131697	0.399627141141
H425	H	0.821061753491	0.110740676671	0.586553439463
H426	H	0.177192879849	0.622611424784	0.452586451980
H427	H	0.795358561124	0.115671794842	0.628566789139
H428	H	0.201158371145	0.619640336976	0.410387227859
H429	H	0.774579182353	0.038786463665	0.602854383940
H430	H	0.226731876508	0.550625998281	0.439237575606
H431	H	0.925831607137	0.859688982753	0.609519913998
H432	H	0.083536574409	0.367154917214	0.430271688602
H433	H	0.014417009473	0.800780765740	0.627359437063
H434	H	0.987870462517	0.306361078940	0.420884074452
H435	H	0.054034321626	0.885988175470	0.628549031523
H436	H	0.951779539293	0.393936275815	0.418745693621
H437	H	0.925449131545	0.923616500988	0.667518892035
H438	H	0.076838001794	0.425454088626	0.377681186427
H439	H	0.976029670541	0.910094456290	0.735907512187
H440	H	0.025565416669	0.406241444110	0.309811779405
H441	H	0.845486127457	0.939090409398	0.711741468703
H442	H	0.157163318469	0.432497874880	0.332808885365
H443	H	0.874122294846	0.949837101708	0.753263412183
H444	H	0.128021416052	0.439162393478	0.291263119140
H445	H	0.918248780015	0.845376330925	0.777814204223
H446	H	0.071944673267	0.334032743831	0.269491658303
H447	H	0.874707818873	0.700785694411	0.751291383018
H448	H	0.117438647988	0.192029905694	0.297530526579
H449	H	0.936201021966	0.636477093691	0.792226643562
H450	H	0.052130831823	0.123516582463	0.261085683018

H451	H	0.992987656060	0.783645617053	0.812993188784
H452	H	0.996047723263	0.266927895614	0.234920349281
H453	H	0.939964850600	0.733641558846	0.836727657788
H454	H	0.049423749796	0.213822658798	0.212979642084
H455	H	0.038914204742	0.667024755100	0.766838381490
H456	H	0.950854968020	0.163667211998	0.286968859047
H457	H	0.005605286762	0.743039102299	0.746698123983
H458	H	0.987596606339	0.242426389344	0.302414182040
H459	H	0.979684852980	0.652382801849	0.737159910759
H460	H	0.011644146926	0.154384650972	0.316287125508
H461	H	0.012836357901	0.624975067664	0.838899882847
H462	H	0.977538453880	0.104888257113	0.212464476019
H463	H	0.041143281398	0.706194794082	0.859146069776
H464	H	0.946717111368	0.182888217450	0.191735070170
H465	H	0.068201187650	0.681326202649	0.819067845974
H466	H	0.922335520961	0.160409330263	0.232493871120
H467	H	0.821390281368	0.630637272400	0.788783840959
H468	H	0.164951339964	0.117371147563	0.258589993304
H469	H	0.767981155008	0.718731833892	0.844386599601
H470	H	0.217820158254	0.207052716297	0.203298524057
H471	H	0.711340677506	0.578699806257	0.814677831324
H472	H	0.283922637291	0.079839786645	0.238605494027
H473	H	0.662407580878	0.733481484897	0.801566589906
H474	H	0.293941556215	0.245809041782	0.251711558641
H475	H	0.709718243540	0.685715095863	0.773082090810
H476	H	0.266683894625	0.178037602073	0.278585577669
H477	H	0.683646027223	0.603858884443	0.874689008979
H478	H	0.314667553763	0.104738082494	0.178797531354
H479	H	0.620661118777	0.621020810981	0.848331826520
H480	H	0.373550956633	0.129209511960	0.206980555860
H481	H	0.664212582818	0.696078651186	0.864104114530
H482	H	0.327075648722	0.198512284667	0.188536890499
H483	H	0.636749150923	0.581270772581	0.769318195484
H484	H	0.366587850273	0.114108814364	0.281630497599
H485	H	0.601251228951	0.665289238141	0.757712593564
H486	H	0.369218605530	0.206712010587	0.296364754412
H487	H	0.586882275123	0.626470732645	0.797314616257
H488	H	0.399644468483	0.183570530097	0.256523901525
H489	H	0.789377222624	0.678093274777	0.895303751492
H490	H	0.195126431643	0.169602570495	0.154487376908
H491	H	0.831973868448	0.522780737592	0.899131243834
H492	H	0.167869680152	0.009983663666	0.146344694129
H493	H	0.894432600498	0.550339784281	0.948545428940
H494	H	0.099987094630	0.033357965345	0.100296254146
H495	H	0.868003217955	0.713847336758	0.935045598307
H496	H	0.121864766176	0.198897129322	0.110569204142
H497	H	0.838464721010	0.661781912671	0.968514775146
H498	H	0.147687153971	0.145384170044	0.076795517603
H499	H	0.979758416198	0.604046918721	0.916967702047
H500	H	0.013611431660	0.081951782650	0.132314654355
H501	H	0.928964848162	0.647725594501	0.888153306766
H502	H	0.062317977731	0.133644615987	0.159123828729
H503	H	0.935609403373	0.550312811765	0.889030166021
H504	H	0.062654943880	0.036357196285	0.161267014902
H505	H	0.943295302992	0.640648475044	0.992021496864
H506	H	0.038866211775	0.119754164255	0.058720013069
H507	H	0.925259145948	0.735733610061	0.988668992137
H508	H	0.057836115194	0.214844821938	0.058622839278
H509	H	0.973876138968	0.694305447433	0.959015831608
H510	H	0.013884442396	0.177791180398	0.091579471718
H511	H	0.678870451700	0.492989460197	0.970177411882
H512	H	0.318920320708	0.036715184061	0.073996386782
H513	H	0.731230278216	0.385944254885	0.013401271435

H514	H	0.312447253649	0.905964678706	0.037672554593
H515	H	0.688478391968	0.370068981669	0.975964389489
H516	H	0.336815194300	0.908514011487	0.080285876356
H517	H	0.825050557210	0.371185447788	0.986353409627
H518	H	0.210017158998	0.872821310848	0.052194130569
H519	H	0.782189166940	0.313545441204	0.959007334135
H520	H	0.251027430964	0.822357217696	0.083197841610
H521	H	0.845576238992	0.434536375344	0.936123213020
H522	H	0.164751177841	0.923721939015	0.101271345829
H523	H	0.775467018856	0.408980285274	0.916246994778
H524	H	0.232591995933	0.913234036259	0.126311085154
H525	H	0.827328092107	0.534496182985	0.003689120224
H526	H	0.168593038720	0.018781371914	0.043670126420
H527	H	0.781421749969	0.575266413741	0.068906831044
H528	H	0.194535025800	0.066026399470	0.975419360049
H529	H	0.854858436876	0.589956995495	0.051937054432
H530	H	0.126312043294	0.076273798198	0.998465297802
H531	H	0.835341361075	0.696242393763	0.088325755349
H532	H	0.156847445912	0.193649308674	0.959088292508
H533	H	0.776048628352	0.820282710132	0.052162782137
H534	H	0.206992770818	0.305623355808	0.006519041925
H535	H	0.743469323822	0.805796564051	0.111250008234
H536	H	0.264651024263	0.294413234120	0.954512257783
H537	H	0.785259846984	0.888642628732	0.105341414685
H538	H	0.224486013929	0.380244170693	0.954828189803
H539	H	0.820167171424	0.811218791100	0.125470244019
H540	H	0.198152609452	0.305570868469	0.929201124665
H541	H	0.913609337918	0.738415758551	0.064568450815
H542	H	0.077788586023	0.232520940610	0.984395909084
H543	H	0.977357983575	0.882323155793	0.068864068642
H544	H	0.014050398483	0.375091785893	0.974581269516
H545	H	0.018082250309	0.730366116336	0.094488644363
H546	H	0.986860516308	0.223394017586	0.944072901116
H547	H	0.939364906800	0.761995650023	0.124867194431
H548	H	0.064086289771	0.272108919939	0.918912514537
H549	H	0.102763078872	0.818388397862	0.085846406668
H550	H	0.891821140197	0.288733636931	0.950840981506
H551	H	0.090631255095	0.801371097744	0.129503121073
H552	H	0.913402262322	0.292389096420	0.908023538025
H553	H	0.065549847560	0.886030835981	0.110238195889
H554	H	0.923410551328	0.372090955047	0.934584349693
H555	H	0.998273140836	0.907073846764	0.017540472807
H556	H	0.992450919465	0.398962968472	0.025601274545
H557	H	0.026196373660	0.800690775626	0.966474701489
H558	H	0.957282635404	0.288056358247	0.073384802493
H559	H	0.054106259236	0.966426525175	0.973696503214
H560	H	0.954271395231	0.457753739033	0.072989765511
H561	H	0.067236838839	0.915666070840	0.936538400048
H562	H	0.933143515755	0.405313070654	0.108410513884
H563	H	0.221833950802	0.769141999018	0.991001405675
H564	H	0.760743427692	0.290329769682	0.047815436597
H565	H	0.261931375572	0.716130283072	0.930686816448
H566	H	0.716601422716	0.211905767019	0.101990698021
H567	H	0.211014158583	0.664202757107	0.955182781018
H568	H	0.761127241331	0.169605038485	0.070692210993
H569	H	0.179294189302	0.767698703471	0.896509592675
H570	H	0.804136594067	0.232272539195	0.138795932836
H571	H	0.157042864790	0.673341694234	0.901036834012
H572	H	0.820565029353	0.143135060186	0.122947232064
H573	H	0.081629395425	0.786277800362	0.920836802582
H574	H	0.901399339309	0.256160857293	0.115927403970
H575	H	0.086579603289	0.702757346918	0.944547187562
H576	H	0.891880891952	0.190058910614	0.083004546276

H577	H	0.312756492280	0.825203113100	0.980695324212
H578	H	0.671281152795	0.338747822366	0.067675788038
H579	H	0.306216763496	0.976583876271	0.964363587649
H580	H	0.681293487871	0.484519056851	0.092825204004
H581	H	0.372898523516	0.925575236145	0.970585885660
H582	H	0.614777247105	0.430354258824	0.089614674626
H583	H	0.319713555192	0.837755631068	0.904801775274
H584	H	0.669930393240	0.319991096778	0.142373135298
H585	H	0.362151303640	0.949140039533	0.856786625983
H586	H	0.630390590095	0.407597016787	0.200128015160
H587	H	0.379741612915	0.843906266424	0.817865198247
H588	H	0.638313823038	0.292316140106	0.232658528013
H589	H	0.420819915349	0.836935968137	0.856177532833
H590	H	0.589264484985	0.287790464946	0.197813506928
H591	H	0.231679071531	0.900745180946	0.886967161155
H592	H	0.763879636501	0.386218209725	0.160812345066
H593	H	0.174364152474	0.954531038380	0.824181681480
H594	H	0.821402783508	0.449342725764	0.221953095644
H595	H	0.119205655432	0.834014930116	0.868028464466
H596	H	0.875962581686	0.329359339515	0.177605139181
H597	H	0.078943547999	0.887429867318	0.838163768695
H598	H	0.917392703284	0.384672522818	0.206544981914
H599	H	0.207448195421	0.748841853965	0.843785546579
H600	H	0.792059148029	0.242837121507	0.203138371010
H601	H	0.079960247769	0.865898860472	0.780734923198
H602	H	0.917548880348	0.366617271765	0.264667838206
H603	H	0.235688176480	0.671330585562	0.795451452018
H604	H	0.763362653310	0.169283665047	0.252640508599
H605	H	0.108317904145	0.789267563231	0.732373319644
H606	H	0.890391831249	0.291862717559	0.314099713623
H607	H	0.166608470911	0.689381938124	0.714566173412
H608	H	0.835163569352	0.190379459704	0.332886436654
H609	H	0.215767390555	0.053077553440	0.859621246188
H610	H	0.778164156017	0.547949639496	0.188974956458
H611	H	0.156327480814	0.068676200125	0.924963983733
H612	H	0.825668121722	0.570097649206	0.122003722412
H613	H	0.271477022855	0.081195388330	0.915379507928
H614	H	0.719590445631	0.599125436900	0.141458505246
H615	H	0.239831966583	0.158932442606	0.935831516684
H616	H	0.753584784897	0.675025675991	0.121145814270
H617	H	0.255421097292	0.163654284146	0.892544680258
H618	H	0.748865492911	0.674174763708	0.165828076394
H619	H	0.121193397063	0.130596272322	0.847281964925
H620	H	0.884170071425	0.619361701697	0.199395266900
H621	H	0.086772444997	0.278766121572	0.872727530049
H622	H	0.927867357455	0.761766232599	0.171948864537
H623	H	0.105371337001	0.227580901549	0.800370142805
H624	H	0.914328510648	0.717669608230	0.245781405751
H625	H	0.091170149197	0.319008245832	0.814421478325
H626	H	0.927849281426	0.806868856004	0.228890734301
H627	H	0.169273252827	0.357747844892	0.862481304912
H628	H	0.846512826250	0.848245970876	0.185512081888
H629	H	0.244522720094	0.330723592313	0.854462079712
H630	H	0.772478303476	0.819342324318	0.194786839851
H631	H	0.958014645814	0.785614237943	0.551918495211
H632	H	0.043091105823	0.544337968985	0.515722514995
H633	H	0.004736998668	0.559656159088	0.484050744308
H634	H	0.965418170630	0.954475406408	0.530134152236
H635	H	0.014242570534	0.773320607378	0.530813460642
H636	H	0.034854108063	0.023449702848	0.506153740449
H637	H	0.009582922144	0.896092473252	0.543348687743
H638	H	0.023623440137	0.140934913000	0.469148779438
H639	H	0.068808733088	0.094657437834	0.515178993483

H640	H	0.993996843497	0.282629018265	0.484629643636
H641	H	0.057712624193	0.214264156200	0.468247953897
H642	H	0.036067530472	0.341654411157	0.499902743189
H643	H	0.578512487570	0.745958765190	0.209730965722
H644	H	0.420009366472	0.511742947918	0.151580941355
H645	H	0.486244618149	0.482614303817	0.152623018857
H646	H	0.561464555381	0.923514030894	0.165198287092
H647	H	0.517329199662	0.778240914204	0.194593042512
H648	H	0.522183830587	0.025704123731	0.192698015655
H649	H	0.566443227782	0.889800380712	0.200441606778
H650	H	0.502353049452	0.156357139734	0.167993613068
H651	H	0.510212928477	0.098445079920	0.212938590052
H652	H	0.539411134366	0.354803881859	0.132170829707
H653	H	0.496729448472	0.239186558267	0.178948453039
H654	H	0.526871833685	0.385740542426	0.169447114792
H655	H	0.726930251966	0.097694320941	0.919826514703
H656	H	0.281851726578	0.535294456779	0.126090625962
H657	H	0.773881778488	0.035212538326	0.912545766441
H658	H	0.212512837598	0.526631653929	0.123897933555
H659	H	0.248182450794	0.149927239037	0.822379036406
H660	H	0.750726536978	0.648666145248	0.227662625417
H661	H	0.281196229001	0.087015023432	0.805058463415
H662	H	0.722129584274	0.587478754392	0.248462855213
H663	H	0.330799123043	0.155016451399	0.848566768614
H664	H	0.649138028386	0.640504451322	0.219586101277
H665	H	0.348578120015	0.184460852888	0.885235761868
H666	H	0.606407034213	0.643601369082	0.187959323305
H667	H	0.295245819702	0.340591346176	0.745422344243
H668	H	0.709296357877	0.836969792142	0.298102348050
H669	H	0.295195859257	0.427965577343	0.751505583010
H670	H	0.711926058434	0.923449352987	0.290269881768
H671	H	0.228657512320	0.399053815462	0.666915572594
H672	H	0.773772521574	0.899517551064	0.376825849817
H673	H	0.239954253337	0.419530003386	0.704542096645
H674	H	0.765274653141	0.917198448845	0.338793897627
H675	H	0.219313977372	0.533472084884	0.676038817024
H676	H	0.782913920346	0.029719562551	0.366890243612
H677	H	0.257064683978	0.601802976960	0.687643908898
H678	H	0.744375583900	0.098118691024	0.356991387816
H679	H	0.317369404483	0.587165040340	0.733640547136
H680	H	0.688496990992	0.084411039417	0.303383366204
H681	H	0.271827738641	0.652950844209	0.730314864903
H682	H	0.733461688405	0.147890475435	0.313015685760
H683	H	0.378890007161	0.499534602484	0.760835383004
H684	H	0.630590114990	0.995465653644	0.282383691868
H685	H	0.326650036516	0.530371850606	0.782784594022
H686	H	0.674778796185	0.007197200615	0.251973934470
H687	H	0.671179390836	0.299954971820	0.820087933237
H688	H	0.342662867703	0.801030383735	0.228723218261
H689	H	0.711844468483	0.333514860863	0.848078736055
H690	H	0.316031352093	0.838730885761	0.196429733924
H691	H	0.896320975449	0.529846890203	0.774191847240
H692	H	0.108826690132	0.018475025931	0.273385567392
H693	H	0.888796350688	0.552515009285	0.735663574593
H694	H	0.112584390646	0.044767227148	0.311569022695
H695	H	0.737992962090	0.557788575045	0.723650359227
H696	H	0.265257582208	0.056573231077	0.323036557876
H697	H	0.790975304597	0.549747279579	0.750427712214
H698	H	0.214461345797	0.040945520473	0.296084839204
H699	H	0.725454418214	0.501290997105	0.670342634164
H700	H	0.271782600379	0.010347456347	0.375754852488
H701	H	0.704925568262	0.585028808876	0.665779817738
H702	H	0.291740637721	0.093367394393	0.381999938205

H703	H	0.019591712108	0.156429422428	0.570820354824
H704	H	0.982083088512	0.733301082459	0.479394002024
H705	H	0.989486998235	0.103366968420	0.595909257004
H706	H	0.031464775313	0.681688628702	0.493920474130
H707	H	0.545007320867	0.213161137966	0.793240949283
H708	H	0.525735563438	0.810774814067	0.246856109709
H709	H	0.583242312522	0.269901098187	0.774332595022
H710	H	0.474995935141	0.775700000235	0.267266899696
H711	H	0.492771818128	0.312232448234	0.755685106123
H712	H	0.549345671232	0.830368298104	0.302648874250
H713	H	0.491509607341	0.349452147397	0.720002076515
H714	H	0.534263953790	0.848027191386	0.339501063103
H715	H	0.473107557329	0.491084255987	0.786592270513
H716	H	0.521714421241	0.986009627478	0.286490686062
H717	H	0.465755921414	0.436932028706	0.755396104118
H718	H	0.563457135017	0.935727145406	0.310333752222
H719	H	0.307421776622	0.205433951921	0.753974828155
H720	H	0.695189032076	0.699453914307	0.293921661755
H721	H	0.336059866824	0.234413510023	0.721827295966
H722	H	0.661053905786	0.742018129890	0.320972854145
H723	H	0.124308136062	0.154258446498	0.563498817867
H724	H	0.878135751103	0.687396322178	0.469839898166
H725	H	0.101108308046	0.204274099775	0.533212517416
H726	H	0.889463153627	0.744466008086	0.497876338709
H727	H	0.078064600190	0.003238001829	0.623646323951
H728	H	0.924138151308	0.516630129497	0.427270484138
H729	H	0.137656960643	0.029320220948	0.608305463448
H730	H	0.860419192172	0.545106376370	0.436698739465
H731	H	0.238970910366	0.858353367538	0.676778551252
H732	H	0.765624421252	0.357672181211	0.375207958471
H733	H	0.197282049433	0.788405486993	0.682034246419
H734	H	0.805617284888	0.287326945222	0.368329714289
H735	H	0.253891653676	0.981917523607	0.521561495635
H736	H	0.745852243238	0.543304108851	0.493448089681
H737	H	0.312599617517	0.013145251061	0.539972185852
H738	H	0.676968744180	0.533495475785	0.499805858953
H739	H	0.348951934926	0.798984676521	0.759216793483
H740	H	0.659844957350	0.280433190200	0.294761542838
H741	H	0.383302196710	0.740020306417	0.780163878777
H742	H	0.636627084695	0.201883565950	0.302128096053
H743	H	0.258464583752	0.849683900697	0.768233598257
H744	H	0.744190651829	0.343300879501	0.281606586912
H745	H	0.258670991736	0.825394124830	0.729035587860
H746	H	0.745134366951	0.323596880300	0.321420158055
H747	H	0.467415831210	0.580483769807	0.748466006336
H748	H	0.550821674980	0.076581847336	0.313941605916
H749	H	0.477410199343	0.650018640828	0.769982162098
H750	H	0.547298039864	0.145053437681	0.290854201414
H751	H	0.509599379754	0.095417873817	0.822862959002
H752	H	0.551392214586	0.595550018080	0.223982548652
H753	H	0.499560205973	0.008575255990	0.824895006875
H754	H	0.517443244370	0.517523481029	0.223335744463
H755	H	0.509021389006	0.719009016901	0.823806430988
H756	H	0.522505414161	0.205927210961	0.237938275025
H757	H	0.441254812439	0.691836112350	0.821740168746
H758	H	0.581735543466	0.160761755304	0.234361999720
H759	H	0.576651424387	0.772205863113	0.914219251275
H760	H	0.443332227004	0.348653228176	0.147946152267
H761	H	0.583217290964	0.792314704158	0.875403620786
H762	H	0.390834606961	0.330080545753	0.173613188694
H763	H	0.126827441193	0.696519328907	0.658327690767
H764	H	0.878177860516	0.194361658277	0.388673318882
H765	H	0.093048604013	0.758807073368	0.678849170246

H766	H	0.910359574205	0.257626873327	0.368131855741
H767	H	0.932150941292	0.697717992336	0.644025248405
H768	H	0.039560580148	0.171750219248	0.410391364801
H769	H	0.950919667842	0.710970967469	0.679939709462
H770	H	0.062269675972	0.205896792945	0.376326789034
H771	H	0.894319816487	0.525298185140	0.828956645240
H772	H	0.116346033204	0.006045458119	0.219992595677
H773	H	0.955637531458	0.482887001293	0.829292321001
H774	H	0.054356299206	0.965680398118	0.221115951793
H775	H	0.692049079335	0.457780206177	0.853585832910
H776	H	0.324488196349	0.965198394528	0.178312161826
H777	H	0.762380133395	0.471344484548	0.849298136181
H778	H	0.256891560812	0.966141682914	0.190584119341
H779	H	0.888562291862	0.448323338578	0.012887770250
H780	H	0.109017748765	0.927219355079	0.037943627530
H781	H	0.942923149724	0.497305682823	0.001695954549
H782	H	0.058971965186	0.983525418684	0.048476327784
H783	H	0.062505974613	0.468668782021	0.004870435332
H784	H	0.942518833906	0.004857842254	0.036619656804
H785	H	0.043254158862	0.516740379992	0.036096613029
H786	H	0.990083589503	0.046395476855	0.013325709437
H787	H	0.955910831753	0.620793892855	0.084523901746
H788	H	0.018015784061	0.109922392499	0.963369495877
H789	H	0.983539194289	0.653750900534	0.051690204852
H790	H	0.988373331640	0.155845801216	0.992780769033
H791	H	0.948492718256	0.537374812900	0.133521839244
H792	H	0.048273769843	0.047990539925	0.909057619253
H793	H	0.992294031445	0.599975182188	0.144521978583
H794	H	0.016562822502	0.120334924388	0.897593397694
H795	H	0.104179720661	0.541642805593	0.084463588870
H796	H	0.875584671798	0.075314977511	0.923444510937
H797	H	0.039052086327	0.559465592691	0.093801444158
H798	H	0.941587430734	0.097513860476	0.925353152768
H799	H	0.314193450610	0.753102077547	0.049943681520
H800	H	0.592053018171	0.307483475951	0.029756146051
H801	H	0.369231270206	0.796182364845	0.034929669674
H802	H	0.600734069817	0.269043503924	0.064957736025
H803	H	0.278197009882	0.660983387468	0.878005430720
H804	H	0.725394441796	0.125644545579	0.159668489203
H805	H	0.268895569019	0.590634893612	0.857102689185
H806	H	0.715936517104	0.045014125667	0.172044574232
H807	H	0.340502942025	0.617895173336	0.825057721213
H808	H	0.656852757845	0.071555030231	0.206902453032
H809	H	0.347842216777	0.534030830513	0.835726890173
H810	H	0.630803365403	0.991246728560	0.199533280873
H811	H	0.980947718670	0.406940065346	0.879384149336
H812	H	0.035955484255	0.897639953595	0.170580970605
H813	H	0.993861211075	0.361874328719	0.847598437469
H814	H	0.021919487829	0.842320130884	0.198921453555
H815	H	0.073084863130	0.419642115650	0.874131286289
H816	H	0.940563491483	0.909180253261	0.177363833572
H817	H	0.095382477192	0.392623400373	0.908380502837
H818	H	0.920503297823	0.878576076575	0.143441820574
H819	H	0.080501831973	0.585282895278	0.876639819660
H820	H	0.828042696494	0.064955979712	0.161599055904
H821	H	0.104049840251	0.522886355288	0.899024378696
H822	H	0.880219555459	0.009720693572	0.158117942073
H823	H	0.632328656799	0.308426526732	0.873573693379
H824	H	0.608150004158	0.388432883854	0.876821770392
H825	H	0.455239938476	0.658174216123	0.086253177704
H826	H	0.460626893949	0.699543346406	0.051244811775
H827	H	0.383949497280	0.738669153979	0.953360533281
H828	H	0.353920114321	0.716596559099	0.918170507331

H829	H	0.391306235860	0.226078977064	0.994854172743
H830	H	0.338329797772	0.172725684617	0.986534059443
H831	H	0.437625681734	0.604735555752	0.951528481974
H832	H	0.391391328439	0.566696149532	0.927222246356
H833	H	0.578775838853	0.927353423695	0.040742644800
H834	H	0.548883192809	0.951887658755	0.005481595426
H835	H	0.295672804156	0.562913372300	0.904998252457
H836	H	0.308201576260	0.510696805552	0.937765849419
H837	H	0.526399409222	0.254864981560	0.090116315555
H838	H	0.541377508683	0.235029565629	0.127738167191
H839	H	0.451434699747	0.644658715158	0.901594297107
H840	H	0.477798546219	0.674563294086	0.866701229044
H841	H	0.512343720753	0.335549928642	0.997823007114
H842	H	0.567977152986	0.383029597654	0.986725466575
H843	H	0.449006729148	0.999925583370	0.092261089935
H844	H	0.495650661220	0.064983691671	0.094388022049
H845	H	0.422252664421	0.921847891801	0.030378469486
H846	H	0.477581960263	0.872128176040	0.034864742433
H847	H	0.425951465704	0.030662839801	0.175373280191
H848	H	0.407419003736	0.041599270420	0.138437417034
H849	H	0.573544429989	0.684030722447	0.951684836608
H850	H	0.518905677129	0.700603530247	0.927282846838
H851	H	0.652708156553	0.958498070438	0.085806041157
H852	H	0.650198192419	0.873670970618	0.074529017756
H853	H	0.617464544925	0.090303223581	0.899198074487
H854	H	0.595150157713	0.015687703021	0.884763514767
H855	H	0.557126487968	0.285723565886	0.921713611526
H856	H	0.565282432909	0.209668312445	0.939343502925
H857	H	0.406634004677	0.746863183229	0.094340436171
H858	H	0.346340433408	0.753546425328	0.112123097262
H859	H	0.426878483986	0.064639063665	0.933493888185
H860	H	0.465012331808	0.111168739417	0.961050612712
H861	H	0.523977478643	0.670188566456	0.013360584686
H862	H	0.483640079234	0.737281863152	0.000333761308
H863	H	0.616427855132	0.079761138223	0.094380697248
H864	H	0.576377708143	0.151511704302	0.096191290057
H865	H	0.333955806784	0.383434712080	0.948194927367
H866	H	0.396487531561	0.392975309265	0.930622830282
H867	H	0.422849813473	0.933727444040	0.134365928867
H868	H	0.414964691523	0.863840760239	0.111015515484
H869	H	0.548019557144	0.508830962774	0.075590363644
H870	H	0.528970298813	0.584440437978	0.092468431755
H871	H	0.472407420778	0.165651689027	0.901470437496
H872	H	0.433474952167	0.185316453994	0.869399250835
H873	H	0.405015043858	0.113407249526	0.073073525187
H874	H	0.371749800985	0.161219625872	0.043060687194
H875	H	0.643278560523	0.771320280108	0.106933543379
H876	H	0.605927285828	0.799772161207	0.136598220053
H877	H	0.706817674415	0.024020427481	0.964661186772
H878	H	0.706019624609	0.006010905072	0.003287839871
H879	H	0.587767871582	0.570828205186	0.926211862153
H880	H	0.540640216732	0.595784997000	0.898874963034
H881	H	0.530973054891	0.646066450000	0.131958759212
H882	H	0.474958315061	0.597609797462	0.137984636716
H883	H	0.320027386107	0.654339082431	0.972731526926
H884	H	0.322706790539	0.690528807189	0.007986037629
H885	H	0.646206444586	0.780452592044	0.041514589059
H886	H	0.672278808063	0.723655444783	0.067626600215
H887	H	0.390374200375	0.170111230294	0.934420355070
H888	H	0.363875441777	0.250477032106	0.932323743737
H889	H	0.674645889192	0.148796051872	0.998246832979
H890	H	0.685453988013	0.227387169058	0.985277368893
H891	H	0.574684234378	0.761205376743	0.007167999240

H892	H	0.596264296621	0.841172528014	0.005599366144
H893	H	0.578599273718	0.899144429843	0.109265903670
H894	H	0.512499082457	0.884931717410	0.122899678111
H895	H	0.533938762646	0.121517188415	0.006835962094
H896	H	0.481788389704	0.173667891605	0.015354111981
H897	H	0.640234577242	0.146999264158	0.952188503149
H898	H	0.584963043730	0.091760026703	0.949370223355
H899	H	0.526824421545	0.410510978538	0.043804479237
H900	H	0.477191551412	0.444464914686	0.066807957502
H901	H	0.450749351647	0.813674702898	0.957383589109
H902	H	0.431114157161	0.827531178497	0.995924608455
H903	H	0.529870492593	0.181566334702	0.853382892772
H904	H	0.585066888219	0.130714706915	0.847853161721
H905	H	0.265782222135	0.542136376349	0.062676124517
H906	H	0.288135384245	0.576910009923	0.028194732290
H907	H	0.431593392189	0.762424557119	0.152873709986
H908	H	0.406535551431	0.784171665830	0.187587487904
H909	H	0.442159769011	0.049442723429	0.015830626683
H910	H	0.372001082687	0.031750774339	0.015913049369
H911	H	0.582177888320	0.888203251442	0.903426034024
H912	H	0.538841930270	0.930232225373	0.879246546766
H913	H	0.335187930457	0.341302311478	0.889160006034
H914	H	0.341804040707	0.289184962691	0.858420278118
H915	H	0.490576384836	0.873254906720	0.911726492442
H916	H	0.467025208931	0.793139170398	0.903891909591
H917	H	0.556116866362	0.437980339661	0.941527879239
H918	H	0.607816994351	0.385511183933	0.932572075512
H919	H	0.448953811042	0.209132156917	0.061742247866
H920	H	0.458342448780	0.287580592000	0.049696692054
H921	H	0.609038559149	0.186093241227	0.028985194195
H922	H	0.598414666748	0.111250417945	0.045925802182
H923	H	0.740225926286	0.924159525591	0.032543061086
H924	H	0.797438989219	0.958685200709	0.047562924493
H925	H	0.376320922184	0.423867906377	0.858601618252
H926	H	0.368102839055	0.489506670207	0.883194866262
H927	H	0.335922799892	0.652737817251	0.079971018530
H928	H	0.272859225485	0.644606179153	0.096685953374
H929	H	0.389854283993	0.427193655845	0.062131674902
H930	H	0.362510196893	0.505923209048	0.057989477444
H931	H	0.718570457110	0.026348617435	0.078176289852
H932	H	0.711218978397	0.049172070617	0.117352982200
H933	H	0.372323854847	0.425199235616	0.140241806995
H934	H	0.371903837422	0.477317025656	0.108642252215
H935	H	0.526018518551	0.343199961630	0.875591215818
H936	H	0.471033728102	0.294678714297	0.882064102292
H937	H	0.283233356367	0.470691687763	0.997603801610
H938	H	0.348935003361	0.457607547875	0.985479704538
H939	H	0.483176026076	0.267274639389	0.961646276317
H940	H	0.423339678491	0.313761371727	0.963226934482
H941	H	0.466071018725	0.485158806781	0.924441399960
H942	H	0.476099239645	0.402050231572	0.909630959598
H943	H	0.623288729515	0.680283571294	0.139343218535
H944	H	0.622588478562	0.589104598728	0.142055306979
H945	H	0.554575651297	0.573405324266	0.035426708244
H946	H	0.509817460869	0.562599579783	0.004779857324
H947	H	0.446735090260	0.440548066324	0.974101396392
H948	H	0.427018427053	0.496737388662	0.002876045329
H949	H	0.607608708191	0.001040879794	0.973181019882
H950	H	0.557060312703	0.979284186320	0.946393756854
H951	H	0.428307603079	0.607828150733	0.031871865275
H952	H	0.381729798745	0.604187419743	0.002655443646
H953	H	0.681900859427	0.602388738626	0.058396047241
H954	H	0.629119620500	0.613966489830	0.081751616739

H955	H	0.607248054702	0.592587145361	0.981695380712
H956	H	0.666485599182	0.608048787111	0.959316604946
H957	H	0.577088483917	0.479973693529	0.842369403006
H958	H	0.598058389104	0.504468687533	0.877387909141
H959	H	0.351796147394	0.324509876128	0.041803379240
H960	H	0.401698674069	0.370444401456	0.021661689845
H961	H	0.464592450602	0.499717360633	0.844107697276
H962	H	0.494196405862	0.569946394299	0.828058202856
H963	H	0.416596171075	0.679716753262	0.244867409153
H964	H	0.409904643214	0.675366844523	0.191223269894
H965	H	0.423009792523	0.598099672437	0.205432610247
H966	H	0.451537766145	0.878704224101	0.238044252302
H967	H	0.477752250929	0.650307013794	0.255705794660
H968	H	0.443013901642	0.987092779880	0.249686517351
H969	H	0.471602878691	0.920858457316	0.206521073033
H970	H	0.347237462957	0.284605266119	0.225990692518
H971	H	0.466850977367	0.065140594886	0.260786275876
H972	H	0.388517432140	0.416460078039	0.219243603213
H973	H	0.417972081876	0.294128077790	0.220463960900
H974	H	0.424636751830	0.477980402516	0.200700870053
C1	C	0.719550514019	0.834193587097	0.945238302822
C2	C	0.288866045517	0.312270938925	0.110235996614
C3	C	0.743972165007	0.835338082578	0.909731866793
C4	C	0.254223871921	0.321812341877	0.142958803689
C5	C	0.769580210343	0.841064866918	0.972624759153
C6	C	0.244883868984	0.308758735263	0.079898254195
C7	C	0.809427427518	0.908770450562	0.969158952593
C8	C	0.209074086108	0.380032418478	0.075602311347
C9	C	0.767887612963	0.912972556268	0.861558690953
C10	C	0.217041167960	0.404486023102	0.185570757933
C11	C	0.828789600157	0.949705489086	0.868838206266
C12	C	0.156402140760	0.431132526083	0.171796126892
C13	C	0.727854486809	0.959096779085	0.837129584291
C14	C	0.239415666120	0.467091261799	0.210010496448
C15	C	0.667522497899	0.920294395296	0.831051610311
C16	C	0.258352774691	0.436716987778	0.244173971185
C17	C	0.937342953428	0.945792982683	0.857643871427
C18	C	0.047696819536	0.426022971232	0.184641462049
C19	C	0.965537082687	0.961116497359	0.823451471118
C20	C	0.024114556248	0.444418497004	0.219831068287
C21	C	0.973505098100	0.884137206466	0.875287760234
C22	C	0.009945665343	0.362952619952	0.169243209453
C23	C	0.014592726447	0.045198931476	0.785642774569
C24	C	0.984226338318	0.533341874161	0.258919925502
C25	C	0.083418756788	0.050010482050	0.785757767026
C26	C	0.915403049982	0.539596003860	0.260958465877
C27	C	0.984317914120	0.115943359305	0.772575308301
C28	C	0.016054314848	0.604208521045	0.270617330168
C29	C	0.177845287074	0.025248500303	0.756917043994
C30	C	0.823698675622	0.516736804517	0.292851710384
C31	C	0.203736550737	0.102366345342	0.751258161131
C32	C	0.798417925899	0.593792096248	0.298929783574
C33	C	0.190939658752	0.972660706439	0.727641137085
C34	C	0.813299528555	0.464851971913	0.322762168613
C35	C	0.136443194120	0.982727770538	0.704052335009
C36	C	0.869907047994	0.474630271304	0.344711958433
C37	C	0.083688632998	0.988218263033	0.728249021465
C38	C	0.920288190578	0.480773563517	0.319008836186
C39	C	0.182814378894	0.230159037436	0.734601534124
C40	C	0.820568780740	0.721978575028	0.313179267312
C41	C	0.128333655448	0.279856995431	0.740388555733
C42	C	0.875555713159	0.769628957210	0.305914628027
C43	C	0.209031870818	0.243861511272	0.699882814493

C44	C	0.793900718849	0.740989036714	0.347223776503
C45	C	0.095828158711	0.406925529917	0.750178319928
C46	C	0.907327487506	0.896873823276	0.294320412664
C47	C	0.057270434648	0.418440029746	0.718236558709
C48	C	0.948320415894	0.905771474076	0.325523551051
C49	C	0.124239806162	0.478558291129	0.764498337637
C50	C	0.875969817479	0.969264574435	0.283512955408
C51	C	0.139238501247	0.466186177438	0.802090690304
C52	C	0.846432438232	0.959955259041	0.248458004651
C53	C	0.083121692189	0.544976796820	0.758828837006
C54	C	0.919434901437	0.034343355211	0.285260348170
C55	C	0.169349191727	0.530434012709	0.820572763389
C56	C	0.811042128447	0.027187506372	0.236141730649
C57	C	0.046505589383	0.425943952405	0.657891596022
C58	C	0.960241348692	0.918910913997	0.386017574801
C59	C	0.009157433009	0.355453830932	0.652763919960
C60	C	0.996311931167	0.848319240003	0.393348346071
C61	C	0.082777538212	0.449593830201	0.626615818998
C62	C	0.923499574891	0.948821480879	0.416017721007
C63	C	0.052696998759	0.425353434651	0.593404760955
C64	C	0.951283239059	0.929595419951	0.450472244394
C65	C	0.092026123633	0.533606756427	0.627864044881
C66	C	0.916218345252	0.032630657467	0.411643087227
C67	C	0.995893239212	0.223569136080	0.660318672732
C68	C	0.009829966275	0.716336358807	0.386144179564
C69	C	0.939958635648	0.233186116741	0.682728612534
C70	C	0.066215541920	0.725409501954	0.363844915125
C71	C	0.032556476112	0.154328041374	0.668802717230
C72	C	0.973217095225	0.647098651300	0.377530773838
C73	C	0.897585538507	0.287882434615	0.734226881799
C74	C	0.110071831532	0.775482596205	0.312067988519
C75	C	0.853974729780	0.342078852234	0.716908486821
C76	C	0.151252649613	0.832505350200	0.329365567377
C77	C	0.921794186768	0.321337764986	0.767360165772
C78	C	0.089057298410	0.804536854776	0.277386077967
C79	C	0.873412434679	0.355001923904	0.789982068649
C80	C	0.139526970450	0.836856611293	0.255522550911
C81	C	0.826467674254	0.302092733456	0.804147382448
C82	C	0.187358402575	0.783603328215	0.242366744488
C83	C	0.830674724008	0.183100358367	0.835221578646
C84	C	0.188156988334	0.674525706404	0.204116858840
C85	C	0.838698373012	0.452025719268	0.683338516280
C86	C	0.162066619895	0.945170476146	0.361625867493
C87	C	0.800892423934	0.410658543648	0.656370898283
C88	C	0.202959955742	0.908622343166	0.388593228087
C89	C	0.874151761777	0.515190666475	0.667246205541
C90	C	0.123976674390	0.006287651638	0.377549597832
C91	C	0.799661004496	0.319143923960	0.611668636602
C92	C	0.213298083182	0.824491509355	0.435438364043
C93	C	0.752433266165	0.267905275395	0.627046570533
C94	C	0.253837252879	0.764522521543	0.420614472414
C95	C	0.845035913490	0.275102828644	0.591234948185
C96	C	0.176069142951	0.797379143040	0.465189270226
C97	C	0.882945547770	0.325014332383	0.568433987552
C98	C	0.160214563554	0.862948677841	0.487924838762
C99	C	0.726874241680	0.185405316506	0.673532483120
C100	C	0.278507608698	0.682991085882	0.374044841314
C101	C	0.668619258698	0.226085436364	0.682395795417
C102	C	0.336628302681	0.723472025687	0.364460972694
C103	C	0.758635262849	0.153477392961	0.705143752402
C104	C	0.246794983494	0.647587901978	0.343187874850
C105	C	0.721608083824	0.094419480306	0.721941470821
C106	C	0.285085950917	0.588362769055	0.327336827106

C107	C	0.679114688017	0.111682112000	0.746611716947
C108	C	0.327303620843	0.605575144176	0.302525115008
C109	C	0.728162285114	0.020556167520	0.711963868370
C110	C	0.280416750079	0.514912425042	0.338285855923
C111	C	0.643726784221	0.056247206126	0.760797475053
C112	C	0.364340462230	0.550703818180	0.289298691237
C113	C	0.692932773633	0.964955713005	0.726099199161
C114	C	0.317812866029	0.460082791926	0.325254272895
C115	C	0.650354444472	0.983041946867	0.750371101745
C116	C	0.360310146699	0.478281871600	0.300973525606
C117	C	0.622258726980	0.332145491944	0.708258421855
C118	C	0.381342174166	0.831830788653	0.338610222791
C119	C	0.582967108589	0.355022529338	0.677793305710
C120	C	0.418908103588	0.855662088441	0.369584701584
C121	C	0.637805214340	0.399161398127	0.730183821566
C122	C	0.362363317854	0.898022488092	0.317120312386
C123	C	0.665497773716	0.379919739676	0.764405702299
C124	C	0.342229297604	0.876939746636	0.281502119065
C125	C	0.576915816246	0.391061490231	0.618650513467
C126	C	0.422590414670	0.892294257637	0.428581188864
C127	C	0.544218584632	0.321647870787	0.605464082691
C128	C	0.457066533818	0.824164179947	0.441678463114
C129	C	0.618051871676	0.427048110943	0.592111079013
C130	C	0.380508956730	0.925258808545	0.455423000677
C131	C	0.589046328402	0.424879959001	0.556966011442
C132	C	0.410061390412	0.923500377949	0.490490136853
C133	C	0.633339355225	0.506087064152	0.602803346428
C134	C	0.362029191829	0.003791945846	0.445523896643
C135	C	0.551119036965	0.189128842840	0.593212202876
C136	C	0.453915801207	0.691652997632	0.453943509679
C137	C	0.494912669410	0.167957458930	0.613628874299
C138	C	0.508809303640	0.671262468655	0.432312850514
C139	C	0.601971289045	0.131332909020	0.595378225496
C140	C	0.404072501639	0.632403925901	0.453795019764
C141	C	0.446856611249	0.170028889076	0.668679437578
C142	C	0.554184952364	0.676353737926	0.376662936340
C143	C	0.393574728078	0.223027251162	0.664157261846
C144	C	0.606173861116	0.731632598712	0.379948345713
C145	C	0.469045015960	0.170193136629	0.705538540773
C146	C	0.529604455315	0.671814515498	0.340361952428
C147	C	0.512955736370	0.107309554353	0.713948510905
C148	C	0.491655985702	0.602395669664	0.333984638077
C149	C	0.482603030000	0.032047045077	0.717710810513
C150	C	0.528513112612	0.531567781510	0.331342915065
C151	C	0.396847470806	0.992170255461	0.754634199390
C152	C	0.613784669403	0.490794585511	0.295104110956
C153	C	0.351128043214	0.339959354520	0.644738632478
C154	C	0.647490069045	0.848275142449	0.400311192641
C155	C	0.294304243279	0.308846849927	0.627211526903
C156	C	0.704100207685	0.815409492823	0.417156744784
C157	C	0.371271189969	0.411516760424	0.627573821602
C158	C	0.626390200499	0.917194293386	0.419301620306
C159	C	0.413096247562	0.462113911958	0.648596727428
C160	C	0.591515086190	0.973845576117	0.397785734961
C161	C	0.429813236697	0.528169687335	0.626431959611
C162	C	0.565922007837	0.032404784706	0.421497359837
C163	C	0.384443289568	0.486474432150	0.681729078369
C164	C	0.631566619100	0.008394426643	0.370912150991
C165	C	0.242943320984	0.229078705550	0.586547052103
C166	C	0.752638996066	0.734133735801	0.458347337554
C167	C	0.222276473789	0.160803334660	0.606032565909
C168	C	0.768125595759	0.660721293975	0.441382892799
C169	C	0.259790569085	0.207380646786	0.550502582591

C170	C	0.736398578218	0.722030389947	0.495135596125
C171	C	0.328598774495	0.199494831099	0.551615988971
C172	C	0.667877709661	0.712445436687	0.494313461961
C173	C	0.349489998184	0.258490696901	0.576575989897
C174	C	0.647275446920	0.768268123130	0.467829055382
C175	C	0.241033790026	0.073913534345	0.652284364819
C176	C	0.755300898960	0.578271752522	0.393089926546
C177	C	0.273787636999	0.002263169271	0.644248751205
C178	C	0.729388857071	0.506106741183	0.406185063673
C179	C	0.351632722946	0.940697111419	0.610967967822
C180	C	0.651419568695	0.446585200216	0.439591483857
C181	C	0.374590708474	0.901358150302	0.642893828227
C182	C	0.632333128053	0.401596523354	0.408506614607
C183	C	0.407848938839	0.967345861822	0.591706834514
C184	C	0.592437634945	0.470455687728	0.456912450574
C185	C	0.442677382097	0.903140285844	0.576728698284
C186	C	0.560140334317	0.404794240265	0.472432844964
C187	C	0.393949487320	0.788972076758	0.674922440475
C188	C	0.616375929535	0.285195940500	0.379659109895
C189	C	0.459502409160	0.809112009629	0.682505882439
C190	C	0.550893136604	0.303944520586	0.371264897938
C191	C	0.388275260892	0.707187311583	0.665150774868
C192	C	0.622845221083	0.205197320897	0.391593083369
C193	C	0.330246639459	0.706217746313	0.643943699031
C194	C	0.679534429363	0.207931429975	0.413949093742
C195	C	0.332036979825	0.778367346022	0.623816945686
C196	C	0.675923723054	0.282783937809	0.431853095768
C197	C	0.536585254449	0.819104648374	0.726456757351
C198	C	0.476779678419	0.317760139095	0.326347359979
C199	C	0.578426901301	0.765039025013	0.708402301715
C200	C	0.431410658120	0.265347470666	0.342621942173
C201	C	0.544609859316	0.809982861241	0.764740778545
C202	C	0.472086101365	0.311231340583	0.287989141453
C203	C	0.513608923882	0.869002671505	0.785954769031
C204	C	0.509943296472	0.367045851804	0.268608875308
C205	C	0.525992444607	0.862819175120	0.823779203268
C206	C	0.509543366523	0.355211477950	0.230710017999
C207	C	0.597804137780	0.640457246659	0.688613347115
C208	C	0.404255003135	0.142005066579	0.360674445723
C209	C	0.615244106351	0.661953967299	0.652575326393
C210	C	0.386260077611	0.163344014757	0.396586193800
C211	C	0.568244893351	0.564115953789	0.689816094467
C212	C	0.428942831142	0.063199684675	0.358857709393
C213	C	0.584684623526	0.714735897679	0.598374389235
C214	C	0.415425630630	0.215689235584	0.451193609850
C215	C	0.623784849447	0.784502582194	0.599137792709
C216	C	0.378040062195	0.286877240781	0.450111593819
C217	C	0.525998505873	0.726400254102	0.577940508190
C218	C	0.473566163657	0.225815014125	0.472277727963
C219	C	0.497059895327	0.654439890233	0.564751012400
C220	C	0.502755506372	0.153034938877	0.484210676975
C221	C	0.431776475596	0.668210478803	0.552750050458
C222	C	0.568316566329	0.166462110951	0.495823252522
C223	C	0.534947504555	0.618812582731	0.537154505603
C224	C	0.465535030135	0.115253927551	0.511529685911
C225	C	0.651880903819	0.897793944730	0.626992260955
C226	C	0.354956121940	0.402432131703	0.422511281050
C227	C	0.716434316149	0.877025421145	0.638469046923
C228	C	0.290970816244	0.384392684783	0.409489409831
C229	C	0.623667146668	0.957210214660	0.649029422531
C230	C	0.384652796202	0.460509426463	0.400364907123
C231	C	0.783130521508	0.788246907082	0.667629188744
C232	C	0.225591436537	0.300178554931	0.376923291877

C233	C	0.817493422175	0.759342812251	0.636924794253
C234	C	0.188342032251	0.271619274231	0.406580742519
C235	C	0.776311690257	0.728195954163	0.694460917252
C236	C	0.233883884670	0.242828277326	0.349151039698
C237	C	0.821126148860	0.687517836874	0.586198512957
C238	C	0.177658190708	0.194048922344	0.454883485171
C239	C	0.830849607332	0.752532924238	0.561960071362
C240	C	0.165977900461	0.257383325415	0.479486969163
C241	C	0.792316967862	0.620258793393	0.568565617773
C242	C	0.203721876405	0.125546739015	0.472753651614
C243	C	0.799511093668	0.548112244006	0.588114499488
C244	C	0.195260307370	0.055576841485	0.451911789996
C245	C	0.799480486087	0.869927224166	0.538066457986
C246	C	0.198873286572	0.369620621244	0.508107213647
C247	C	0.852849767287	0.920082973342	0.547767862808
C248	C	0.146286656302	0.419886980185	0.496834248087
C249	C	0.741361405972	0.916829847482	0.533856841309
C250	C	0.257394233930	0.415439565079	0.512525254699
C251	C	0.749683124310	0.975369367968	0.507177624344
C252	C	0.249762598417	0.476346707219	0.538023242612
C253	C	0.759189898511	0.954467056572	0.473761118709
C254	C	0.244763922856	0.458634918399	0.572127405899
C255	C	0.754111351259	0.050573730660	0.514994505130
C256	C	0.243816906666	0.550704097419	0.528834541394
C257	C	0.773332358118	0.005675038735	0.449040887963
C258	C	0.234453396550	0.512282263647	0.596361806083
C259	C	0.768492067849	0.102831561127	0.490557593269
C260	C	0.233339510259	0.605366893046	0.552794117899
C261	C	0.779266999889	0.080737335355	0.457354659404
C262	C	0.228079780794	0.586692828984	0.586896837693
C263	C	0.899064181380	0.993165162773	0.593623013676
C264	C	0.106753258500	0.497591266854	0.451774995122
C265	C	0.958461837452	0.964826460560	0.608624459815
C266	C	0.045187885737	0.466552165686	0.441364218831
C267	C	0.867994520143	0.038847803551	0.621471757470
C268	C	0.133090938205	0.539839264142	0.421597011070
C269	C	0.811021227837	0.077899956059	0.609054035645
C270	C	0.188239098254	0.585429357969	0.431654882299
C271	C	0.010754185165	0.859208328926	0.634419246960
C272	C	0.993384167631	0.363848912775	0.412608516251
C273	C	0.002434744357	0.862015286615	0.672562179059
C274	C	0.000174005100	0.362778811341	0.374379330735
C275	C	0.937264162551	0.898539090853	0.719234280885
C276	C	0.064010073444	0.394859379599	0.326714774271
C277	C	0.915510399871	0.819468140020	0.727748578066
C278	C	0.083259237974	0.314055078574	0.319913887866
C279	C	0.886074171936	0.953283253564	0.726627795399
C280	C	0.116656557839	0.446430332441	0.317821051774
C281	C	0.892090268552	0.732943810342	0.772687350836
C282	C	0.098321086556	0.222254337376	0.275933371811
C283	C	0.843042880188	0.737668300907	0.799175951734
C284	C	0.145157217820	0.225039179214	0.248257537867
C285	C	0.950075723008	0.692923157709	0.785942797936
C286	C	0.039156479837	0.181436317783	0.264992633368
C287	C	0.976577574793	0.727479428285	0.818335645637
C288	C	0.012572717665	0.210136652484	0.231391895071
C289	C	0.995898923719	0.688681701459	0.757525619422
C290	C	0.995015613584	0.185403226153	0.294257898302
C291	C	0.027601791208	0.682323921123	0.834677253089
C292	C	0.962212733055	0.162014883478	0.216373303509
C293	C	0.770835814340	0.665050737714	0.831782893335
C294	C	0.218277058116	0.153845193721	0.216431238107
C295	C	0.799813727479	0.608592506051	0.855449687020

C296	C	0.197205633194	0.093880420283	0.192253996089
C297	C	0.706348651714	0.636641460732	0.822697414688
C298	C	0.283261514345	0.136924753573	0.229041127408
C299	C	0.676549874223	0.678027391737	0.793124907774
C300	C	0.300071135293	0.186937543762	0.258841765541
C301	C	0.666820052478	0.639408246921	0.854200374037
C302	C	0.326878526526	0.142544512326	0.199394354196
C303	C	0.622392578263	0.635569082445	0.778690795656
C304	C	0.362547776427	0.172230341555	0.274014910891
C305	C	0.823928731837	0.573831550574	0.913098437254
C306	C	0.171016102304	0.064063539887	0.134130607726
C307	C	0.773017589721	0.561230607667	0.937964530687
C308	C	0.223656030117	0.064359935898	0.109505307972
C309	C	0.884182967798	0.595889127566	0.931025257664
C310	C	0.108745305796	0.080787636310	0.116944076250
C311	C	0.877839989596	0.666971050839	0.952042433653
C312	C	0.110579223988	0.150563155321	0.094748663555
C313	C	0.934811047644	0.599924839594	0.905057963500
C314	C	0.059210520346	0.083239414776	0.143694227155
C315	C	0.933106056098	0.685192857753	0.973990502911
C316	C	0.052138545788	0.166307863723	0.074968849134
C317	C	0.724847840240	0.480482481814	0.979051527181
C318	C	0.280950131489	0.001612414368	0.066065708767
C319	C	0.735255339100	0.528266214404	0.010332972644
C320	C	0.259044217906	0.034229772476	0.032519684015
C321	C	0.729353258252	0.396646917447	0.986242614200
C322	C	0.298553466041	0.919842237143	0.063194540078
C323	C	0.786787813143	0.370215536215	0.968600851269
C324	C	0.241385605521	0.878171020569	0.073728036177
C325	C	0.797347042265	0.425543131984	0.940193013536
C326	C	0.214116218118	0.926809909458	0.101146713296
C327	C	0.806124144175	0.595842758093	0.046935856329
C328	C	0.175693724481	0.083030424095	0.999683645380
C329	C	0.792185727240	0.678668862384	0.042949252200
C330	C	0.190140179877	0.164903307371	0.006253887665
C331	C	0.808773606675	0.801264011382	0.070834858751
C332	C	0.182723708779	0.292091309507	0.983088618151
C333	C	0.869225373278	0.838879185753	0.062887892964
C334	C	0.121501919009	0.332155857847	0.984188092958
C335	C	0.788244444790	0.828090716459	0.105565204332
C336	C	0.219762493648	0.320127448414	0.953355733897
C337	C	0.979884199049	0.822038193826	0.066479482047
C338	C	0.011119699149	0.314862906962	0.977019135353
C339	C	0.013275531755	0.801234495101	0.034449529237
C340	C	0.972877101762	0.295413422008	0.007464593444
C341	C	0.010620375839	0.789826805733	0.098415383714
C342	C	0.987994216519	0.284029356574	0.943043746905
C343	C	0.070937739157	0.826124996946	0.106550719857
C344	C	0.925344851681	0.311334397287	0.933538781822
C345	C	0.045925003247	0.847670807317	0.980152003804
C346	C	0.942188377766	0.340639633233	0.062537959920
C347	C	0.113567703903	0.834327929888	0.985909547281
C348	C	0.873294921955	0.340752832613	0.058534567092
C349	C	0.039095904081	0.917975621791	0.959071275746
C350	C	0.960919301035	0.404270132247	0.085710629858
C351	C	0.206548695304	0.781335311723	0.965180739632
C352	C	0.776797633481	0.287437658410	0.074070788899
C353	C	0.237359219567	0.852825892061	0.953238742240
C354	C	0.751274206335	0.353820673582	0.093419188630
C355	C	0.216628787572	0.715980902498	0.941058207209
C356	C	0.761308752965	0.212418637195	0.090369081258
C357	C	0.166450867153	0.724767953937	0.914603582251
C358	C	0.813188297968	0.200764522425	0.115756751873

C359	C	0.112526290529	0.750255372424	0.935173215077
C360	C	0.868518710973	0.232100518417	0.098097033743
C361	C	0.330644141399	0.927127398585	0.955919250014
C362	C	0.660922475103	0.431593481710	0.100041646215
C363	C	0.346368182642	0.941635494095	0.919247834621
C364	C	0.655717173193	0.432816465065	0.138221449102
C365	C	0.337787941186	0.896885367636	0.860673499117
C366	C	0.661468411534	0.364637820924	0.191374232604
C367	C	0.276384708010	0.906807174311	0.842330582108
C368	C	0.722610167264	0.387595767460	0.206855948495
C369	C	0.375207014536	0.834588029762	0.844952293494
C370	C	0.637177014572	0.291345381527	0.205020271024
C371	C	0.351556390108	0.756347411752	0.850294624504
C372	C	0.663953970583	0.217861106881	0.193586789439
C373	C	0.167300121957	0.928460318499	0.848756638097
C374	C	0.827892504407	0.423123100532	0.197356547150
C375	C	0.143674767404	0.987860190944	0.872666702091
C376	C	0.848914487714	0.483623752369	0.173187574966
C377	C	0.123429935477	0.863788787713	0.844092717009
C378	C	0.873169561570	0.359845675118	0.201494755433
C379	C	0.141472440256	0.813689046983	0.815767619124
C380	C	0.857239005221	0.310293614720	0.230372663506
C381	C	0.185529610537	0.758503767447	0.819468119200
C382	C	0.813967783295	0.254110350554	0.227338253907
C383	C	0.114842307751	0.823770118560	0.784005839706
C384	C	0.883453672590	0.323431684248	0.261980910795
C385	C	0.201576269732	0.714294198857	0.792357962340
C386	C	0.797880448796	0.211940849755	0.255072840577
C387	C	0.130505401927	0.780353430415	0.756579033657
C388	C	0.868348634612	0.281406481358	0.289956045028
C389	C	0.173517496070	0.724391365201	0.760855137204
C390	C	0.826218194157	0.224297551872	0.286310917662
C391	C	0.176192893907	0.098205157859	0.903596115745
C392	C	0.816471658820	0.598574136310	0.145880653471
C393	C	0.134762604744	0.163479299407	0.896062290678
C394	C	0.867775208069	0.653995884485	0.151030366532
C395	C	0.239378665470	0.127064551646	0.912518586851
C396	C	0.756071065863	0.639856048893	0.143431174774
C397	C	0.082320057036	0.236437445470	0.853259826992
C398	C	0.931647855588	0.719726071897	0.191555501929
C399	C	0.014001666864	0.222031934449	0.849782406600
C400	C	0.999835512813	0.702286394005	0.193299101904
C401	C	0.112083541858	0.266132576468	0.821293239347
C402	C	0.905935913039	0.753445101637	0.224035221490
C403	C	0.180248976256	0.274369560987	0.827249806398
C404	C	0.837478882434	0.762284566953	0.220143182592
N1	N	0.687000800878	0.762948841751	0.949823318069
N2	N	0.324350968390	0.242820872542	0.113414165207
N3	N	0.738307731771	0.900027665418	0.893436021761
N4	N	0.257891393806	0.387536641263	0.158519124419
N5	N	0.875078237985	0.923401094681	0.851353353944
N6	N	0.110220792700	0.404619344290	0.189529112027
N7	N	0.993235281397	0.026023602299	0.818869737004
N8	N	0.001921853876	0.512261804958	0.225216740218
N9	N	0.111416653234	0.024329390087	0.758287357642
N10	N	0.889864917354	0.516066253699	0.289618005269
N11	N	0.165328191039	0.154041628654	0.739637899035
N12	N	0.837275705666	0.645191540681	0.310060821647
N13	N	0.142211764317	0.350901850302	0.746048337812
N14	N	0.861882466190	0.840004490235	0.299040785740
N15	N	0.084429720830	0.421124517503	0.688198230726
N16	N	0.922237424305	0.911470775788	0.355820087140
N17	N	0.033488970689	0.289938369417	0.661604120564

N18	N	0.972403920756	0.782621124959	0.384314030398
N19	N	0.947983825300	0.266323776938	0.712901356045
N20	N	0.058454915822	0.754630557449	0.332756312728
N21	N	0.855402672976	0.247402355384	0.826179401643
N22	N	0.160513336392	0.733158479771	0.217450473710
N23	N	0.772044049820	0.169487130703	0.829294396239
N24	N	0.247882283613	0.665477901603	0.206711898196
N25	N	0.866413560218	0.130907911309	0.849047446382
N26	N	0.153426311504	0.623084118015	0.188600672275
N27	N	0.878084581805	0.400391425783	0.701054028787
N28	N	0.124868645455	0.889640407847	0.344896565687
N29	N	0.830650142914	0.362666979759	0.637399187760
N30	N	0.176561576990	0.861993715336	0.410090394455
N31	N	0.933556604104	0.292796612545	0.557124706129
N32	N	0.101397209491	0.870559793829	0.494490101458
N33	N	0.768009950096	0.234219223283	0.656004896065
N34	N	0.238049926131	0.733849204958	0.391089705003
N35	N	0.675593930073	0.291824182583	0.698021957856
N36	N	0.329230588413	0.788425711795	0.348467263850
N37	N	0.703386972536	0.429095460716	0.777664284413
N38	N	0.310328035340	0.928321627739	0.264981497032
N39	N	0.611180819676	0.375777340121	0.649590931210
N40	N	0.389230229911	0.876089380142	0.397429786869
N41	N	0.577069286099	0.259217136181	0.604173695762
N42	N	0.425912534484	0.760405786365	0.442918494491
N43	N	0.496682595442	0.185811316820	0.646417413390
N44	N	0.505368739749	0.690357572446	0.399787138616
N45	N	0.447316347069	0.030614402291	0.748423896608
N46	N	0.564965112964	0.532645543461	0.300953802649
N47	N	0.366577087608	0.954963189159	0.730958461764
N48	N	0.640745640606	0.450989102880	0.319015120305
N49	N	0.376608049687	0.992981249577	0.786379833803
N50	N	0.636188890928	0.490612176492	0.263806888604
N51	N	0.400668378683	0.287059105413	0.647917465156
N52	N	0.598556391842	0.794633697095	0.396714209111
N53	N	0.298117209230	0.264025291570	0.600287847991
N54	N	0.698809331321	0.770639881028	0.444011279522
N55	N	0.257329392008	0.136599132272	0.631645703679
N56	N	0.739244824691	0.643001236540	0.412536118479
N57	N	0.315926976652	0.004464228250	0.620268432312
N58	N	0.684354655598	0.511355451345	0.428597966512
N59	N	0.368954294497	0.827842044279	0.645415680869
N60	N	0.639784539936	0.328274698817	0.408228632458
N61	N	0.474139835227	0.810516374101	0.715436174179
N62	N	0.538018169035	0.305139315614	0.338241810309
N63	N	0.558013994303	0.695774591347	0.703161266033
N64	N	0.447534179793	0.194474408287	0.347081836213
N65	N	0.571174439886	0.688645294901	0.632290530353
N66	N	0.429783366830	0.189406933093	0.417409063505
N67	N	0.613081552350	0.833800128976	0.623775580624
N68	N	0.392053045458	0.336992687797	0.426288785850
N69	N	0.723823057593	0.817469488110	0.657833363715
N70	N	0.284353211502	0.326318379943	0.389232508658
N71	N	0.789511013516	0.710268011822	0.616389254415
N72	N	0.212848252499	0.218364111841	0.426306939069
N73	N	0.790973720947	0.808730249909	0.561498430449
N74	N	0.208660115498	0.308814446556	0.484569442156
N75	N	0.859119641106	0.935563192763	0.580956353265
N76	N	0.148857292047	0.442966332786	0.464570986579
N77	N	0.962198963825	0.892291133505	0.615043747915
N78	N	0.044305404808	0.397107163389	0.429856550143
N79	N	0.956015061998	0.902174324243	0.684427790515
N80	N	0.045553457957	0.403178882410	0.361365374934

N81	N	0.905140062099	0.806660432406	0.760427274925
N82	N	0.087025491919	0.297039126788	0.287144330507
N83	N	0.810923077840	0.675182363854	0.803406978166
N84	N	0.174064852295	0.160882990978	0.242881676719
N85	N	0.804116988755	0.627006420390	0.887908456550
N86	N	0.185920997196	0.115929162419	0.160789309824
N87	N	0.768660678423	0.494207213712	0.952229743657
N88	N	0.234196550781	0.002031669369	0.091896185188
N89	N	0.791219720600	0.552447054358	0.017473570627
N90	N	0.199413043545	0.038470054330	0.026738814068
N91	N	0.812054907449	0.720830255744	0.068770474075
N92	N	0.175297614622	0.212150954036	0.981481419587
N93	N	0.918217060047	0.794931964684	0.063679081551
N94	N	0.072052829028	0.288299572009	0.983821815960
N95	N	0.015559136291	0.856171087354	0.011750199439
N96	N	0.970342304232	0.349878954069	0.030200403117
N97	N	0.140999886065	0.790320731158	0.963795669648
N98	N	0.842761238506	0.289083967466	0.075410683986
N99	N	0.295933438856	0.860789116852	0.963052199682
N100	N	0.693096218665	0.369478848027	0.085895741079
N101	N	0.330866652906	0.889219419281	0.896684504455
N102	N	0.660058310869	0.367643321748	0.154802419319
N103	N	0.226868734705	0.906570481276	0.861403459911
N104	N	0.768855313427	0.396986692590	0.186033457450
N105	N	0.180642911382	0.046277929308	0.876063368688
N106	N	0.812482227343	0.543009283000	0.172010427783
N107	N	0.115003137240	0.171707489821	0.864680988499
N108	N	0.894491497113	0.657513276077	0.181187035233
N109	N	0.198812656737	0.323691320507	0.850314069255
N110	N	0.817824964849	0.812458780386	0.197607291468
O1	O	0.768112167378	0.779790456872	0.897747982614
O2	O	0.222875723795	0.269957540121	0.153199720216
O3	O	0.739822106898	0.841190785146	0.004029396113
O4	O	0.280302165936	0.294449312092	0.050925242328
O5	O	0.834013260654	0.000879028994	0.889916377557
O6	O	0.151999735450	0.478099186172	0.148855379954
O7	O	0.721234236691	0.032393399165	0.849107870723
O8	O	0.282498355898	0.512867111661	0.194103449912
O9	O	0.962451739818	0.913219932739	0.801123753768
O10	O	0.026893681791	0.396824953918	0.242268228354
O11	O	0.112271959529	0.077069693142	0.809743526009
O12	O	0.884570748696	0.565922950438	0.237578180369
O13	O	0.258628142783	0.113144106618	0.756330466267
O14	O	0.743275994627	0.605230068068	0.294686653953
O15	O	0.075745909995	0.255540968200	0.739187874687
O16	O	0.927887410780	0.744471783471	0.307095669367
O17	O	0.162646867739	0.243262556057	0.674643008881
O18	O	0.839526198778	0.742151138767	0.372871151610
O19	O	0.001695159366	0.424222694570	0.721163867512
O20	O	0.003866399441	0.907396364498	0.321855692958
O21	O	0.957026090485	0.360690643702	0.641230890887
O22	O	0.047472482290	0.853545643858	0.406287543916
O23	O	0.889836650655	0.211155290654	0.672516852164
O24	O	0.116466878068	0.706277020127	0.374964066681
O25	O	0.798457576436	0.332205754291	0.718758308607
O26	O	0.207068425166	0.825224889994	0.327993023414
O27	O	0.745241633751	0.421748819660	0.652845703621
O28	O	0.258310258214	0.922520753008	0.390334881986
O29	O	0.908437948093	0.553543369972	0.692152671478
O30	O	0.084807092699	0.039259508705	0.353216853940
O31	O	0.702939479273	0.258194505924	0.612595046091
O32	O	0.301172047119	0.748133312889	0.436072823462
O33	O	0.865740158160	0.387636525630	0.560912053570

O34	O	0.200902698794	0.904563381848	0.498388022754
O35	O	0.618408038506	0.199051392840	0.675825590160
O36	O	0.386854794431	0.696877126522	0.371434460619
O37	O	0.526930494269	0.353798521793	0.679740181734
O38	O	0.475062821892	0.854908371871	0.368979649258
O39	O	0.650094225557	0.322048515223	0.779507795319
O40	O	0.358431490808	0.817035039511	0.268989473109
O41	O	0.490267591390	0.324244148244	0.596633111778
O42	O	0.510769827360	0.828810896802	0.450709552479
O43	O	0.450721565940	0.137210435051	0.600026811531
O44	O	0.554015019674	0.640501266877	0.444971739667
O45	O	0.343649303827	0.204973172165	0.676160785057
O46	O	0.655934814490	0.714772730567	0.367411471787
O47	O	0.243726832779	0.326269852705	0.638525408175
O48	O	0.755018597492	0.830662173585	0.405702742080
O49	O	0.175279382056	0.129038953615	0.596586684951
O50	O	0.806565174953	0.620011253724	0.455111924847
O51	O	0.262251209692	0.945419146908	0.660180452098
O52	O	0.747887459989	0.446101948272	0.395766657813
O53	O	0.401780034573	0.938679111499	0.664542565267
O54	O	0.606072299122	0.434821700491	0.385131520722
O55	O	0.388986513495	0.019571984487	0.566597711710
O56	O	0.604979548316	0.527098218922	0.481058114802
O57	O	0.497093727105	0.816621860292	0.659681301755
O58	O	0.512356000214	0.312136593353	0.393583844445
O59	O	0.629330442701	0.785946316525	0.699784580732
O60	O	0.380951425266	0.289527382207	0.350445037656
O61	O	0.541404300499	0.801047943924	0.836415589083
O62	O	0.493164587855	0.291479360014	0.219476777852
O63	O	0.518751960647	0.921236587390	0.840934163277
O64	O	0.526226547230	0.407357383152	0.212090290161
O65	O	0.667671854791	0.652783341933	0.642404817385
O66	O	0.333388157959	0.154738773002	0.406129672797
O67	O	0.663615855696	0.793968939701	0.577977964408
O68	O	0.336890274799	0.296963801344	0.470388590257
O69	O	0.759240878825	0.915565742602	0.628245257904
O70	O	0.248219465515	0.424558953999	0.418473622134
O71	O	0.870085096320	0.779969095301	0.631605419465
O72	O	0.137937796904	0.297811737683	0.412470291141
O73	O	0.876063486684	0.752107741169	0.543697903188
O74	O	0.116592199699	0.260060679285	0.494454564030
O75	O	0.730628842418	0.636954271004	0.561779682767
O76	O	0.265296682569	0.139770081605	0.481021142579
O77	O	0.884885893150	0.946662591488	0.525766346122
O78	O	0.106507226568	0.439846007052	0.516257781990
O79	O	0.795983846022	0.132219956617	0.434523896355
O80	O	0.216126055702	0.640438246848	0.609494627996
O81	O	0.997977210808	0.010387908720	0.616971485918
O82	O	0.000792759354	0.508718821259	0.441694857972
O83	O	0.857347615982	0.992344528382	0.650084188297
O84	O	0.144732206900	0.490487607977	0.394219941617
O85	O	0.038713056464	0.828366706652	0.691229587305
O86	O	0.964001034404	0.326756965539	0.356637065796
O87	O	0.907523057074	0.772959284393	0.705422136334
O88	O	0.095095110405	0.270799620093	0.343038812447
O89	O	0.834919271012	0.794623584420	0.815975447105
O90	O	0.154749301832	0.282343521215	0.231900525240
O91	O	0.815754749882	0.546843588087	0.844308982554
O92	O	0.192437884225	0.028160278978	0.201846479067
O93	O	0.738549121320	0.613184379359	0.945695057210
O94	O	0.254196345079	0.120946115244	0.105257476335
O95	O	0.690386323641	0.540583701531	0.027977537735
O96	O	0.296579801980	0.054972739187	0.011341209993

O97	O	0.764561551178	0.702187962767	0.017729107046
O98	O	0.213323404723	0.183736653925	0.033415955255
O99	O	0.871368019605	0.906927923747	0.058267042243
O100	O	0.119642969816	0.400484527419	0.984620036997
O101	O	0.034181963825	0.738805777159	0.029721149602
O102	O	0.949352983658	0.233723469138	0.011307701819
O103	O	0.971495182994	0.799943438905	0.126234614584
O104	O	0.029572982874	0.306777838792	0.917960069633
O105	O	0.141683050704	0.866551496857	0.008931391529
O106	O	0.847622790364	0.388642621808	0.040957631497
O107	O	0.211250950908	0.897589962825	0.934491002701
O108	O	0.781423716137	0.387673912490	0.114909090243
O109	O	0.369940827734	0.001333731975	0.911566122791
O110	O	0.646679731775	0.491949924549	0.153041122358
O111	O	0.274344038273	0.916604779320	0.811307641748
O112	O	0.726762798612	0.399587237223	0.237739621680
O113	O	0.319096303848	0.743491801568	0.875686807143
O114	O	0.699452146486	0.215897862574	0.169030953628
O115	O	0.367112393840	0.707493434926	0.829038238525
O116	O	0.645004955224	0.162070959979	0.209886697317
O117	O	0.095907906121	0.980589660098	0.888442415534
O118	O	0.895068208522	0.477560379029	0.155919932512
O119	O	0.189460650076	0.678111117886	0.735518721583
O120	O	0.811220799962	0.179018120604	0.312306864943
O121	O	0.123438763174	0.208484248503	0.919109126017
O122	O	0.880457545230	0.696393674998	0.127252457524
O123	O	0.993656788429	0.159883289645	0.858998289649
O124	O	0.018528260194	0.639835109880	0.183408660050
O125	O	0.216055891924	0.234175819506	0.811758164740
O126	O	0.802638388687	0.722009863316	0.236075468329
O127	O	0.981810868098	0.273655086041	0.838100383499
O128	O	0.033857626050	0.752866476728	0.204421802353
O129	O	0.020920447105	0.583856199636	0.504344572065
O130	O	0.000886665045	0.798685489580	0.551357681108
O131	O	0.008274022299	0.948612265699	0.536403840048
O132	O	0.053646674085	0.066554093595	0.495597986785
O133	O	0.023865969095	0.188771122812	0.456912072343
O134	O	0.993638139165	0.331632340939	0.494902808577
O135	O	0.460512347420	0.521796717912	0.161894859135
O136	O	0.555273655738	0.792133853442	0.206501035530
O137	O	0.561278594134	0.938546141602	0.189545120764
O138	O	0.496149018095	0.069851169486	0.192960368591
O139	O	0.497405600723	0.206825803460	0.159119625053
O140	O	0.513069433511	0.386513944777	0.145465813165
O141	O	0.737447233474	0.047316347749	0.925722074042
O142	O	0.246555673191	0.551948284276	0.112929733815
O143	O	0.270647643737	0.103797202282	0.827700625050
O144	O	0.724203696641	0.605044420089	0.225103008270
O145	O	0.356938994796	0.190408666595	0.860936206816
O146	O	0.606888951117	0.652405223939	0.212824431751
O147	O	0.270752580479	0.386052266607	0.742456393783
O148	O	0.734478160591	0.881837032836	0.300993038721
O149	O	0.216107449938	0.434600901946	0.684309109976
O150	O	0.788765279556	0.932781265869	0.359049756386
O151	O	0.224044540800	0.586737773087	0.671841370919
O152	O	0.778380803318	0.083033814373	0.371572233698
O153	O	0.306679943521	0.630277343857	0.719526742868
O154	O	0.693537929040	0.127621568275	0.318390030677
O155	O	0.334482936317	0.508411793919	0.760351680438
O156	O	0.674068507901	0.002416914649	0.276827801825
O157	O	0.688670799380	0.288551357021	0.842749913730
O158	O	0.331603991788	0.791038008244	0.204582468326
O159	O	0.868465969447	0.552315906644	0.757634229283

O160	O	0.135076082318	0.043366152115	0.290206213021
O161	O	0.746679865646	0.554727719959	0.748118209514
O162	O	0.258632509323	0.046154782356	0.298833820098
O163	O	0.718202572925	0.548224702835	0.682520308598
O164	O	0.271190032505	0.060311426082	0.365836732280
O165	O	0.982604794288	0.150035517718	0.583981621307
O166	O	0.024820291370	0.733367433663	0.486927099877
O167	O	0.545568150526	0.241198236829	0.772168355190
O168	O	0.499078830455	0.822072115572	0.266001221468
O169	O	0.473655729744	0.352287742642	0.742498040875
O170	O	0.568382819714	0.847869327225	0.323830307767
O171	O	0.458110294977	0.488957527091	0.763147780886
O172	O	0.560632822893	0.985147493361	0.299145790847
O173	O	0.328720800732	0.248680396048	0.745183830165
O174	O	0.673365302092	0.745849983795	0.297442845629
O175	O	0.090605123566	0.161993473248	0.547350131439
O176	O	0.898514464459	0.734480030959	0.474176423189
O177	O	0.121891851425	0.995501906620	0.625123499233
O178	O	0.882470647501	0.520528419227	0.418844713127
O179	O	0.234510439394	0.812837492930	0.689884149752
O180	O	0.767933544512	0.312515629163	0.361707246857
O181	O	0.276178547235	0.027055514940	0.527332576098
O182	O	0.716795571819	0.534979507987	0.511238946669
O183	O	0.390308668594	0.781896801205	0.765325524469
O184	O	0.622478721009	0.250325421778	0.293935671484
O185	O	0.284029380881	0.840413028312	0.748944183561
O186	O	0.719426659011	0.337024323204	0.301493842148
O187	O	0.471052904553	0.634771624701	0.746628462528
O188	O	0.544353022154	0.130589817894	0.314598720517
O189	O	0.483298077543	0.054644482712	0.814946896802
O190	O	0.519149606561	0.565400970228	0.234824876733
O191	O	0.483683202677	0.673580662966	0.821233057784
O192	O	0.539639952594	0.156952367536	0.243350273729
O193	O	0.603691972090	0.796963482900	0.897734355122
O194	O	0.398999223452	0.339359134335	0.149620092511
O195	O	0.130480575802	0.728884587029	0.677756908036
O196	O	0.872775101079	0.227900101114	0.369763415997
O197	O	0.958008527096	0.675844262376	0.661499095882
O198	O	0.045169185540	0.161257099043	0.386319416900
O199	O	0.931953559301	0.519343071542	0.816503333168
O200	O	0.078204291741	0.004472329702	0.232256087211
O201	O	0.731487664889	0.432723238242	0.851842677955
O202	O	0.291562395496	0.933727194950	0.186374805874
O203	O	0.901034745439	0.484580945857	0.995790421564
O204	O	0.099588884595	0.966155885982	0.054537194200
O205	O	0.027220566490	0.487802411350	0.016841845079
O206	O	0.984115044012	0.004306839222	0.028995603579
O207	O	0.945234548194	0.639690532958	0.062033605886
O208	O	0.019298940710	0.117975907180	0.987883483337
O209	O	0.979241553116	0.571240817588	0.124588494854
O210	O	0.024483947201	0.089631397287	0.917720346251
O211	O	0.067754304382	0.564061412577	0.075128539547
O212	O	0.901013007934	0.118125220382	0.927652208786
O213	O	0.331466850091	0.770492378013	0.028823554039
O214	O	0.620996620528	0.276772640643	0.043442576058
O215	O	0.256993014525	0.612783571151	0.878653881783
O216	O	0.737742078240	0.073674137368	0.154855810818
O217	O	0.321809633958	0.568307516871	0.822501046424
O218	O	0.667474161614	0.017877243501	0.207008911436
O219	O	0.996874769985	0.413247536850	0.856847863269
O220	O	0.018227994720	0.895855275848	0.192831247192
O221	O	0.110402799773	0.416437014489	0.888066849735
O222	O	0.903929552377	0.904119809711	0.163051575185

O223	O	0.087318664702	0.572869866433	0.900134361581
O224	O	0.871989735767	0.062646272240	0.159415517047
O225	O	0.605669016926	0.338991302136	0.887860675754
O226	O	0.430979377051	0.675028213265	0.066398321033
O227	O	0.361244198235	0.699625073487	0.941240774949
O228	O	0.356134891834	0.201355767248	0.004853843879
O229	O	0.435813585557	0.576033293870	0.930814115356
O230	O	0.551413570107	0.911651916726	0.022150560856
O231	O	0.326270482618	0.530726629534	0.917182267045
O232	O	0.560494146287	0.254224950370	0.106811091285
O233	O	0.475456709208	0.684581943677	0.891008220913
O234	O	0.547400201152	0.363127972379	0.006978497553
O235	O	0.452097784246	0.053681827731	0.095397461036
O236	O	0.433093551946	0.868734280447	0.033936209447
O237	O	0.396240672924	0.013020935335	0.158435843044
O238	O	0.544113052264	0.722688539959	0.945119603421
O239	O	0.625973925325	0.919023382360	0.076571695785
O240	O	0.616072634833	0.061264499701	0.877900720956
O241	O	0.547555763491	0.259456525091	0.942404415380
O242	O	0.386620482600	0.776017068186	0.112023638596
O243	O	0.450316587926	0.110147284950	0.937382084013
O244	O	0.509595055580	0.720211219805	0.019505729089
O245	O	0.575956863426	0.099751010178	0.088529533056
O246	O	0.362959157159	0.357426006851	0.933905622276
O247	O	0.439243874430	0.909207986989	0.114229559243
O248	O	0.558030336851	0.562543162954	0.076610553086
O249	O	0.472375379239	0.196811978617	0.881107398294
O250	O	0.385008544256	0.160712056780	0.067084643883
O251	O	0.639564872709	0.766088185065	0.131468441835
O252	O	0.681645625792	0.003217038468	0.982610268054
O253	O	0.564214812633	0.553324271661	0.906349184248
O254	O	0.489623208148	0.636861510985	0.122588043056
O255	O	0.313823932765	0.642472886876	0.996772486420
O256	O	0.667715895890	0.777811187386	0.063537808092
O257	O	0.352845888766	0.198592028328	0.929735163983
O258	O	0.694911632189	0.176229197920	0.980065196637
O259	O	0.611926968274	0.790200627575	0.002579250409
O260	O	0.556076651553	0.880455536982	0.128962684593
O261	O	0.489788793438	0.127818549206	0.002970317042
O262	O	0.606495823025	0.131233988346	0.936917335086
O263	O	0.518591641885	0.425570203095	0.067282649328
O264	O	0.432340515301	0.790410222631	0.977444975539
O265	O	0.558878228049	0.166118379297	0.835847382166
O266	O	0.290382352258	0.532985184218	0.042784490516
O267	O	0.442778320244	0.762432880748	0.176877878258
O268	O	0.412551888397	0.009619412817	0.019065616102
O269	O	0.557425097969	0.934081779594	0.902708214083
O270	O	0.329688988936	0.340036303264	0.864606627949
O271	O	0.457620240881	0.838410969039	0.916163349378
O272	O	0.595828368767	0.421214066857	0.949816394271
O273	O	0.482119495381	0.242657138675	0.054472912989
O274	O	0.607624367916	0.132582351347	0.023591965103
O275	O	0.754532445800	0.970317115280	0.043040397677
O276	O	0.389545326978	0.475536635568	0.862513351502
O277	O	0.299696183174	0.680446120687	0.085785032542
O278	O	0.395015998103	0.479243529887	0.070319982526
O279	O	0.689065511184	0.038512106383	0.095598376069
O280	O	0.357819335321	0.474744970571	0.132576752428
O281	O	0.482046644619	0.347123213575	0.878624253409
O282	O	0.306278679191	0.459671066008	0.977424370004
O283	O	0.450091709751	0.283030231786	0.977132604497
O284	O	0.470295831486	0.432655015850	0.930752501058
O285	O	0.601072692607	0.635137410077	0.147307312647

```
O286   O     0.550306449618   0.580028820399   0.010501879163
O287   O     0.423611398325   0.444619770291   0.994988426698
O288   O     0.563872068541   0.006902477902   0.967407415948
O289   O     0.423179586067   0.586516856727   0.009124262978
O290   O     0.669953183180   0.633184086679   0.077892834078
O291   O     0.622041087609   0.613312151445   0.960284495513
O292   O     0.610985562322   0.472199884311   0.858314221571
O293   O     0.392686975640   0.346990637090   0.043402003588
O294   O     0.498331182384   0.516070714283   0.829887440251
O295   O     0.391214359104   0.635174058701   0.204182262490
O296   O     0.447820625944   0.686786231235   0.262524086238
O297   O     0.436830421502   0.915033327174   0.221164003455
O298   O     0.452787447051   0.018302960489   0.269686055889
O299   O     0.377460900350   0.316581172938   0.216111875432
O300   O     0.393305971434   0.470070794590   0.217540428620
S1   S     0.941351103191   0.850691100809   0.914571646238
S2   S     0.042493632907   0.316776860735   0.132962021345
S3   S     0.903097185098   0.102800769454   0.764277483404
S4   S     0.097964425046   0.591988483542   0.276566221533
S5   S     0.579722694863   0.036458206068   0.584971187223
S6   S     0.428763650376   0.538735078525   0.464456989352
S7   S     0.552652196222   0.993982066447   0.631161291304
S8   S     0.455436871585   0.496801600903   0.418147566243
S9   S     0.901445118690   0.051396585294   0.717273896802
S10   S     0.104799932424   0.546179432377   0.324713247361
S11   S     0.960363124356   0.937757835074   0.947186047034
S12   S     0.041388806464   0.401191067882   0.097608385108
#END
```
